# Mediation analysis reveals common mechanisms of *RUNX1* point mutations and *RUNX1/RUNX1T1* fusions influencing survival of patients with acute myeloid leukemia

**DOI:** 10.1038/s41598-018-29593-2

**Published:** 2018-07-26

**Authors:** Roman Hornung, Vindi Jurinovic, Aarif M. N. Batcha, Stefanos A. Bamopoulos, Maja Rothenberg-Thurley, Susanne Amler, Maria Cristina Sauerland, Wolfgang E. Berdel, Bernhard J. Wörmann, Stefan K. Bohlander, Jan Braess, Wolfgang Hiddemann, Sören Lehmann, Sylvain Mareschal, Karsten Spiekermann, Klaus H. Metzeler, Tobias Herold, Anne-Laure Boulesteix

**Affiliations:** 1 Institute for Medical Information Processing, Biometry and Epidemiology, LMU Munich, Munich, Germany; 2 German Cancer Consortium (DKTK), partner site Munich, Munich, Germany; 3 German Cancer Research Center (DKFZ), Heidelberg, Germany; 4 Department of Medicine III, University Hospital, LMU Munich, Munich, Germany; 5 Institute of Biostatistics and Clinical Research, University of Muenster, Muenster, Germany; 6 Department of Medicine A, Hematology and Oncology, University of Muenster, Muenster, Germany; 7 German Society of Hematology and Oncology, Berlin, Germany; 8 Department of Molecular Medicine and Pathology, University of Auckland, Auckland, New Zealand; 9 Department of Oncology and Hematology, Hospital Barmherzige Brüder, Regensburg, Germany; 10 Department of Medical Sciences, Uppsala University Hospital, Uppsala, Sweden; 11 Department of Medicine, Karolinska Institute, Stockholm, Sweden; 12 Department of Biosciences and Nutrition, Karolinska Institute, Stockholm, Sweden

## Abstract

Alterations of *RUNX1* in acute myeloid leukemia (AML) are associated with either a more favorable outcome in the case of the *RUNX1/RUNX1T1* fusion or unfavorable prognosis in the case of point mutations. In this project we aimed to identify genes responsible for the observed differences in outcome that are common to both *RUNX1* alterations. Analyzing four AML gene expression data sets (n = 1514), a total of 80 patients with *RUNX1/RUNX1T1* and 156 patients with point mutations in *RUNX1* were compared. Using the statistical tool of mediation analysis we identified the genes *CD109, HOPX*, and *KIAA0125* as candidates for mediator genes. In an analysis of an independent validation cohort, *KIAA0125* again showed a significant influence with respect to the impact of the *RUNX1/RUNX1T1* fusion. While there were no significant results for the other two genes in this smaller validation cohort, the observed relations linked with mediation effects (i.e., those between alterations, gene expression and survival) were almost without exception as strong as in the main analysis. Our analysis demonstrates that mediation analysis is a powerful tool in the identification of regulative networks in AML subgroups and could be further used to characterize the influence of genetic alterations.

## Introduction

Alterations of the Runt-related transcription factor (*RUNX1)* have been observed to strongly influence the outcome of patients with acute myeloid leukemia (AML)^1^. In the case of missense or frameshift mutations, *RUNX1* has a negative effect, that is, the affected patients have a considerably shorter survival compared to those without these mutations. In contrast, the *RUNX1*/*RUNX1T1* fusion, in the following denoted as t(8; 21), is associated with a more favorable outcome in comparison to patients without fusions.

The observed influences of mutations and fusions on survival are not fully direct, but can be assumed to be in part due to transcriptional deregulation of other genes resulting from alterations of the transcription factor *RUNX1*. The latter means that the considered alterations have an effect on the expression of these genes and, at the same time, they themselves have an effect on survival independent of the alteration. However, it is not known which genes play an important role here. We were particularly interested in identifying genes with altered expression that contribute to the strong effect of *RUNX1* mutations and fusions on survival outcome. Such genes cannot be identified through standard differential expression analyses (e.g., using the limma approach^2^) by simply comparing expression levels in the altered group to those in the non-altered group, because this approach is not appropriate to identify genes with an effect on survival. On the other hand, identifying genes associated with survival (e.g., using Cox regression models) does not allow finding genes whose expression levels are affected by the considered alteration. We aim at identifying genes that *both* are affected by alterations and affect the survival outcome. Such questions can be addressed by “mediation analysis” methods that allow testing whether variables (e.g. genes) are affected by an exposure and, at the same time, have an influence on the outcome. While statistical procedures for mediation analyses are widely applied in general, they have not often been used in gene expression analyses. To our knowledge, there exists only one publication in this context: Huang *et al*.^3^ used a mediation analysis procedure to identify gene expression variables that help explain the influence of micro-RNA expression variables on survival. In general, mediation analyses are concerned with measuring and testing indirect effects of risk factors of interest on target variables through particular *mediator* variables. Such an analysis is well suited for the main goal of our approach - identifying genes through which *RUNX1* mutations and fusions influence the outcome. Given the fact that large gene expression and sequencing data from the same patients are necessary for such an analysis, it is not surprising that mediation analysis has not been applied in this context to date.

After identifying mediator genes with an appropriate mediation analysis approach, we performed several descriptive analyses in order to gain further insights into the role of these genes and on their interplay. In an analysis of an independent validation cohort, one of the three identified genes was again a significant mediator gene with respect to the impact of the *RUNX1/RUNX1T1* fusion. While there were no significant results for the other two genes in this smaller validation cohort, the observed relations linked with mediation effects (i.e., those between alterations, gene expression and survival) were almost without exception as strong as in the main analysis.

## Methods

Using the terminology of mediation analysis, in the following, the influential genes will be termed “mediator genes” or “mediators”. The mutation and the fusion will be called the exposure.

### Patient cohorts and gene expression data

Four data sets were included in our main analysis, to which we will refer to as AMLCG Cohort 1, HOVON, TCGA and AMLCG Cohort 2. The AMLCG Cohort 1 consisted of 488 patients treated on the AMLCG-1999 trial of the German AML Cooperative Group (GSE37642)^4–6^. The HOVON cohort had 462 patients treated on various trials of the Dutch Haemato-Oncology Cooperative Study Group (HOVON). Clinical and gene expression data are publicly available (GSE14468)^7,8^. The TCGA cohort was composed of 179 AML samples published by The Cancer Genome Atlas Project^9^. For the TCGA cohort, we used the corrected clinical data published with Data Release 9.0 on October 24, 2017. AMLCG Cohort 2 had 260 patients treated on the AMLCG-2008 and the AMLCG-1999 trials^10^. The gene expression data is publicly available (GSE106291). After excluding patients with missing data and non-intensive induction treatment (TCGA), the following case numbers were obtained: n = 469 (AMLCG Cohort 1), n = 461 (HOVON), n = 100 (TCGA), and n = 252 (AMLCG Cohort 2) (Supplementary Fig. S1). For all variables with missing values, missingness can be assumed to be independent from the missing values themselves and also to be independent from the values of other variables in the data sets. Each data set contains several ten thousand gene expression variables, either measured by Affymetrix Arrays (AMLCG Cohort 1 and HOVON) or RNA sequencing (RNAseq, TCGA and AMLCG Cohort 2). The microarray cohorts were preprocessed with the RMA (Robust Multichip Average) method^11^, and the raw counts created by RNASeq were preprocessed using the R-package DESeq2^12^. All study protocols were in accordance with the Declaration of Helsinki and were approved by the institutional review boards. Informed consent was obtained from all subjects and all experiments were performed in accordance with relevant guidelines and regulations. The analysis was approved by the ethics committee of the medical faculty of the University of Munich.

**Figure 1:**
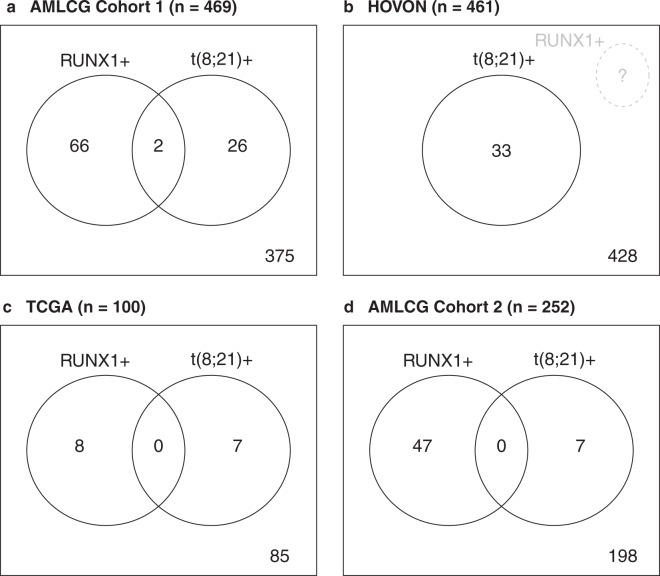
Venn diagrams of case numbers in the data sets. Panels a, b, c, and d show the case numbers available for AMLCG Cohort 1, HOVON, TCGA, and AMLCG Cohort 2, respectively.

In the following, the suffixes “+” and “−” will be added to “*RUNX1*” and “t(8; 21)” to indicate the presence and absence of *RUNX1* point mutations and the *RUNX1/RUNX1T1* fusion, respectively. For better readability, the suffix “+” is omitted in cases where there is no risk of confusion. For each data set, the survival information, that is, survival and censoring times, is available. Information on the number of observations per data set with information on fusions, mutations, and both fusions and mutations, is given in Fig. 1. For AMLCG Cohort 2 genes that were not expressed in more than 90% of samples were excluded from the analysis.

We also used an independent validation cohort^13^. From this validation cohort, we were able to use the data from 232 patients. Information on overall survival as well as mutation and fusion status, sex and age was available for each patient. As gene expression values, we used reads per kilobase per million reads (RPKM) values for these data.

### Analysis outline

The following three comparisons were performed for the selection of influential genes (for details, see further down): (1) patients with and without fusion (t(8; 21)+ vs. t(8; 21)−), (2) patients with and without mutation (*RUNX1*+ vs. *RUNX1*−), (3) patients with fusion only and patients with mutation only (t(8; 21)++ vs. *RUNX1*++). See the next two subsections for a detailed description of the procedures performed for the selection of influential genes.

After determining a set of influential genes for each comparison, we investigated these genes further. For sets resulting from comparisons 1 and 2, we produced heatmaps in order to obtain insights into the relationships between the genes in these sets. Because the set of genes found to be mediators with respect to the survival differences between t(8; 21)+ and *RUNX1*+ (comparison 3) was much smaller than for the other two comparisons and because this set is the most interesting of the three sets as it contains genes relevant to both mutations and fusions, we investigated this set more thoroughly by estimating partial correlations between these genes. Moreover, we produced Kaplan-Meier curves for these genes comparing high and low levels (where the median is used as cutoff) of gene expression not only for the whole patient collectives, but also separately for patients without mutations or fusion, for patients with mutations only, and for patients with fusions only. In order to obtain insights into the biological determinants that drive the differences in outcome between *RUNX1*+ and t(8; 21)+, we performed an over-representation analysis^14^. Lastly, in order to affirm the relevance of the set of genes found to be mediators with respect to the survival differences between t(8; 21)+ and *RUNX1*+ (comparison 3) we analyzed the independent validation cohort.

In all analyses, the primary outcome was overall survival. Unless otherwise stated, *p* values smaller than 0.05 are considered significant (α = 0.05).

### Definition of mediator effects and testing procedure

**Figure 2:**
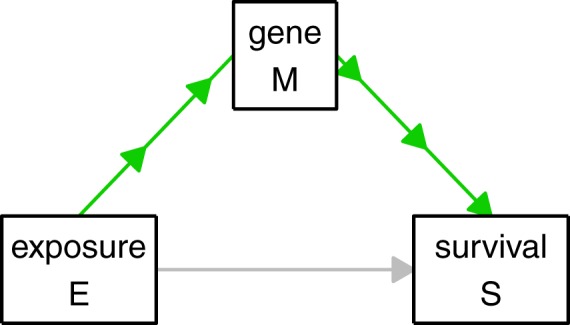
Illustration of mediator effect. The direct effect of E on S is depicted in grey and the mediator effect also known as indirect effect is depicted in green.

Let E be the exposure (i.e., mutation or fusion) and S the survival outcome, which is influenced by E. Then a mediation effect for gene M means the following: the relationship between the survival outcome S and the exposure E is partially or totally due to gene M which is taking the role of a mediator^15^, see Fig. 2. To make the concept of mediation more clear, consider the following fictional example: A low socioeconomic status might be observed to lead to a higher risk of death. Here, the low socioeconomic status would take the role of the exposure. A potential mediator in this context could be ‘nutrition’: A low socioeconomic status might be associated with a less balanced nutrition which in turn leads to higher risk of death. By identifying mediator genes we attempt to explain the strong influence mutations and fusions have on the survival outcome. We consider only those mediator effects which support, not inhibit, the observed influence of the exposure on the survival outcome. For example, as *RUNX1* mutations lead to shorter survival times, we only target those genes as mediators for which the corresponding mediator effect leads to shorter survival times, that is, genes that are more expressed in *RUNX1*+ patients and negatively associated with survival or genes that are less expressed in *RUNX1*+ patients and positively associated with survival. Other mediation effects are ignored in our analysis, since they do not contribute to capturing the survival differences between *RUNX1*+ and *RUNX1*−. We used the testing framework introduced by Lange and Hansen^16^, adjusting for the (possibly) confounding variables sex, age, and cytogenetics. See the Supplementary Information for details on the followed procedure. We implemented our analysis using our new R package ‘survmediation’ (available from GitHub, link: https://github.com/RomanHornung/survmediation) which is based on the original code by Lange and Hansen.

### Pre-selection of promising genes and adjustment for multiple testing

In identifying the relevant genes through testing each gene separately, we faced a multiple testing problem. An adjustment for multiple testing, for example by the Benjamini-Hochberg procedure, for all of the many thousands of tests necessary for each of the three comparisons would have resulted in a very small number of significant genes. Moreover, using the combined data of all four data sets for testing would have resulted in a suboptimal performance of the testing procedure for the following reason: the data behave quite differently in the individual data sets, because of batch effects^17^, in particular, because the data sets have different data types.

As a solution to these problems, we pre-selected promising genes using one data set, termed “pre-selection data set”, and validated this reduced list of genes in another data set, termed “validation data set”. Adjusting for multiple testing was only performed in the “validation data set”. First, for each of the three comparisons successively, using the corresponding pre-selection data set we pre-selected the genes that had a *p* value smaller than 0.05. This pre-selection led to a comparably short list of genes that was expected to contain a high density of true influential genes. Second, as already noted above, these promising genes were tested again – this time using the validation data set. The *p* values from these tests were then adjusted by the Benjamini-Hochberg procedure. By pre-selecting promising genes using independent data sets as described above, the problem of multiple testing was mitigated in the validation data sets, because the number of tests was reduced strongly.

**Figure 3:**
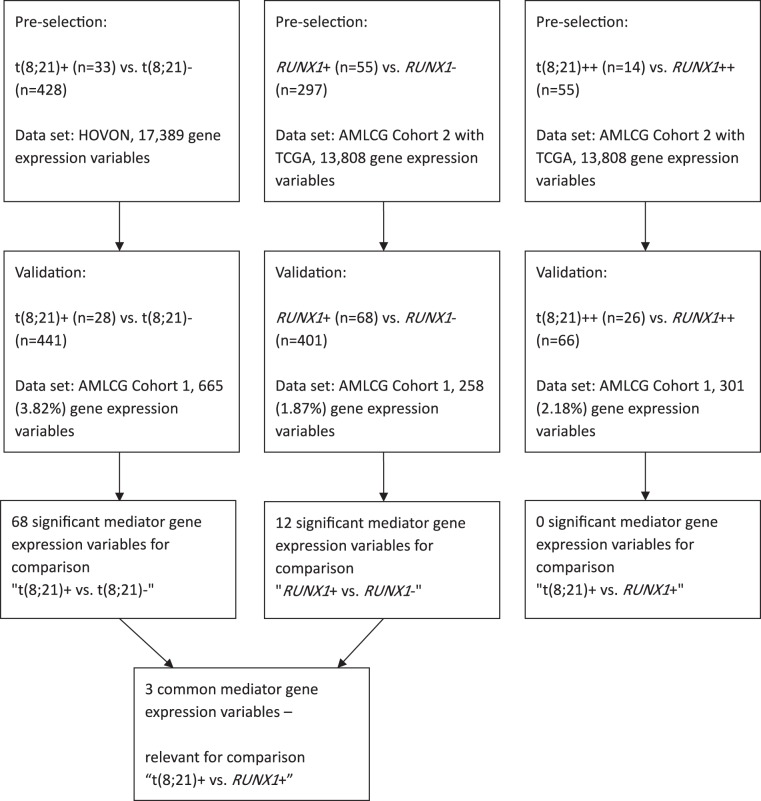
Workflow of mediator selection.

For two of the three comparisons, we used a concatenation of the two data sets AMLCG Cohort 2 and TCGA in the pre-selection. ComBat^18^ was used to remove batch effects between AMLCG Cohort 2 and TCGA in the concatenated data set denoted as “AMLCG Cohort 2 with TCGA” in the following. See Fig. 3 for an overview on the data sets used for the pre-selection and validation for each of the three comparisons and the number of genes selected for each comparison in the pre-selection and validation, respectively. For details on the processing of the pre-selection data sets and the validation data sets, see the Supplementary Information.

### Distinction of mediation analysis from differential expression analysis

Many of the genes identified in this paper as candidates for mediator genes with respect to the influence of *RUNX1* and t(8; 21) on the outcome have been identified before as being differentially expressed with respect to *RUNX1*+ vs. *RUNX1*− ^19^. In this subsection, we demonstrate that, while differential expression is a prerequisite for mediation activity, genes that are differentially expressed are by no means necessarily mediator genes.

If the expression of a gene is influenced by the exposure (e.g. *RUNX1*+ vs. *RUNX1*−), the gene is said to be differentially expressed with respect to the exposure. However, a gene that is differentially expressed does not necessarily have an influence on the patients’ outcome. By contrast, mediation activity of a gene requires the following two conditions to be fulfilled: (1) The gene is differentially expressed with respect to the exposure, that is, its expression is influenced by the exposure; (2) The gene’s expression has an effect on the outcome after adjusting for (or ‘independent of’) the influence of the exposure on the outcome. The latter requirement that the gene should be influential after adjusting for the influence of the exposure on the outcome is a crucial requirement for the existence of mediation activity: If, after adjusting for the influence of the exposure on the outcome, the gene had no influence on the outcome, this would mean that the observed influence of the gene on the outcome would be merely due to the influence of the exposure on the gene (perfect confounding). In this case, the gene itself would have no actual influence on the outcome. Thus, it would not help to explain the influence of the exposure on the outcome, but it would merely be differentially expressed.

We performed the following analysis to demonstrate that it can be expected that the majority of differentially expressed genes show no mediator actitivity. First, we applied linear models using moderated t-statistics^20^ as implemented in the BioConductor package limma^2^ to AMLCG Cohort 1 to select genes differently expressed between *RUNX1*+ and *RUNX1*−. The *p* values obtained for each gene from this analysis were adjusted using the Benjamini-Hochberg procedure. Due to the high number of genes found differentially expressed at the 5% significance level and in order to increase confidence in the relevance of the identified genes, in this analysis, we considered genes with *p* values smaller than 0.01 as significant (α = 0.01), resulting in 1146 significantly differentially expressed genes. Subsequently, for each of these 1146 genes we performed two Cox regressions: (a) using only the gene as covariate for survival and (b) using the gene plus the exposure variable *RUNX1*+ vs. *RUNX1*− as covariates for survival. For each of these regressions, we recorded the *p* value obtained for the regression coefficient of that gene. Again using the Benjamini-Hochberg procedure we corrected these *p* values, for both types of regressions (a) and (b), separately.

**Figure 4:**
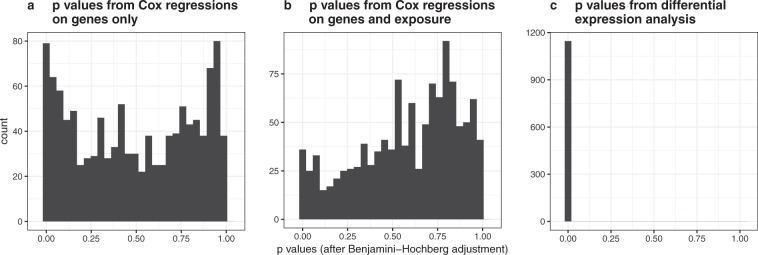
Histograms of *p* values from differentially expressed genes in Cox regression analysis. The left and middle panels show the Benjamini-Hochberg adjusted *p* values of the influences of the genes in Cox regressions performed separately for each gene that used as covariates the corresponding genes (panel a) or the corresponding genes as well as the exposure *RUNX1*+ vs. *RUNX1*− (panel b). Panel c shows the Benjamini-Hochberg adjusted *p* values of the genes found to be differentially expressed at the 1% significance level from the differential expressions analysis performed using limma.

Figure 4 shows the distributions of the *p* values obtained for the regressions (a) and (b), respectively, as well as the *p* values obtained for these genes in the differential expression analysis (which are all smaller than 0.01 due to the selection described above). We make two main observations: (1) The majority of differentially expressed genes are not significantly associated with survival, neither when considered alone (regressions (a)) nor when considered in combination with the exposure (regressions (b)); (2) When considered as covariates in combination with the exposure (regressions (b)), much less genes are significantly associated with survival compared to when the genes are considered as covariates alone (regressions (a)). Observation (1) illustrates the fact described above: Differentially expressed genes do not necessarily affect the patients’ outcome. Observation (2) demonstrates that, when investigating the influence of genes on the outcome, it is crucial to adjust for the influence of the exposure. If this adjustment is not performed, many genes will significantly influence the survival outcome although they do not have a genuine influence on the outcome; in these cases, the significant influence of the gene’s expression on survival is likely due to the gene’s expression values being merely correlated with the outcome, because the exposure has an influence on both the gene’s expression as well as on the outcome. As already described above, only genes that are influenced by the exposure and at the same time influence the outcome when adjusted for the influence of the exposure can help explain the strong influence of the exposure on the outcome, that is, can be considered as candidates for mediator genes.

In the main mediation analysis described in the next section we identified three genes as candidates for mediators with respect to the observed differences in outcome of patients with *RUNX1* point mutations and those with *RUNX1/RUNX1T1* fusions. These three genes were all among the differentially expressed genes identified in this subsection. Moreover, their influences were significant in both Cox regressions (a) and (b) described above (results not shown). This demonstrates that genes that show mediator activity are both influenced by the exposure and at the same time have an influence on the outcome, even after adjusting for the influence of the exposure on the outcome. Separately investigating two regression models, one measuring the influence of the exposure on the mediator and one measuring the influence of mediator and exposure on the outcome as performed in this subsection corresponds to the traditional approach to mediation analysis. This approach has, however, been shown to be flawed in many cases^21^. Modern analyses mainly use approaches based on the so-called counterfactual framework^21^. The approach by Lange and Hansen used in our analyses is as well based on this framework.

### Availability of data and materials

The TCGA data is publicly available in processed form through https://cancergenome.nih.gov/. The HOVON and AMLCG data sets are available through the Gene Expression Omnibus (GEO) website (GSE37642, GSE14468, GSE106291).

The complete R code written to perform and evaluate the analyses is available in the Electronix Appendix to this article.

## Results

### Overall influence of *RUNX1* mutations and t(8; 21) on survival

**Figure 5:**
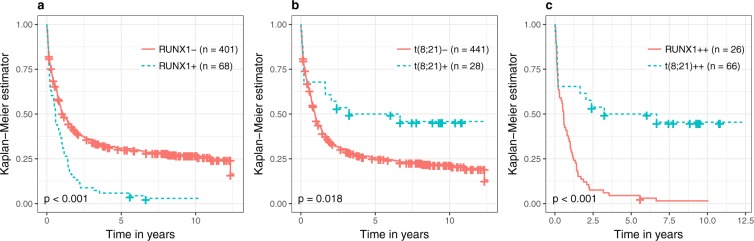
Survival differences between patients with and without *RUNX1* and t(8; 21), respectively. Panel a shows two Kaplan Meier curves, separately for patients with *RUNX1*+ and *RUNX1*−, panel b two Kaplan Meier curves, separately for patients with t(8; 21)+ and t(8; 21)−, and panel c two Kaplan Meier curves, separately for patients with *RUNX1*++ and t(8; 21)++ treated within the AMLCG Cohort 1. Censorings are indicated as plus signs. The *p* values are the results of log-rank tests used to test for survival differences between the two groups associated with the respective Kaplan Meier curves.

*RUNX1* mutations (n = 123) and fusions (n = 75) were almost mutually exclusive in all three data sets which had information on *RUNX1* and t(8; 21). This is in line with previous analyses showing that these alterations define specific genetic groups in AML^22,23^. Figure 5 shows the observed influence of *RUNX1* mutations and fusions on the survival function. Clearly, patients with *RUNX1* mutations have a very unfavorable outcome, while t(8; 21) positive patients have a more favorable prognosis.

### Selection of influential genes

For the comparison t(8; 21)+ vs. t(8; 21)−, 665 (3.82%) genes had *p* values smaller than 0.05 in the pre-selection (see Fig. 3). Testing these 665 genes using the corresponding validation data set, 68 genes were still significant after adjustment for multiple testing (Supplementary Table S1).

For the comparison *RUNX1*+ vs. *RUNX1*−, 258 (1.87%) genes were pre-selected (see Fig. 3). After testing these pre-selected genes using the validation data set and adjusting for multiple testing, 12 genes remained significant (Supplementary Table S2).

A total of 301 (2.18%) genes were pre-selected for the last comparison t(8; 21)++ vs. *RUNX1*++ (see Fig. 3 and Supplementary Information). When testing these genes using the validation data set, no significant results were obtained after adjustment for multiple testing. Thus, in this direct comparison, we could not identify genes associated with the differing survival behaviors of patients with t(8; 21) and *RUNX1*. Another possibility of identifying such genes is to compare Supplementary Tables S1 and S2. Genes occurring in both tables are associated with both t(8; 21) and *RUNX1* and are therefore also responsible for the differing survival behaviors between patients with t(8; 21) and *RUNX1*. The following three genes are present in both tables: *CD109, KIAA0125*, and *HOPX*. The fact that no genes were identified in the t(8; 21)++ vs. *RUNX1*++ comparison can probably be attributed to the small sample size. Note that one of the three genes that occur in both tables, namely *HOPX*, is regulated by *RUNX1* (see the ORegAnno Database^24^). Given that we could not identify any significant candidates for mediator genes in the direct comparison t(8; 21)++ vs. *RUNX1*++, we decided to perform a further, more robust, mediation analysis for this direct comparison. In this analysis, we considered only genes used in the LSC17 prognostic score^25^, because these genes are known to have prognostic relevance and might thus be promising candidates for mediator genes for t(8; 21)++ vs. *RUNX1*++. This additional analysis is more robust than the main analysis presented above, because, due to the much smaller number of genes considered in this additional analysis, the effect of the adjustment for multiple testing is much less severe. In this analysis, the following three genes were significant after adjustment for multiple testing: *GPR56*, *KIAA0125*, and *NGFRAP1*. Note that *KIAA0125* was also in the previous analysis among the three genes identified as candidates for mediation activity with respect to t(8; 21)++ vs. *RUNX1*++. The other two of the three genes discussed before, *CD109* and *HOPX*, were not considered in the current analysis, because these two genes are not among the genes featured in LSC17 that were studied here. Note that in performing this analysis we have used the same data a second time, which is why we attribute a lower priority to the two genes *GPR56* and *NGFRAP1* which were identified only in this analysis than to the three genes identified in the main analysis. See the Supplementary Information for details on the design and the results of this analysis. In the subsection ‘Distinction of mediation analysis from differential expression analysis’ we discussed the conceptual differences between mediation analysis and differential expression analysis using, for example, limma. In order to illustrate that, while mediator genes are differentially expressed, differentially expressed gene are not (necessarily) mediator genes, we performed an additional analysis: We applied limma to select differentially expressed genes, using AMLCG Cohort 2 with TCGA as pre-selection data set (*p* value threshold for pre-selection: 0.05) and AMLCG Cohort 1 as validation data set, again adjusting the *p* values obtained on the validation data set using the Benjamini-Hochberg procedure. Adjusting for sex, age, and cytogenetics as in the mediation analysis we obtained the following results: 3078 (22.29%) genes were pre-selected and 1317 (9.54% from all 13808 genes tested) of these 3078 genes were significant after Benjamini-Hochberg adjustment in the validation analysis (Supplementary Information). All five candidate genes *CD109*, *HOPX*, *KIAA0125*, *GPR56*, and *NGFRAP1* were among these 1317 genes selected by limma. This demonstrates that only a very small proportion of differentially expressed genes identified by limma can be expected to be mediator genes. This illustrates that limma is not suitable for detecting mediator genes. Note that mediation activity of a gene means that this gene is differentially expressed and - at the same time - that its expression influences the outcome independently from the influence the exposure (e.g., t(8; 21)++ vs. *RUNX1*++) has on the expression of that gene.

### Descriptive analyses of the selected genes

First, in order to quantify the prognostic relevance of the three genes selected in both comparisons, t(8; 21)+ vs. t(8; 21)− and *RUNX1*+ vs. *RUNX1*−, we performed a univariate Cox proportional hazards regression analysis for each gene using the validation data set AMLCG Cohort 1. Before applying Cox regression, we centered and scaled the log2 transformed expression values of the three genes to have mean zero and variance one in order to make comparisons of the values of the hazard ratio possible. The results of the Cox regression are shown in Supplementary Table S4. The values of the hazard ratio and that of Harrell’s concordance index are quite similar across genes, with *CD109* having the highest values. While the values of the concordance index (range: 0.552–0.587) would be considered small in the context of evaluating prediction models, they can be regarded as large in this analysis, because here we do not evaluate multivariable predictive models, but consider the predictive performance of individual genes. More than 98% of the concordance indices obtained for the other 17,384 gene expression variables in AMLCG Cohort 1 were smaller than the smallest of the concordance indices obtained for the three genes considered here.

Next, in order to detect possible subgroups among the genes selected for comparisons t(8; 21)+ vs. t(8; 21)− and *RUNX1*+ vs. *RUNX1*−, respectively, we created heatmaps for both of these two comparisons, clustering the genes using the agglomerative hierarchical clustering approach. For the genes selected from comparison t(8; 21)+ vs. t(8; 21)−, we created a heatmap for both HOVON and AMLCG Cohort 1, because these data sets were used in the pre-selection and validation for this comparison (see Fig. 3). For AMLCG Cohort 1, we only considered samples with both t(8; 21)− and *RUNX1*−. For the HOVON cohort, we considered the samples with t(8; 21)− (the samples had no information on the mutation status in this data set). Supplementary Figs S2 and S3 show the heatmaps obtained for the data sets HOVON and AMLCG Cohort 1, respectively. For both data sets, the genes cluster into two quite distinct groups of similar size. The k-means clustering method with k = 2 found similar clusters as agglomerative hierarchical clustering (with complete linkage and Euclidean distance) in both data sets (adjusted Rand index: HOVON: 0.83, AMLCG Cohort 1: 0.72). Thus, the two clusters seem to be quite stable within the two data sets. However, the two largest clusters found using hierarchical clustering differ to a stronger degree between the two data sets (adjusted Rand index: 0.27) than do the clusterings generated with the two clustering methods within the same data sets. The clusterings obtained for the two data sets differ also with respect to the cluster memberships of the three genes selected in both comparisons (t(8; 21)+ vs. t(8; 21)− and *RUNX1*+ vs. *RUNX1*−): the gene *HOPX* is in the same cluster as *CD109* for the HOVON data set and in the same cluster as *KIAA0125* for AMLCG Cohort 1.

In the case of the genes selected for comparison *RUNX1*+ vs. *RUNX1*−, the combined data set AMLCG Cohort 2 with TCGA and AMLCG Cohort 1 were used to create heatmaps (see Supplementary Figs S4 and S5). These two data sets were used for pre-selection and validation, respectively, for this comparison (Fig. 3). For both data sets only the samples with both t(8; 21)− and *RUNX1*− were used. In the case of AMLCG Cohort 2 with TCGA, there are again two large clusters, where the three genes selected in both comparisons are contained in the same cluster. In the case of AMLCG Cohort 1, however, the 12 considered genes seem to cluster into three distinct groups. Again, as in Supplementary Fig. S3, *HOPX* and *KIAA0125* are in the same cluster, whereas *CD109* is in a different cluster, which is, however, not surprising since the results shown in Supplementary Figs S3 and S5 were obtained using the same data set.

**Figure 6:**
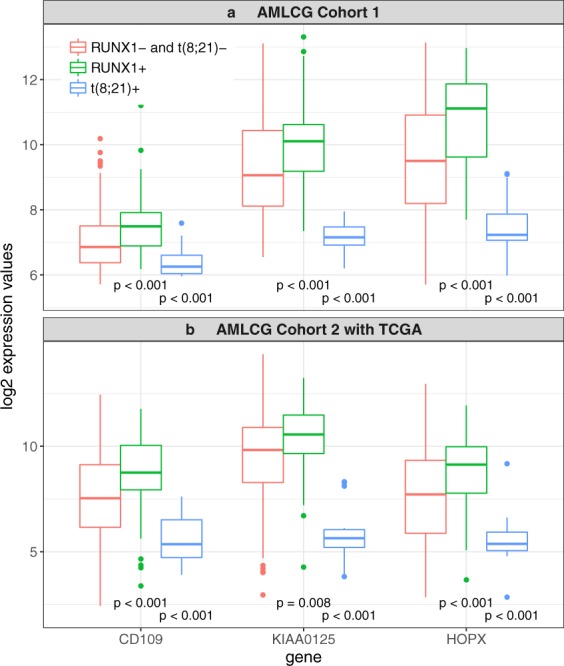
Log2 expression values for the three genes selected as mediators both for the *RUNX1*+ vs. *RUNX1*− comparison and the t(8; 21)+ vs. t(8; 21)− comparison. Log2 expression values in AMLCG Cohort 1 (panel a) and AMLCG Cohort 2 with TCGA (panel b) for the three genes that appear in both, Supplementary Tables S1 and S2, separated by whether only mutations (*RUNX1*+), only fusion (t(8; 21)+) or neither of these two (*RUNX1*− and t(8; 21)−) are present in the respective patients. The *p* values are the results of Wilcoxon tests. For each gene, we tested ‘*RUNX1*+’ against ‘*RUNX1*− and t(8; 21)−’ and ‘t(8; 21)+’ against ‘*RUNX1*− and t(8; 21)−’.

Figure 6 shows the expression values of *CD109, KIAA0125*, and *HOPX* (data sets AMLCG Cohort 1 and AMLCG Cohort 2 with TCGA) separated by whether mutations, fusions, or neither of these two were present in the samples. Quite apparently, *RUNX1* mutations are associated with higher and the t(8; 21) with lower expression levels for all three genes. For each of these genes, higher expression values are associated with a poorer prognosis.

**Figure 7:**
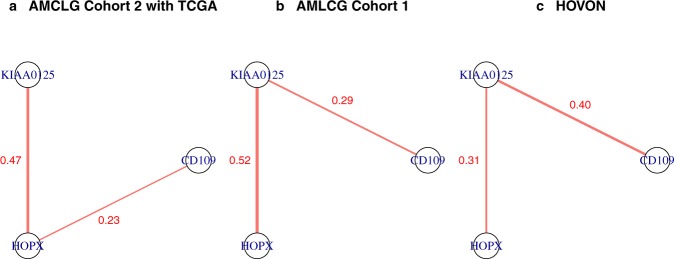
Partial correlations between the expression levels of the three genes selected both for *RUNX1*+ vs. *RUNX1*− and t(8; 21)+ vs. t(8; 21)− estimated using AMLCG Cohort 2 with TCGA (panel a), AMLCG Cohort 1 (panel b), and HOVON (panel c), respectively. Only partial correlations larger than 0.2 are shown. The strengths of the partial correlations are reflected by the widths of the lines. In the case of the data sets AMCLG Cohort 2 with TCGA and AMLCG Cohort 1 all patients with *RUNX1*− and at the same time t(8; 21)− were used. In the HOVON data set all patients with t(8; 21)− were used (the information on the presence of *RUNX1*+ was not given for this data set).

Figure 7 shows the partial correlations between the expression levels of these three genes for each of the three data sets. For all three data sets, there is a partial correlation larger than 0.2 between *KIAA0125* and *HOPX*, where the partial correlation between these genes is stronger for AMLCG Cohort 2 with TCGA and AMLCG Cohort 1 than for HOVON. Note that HOVON was also the only data set for which *KIAA0125* and *HOPX* were not in the same cluster. Moreover, there is a partial correlation larger than 0.2 between *KIAA0125* and *CD109* for both AMLCG Cohort 1 and HOVON and a partial correlation larger than 0.2 for AMLCG Cohort 2 with TCGA.

**Figure 8:**
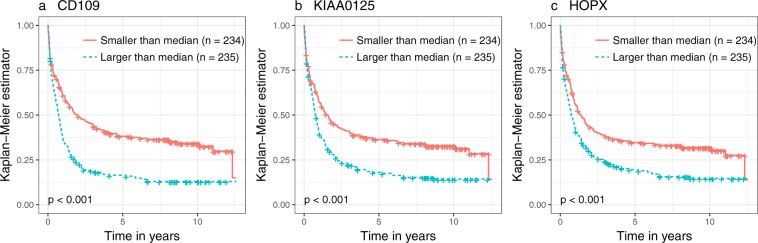
Survival differences between patients with high and low gene expression values. Each subplot shows the Kaplan Meier curves of patients with expression values higher/lower than the median expression value for one of the three genes (panel a: *CD109*, panel b: *KIAA0125*, panel c: *HOPX*) selected from both the *RUNX1*+ vs. *RUNX1*− comparison and the t(8; 21)+ vs. t(8; 21)− comparison. In each case, the data set AMLCG Cohort 1 was used. Censorings are indicated as plus signs. The *p* values are the results of log-rank tests used to test for survival differences between the two groups associated with the respective Kaplan Meier curves.

Interestingly, the prognostic effect of patients with high and low expression values of the three mediator genes was not limited to patients with *RUNX1* mutations or fusions. Figure 8 shows Kaplan Meier curves comparing the survival outcomes of all patients with high and all patients with low expression values of the three genes selected for both comparison “*RUNX1*+ vs. *RUNX1*−” and “t(8; 21)+ vs. t(8; 21)−” (AMLCG Cohort 1 data set). Moreover, Supplementary Fig. S6 shows corresponding Kaplan Meier curves for the subgroup of patients without *RUNX1* mutations or fusions. These Kaplan Meier curves suggest that these genes also influence survival for patients without *RUNX1* mutations or the t(8; 21) fusion.

### Over-representation analysis

Above we have determined lists of genes that contribute to the strong observed influences of *RUNX1* mutations and fusions on survival. It would be desirable to additionally identify mechanisms for the differences in outcome between *RUNX1*+ and t(8; 21)+ that are better interpretable than are lists of genes. We therefore performed an over-representation analysis with the aim of identifying specific annotation sets associated with the outcome difference of *RUNX1* mutations and fusions.

We identified candidate drivers separately for the comparisons *RUNX1*+ vs. *RUNX1*− and t(8; 21)+ vs. t(8; 21)−. For each of these two comparisons we considered those genes in the over-representation analysis that were significant to the significance level 0.10 on the validation data sets before adjustment for multiple testing. The reason why we chose 0.10 as significance level here instead of 0.05 as in the rest of the paper, was that by choosing a high significance level more candidates for potential drivers can be expected to be obtained. Note that while increasing the significance level would be considered as fishing for significance in other situations, this is not problematic in the analysis at hand, because, as explained below, the considered analysis flow is of a mere explorative nature. This selection resulted in 62 genes for the comparison *RUNX1*+ vs. *RUNX1*− and 252 genes for the comparison t(8; 21)+ vs. t(8; 21)−, respectively (Supplementary Information). These two lists were analyzed with ConsensusPathDB^14^. Subsequently, we determined those drivers from the identified annotation sets that were common to both lists. We identified the gene ontology-based sets: GO:0010876 lipid localization and GO:0005548 phospholipid transporter activity as common and enriched in both comparisons.

Note that these drivers should be interpreted with caution. They can merely be considered in an informal discussion on potential drivers for the large differences in outcome between patients with *RUNX1*+ and those with t(8; 21)+. The reason for this limited interpretability of the identified drivers is the fact that the considered analysis flow is of explorative nature. This analysis flow did not allow us to perform statistical testing in the strict sense, that is, the identified drivers cannot be declared as “significant”. As they are the result of a descriptive analysis, their relevance must be validated in future studies before drawing valid conclusions from these findings.

### Validation of the candidate mediator genes in an independent cohort

Using the validation cohort described in the subsection ‘Patient cohorts and gene expression data’, we re-performed the mediation analysis for the selected genes *CD109, KIAA0125*, and *HOPX*. From the 232 patients available, 33 had a *RUNX1* mutation and five a *RUNX1*/*RUNX1T1* fusion. Mutations and fusions were mutually exclusive.

In order to test for mediation activity of the three genes, again we employed the testing framework introduced by Lange and Hansen using the same setup as in the main analysis, adjusting for sex and age. Given the comparably small sample size of the validation cohort, we only considered the comparisons *RUNX1*+ vs. *RUNX1*− and t(8; 21)+ vs. t(8; 21)−, not the comparison t(8; 21)++ vs. *RUNX1*++; for the latter comparison only 38 patients would have been available (t(8; 21)+: n = 5; *RUNX1*+: n = 33). This choice was also motivated by the fact that in the main analysis we were not able to find any significant genes in the direct comparison t(8; 21)++ vs. *RUNX1*++.

In the comparison *RUNX1*+ vs. *RUNX1*− only the gene *KIAA0125* was significant (p = 0.014), whereas the genes *CD109* (p = 0.076) and *HOPX* (p = 0.096) were merely ‘weakly significant’ (i.e., significant at the level α = 0.10). In the comparison t(8; 21)+ vs. t(8; 21)− none of the three genes were significant or ‘weakly significant’ (*KIAA0125*: p = 0.121, *CD109*: p = 0.351, *HOPX*: p = 0.176). Note, however, that only five patients with t(8; 21)+ were available for the comparison t(8; 21)+ vs. t(8; 21)−. In a comparison, in which one of the two groups is that small, it is unlikely to obtain significant results even when the actual effects are strong. Also, for the comparison *RUNX1*+ vs. *RUNX1*− we had much less observations available than in the main analysis – in the validation cohort there were less than half as many observations with *RUNX1*+ and *RUNX1*−, respectively, than there were available in the main analysis for validation using AMLCG Cohort 1.

Beyond statistical significance – which highly depends on sample size, the magnitudes of the effects observed in the validation cohort are of crucial importance. Supplementary Fig. S7 shows the (standardized) RPKM values of the three genes, separated by whether *RUNX1* mutations, fusions, or neither of these two are present in the samples (cf. also Fig. 6 for the corresponding values obtained for the data used in the main analysis). For each gene, the RPKM values differ strongly between these groups, where the differences between patients with mutations and fusions are particularly strong. Supplementary Fig. S8 shows for all patients without mutations or fusions from the validation cohort, Kaplan Meier curves comparing the survival outcomes of patients with high and patients with low RPKM values with respect to the three considered genes. These curves give a very similar picture as those obtained in the main analysis (cf. Supplementary Fig. S6): The three genes seem to influence survival also for patients without *RUNX1* mutations or the t(8; 21) fusion. Moreover, higher RPKM values are associated with higher risks for each of the three genes.

To summarize: The sample sizes available in the validation cohort were much smaller than those available in the main analysis, in particular for the comparison t(8; 21)+ vs. t(8; 21)−, which leads to a lower power of the tests on mediation activity. Against this background, a (strictly) significant result was only obtained for *KIAA0125* with respect to the comparison *RUNX1*+ vs. *RUNX1*−. However, the descriptive analysis (Supplementary Figs S7 and S8) of the validation cohort was indicative that these three genes indeed show mediation activity with respect to the survival differences observed between patients with *RUNX1* mutations and fusions, respectively.

## Discussion

Using mediation analysis we identified three genes that may help to explain the strong differences in outcome between AML patients with *RUNX1* mutations and *RUNX1/RUNX1T1* fusions. We aimed to validate our results in a smaller, independent validation cohort. In this analysis, one of the three genes, *KIAA0125*, was again significant with respect to playing a mediating role in the influence *RUNX1/RUNX1T1* fusions have on survival. While the other investigated influences associated with the three genes were not significant, we note that the observed effects were almost without exception as strong as in the main analysis. Thus, the lack of significant results obtained using the independent validation cohort may well be due to the small sample sizes available for this analysis.

In the main analysis we used one data set for pre-selecting promising candidate genes and one data set for testing the candidate genes from the pre-selection. For testing the candidate genes in the second step, we used the largest data set AMLC Cohort 1, because for the pre-selection (i.e., the first step) less statistical power was required, since in this step we merely filtered out genes that showed little indications for mediation activity. As a sensitivity analysis, it would have been possible to change the roles of the data sets used for the pre-selection and the validation, in order to verify, whether we would have identified the same genes if the roles of pre-selection and validation data sets had been switched. However, we decided not to perform this kind of sensitivity analysis. It is known that variable selection using high-dimensional data is highly dependent on the data used for the selection^26,27^. Moreover, the variables identified in such analyses are usually only a small subset of the actually relevant variables. Thus, we expect that we would have obtained different selected genes in the sensitivity analysis described above, where both sets (this new one and the one we identified in our analysis) contain relevant genes but do not fully overlap. However, reporting all these genes as significant would lead to accumulating false positive results (the global significance level is 0.05 for one repetition of the analysis only - it inflates when several repetitions are performed).

The transcription factor *RUNX1* plays a crucial role in the hematopoietic system and is essential for differentiation^28^. Alterations of *RUNX1* (*RUNX1* mutations and *RUNX1/RUNX1T1* fusion) are observed in about 20% of AML patients and therefore belong to the most common genetic abnormalities associated with AML^23^. This frequency and the fact that *RUNX1* mutations and the *RUNX1/RUNX1T1* fusion are almost never observed in the same patient suggest that alterations of *RUNX1* play an important role in leukemogenesis and that *RUNX1/RUNX1T1* fusion and *RUNX1* mutations are competing events. In addition, t(8; 21) positive leukemias have a specific set of cooperating mutations (e.g. tyrosine kinase and RAS pathway activations) which differ from *RUNX1* mutated leukemias that show a different set of cooperating mutations^23,29^. Furthermore, patients harboring a *RUNX1* fusion are usually of younger age than patients having *RUNX1* mutations^19,30^. We were interested in the question, whether, despite these differences, there is a common ground that connects both types of leukemia which dramatically differ in their clinical behavior. Our analysis aimed to elucidate these relationships by the analysis of gene expression data from 1514 AML patients. By applying a mediation analysis and performing validation analyses of our results in different independent data sets that used different techniques to measure gene expression, we identified three genes that show strong indications for association with the observed difference in prognosis in the considered data, suggesting a common mechanism and connecting both types of *RUNX1* alterations. Higher expression of each of these genes was associated with inferior prognosis in intensively treated AML patients. Interestingly, we could demonstrate that these three genes show predictive potential even in patients without *RUNX1* mutations or *RUNX1* fusions. Hence, *CD109, KIAA0125*, and *HOPX* seem to be involved in or are surrogates of cellular processes that reflect a more aggressive disease independent of the fact that their expression levels might be directly affected by *RUNX1* alterations in AML. In subsequent analyses we were able to identify indications for specific cellular processes like lipid localization and phospholipid transporter activity associated with this difference.

One of the identified genes, namely *HOPX*, has been shown to be a direct target of the *RUNX1* transcription factor^24^. It is therefore tempting to speculate that expression levels of this gene are affected by the mutated *RUNX1* protein or the *RUNX1* fusion protein. The regulation of the other candidates seems to be more complex. Interestingly, *CD109* and *KIAA0125* (*FAM30A*) were previously identified by other groups to have prognostic relevance in cancer. High expression of *CD109* is associated with unfavorable prognosis in diffuse large B-cell lymphoma (DLBCL), soft tissue sarcoma and several other cancers^31–33^. *CD109* codes a glycosyl phosphatidylinositol (GPI)-linked cell surface glycoprotein and regulates TGF-β signaling^32,33^. Since *CD109* is also expressed on platelets and is associated with alloimmune thrombocytopenia, it may be a potential therapeutic target^34^. The gene *KIAA0125*, a long non-coding RNA (lnc RNA), was shown to be involved in migration and invasion in gallbladder cancer^35^.

Mediation analysis is an analysis technique for testing whether variables of interest are mediators in the observed influence of certain, usually binary variables on the outcome. In our analysis the potential mediators were the gene expression variables, the influencing variables were the mutation and the fusion (presence vs. absence) and the outcome was overall survival. In this context, mediation analysis can be viewed as an explorative statistical approach towards large-scale systems biology. Due to the generality of mediation analysis, such approaches may be applied in any settings in the context of gene expression analysis, in which the interest lies in identifying genes that play a role in the observed influence of exploratory (binary) variables on the outcome. Mediation analysis is not restricted to survival data: There exist corresponding methods to that of Lange and Hansen^16^ for other outcome types as well.

As stated in the introduction, only a single publication that uses mediation analysis in gene expression analysis seems to exist. In this regard our work is pioneering, which is why we placed a great focus on describing the methodology we used in performing our analyses. Mediation analysis offers great potential to address important, still unanswered questions in the area of hematologic malignancies (and beyond). Researchers who have access to respective data sets may use our analysis design as a template for their own analyses. In doing so, they may obtain new insights in hematologic malignancies that are beyond the scope of traditional analysis approaches such as differential expression analysis using limma. We leave it to the interested reader to choose research questions that can be tackled using mediation analysis.

Note, however, that mediation analysis does not *per se* prove the *causal* character of relationships between exposure and mediator and between mediator and outcome adjusted for the influence of the exposure on outcome. Mediation analysis is based on regression models. Whether the effects identified in these regression models reflect causal mechanisms, and if so whether these causal effects are direct, should be further investigated. For example, the expression of a gene might itself not be a causal factor influencing survival, but can instead be associated with survival because it is associated with a causal factor. Mediation analysis cannot clarify such issues. It should thus be seen as an exploratory approach suggesting potential candidates for causal relationships.

Mediation analysis methods with high-dimensional molecular data, as used in our study, are still in their infancy from a statistical point of view. In the future, methodological developments may improve its performance, for example with respect to the multiple testing procedures or to the simultaneous consideration of a large number of candidate biomarkers within a multivariable regression modeling framework. For the special case of a metric outcome variable, such a multivariable method has been published recently^36^. However, multivariable mediator methods for survival outcomes still have to be developed. Methodological comparison studies are also needed to systematically assess mediation analyses approaches.

In summary, our analysis identified three gene expression markers that might help in explaining the observed prognostic difference between patients with *RUNX1* mutations and the *RUNX1/RUNX1T1* fusion. The expression levels of these genes exhibit prognostic potential also in patients without alterations of *RUNX1* and point towards an oncogenic cooperative network. Further research is required to elucidate the specific role of these genes in AML.

## Electronic supplementary material

Supplementary Information

Electronic appendix
