# Supplementary material for: Mediation analysis reveals common mechanisms of RUNX1 point mutations and RUNX1/RUNX1T1 fusions influencing survival of patients with acute myeloid leukemia
Source: Sci Rep. 2018 Jul 26;8:11293. doi: 10.1038/s41598-018-29593-2 (PMC6062501; doi:10.1038/s41598-018-29593-2)
Supplement: Supplementary file 1 — Supplementary Information [file 41598_2018_29593_MOESM1_ESM.pdf]

Supplementary Information

Mediation analysis reveals common mechanisms of *RUNX1* point mutations and *RUNX1*/*RUNX1T1* fusions influencing survival of patients with acute myeloid leukemia

Roman Hornung[1], Vindi Jurinovic[1], Aarif M. N. Batcha[1,2,3], Stefanos A. Bamopoulos[4], Maja Rothenberg-Thurley[4], Susanne Amler[5], Maria Cristina Sauerland[5], Wolfgang E. Berdel[6], Bernhard J. Woermann[7], Stefan K. Bohlander[8], Jan Braess[9], Wolfgang Hiddemann[2,3,4], Sören Lehmann[10,11], Sylvain Mareschal[10,12], Karsten Spiekermann[2,3,4], Klaus H Metzeler[2,3,4], Tobias Herold[2,3,4,§] and Anne-Laure Boulesteix[1,§]

1 Institute for Medical Information Processing, Biometry and Epidemiology, LMU Munich, Munich, Germany; 2 German Cancer Consortium (DKTK), partner site Munich; 3 German Cancer Research Center (DKFZ), Heidelberg, Germany; 4 Department of Internal Medicine III, LMU Munich, Munich, Germany; 5 Institute of Biostatistics and Clinical Research, University of Münster, Münster, Germany; 6 Department of Medicine A, Hematology and Oncology, University of Münster, Münster, Germany; 7 German Society of Hematology and Oncology, Berlin, Germany; 8 Department of Molecular Medicine and Pathology, University of Auckland, Auckland, New Zealand; 9 Department of Oncology and Hematology, Hospital Barmherzige Brüder, Regensburg, Germany; 10 Department of Medical Sciences, Uppsala University Hospital, Uppsala, Sweden; 11 Department of Medicine, Karolinska Institute, Stockholm, Sweden; 12 Department of Biosciences and Nutrition, Karolinska Institute, Stockholm, Sweden

§ Contributed equally

Corresponding author: Roman Hornung, PhD; Marchioninistr. 15; 81377 München; Germany;

Phone: +49 89 4400-74497; Email: hornung@med.uni-muenchen.de

Details on the procedure followed to test for mediation effects

In order to adjust for the (possibly) confounding variables sex, age, and cytogenetics, we considered these variables as baseline covariates, both in the regression model that relates the mediator to the exposure and in the regression model that relates survival to the mediator (see Lange and Hansen[1] for details on these two models). The influences of exposure and mediators were modeled time-independent, because the estimation of time-independent influences is generally much more robust as opposed to the estimation of time-dependent influences. The robustness of the time-independent model is associated with a greater power of the corresponding tests. The advantage of time-dependent effects that the true dependencies can be better rendered is important for descriptive analyses, but less in this analysis in which the greater power achieved from using time-independent effects is much more important. We used the simulation approach by Lange and Hansen[1] to calculate *p* values. This approach is more accurate than the less computationally intensive alternative presented in the same paper that uses a delta rule approximation.

Pre-selection data sets and validation data sets

As validation data set, for all three comparisons we chose AMLCG Cohort 1. For the comparison t(8;21)+ vs. t(8;21)- we used the HOVON as pre-selection data set. The AMLCG Cohort 1 data had 17,389 gene expression variables that were all contained in the HOVON data set, the latter having 19,204 gene expression variables. Thus we used the 17,389 gene expression variables common to both data sets for this comparison. For both, the *RUNX1*+ vs. *RUNX1*- and t(8;21)++ vs. *RUNX1*++ comparison we combined the TCGA and the AMLCG Cohort 2 data set to obtain a pre-selection data set. After combining the two data sets, ComBat[2] was used to remove batch effects. Combining the data sets enabled us to use a much larger sample size for the pre-selection. Here, 13,808 gene expression variables appeared both in the pre-selection data set as well as in the AMLCG Cohort 1 data set. Consequently, these 13,808 gene expression variables were used in the respective comparisons. The combined pre-selection data set is referred to as “AMLCG Cohort 2 with TCGA”. We note that combining different data sets for analysis, as performed in our analysis in the cases of the pre-selections for the comparisons *RUNX1*+ vs. *RUNX1*- and t(8;21)++ vs. *RUNX1*++, can lead to less precise results than if using a single data set of the same size as the combined data set. In the case of the comparison t(8;21)+ vs. t(8;21)- a large (single) data set, the HOVON data set, was available for the pre-selection, which is why, for this comparison, we did not deem it necessary to use a combined data set.

Mediation analysis for the t(8;21)++ vs. *RUNX1*++ comparison of genes used in LSC17

The LSC17 is a prognostic score for AML patients that features 17 genes[3]. It seems interesting to study, whether some of these genes show indications for mediation activity with respect to the t(8;21)++ vs. *RUNX1*++ comparison. In the main analysis presented in the paper, we were not able to identify any candidates for mediator genes in this direct comparison of *RUNX1* point mutations and *RUNX1/RUNX1T1* fusions. As also stated in the paper, the latter can likely be explained by the limited sample size available for that comparison.

In this supplementary section, we present the results of an additional mediation analysis in which we only considered genes that are featured in LSC17. This restriction to only a few promising genes greatly alleviates the multiple testing problem in comparison to the main analysis. Due to this restriction, only a small number of *p* values have to be adjusted for multiple testing instead of – as in the main analysis - hundreds of *p* values. Moreover, given the prognostic power of LSC17, it seems much more likely that there are mediator genes among the genes considered in this prognostic score than among genes for which there is no known (strong) relation to the outcome of AML patients. Given the small number of considered genes and the small sample size in this analysis, we did not use a part of the data for pre-selecting promising genes here. Instead, we combined the data sets AMLCG Cohort 1 and AMLCG Cohort 2 with TCGA to a single data set in order to increase the statistical power of the analysis. Again, ComBat was used to remove batch effects between these two data sets. This combined data set featured 16 of the 17 genes considered in LSC17. Using the combined data, each of these 16 genes was tested for mediation activity with respect to the t(8;21)++ vs. *RUNX1*++ comparison. For this task, the testing framework by Lange and Hansen was employed in exactly the same way as described in section 'Definition of mediator effects and testing procedure' of the paper. The *p* values from these tests were again adjusted for multiple comparisons using the Benjamini-Hochberg procedure. The results are presented in Supplementary Table S3. Three of the 16 genes show a

statistically significant mediation activity in this analysis: *GPR56*, *KIAA0125*, and *NGFRAP1*. Also in the main analysis presented in the paper, *KIAA0125* was among the three genes identified as candidates for mediation activity with respect to t(8;21)++ vs. *RUNX1*++. The other two of the three genes discussed in the paper, *CD109 and HOPX*, were not considered in the current analysis, because these two genes are not among the 16 genes from LSC17 studied here.

Summarizing, by considering a more robust analysis flow in this supplementary subsection, we were able to identify two further genes that are promising with respect to mediation activity for the comparison t(8;21)++ vs. *RUNX1*++. Note, however, that we used the same data sets, AMLCG Cohort 1 and AMLCG Cohort 2 with TCGA, for this analysis as for the main analysis. Thus, there is a higher danger that the results obtained from this analysis are over-optimistic than there would be, if we would have had independent data available for this second analysis.

Supplementary Tables

Variable	BH-adjusted *p* value
FAM92A1	<0.001
FADS1	<0.001
PHGDH	0.001
CD109	0.001
GALNT12	0.001
GPR56	0.001
CLU	0.001
DDIT4	0.001
B3GNT1	0.001
NGFRAP1	0.002
DOCK1	0.002
NRBP1	0.003
SH3BP4	0.003
IRX1	0.003
WBP5	0.003
KIAA0125	0.003
SYNJ2	0.004
F12	0.007
DLC1	0.007
HDAC4	0.010
MICALL2	0.011
DNMT3B	0.011
ZC3H12C	0.013
HOPX	0.017

Variable	BH-adjusted *p* value
ADRM1	0.031
ATP13A2	0.034
CALCRL	0.035
ADRBK1	0.035
KDELC1	0.035
C16orf93	0.035
MBTPS1	0.035
ZNF334	0.035
SCHIP1	0.035
SEL1L3	0.035
BEX2	0.035
GOLGA8A	0.036
IGF2BP3	0.037
TPK1	0.037
BIVM	0.037
NYNRIN	0.037
MPO	0.037
PCTP	0.037
NCRNA00205	0.037
HTR1F	0.038
CYB5A	0.038
RFTN1	0.038
TRAT1	0.040
TSC22D4	0.040

Variable	BH-adjusted *p* value	Variable	BH-adjusted *p* value
GPR114	0.017	*CCNG2*	0.042
GC18M052797_at	0.017	*RAB27A*	0.042
TANC1	0.018	*YPEL2*	0.042
ACOT7	0.019	*TMEM163*	0.042
TM4SF1	0.026	*WDR54*	0.043
MMRN1	0.026	*PCCA*	0.043
ALDH2	0.026	*PYGB*	0.043
ARHGAP22	0.027	*KIAA1274*	0.044
SAP30L	0.028	*HMGN5*	0.048
HOMER3	0.028	*KCTD15*	0.048

Supplementary Table S1: Significant mediator genes for the comparison t(8;21)+ vs. t(8;21)- Genes which also appear in Supplementary Table S2 are printed in bold.

Variable	BH-adjusted *p* value
C10orf58	<0.001
BEND4	0.001
CD109	0.002
SCRN1	0.005
IFITM3	0.015
KIAA0125	0.015
SLITRK5	0.020
HOPX	0.022
CHRDL1	0.036
FAM171B	0.038
FHL1	0.038
CCDC85C	0.040

Supplementary Table S2: Significant mediator genes for the comparison *RUNX1*+ vs. *RUNX1*- Genes which also appear in Supplementary Table S1 are printed in bold.

Variable	BH-adjusted *p* value
GPR56	0.040
KIAA0125	0.040
NGFRAP1	0.040
DPYSL3	0.081
MMRN1	0.081
NYNRIN	0.210
ARHGAP22	0.673
DNMT3B	0.693
CDK6	0.795
EMP1	0.795
SOCS2	0.795
AKR1C3	0.979
CD34	0.979
LAPTM4B	0.979
ZBTB46	0.979
CPXM1	0.992

Supplementary Table S3: Results of the additional mediation analysis performed for the comparison t(8;21)++ vs. *RUNX1*++ in which only LSC17 genes were considered Genes with significant results are printed in bold.

	Hazard ratio	Harrel's concordance index
CD109	1.312	0.587
KIAA0125	1.281	0.552
HOPX	1.259	0.556

Supplementary Table S4: Results of univariate Cox proportional hazards regression analyses using AMLCG Cohort 1 Note that we do not report *p* values here, because these genes were determined to be significant mediators and mediators influence the outcome by definition.

Supplementary Figures

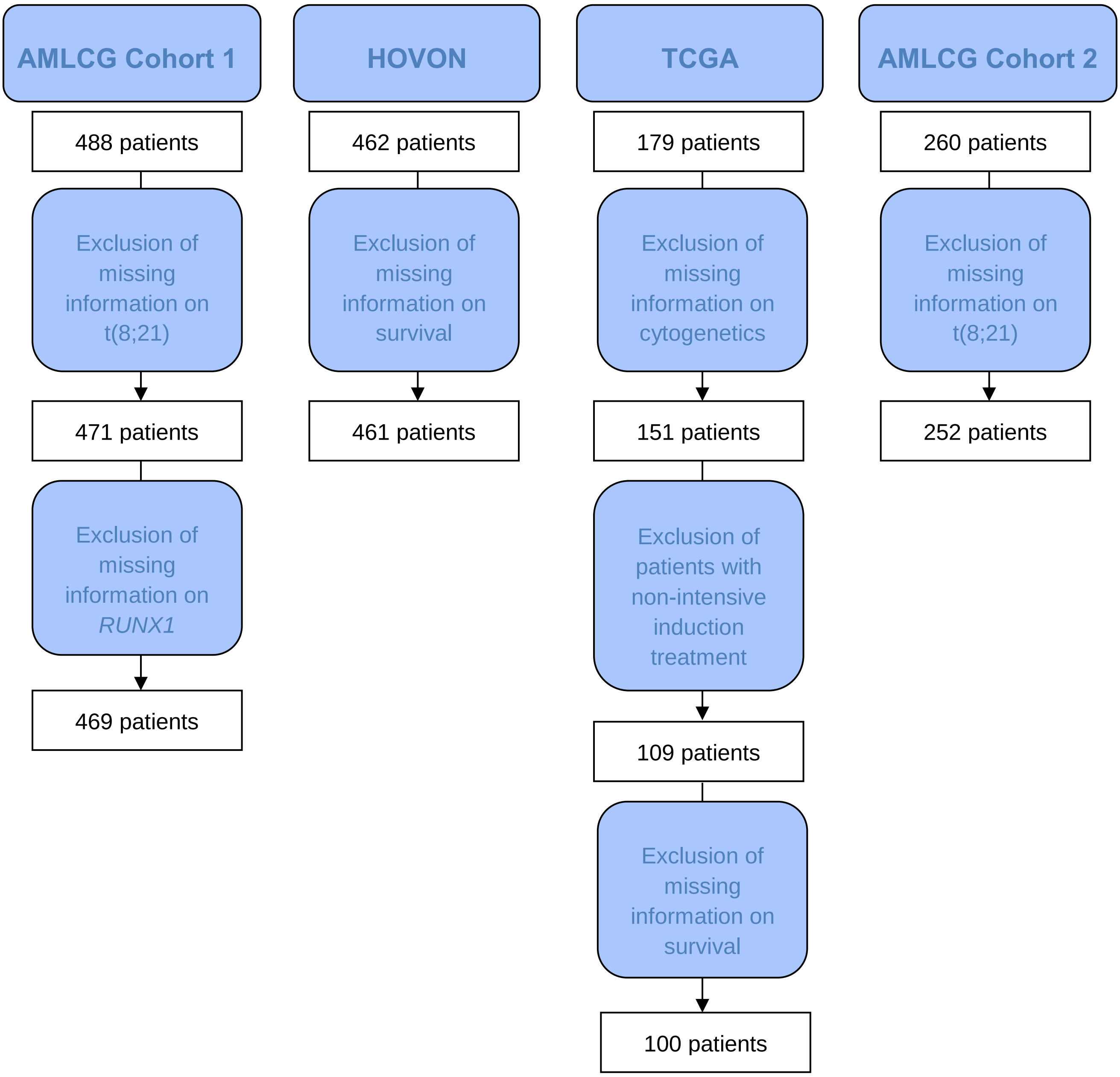

Supplementary Figure S1: Reasons for exclusion of patients

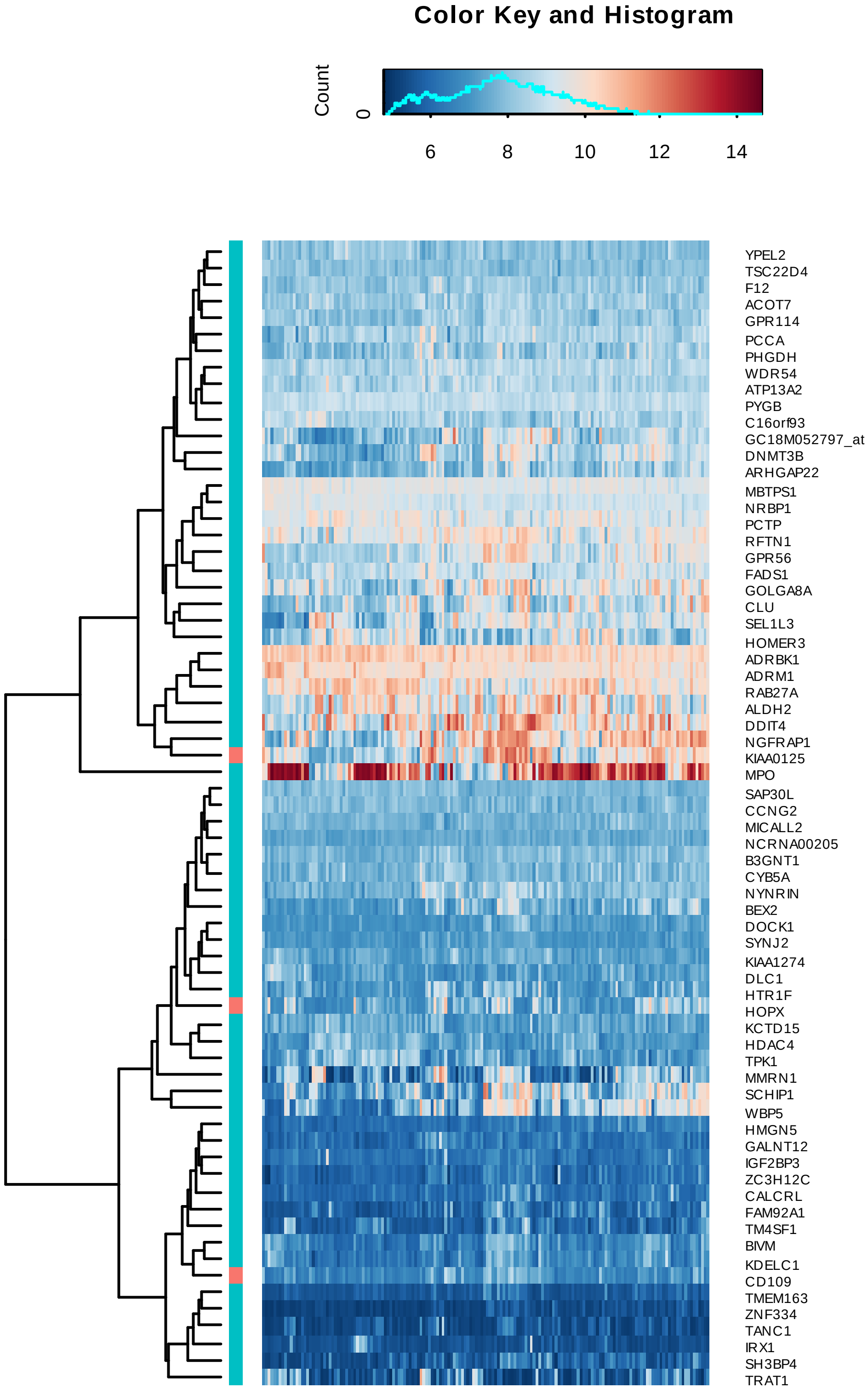

Supplementary Figure S2: Heatmap of the genes selected for comparison t(8;21)+ vs. t(8;21)- (data set HOVON) The heatmap shows the expression values of each of the genes selected for comparison t(8;21)+ vs. t(8;21)- for each sample in data set HOVON with t(8;21)-. The genes are in the rows and the observations are in the columns. The dendrogram on the left hand side shows the result of agglomerative hierarchical clustering using the Euclidian distance and complete linkage, where the dendrogram was reordered based on the mean expression values of the genes. The genes that were selected both from the comparison t(8;21)+ vs. t(8;21)- and from the comparison *RUNX1*+ vs. *RUNX1*- are marked in red on the left margin.

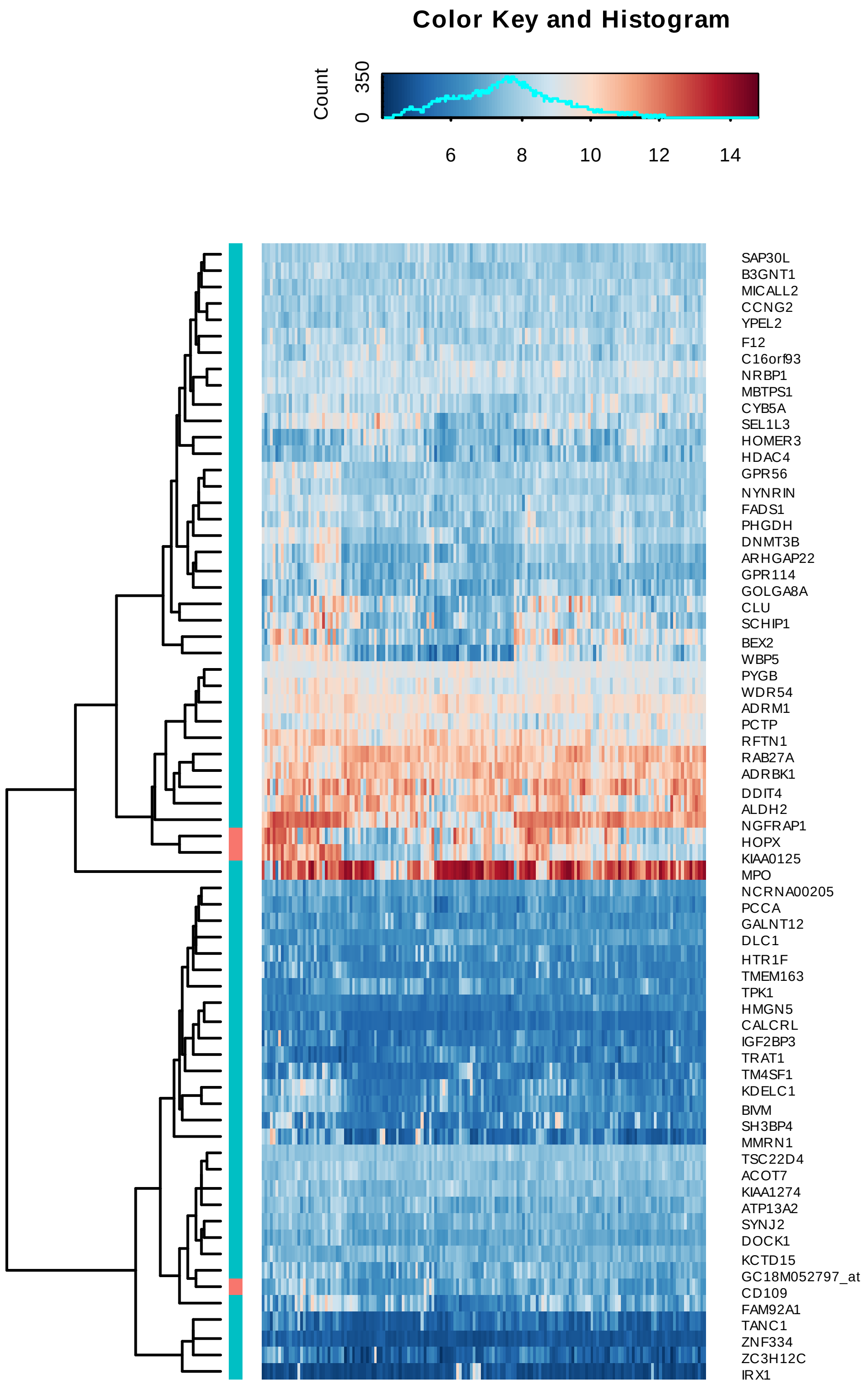

Supplementary Figure S3: Heatmap of the genes selected for comparison t(8;21)+ vs. t(8;21)- (data set AMLCG Cohort 1) The heatmap shows the expression values of each of the genes selected for comparison t(8;21)+ vs. t(8;21)- for each sample in data set AMLCG Cohort 1 with t(8;21)- and at the same time *RUNX1*-. The genes are in the rows and the observations are in the columns. The dendrogram on the left hand side shows the result of agglomerative hierarchical clustering using the Euclidian distance and complete linkage, where the dendrogram was reordered based on the mean expression values of the genes. The genes that were selected both from the comparison t(8;21)+ vs. t(8;21)- and from the comparison *RUNX1*+ vs. *RUNX1*- are marked in red on the left margin.

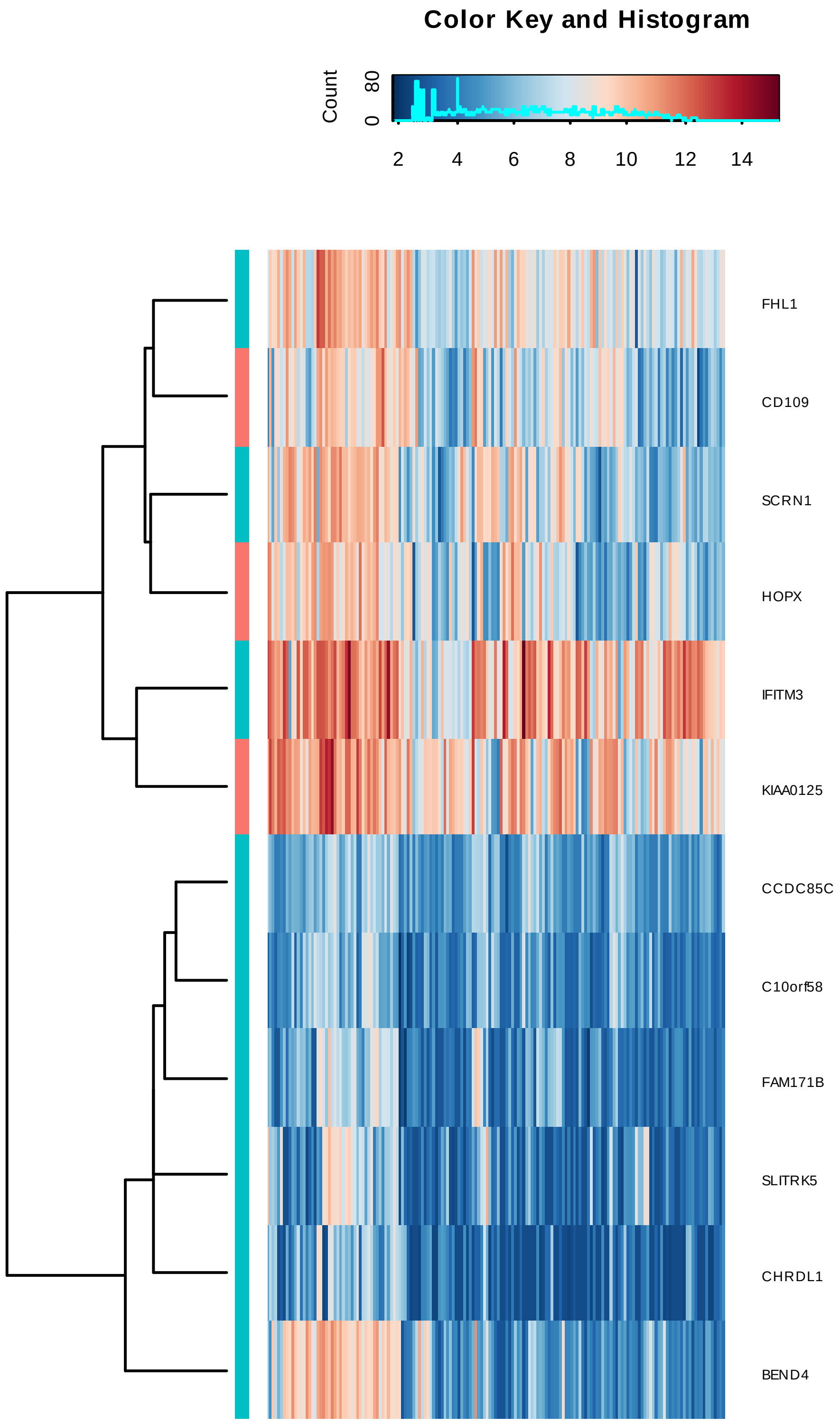

Supplementary Figure S4: Heatmap of the genes selected for comparison *RUNX1*+ vs. *RUNX1*- (data set AMLCG Cohort 2 with TCGA) The heatmap shows the expression values of each of the genes selected for comparison *RUNX1*+ vs. *RUNX1*- for each sample in data set AMLCG Cohort 2 with TCGA with t(8;21)- and at the same time *RUNX1*-. The genes are in the rows and the observations are in the columns. The dendrogram on the left hand side shows the result of agglomerative hierarchical clustering using the Euclidian distance and complete linkage, where the dendrogram was reordered based on the mean expression values of the genes. The genes that were selected both from the comparison t(8;21)+ vs. t(8;21)- and from the comparison *RUNX1*+ vs. *RUNX1*- are marked in red on the left margin.

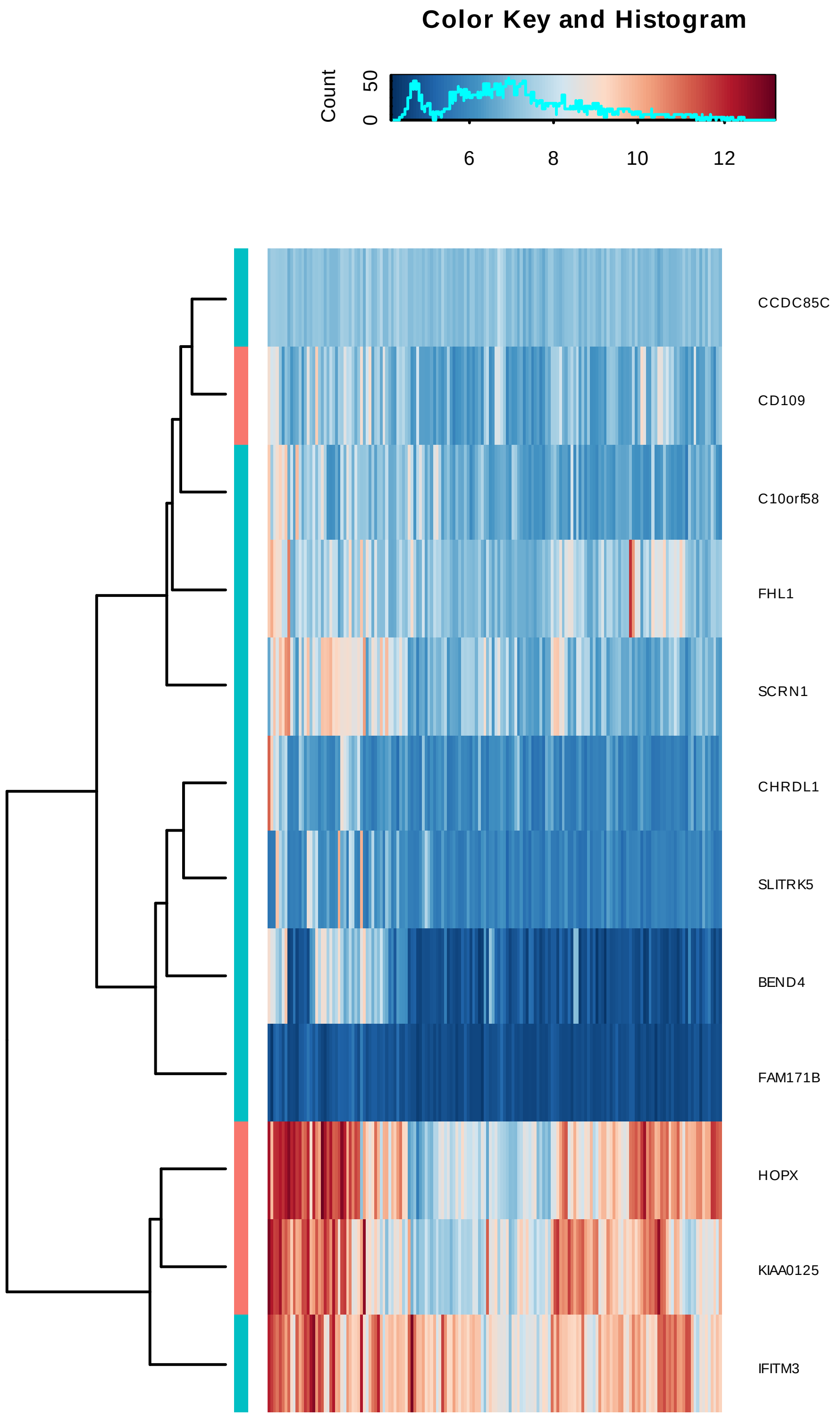

Supplementary Figure S5: Heatmap of the genes selected for comparison *RUNX1*+ vs. *RUNX1*- (data set AMLCG Cohort 1) The heatmap shows the expression values of each of the genes selected for comparison *RUNX1*+ vs. *RUNX1*- for each sample in data set AMLCG Cohort 1 with t(8;21)- and at the same time *RUNX1*-. The genes are in the rows and the observations are in the columns. The dendrogram on the left hand side shows the result of agglomerative hierarchical clustering using the Euclidian distance and complete linkage, where the dendrogram was reordered based on the mean expression values of the genes. The genes that were selected both from the comparison t(8;21)+ vs. t(8;21)- and from the comparison *RUNX1*+ vs. *RUNX1*- are marked in red on the left margin.

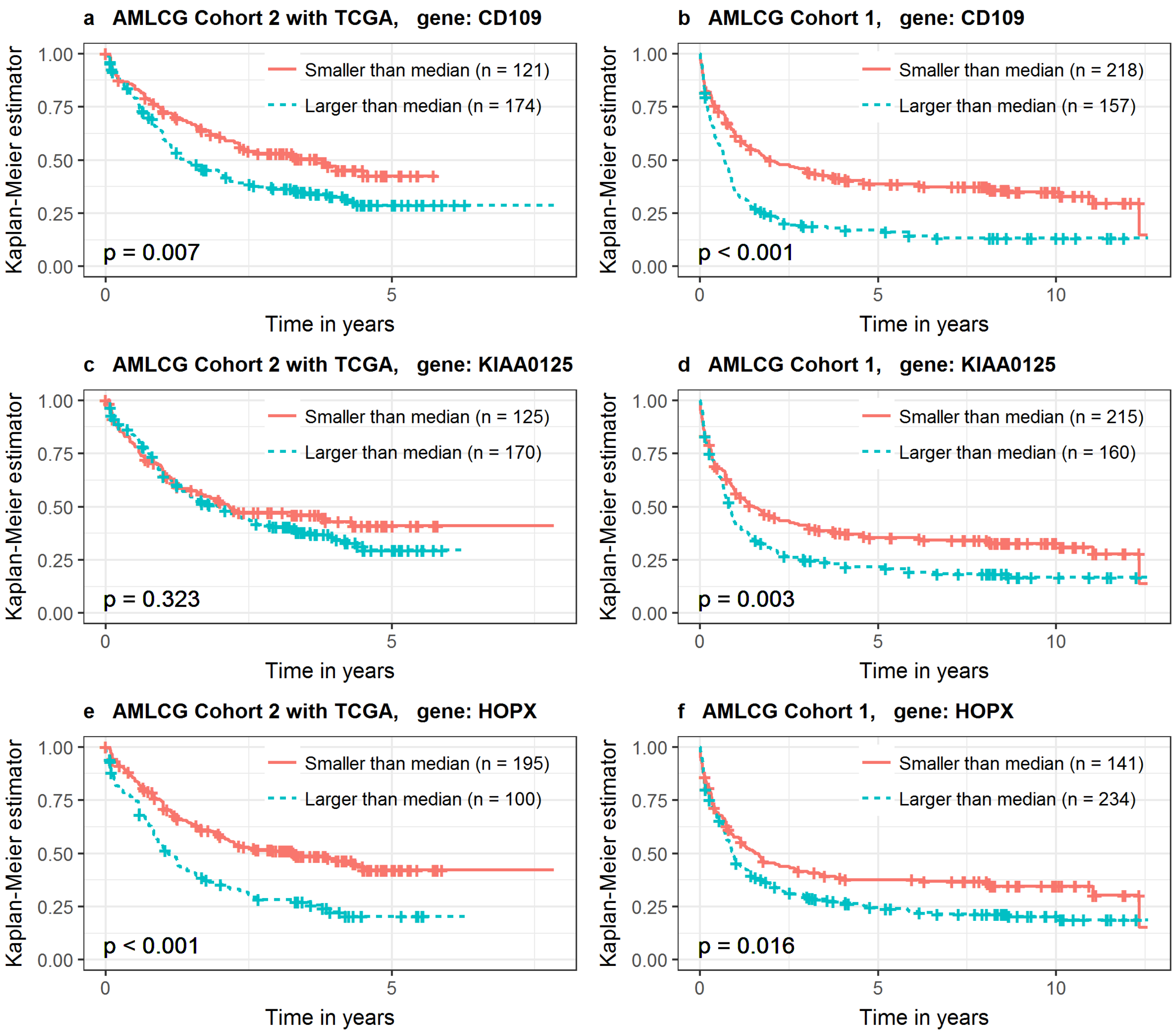

Supplementary Figure S6: *RUNX1*- with t(8;21)-: Survival differences between patients with high and low gene expression values Each subplot shows the Kaplan Meier curves of patients with expression values higher/lower than the median expression value for one of the three genes selected both from the comparison t(8;21)+ vs. t(8;21)- and from the comparison *RUNX1*+ vs. *RUNX1*-. In each case one of the data sets AMLCG Cohort 2 with TCGA and AMLCG Cohort 1 was used. For this plot only patients without *RUNX1* mutations and without a *RUNX1* fusion (t(8;21)-) were considered. The medians were in all cases taken across the data sets AMLCG Cohort 2 with TCGA and AMLCG Cohort 1. Censorings are indicated as plus signs in the subplots. The p values are the results of log-rank tests used to test for survival differences between the two groups associated with the respective Kaplan Meier curves.

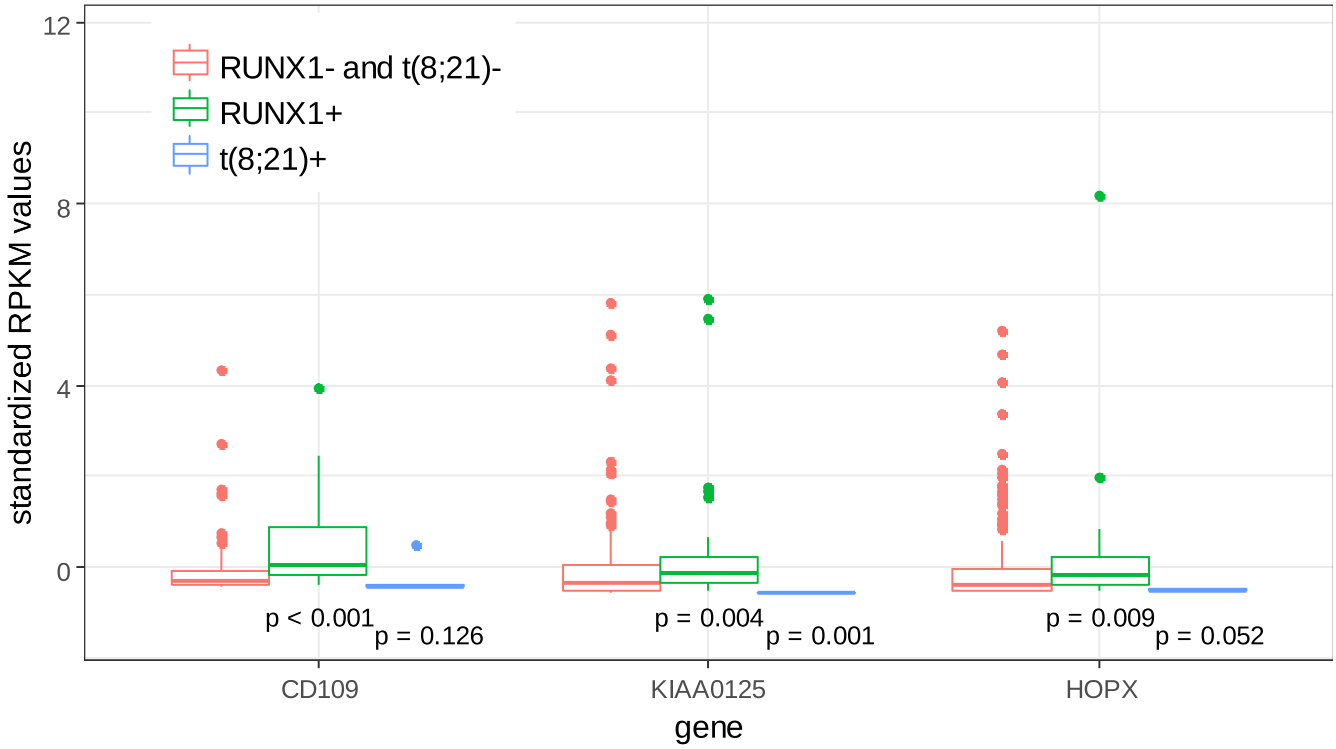

Supplementary Figure S7: Validation cohort: standardized RPKM values for the three genes selected as mediators both for the *RUNX1*+ vs. *RUNX1*- comparison and the t(8;21)+ vs. t(8;21)- comparison standardized RPKM values in validation cohort for the three genes that appear in both, Supplementary Table S1 and Supplementary Table S2, separated by whether only mutations (*RUNX1*+), only fusion (t(8;21)+) or neither of these two (*RUNX1*- and t(8;21)-) are present in the respective patients. The *p* values are the results of Wilcoxon tests. For each gene, we tested '*RUNX1*+' against '*RUNX1*- and t(8;21)-' and 't(8;21)+' against '*RUNX1*- and t(8;21)-'.

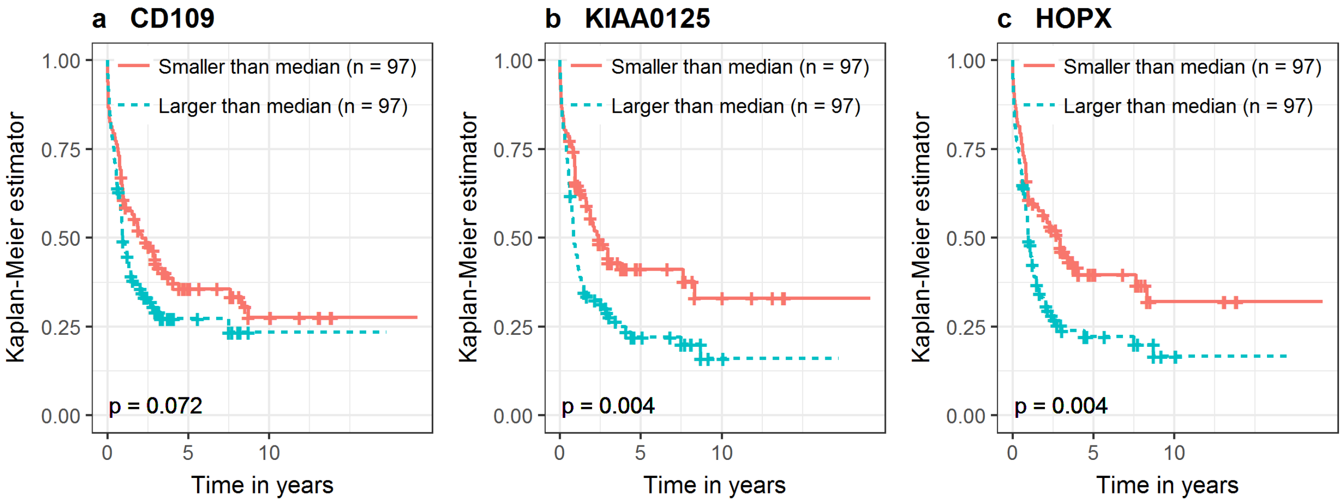

Supplementary Figure S8: Validation cohort: patients with *RUNX1*- and t(8;21)-: Survival differences between patients with high and low gene expression values Each subplot shows the Kaplan Meier curves of patients in the validation cohort with expression values higher/lower than the median expression value for one of the three genes selected both from the comparison t(8;21)+ vs. t(8;21)- and from the comparison *RUNX1*+ vs. *RUNX1*-. For this plot only patients without *RUNX1* mutations and without a *RUNX1* fusion (t(8;21)-) were considered. Censorings are indicated as plus signs in the subplots. The *p* values are the results of log-rank tests used to test for survival differences between the two groups associated with the respective Kaplan Meier curves.

References

1 Lange, T. & Hansen, J. V. Direct and indirect effects in a survival context. *Epidemiology* **22**, 575-581 (2011).

2 Johnson, W. E., Li, C. & Rabinovic, A. Adjusting batch effects in microarray expression data using empirical Bayes methods. *Biostatistics* **8**, 118-127 (2007).

3 Ng, S. W. *et al.* A 17-gene stemness score for rapid determination of risk in acute leukaemia. *Nature* **540**, 433-437, doi:10.1038/nature20598 (2016).

List of genes pre-selected for the comparison t(8;21)++ vs. *RUNX1*++ together with corresponding (unadjusted) *p* values obtained in the pre-selection

Variable	*p* value from pre-selection analysis
PPCDC	0.000588
PDIA3	0.000678
ABCA2	0.000954
PTGES	0.001043
MAPRE1	0.001254
GALNT7	0.001277
HTRA3	0.001481
LMO4	0.001893
NRIP3	0.001941
FAM125B	0.002256
HTT	0.002778
SLITRK5	0.002834
BTBD11	0.003208
ANKRD39	0.004207
GALR3	0.004363
LPHN1	0.004394
PDIA6	0.004416
CBX6	0.004488
SCN8A	0.004839
CATSPERG	0.004884
BEX2	0.005852
MAFG	0.005943
MGAT3	0.006255
CD59	0.006481
PGM2	0.006783
RAB4A	0.007271
MED12L	0.007618
SLC48A1	0.007703
ARHGAP5	0.007916
DLAT	0.007923
EPB49	0.008318
MLLT6	0.008384
KIAA0182	0.008736
RPAP2	0.008783
SLC25A37	0.008911
SLC24A6	0.008973
HOXA9	0.009422
CLCN2	0.009432

ST6GALNAC3	0.009711
HSP90B1	0.010094
TUFT1	0.010294
KLHDC8A	0.010402
CHST12	0.010407
CTNNBL1	0.010429
SETBP1	0.010894
C9orf142	0.010925
PANX2	0.01105
OSGIN1	0.011146
DPPA4	0.01115
NGFRAP1	0.011466
DPYSL3	0.011651
C21orf63	0.011683
NYNRIN	0.011686
NAPA	0.011809
TRIM2	0.012051
SLC25A13	0.012054
SLC9A5	0.012107
SLC45A4	0.01212
ANKH	0.012508
ARHGAP31	0.012684
MRS2	0.012724
ST6GALNAC4	0.012863
GRK6	0.012878
LTBP3	0.013008
SYNJ2	0.013061
IGF2R	0.013256
DVL3	0.013382
BEND4	0.013591
SLC6A8	0.013636
TTTY14	0.013771
NPDC1	0.01386
WNK1	0.01437
EIF3A	0.014429
SLC6A9	0.014945
ABCB6	0.015081
PAPSS1	0.015163
FAM169A	0.015398
PHF2	0.015472
PAFAH1B1	0.015606
TSPAN32	0.015721
SNAP23	0.015949
PVRL1	0.015979
HDAC5	0.016157
SWAP70	0.016194
MMRN1	0.016336

PDE8B	0.016714
PHKA1	0.016845
PAWR	0.016983
ANKMY2	0.017478
SEPT11	0.017498
SPRED2	0.01752
C1QTNF6	0.017549
DNAH10	0.017919
PHGDH	0.018195
GNPNAT1	0.018515
CDC123	0.018671
TSN	0.018675
CDKL5	0.019006
ADH5	0.019059
C1QBP	0.019068
OPTN	0.019204
BZW2	0.019736
PMM1	0.01999
FUT7	0.020124
YWHAE	0.020402
RASSF7	0.020695
NFKBID	0.020781
C7orf50	0.020819
FGFRL1	0.020947
ANK1	0.021499
PINK1	0.022045
TWSG1	0.022057
BMPR2	0.022116
C9orf103	0.022151
GPN3	0.022332
TMCC2	0.022469
IPO11	0.022491
GFI1B	0.022497
CPNE8	0.023057
GPATCH8	0.023262
TIA1	0.023358
CCT2	0.023428
ITPR3	0.023692
ULK1	0.023926
NOTCH1	0.023972
MAGED1	0.024448
ZNF135	0.024541
PTPN1	0.024653
HAT1	0.024854
SHROOM4	0.024913
FAM160A1	0.025083
CCND3	0.025144

RAP1GAP	0.025221
MTM1	0.025255
PITPNB	0.02561
OSBP2	0.025643
HPCAL1	0.025672
SLC2A1	0.025681
GRM2	0.025747
SLC25A39	0.026246
TM6SF1	0.02634
EFNA1	0.026531
ACVR2A	0.026789
ANKRD2	0.026948
PHAX	0.027133
GPR35	0.027157
CTXN1	0.027213
SLC25A24	0.027333
SLC4A1	0.027568
CTSC	0.027676
ABCG1	0.027828
CHST11	0.027874
MAP6D1	0.02788
MTCH1	0.027897
STAP1	0.028266
HOPX	0.028348
FKBP1B	0.028377
ADARB1	0.02859
CLIC2	0.028703
PIM2	0.028802
OBSL1	0.028946
SMOX	0.029006
KCNA3	0.029524
PRDX4	0.029541
F12	0.02955
KLC2	0.02961
ASB2	0.029663
PUS7	0.029753
CHD3	0.029772
TSPAN17	0.029954
LARP6	0.030141
PSTPIP1	0.03022
THAP10	0.030357
CYYR1	0.030539
ATG4D	0.030858
ARL1	0.030926
PRKD2	0.031301
RNF208	0.03143
PDCD4	0.03155

NT5C3L	0.031632
BRF1	0.031847
TMEM63B	0.031868
CUEDC1	0.03211
COMMD8	0.032142
GRHL1	0.03221
TANC1	0.032433
DNAJB11	0.032514
SPNS3	0.032694
FBXO7	0.033263
NFE2	0.033539
C5orf4	0.033607
RG9MTD2	0.033671
JOSD2	0.033732
ZNF664	0.033897
UGT8	0.034034
PRDX3	0.034198
INF2	0.034239
TMOD1	0.034318
TRAF4	0.034561
ALAS2	0.034754
LOC388588	0.034865
LYPLA1	0.035065
SLC7A5	0.035307
NBEAL2	0.03534
TP53I11	0.035473
TNS1	0.035719
POLR1B	0.035858
NOL8	0.035981
DAB1	0.036349
SETMAR	0.036533
TFE3	0.036618
KBTBD3	0.03666
AGPS	0.036846
IDS	0.037093
CYB5R4	0.037245
SPIB	0.03731
ZFAND2B	0.037348
ELF1	0.037412
SELO	0.037617
PLEKHM1	0.037646
INPP5K	0.037736
KLF12	0.03795
SERPINB1	0.037965
SPSB1	0.038111
SDK2	0.038162
C5orf56	0.038344

ABHD10	0.039459
YARS2	0.039568
RCN1	0.03958
ZNF295	0.039657
KLHL3	0.039682
ANKS6	0.039952
RBM38	0.040262
MFAP3L	0.040313
FAM32A	0.040398
FTL	0.04047
SLC41A1	0.04082
VDAC3	0.040864
SPTB	0.041069
CSPP1	0.041191
CD7	0.041467
C10orf10	0.041555
TFB2M	0.041619
HOXA7	0.041683
SPON2	0.041688
RFTN1	0.041839
TMEM86A	0.041862
ERLIN2	0.041967
ADAM17	0.041991
ZNF85	0.042183
RTN1	0.042199
CD109	0.042824
APOLD1	0.042844
ICAM2	0.0432
KRTAP5-9	0.04328
CAV1	0.043486
SFXN4	0.043807
SIPA1	0.04386
ARID5A	0.04403
SPATC1	0.044306
SCML1	0.044407
MRPL3	0.044427
GALNT1	0.044544
SLC29A2	0.044946
PFKM	0.044963
ITGA4	0.045045
DMKN	0.045152
PRKCZ	0.045162
SNX14	0.04522
SLC25A28	0.045237
C10orf88	0.046025
CRBN	0.046101
SIAH2	0.046297

ERMAP	0.046533
THBD	0.046592
ZNF532	0.046641
SCARB2	0.046846
EXPH5	0.046981
ERCC1	0.047007
DEPDC7	0.04708
NCOR2	0.047087
RHAG	0.047222
USF2	0.047384
ANKMY1	0.047512
NMI	0.047668
ID2	0.047983
AUTS2	0.047986
UGP2	0.048065
GLA	0.048107
EPS8L2	0.048192
SIAE	0.048305
HAGH	0.048417
RABEP1	0.048632
DEDD2	0.048647
EMILIN1	0.048813
YWHAH	0.049201
CRISP3	0.049338
SEPT9	0.049362
GPR108	0.049365
PDIA4	0.049739
PNPT1	0.049918

List of genes found to be differentially expressed using limma for the comparison t(8;21)++ vs. *RUNX1*++ together with p values after Benjamini-Hochberg adjustment obtained in the validation analysis

Variable	*p* value from validation analysis (using AMLCG Cohort 1)
RUNX1T1	1.26E-41
POU4F1	1.94E-35
CAV1	6.84E-17
PALM	8.29E-15
IL5RA	1.12E-14
HYAL3	1.95E-14
HOXA9	1.07E-13
TRH	1.16E-13
LAMA5	1.94E-13
VOPP1	4.78E-13
KIAA0125	5.41E-13
PRAME	2.98E-12
KDM4B	1.42E-11
LAT2	1.71E-11
SIPA1L2	2.89E-11
GDF11	3.23E-11
SPINK2	3.31E-11
HOPX	7.07E-11
CACNA2D2	7.34E-11
NTNG2	7.34E-11
CD19	1.73E-10
PRKD3	1.79E-10
LOXL3	1.9E-10
NGFRAP1	2.33E-10
WFDC1	3.17E-10
CDH26	4.37E-10
ADRM1	5.94E-10
SLC28A3	8.77E-10
SLC25A1	1E-09
SSX2IP	1.21E-09
C15orf39	1.28E-09
ROBO1	1.93E-09
SLC24A3	2.88E-09
WBP5	3.78E-09
CD38	4.79E-09
TRAF7	4.93E-09

TNS3	5.07E-09
SLC5A10	5.08E-09
ARHGAP21	5.37E-09
ZG16B	1.12E-08
MEIS1	1.29E-08
SCCPDH	1.32E-08
OPTN	1.32E-08
STXBP5	1.38E-08
GLT25D1	1.38E-08
KCNE1L	1.5E-08
NAT6	1.88E-08
TSPAN32	1.88E-08
NIPA1	1.88E-08
PPBP	1.89E-08
CMTM4	2.47E-08
CBX6	2.47E-08
PXK	2.51E-08
MSI2	2.52E-08
VEGFA	3.14E-08
HPGDS	3.46E-08
STAR	3.46E-08
HSPG2	3.59E-08
TMEM44	3.83E-08
CLIC2	3.83E-08
PLTP	4.21E-08
PURA	5.54E-08
TPK1	5.54E-08
USP6NL	5.54E-08
CLEC11A	6.08E-08
CLEC5A	7.03E-08
FNIP2	8.29E-08
NIT2	8.43E-08
FNDC3B	9.7E-08
SLC48A1	9.84E-08
KCTD6	1.11E-07
IGLL1	1.11E-07
HAL	1.32E-07
CALR	1.4E-07
SHC1	1.44E-07
BMPR2	1.44E-07
LOC374443	1.51E-07
LAMC3	1.61E-07
DSE	1.77E-07
ADCY7	1.98E-07
GPM6B	1.98E-07
MAGED2	1.98E-07
DAGLB	2.29E-07

CORO2A	2.96E-07
HOXB4	3.12E-07
PKIG	3.27E-07
RWDD2A	3.33E-07
DEPDC7	3.38E-07
RETN	3.48E-07
HYAL2	3.82E-07
CD109	4.19E-07
ANGPTL6	4.2E-07
FBXO21	4.21E-07
SRXN1	4.21E-07
HOXA3	4.43E-07
CD96	4.78E-07
PHYHD1	4.83E-07
MPO	4.97E-07
HOMER3	4.97E-07
TIE1	5.06E-07
VLDLR	5.96E-07
EPCAM	6.79E-07
SERPINB1	7.26E-07
ABCA1	7.26E-07
VAT1	7.38E-07
ZC3H12C	8.36E-07
ZNF664	8.36E-07
EEF2K	8.4E-07
TP53I3	8.41E-07
ITGA9	9.45E-07
DPY19L3	9.45E-07
FBLN5	1.02E-06
TPSAB1	1.08E-06
IRX1	1.09E-06
BTBD11	1.14E-06
PON2	1.18E-06
CPVL	1.21E-06
C16orf93	1.26E-06
CD36	1.34E-06
LPCAT4	1.36E-06
ALCAM	1.37E-06
CD59	1.38E-06
HDAC4	1.53E-06
PIAS3	1.54E-06
SCHIP1	1.74E-06
ALDH2	1.81E-06
HOXA7	1.82E-06
THBS3	1.95E-06
CHIC1	1.95E-06
CMTM7	2.01E-06

SCMH1	2.04E-06
MLLT3	2.12E-06
NXF3	2.14E-06
CAMKK2	2.14E-06
SLC41A1	2.15E-06
GPR56	2.21E-06
RUNX3	2.21E-06
CTNND1	2.24E-06
SLC46A3	2.35E-06
BEX4	2.48E-06
P2RX5	2.48E-06
DNM1	2.8E-06
DHCR7	2.83E-06
SRPRB	2.87E-06
SLC45A3	2.88E-06
PTPN7	2.9E-06
SGMS1	2.9E-06
ACCS	3.08E-06
TMEM43	3.22E-06
C11orf21	3.22E-06
ACVR1	3.31E-06
GALNT2	3.56E-06
WIPF3	3.68E-06
MAFK	3.69E-06
PHF19	4.36E-06
PCGF5	4.38E-06
IL17RB	4.43E-06
SYNGR1	4.61E-06
NTRK1	4.8E-06
ZC3H3	5.03E-06
NARFL	5.25E-06
CLIP4	5.31E-06
LRWD1	5.33E-06
ACP5	5.33E-06
GPR153	5.51E-06
CTNNBL1	5.52E-06
BEND4	5.6E-06
LAIR1	5.6E-06
ARHGAP5	5.6E-06
LFNG	5.96E-06
GCLM	6.26E-06
PLXNC1	6.51E-06
TM6SF1	6.51E-06
CRIM1	6.65E-06
PF4	6.65E-06
SLC12A7	6.65E-06
CPNE8	6.65E-06

ZNF238	7.01E-06
SDPR	7.23E-06
HOXB3	7.45E-06
BDH1	7.6E-06
EDEM1	7.88E-06
ALOX12	7.9E-06
GRAMD1A	7.9E-06
GSDMD	8.42E-06
BLVRB	8.46E-06
TMC4	8.46E-06
CTSC	8.63E-06
CAPRIN2	8.63E-06
TMEM216	9.35E-06
ATP1B1	9.55E-06
SMAD3	9.73E-06
DOK1	1.07E-05
GSN	1.08E-05
HOXB2	1.14E-05
PLBD2	1.18E-05
SLC25A29	1.18E-05
GNB5	1.18E-05
EGLN1	1.22E-05
ACP6	1.3E-05
EDEM2	1.3E-05
TM7SF3	1.3E-05
F2RL1	1.34E-05
POLE	1.34E-05
TMEM38B	1.4E-05
TUSC1	1.42E-05
ESYT1	1.42E-05
ASL	1.43E-05
TSPAN17	1.49E-05
CSF3R	1.57E-05
CXXC5	1.63E-05
ABCG1	1.78E-05
CAV2	1.84E-05
C3AR1	1.84E-05
TM4SF1	1.87E-05
RASSF1	2.12E-05
C1QTNF4	2.19E-05
NAT14	2.44E-05
DNMT3A	2.48E-05
TMEM161A	2.51E-05
JAZF1	2.57E-05
GPR114	2.57E-05
SMOX	2.61E-05
C6orf192	2.71E-05

TTC7B	2.72E-05
PGAP2	2.95E-05
IFFO2	3E-05
SLC18A2	3.04E-05
CRIP2	3.1E-05
ACOT13	3.14E-05
WNK1	3.34E-05
PRKCH	3.57E-05
CLINT1	3.58E-05
LPAR5	3.58E-05
HEBP1	3.58E-05
GFI1B	3.71E-05
TRIO	3.84E-05
CRELD1	3.86E-05
TMOD1	3.91E-05
DAD1	3.93E-05
ANKRD37	4.12E-05
WWP1	4.23E-05
RALGAPA2	4.24E-05
RBM38	4.62E-05
GPR146	4.66E-05
ARHGAP12	4.78E-05
FKBP1B	4.79E-05
LPCAT2	4.79E-05
EVC2	4.86E-05
CHST12	4.99E-05
TANC1	5E-05
SYNJ2	5E-05
SOX15	5.02E-05
ANKRD13B	5.34E-05
FAM92A1	5.49E-05
SECTM1	5.49E-05
XK	5.57E-05
C10orf125	5.57E-05
GANAB	5.57E-05
SLC6A8	5.66E-05
AEBP1	5.8E-05
TUBA1A	5.8E-05
EFNA1	5.8E-05
INTU	6.03E-05
PRKAR2B	6.12E-05
GALNT1	6.2E-05
ITPKA	6.35E-05
TPPP3	6.47E-05
CD300A	6.51E-05
MXRA7	6.58E-05
EPS15L1	6.58E-05

PI4K2A	6.8E-05
SBF2	6.96E-05
HOXB5	6.97E-05
FBXO7	7.51E-05
CA1	7.62E-05
ANXA6	7.69E-05
BEX2	7.93E-05
ARHGEF6	8.14E-05
CSRP2	8.17E-05
GUSB	8.17E-05
CPXM1	8.31E-05
EPB49	8.35E-05
DBN1	8.44E-05
NOC2L	8.66E-05
HK2	8.77E-05
ATP6V1A	8.95E-05
COMT	9.21E-05
IGF2BP3	9.37E-05
MAP4K2	9.43E-05
AP1B1	9.43E-05
ZCCHC2	9.44E-05
PODXL2	9.83E-05
RXRA	9.83E-05
PTPN4	9.9E-05
C21orf56	9.92E-05
SLC40A1	0.000101
SUCNR1	0.000101
BTK	0.000101
PTX3	0.000102
ZCCHC24	0.000103
HTR1F	0.000104
LTBP1	0.000104
TPM1	0.000107
CD244	0.000108
ABR	0.000108
MSLN	0.000109
C10orf10	0.000114
HOMER2	0.000114
SPNS3	0.000114
MBP	0.000114
GALNT12	0.000117
NPHP4	0.000117
CDK5RAP2	0.000118
FSCN1	0.000118
DOCK11	0.000118
PRMT1	0.000121
STAP1	0.000123

ELANE	0.000127
CYYR1	0.000127
PLAU	0.000131
SNCA	0.000132
ACVR2A	0.000134
IDS	0.000134
TSTD1	0.000135
HOXA5	0.000136
DHRS3	0.000137
NPW	0.000137
LRP3	0.000144
CTSW	0.000147
PRTFDC1	0.000147
KLHDC8A	0.000154
FAM46A	0.000154
GALC	0.000154
MANF	0.000156
RFXANK	0.000157
EPB42	0.000159
SMARCA4	0.000167
DVL3	0.000167
C16orf5	0.000167
PGM2	0.000168
QPRT	0.00017
PCTP	0.000172
KIAA0182	0.000172
TUBB1	0.000172
KBTBD7	0.000181
JUP	0.000185
ISYNA1	0.000188
HTT	0.000192
ABCA5	0.000193
RNF187	0.000194
TCEAL3	0.000194
TKTL1	0.000194
PDLIM1	0.000194
ATXN1	0.000197
KIAA1274	0.0002
KCNQ5	0.000204
CALCRL	0.000204
CDCA7L	0.000208
RPUSD3	0.000213
SH2D3C	0.000218
TRIM58	0.000218
AVEN	0.000218
UTY	0.000227
FYN	0.000229

DHX33	0.000229
PLD1	0.000231
CD58	0.000232
CLEC2L	0.000237
PSMF1	0.000238
TMIGD2	0.000242
DCBLD1	0.000242
CMTM5	0.00025
DEF8	0.000255
DOCK1	0.000257
ARHGEF3	0.00026
PGRMC1	0.00026
CCDC125	0.000262
CYTL1	0.000262
CEBPE	0.000262
RIN2	0.000262
HSP90B1	0.000264
VSTM1	0.000267
SLC2A1	0.00027
ANK1	0.00027
NOTCH1	0.000275
EMR1	0.000278
ZNF652	0.000278
SORL1	0.000281
USP10	0.000286
CD72	0.00029
DTX3	0.000298
FNDC3A	0.000305
SLC35B2	0.000308
ITGAE	0.000312
CCDC86	0.000313
CD84	0.000315
SLC44A1	0.000319
NME4	0.000333
SFXN3	0.000334
RHBDF1	0.000338
BAIAP3	0.000338
IL1RL1	0.000343
NUDT13	0.000345
PLXNB2	0.000349
ITGB4	0.000362
LDHA	0.000376
AGPS	0.000383
FAM125B	0.000383
LSP1	0.000392
LRP11	0.000396
PAPSS1	0.000399

PCNX	0.000403
L3MBTL4	0.000409
NEDD4L	0.000416
FNBP1	0.000424
ZEB1	0.000424
NRBP1	0.000426
XYLT1	0.000426
CCDC102A	0.000428
SELENBP1	0.000436
ST6GALNAC4	0.000436
SPTLC2	0.000436
GPATCH8	0.00044
TSPAN4	0.000447
UBA1	0.000456
ZNF711	0.000456
CYP4F2	0.000456
IQCE	0.000462
QSOX1	0.000465
GABARAPL1	0.000479
TARBP2	0.000479
NFE2	0.000479
GPI	0.00048
KDM5D	0.000487
AKAP2	0.000488
GBE1	0.000498
PGM3	0.000502
RAB40C	0.000517
MAPKAPK3	0.000524
KBTBD8	0.000524
FBXW9	0.000525
ITGA2B	0.000531
PITPNM2	0.000538
SDCCAG8	0.000542
CAMK2D	0.000542
DLL1	0.000542
PGD	0.000546
DUSP7	0.000554
CHST2	0.000554
EXT2	0.000554
ADRA2C	0.000561
NR2F6	0.000568
PRSS21	0.000568
ANKH	0.000568
PDLIM2	0.000569
GCNT2	0.000574
ACADSB	0.000577
RHAG	0.000585

GATM	0.000588
HPGD	0.000588
C11orf63	0.000588
HOXB6	0.000588
PYGB	0.000591
ARC	0.000597
C19orf76	0.000599
DACT1	0.000606
ANPEP	0.000621
FSTL3	0.000625
PTPN12	0.00063
AHSP	0.000634
HEXA	0.000645
C2orf18	0.000649
WIPF1	0.000651
SELS	0.000652
SH2B3	0.000655
INO80C	0.000657
SPRED1	0.000671
PPP1R16B	0.000673
ATP2B4	0.000674
METRNL	0.000675
IL15	0.000679
NOTCH2	0.000684
TNS1	0.000684
OSGEP	0.000684
MRS2	0.000691
KCTD15	0.000695
FAM169A	0.000698
SIAE	0.000698
SMYD2	0.000708
ALAS2	0.000713
FRMD4B	0.000722
STIM2	0.000724
BSPRY	0.000727
SORD	0.000731
C1orf174	0.000736
ZNF804A	0.000736
CCDC92	0.000736
ARSD	0.000758
TRIM8	0.000775
YWHAH	0.000784
DISC1	0.000785
MYLK	0.00079
MTMR1	0.000799
KCTD5	0.000799
NUAK2	0.000816

UBE2QL1	0.000818
CHD4	0.000829
FUT4	0.000855
E2F7	0.000855
CPA3	0.000857
METTL9	0.000866
NFE2L3	0.00087
SH3BP4	0.000881
ATP13A2	0.000915
DOPEY1	0.000915
PCCA	0.000924
FNBP1L	0.000927
CPEB4	0.000927
RASA3	0.000927
PALLD	0.000929
KEL	0.000929
SLC4A1	0.000929
SPINK4	0.000943
GCNT1	0.00095
HTATIP2	0.00095
TSLP	0.000973
ECHS1	0.000976
TK2	0.000981
WASL	0.000982
DDIT4L	0.000994
GNA11	0.000994
FAM60A	0.000994
C20orf194	0.000996
RAP1GAP2	0.001008
KIAA1549	0.001017
PLXDC2	0.001017
C12orf29	0.001017
AHCY	0.001027
ALAS1	0.001032
MED12	0.001053
KLF11	0.001084
PTPN1	0.001096
GTF3C5	0.001128
IGSF1	0.001128
VASN	0.001128
CEACAM4	0.001128
PAWR	0.001131
NCAM1	0.001151
TBC1D1	0.001167
SEL1L3	0.001169
FHDC1	0.001173
FGD6	0.001173

DSG2	0.001173
ALS2	0.001195
TRIM6	0.001199
MYCT1	0.001214
UBP1	0.001217
MEST	0.001224
SH3TC1	0.001236
LYPLA1	0.001236
SIDT2	0.001236
SLCO4A1	0.001236
C9orf25	0.001237
DDIT4	0.001237
UBE2F	0.001247
OSBPL3	0.00127
ASGR2	0.00127
TNFRSF1B	0.001278
MTL5	0.001311
PIGB	0.001326
HEMK1	0.001332
LPIN1	0.001341
GBGT1	0.001342
PRKCZ	0.001342
FAM159A	0.001342
MYL4	0.001351
MAFB	0.001365
MAN1A1	0.001386
MYH10	0.001411
FBXO3	0.001418
SNAP23	0.001434
MAPRE1	0.001482
HMBS	0.001502
HSF5	0.001502
ADD2	0.001503
KCNK17	0.001503
NOD2	0.001503
CDCA7	0.001533
DUSP28	0.001533
HAGH	0.001549
BCL3	0.001568
PANX2	0.001575
SLC22A18	0.001613
MLEC	0.001613
EMR2	0.001627
ABCB6	0.001631
CNN3	0.001634
ERMAP	0.00166
BCL2L11	0.001687

IKZF2	0.001691
MEX3A	0.001703
TXNIP	0.001723
RTCD1	0.001727
RMND5B	0.001727
SIGLECP3	0.00178
BCR	0.001785
TMEM158	0.001805
ZYG11B	0.001809
RAB5B	0.001849
FAM81A	0.001896
ZBTB38	0.001897
C1orf186	0.001898
CASP2	0.001949
FAM129B	0.001954
PLIN2	0.001955
PDIA6	0.001965
SFXN4	0.001965
LYSMD4	0.001974
MICAL2	0.001984
CCT2	0.002032
TRAK2	0.002059
DLC1	0.002077
ARRB1	0.002077
PEA15	0.002111
CCDC109B	0.002122
H2AFJ	0.002139
LCMT1	0.002139
ID2	0.002141
MAP3K14	0.002141
SYTL4	0.002146
EGFL7	0.002148
TXLNA	0.002163
RCN1	0.002163
SPTB	0.002192
CLU	0.002197
IFNAR2	0.002197
CECR6	0.002197
ZCCHC6	0.002198
FRMD4A	0.002202
C5orf25	0.002226
ADAMTSL4	0.002267
SMARCD2	0.002272
NCEH1	0.002272
ARL4C	0.002337
LAG3	0.002354
AKT3	0.002378

GLIS3	0.002378
MST4	0.002378
ZNF518A	0.002411
TGFBI	0.002411
STXBP1	0.00242
PIM2	0.00242
SAP30L	0.002434
GDPD5	0.002441
DOPEY2	0.002441
ORAI2	0.002446
ZFY	0.00246
KPTN	0.00248
DPPA4	0.002492
LMO4	0.002504
UGP2	0.00253
LAPTM5	0.002544
ARFGAP2	0.002557
RCBTB1	0.002581
LTBP3	0.002588
ANGPT1	0.002626
C14orf101	0.002677
LPPR3	0.002677
MAN1B1	0.002699
EFHC2	0.002712
DNAJB11	0.002727
INPP5J	0.002745
IRF8	0.00277
C5orf4	0.002776
COPG2	0.002786
F11R	0.002807
ACSM3	0.002812
CDAN1	0.002822
SKP2	0.00283
E2F2	0.002858
SNX9	0.002866
ZBTB42	0.002882
SLC23A2	0.002887
SETBP1	0.002971
SLC22A16	0.002992
ALS2CL	0.003024
CPNE7	0.003024
TBXA2R	0.003024
RFTN1	0.003028
PMP22	0.003028
MMRN1	0.003037
MEIS2	0.003063
LPHN1	0.003081

OLIG1	0.003084
PPAP2A	0.003094
PRR5L	0.003108
JAM3	0.003117
TBL1X	0.003119
MED21	0.003172
PTPRM	0.0032
CLCN3	0.003229
PDIA4	0.003295
TREX1	0.003298
KIAA1211	0.003313
TNFRSF4	0.003343
TMEM163	0.003364
SGSH	0.003364
CDKN2C	0.003439
MTMR9	0.003465
DFFB	0.003491
C9orf72	0.003491
TUBB6	0.003542
CYP1B1	0.003557
FAM82A1	0.003557
MTHFD1L	0.003557
C9orf9	0.003557
ELK3	0.003561
KAT2B	0.003625
LOC642852	0.003688
SORBS3	0.003706
FBXL19	0.003712
TMEM150A	0.003742
BZW2	0.003763
DDOST	0.003833
SMARCC1	0.00384
CALU	0.003842
ATG4D	0.003842
PROS1	0.003866
THOC5	0.003866
CEP135	0.003868
FAM54B	0.00387
CYTH4	0.00387
C6orf162	0.003883
HERPUD2	0.003901
PORCN	0.003901
S100A16	0.00392
RERE	0.003947
FXYD6	0.003951
DPYSL2	0.00396
ULK1	0.003963

SLC23A1	0.003974
ANXA5	0.003977
CCM2	0.003996
ARNTL	0.004034
CHST10	0.004038
SLC16A7	0.004058
DOK3	0.004138
NEFH	0.004164
FAM53C	0.004181
SLC25A37	0.004203
B3GNT1	0.004255
TMCC2	0.004307
SH3BP5	0.004313
KIAA1598	0.00439
NT5DC3	0.004426
MED12L	0.004435
TCF3	0.004435
PLIN3	0.004435
ZNF135	0.004479
MCCC1	0.004494
PBX3	0.004494
MAGED1	0.004494
MLLT1	0.004548
IGSF10	0.004602
PES1	0.004632
NPDC1	0.004647
CHN2	0.00468
STK32B	0.004699
HYOU1	0.00474
PPCDC	0.00474
C1QBP	0.00474
ZNF662	0.004758
RUVBL2	0.004758
IQSEC1	0.004796
MRPL44	0.004805
MMS19	0.004844
CERCAM	0.004853
DDX3Y	0.004853
ETS2	0.004853
CCDC6	0.004901
FAF1	0.004917
CSNK1E	0.004917
C9orf40	0.004928
PPIP5K2	0.004936
LRRC34	0.004959
SYNE2	0.004997
PTGR1	0.005012

LARP6	0.00504
DDAH1	0.005069
SPP1	0.005094
BCL7A	0.005094
NINJ1	0.005097
ELF4	0.005097
ADAMTS3	0.005114
LAMB2	0.005148
PADI2	0.005154
TTLL4	0.005154
ACOX2	0.005156
ATP6V0A1	0.005166
FARSA	0.00518
SLC39A8	0.005364
KLHDC3	0.005364
RAP1GAP	0.005377
TOB2	0.005377
DNAJB6	0.0054
GPR183	0.005432
TNFAIP2	0.005457
TUBG2	0.005483
RWDD1	0.005519
DLL3	0.005608
KIAA0226	0.005611
C7orf58	0.005635
TES	0.005704
IL6R	0.005712
MAP7	0.005728
ZNF184	0.005798
CYB5R4	0.005822
SELL	0.005829
AGPAT9	0.005829
PHKA1	0.005909
CCND3	0.005973
NME1	0.005976
ANKLE2	0.006007
MRPL1	0.006052
ZMYM4	0.006072
RUNDC3A	0.006072
LGALS3	0.006157
TMEM2	0.006195
LGALS2	0.006353
ALDH1A1	0.006396
ICA1	0.006413
DLGAP3	0.006426
EIF3A	0.006456
ARHGEF7	0.006499

OBSL1	0.006574
PRDM1	0.006685
GDF15	0.006693
SLC7A5	0.006716
PDIA3	0.00672
F3	0.006747
HNRNPF	0.006835
UXS1	0.006852
NCALD	0.006863
STK24	0.006924
PINK1	0.006935
SLC6A19	0.007068
ALDH18A1	0.007068
TMEM50B	0.007068
E2F8	0.007178
TSPAN31	0.00719
RASD1	0.007216
SLC25A39	0.007216
TMEM8B	0.007243
TDRKH	0.007253
BAG3	0.007446
GNA12	0.007456
TET1	0.00754
APEX1	0.00754
SDSL	0.007719
FBXO15	0.007833
EIF4E3	0.00787
ARMCX2	0.007903
CBR3	0.007904
PDRG1	0.007947
ZNF318	0.007985
HCFC2	0.00799
PRDM16	0.008001
ITFG3	0.008001
SMAD7	0.008007
UNG	0.008009
ZNF574	0.008009
OSBP2	0.008009
CYR61	0.008123
CABLES2	0.008153
JHDM1D	0.008155
USP9Y	0.008177
MATN2	0.008245
SLITRK5	0.008276
AP1S2	0.00828
GNGT2	0.008306
EMILIN1	0.008309

CREB3L2	0.008309
HEPACAM2	0.008313
SLC43A3	0.008337
KIAA0247	0.008368
HMGA2	0.008479
CDK2AP1	0.008484
CCNJ	0.008537
MRPL3	0.008545
PLAGL1	0.008545
NACC2	0.008545
ABI3	0.008545
TNFRSF10B	0.00855
BCAR3	0.008605
GAS2L1	0.008605
KIAA0090	0.008624
PPARD	0.008632
ME1	0.008675
SLC9A6	0.008701
PELI1	0.008969
SNX24	0.008996
EVPL	0.009021
PARP3	0.009046
PITPNA	0.009046
C19orf51	0.009046
F12	0.009051
BRP44	0.009204
SPON1	0.009274
PDCD4	0.00929
KIAA1383	0.00931
LRRC42	0.009384
SDC2	0.009384
FAM131A	0.009467
MTCH1	0.009467
WDR35	0.009505
HEXB	0.009505
MACROD2	0.009672
NTN1	0.009694
NECAB3	0.009806
PRNP	0.009816
ZNF786	0.009827
DEK	0.009853
INPP5D	0.009936
TSKS	0.00995
NEGR1	0.010026
SLC9A9	0.010151
CCNG2	0.010158
SMPDL3A	0.010363

COCH	0.010363
ATP1B2	0.01037
SOX4	0.01038
EXOG	0.010407
DACH1	0.01044
GTF2H4	0.010469
HDAC1	0.010489
TUFT1	0.010489
AAK1	0.010602
ANKRD35	0.010694
MARCKSL1	0.010709
AASS	0.010737
CUL7	0.010784
CR1L	0.010832
GAS2L3	0.010865
GTSF1	0.010923
NBN	0.010991
FAM69B	0.011125
ADARB1	0.011189
LRRC32	0.011211
KIAA0020	0.011387
DECR2	0.011493
SNAI1	0.011493
CAPG	0.011616
TXNDC5	0.011741
COBLL1	0.011754
SRPK1	0.011754
PNMT	0.011801
RBM47	0.011931
RAPGEF1	0.012055
STAT5A	0.012056
TRAF3IP2	0.012211
SLC6A9	0.012275
C9orf89	0.012305
LAYN	0.012405
CCDC24	0.012513
APOL3	0.012552
HSDL1	0.012584
TRIM46	0.012613
PRKD2	0.012693
CALML4	0.012735
YARS2	0.012891
ALPK3	0.012927
TMEM25	0.013065
MN1	0.013066
TMCO3	0.013179
RHOH	0.013188

CDK9	0.013188
DFNA5	0.013244
KCNA3	0.013341
DPYSL3	0.013341
RPS6KL1	0.013341
P2RX4	0.013381
SLC16A6	0.013386
CBFA2T3	0.013403
PTK2	0.013452
C21orf7	0.013467
MRPS24	0.01347
SLC26A11	0.013579
LOC388588	0.013732
TMEM65	0.013911
CASZ1	0.013924
TARS	0.013984
SPRYD4	0.01407
MCAM	0.014076
FAM20C	0.014105
FBP1	0.014167
PDGFA	0.014222
NCOR2	0.014222
GLB1L	0.014303
KRI1	0.014303
PYCR1	0.014353
BCL2	0.014475
BPGM	0.014516
GRAMD3	0.014536
ELMO1	0.014605
MYB	0.014621
KDM1A	0.014635
SLC25A42	0.014635
DNAJB2	0.014688
LSM14B	0.01477
CLCN5	0.01479
TUBA4B	0.014809
CERK	0.014849
FZD2	0.014883
TPCN1	0.015036
CDC123	0.01521
MYO1C	0.015264
LOC146880	0.015266
CD320	0.015313
MPEG1	0.015331
SLC25A24	0.015537
TOP1MT	0.015539
VAMP2	0.015607

TNFSF14	0.015607
SETDB2	0.015649
DCAF12	0.015769
MBTPS1	0.015811
RAB33A	0.01584
COMMD8	0.015849
SAMD8	0.015859
N4BP2L1	0.015933
FAM40B	0.015989
KIAA0664	0.016004
PRKCE	0.01601
KLRG2	0.016116
AR	0.016207
PIP4K2A	0.016272
VWF	0.016291
ULBP2	0.016354
TRIM62	0.01636
LDLRAP1	0.01636
PTPLAD1	0.016437
ITGA4	0.016519
CDK4	0.016663
SLC20A1	0.016905
PSD3	0.016934
KIAA0556	0.016956
KCNJ2	0.017019
AIFM2	0.017063
PLEKHM1	0.017067
HMGN3	0.0172
LARP1	0.017261
PPFIBP2	0.017261
GCDH	0.017538
CDK6	0.017666
LITAF	0.017724
HTR7	0.017794
BTG2	0.017897
DNAJC12	0.017927
ADM	0.017992
MYO1F	0.018077
MLLT6	0.018205
MS4A2	0.018659
CDKN2A	0.018784
ZBTB16	0.018845
SLC29A1	0.019083
RLTPR	0.019083
RAB40B	0.01952
IL12A	0.019529
KCTD7	0.019539

SCARA3	0.019712
SH3BP2	0.01978
SVIL	0.01978
BLNK	0.019807
TCTN3	0.019834
TICAM1	0.01993
GNPDA1	0.020247
RABGAP1	0.020316
RPAP1	0.020389
BASP1	0.020425
AOAH	0.020445
RAC3	0.020469
SLC46A2	0.020534
IL11RA	0.020546
SLC27A5	0.020584
PANK1	0.020605
SULF2	0.02075
C1orf54	0.020948
GFI1	0.021131
OBFC1	0.021131
CLECL1	0.021249
SPSB1	0.02154
STAB1	0.021544
AIF1	0.021553
CD48	0.02168
RECK	0.021739
AARSD1	0.022084
HOXA4	0.02211
LCAT	0.022142
KDM5B	0.022183
FAM70A	0.022325
MAP1A	0.022337
KCTD12	0.022362
DEAF1	0.02239
SOD1	0.022397
ARHGAP6	0.022418
UPK3A	0.022422
SLC44A3	0.022498
CORO1C	0.022568
NMD3	0.022703
LDLRAD3	0.022888
PAFAH2	0.022949
IL10RA	0.022986
LTK	0.023013
TSC22D2	0.023044
SSBP2	0.023357
CHST15	0.023357

TRIM38	0.023443
LOXHD1	0.023461
TMEM111	0.023832
NDRG2	0.023832
CXCL16	0.023878
ARHGEF5	0.024163
RAPH1	0.02424
PLVAP	0.024418
TFEB	0.024633
TP53BP1	0.024673
TRUB2	0.024792
TTTY15	0.024797
ART4	0.024797
HOXC4	0.024807
SEMA4D	0.024837
COX10	0.024914
MMP28	0.024949
WDR92	0.025063
CD86	0.025082
GPC6	0.025105
IPO4	0.025418
FAM65A	0.025418
ARHGEF10L	0.026184
SMARCA2	0.026184
ATP5H	0.026624
C1orf43	0.02673
ZCCHC7	0.026783
TBC1D12	0.027163
TFB2M	0.027251
YWHAE	0.027342
ZNF532	0.027342
ITPK1	0.027517
CCR5	0.027757
PHLDA1	0.027791
GTF2IRD1	0.028108
NANOS1	0.028108
GRIK5	0.02814
HES1	0.028186
THBD	0.028407
ACHE	0.028472
AMFR	0.028472
GABBR1	0.028501
RNF168	0.028829
GLMN	0.028863
SCAMP5	0.02908
OGG1	0.029097
ABCA2	0.02932

ZMYM6	0.029569
TNFSF10	0.029673
CD69	0.029673
DSC2	0.029874
UBQLN4	0.029915
ABHD12B	0.030026
DENR	0.030103
C18orf1	0.0302
DPM2	0.030359
TNFRSF11A	0.030515
TMEM40	0.030613
KIF21A	0.030613
B4GALT2	0.030734
MAFG	0.030734
CDC42EP1	0.030842
ST14	0.031036
NBEA	0.031036
CRHBP	0.031138
CARHSP1	0.03122
PGM2L1	0.031262
LRP1	0.031292
ATG16L2	0.031543
LDOC1	0.031652
ELAVL4	0.031817
NOTCH2NL	0.031893
EPB41L3	0.031893
PDZD8	0.032158
KLHDC9	0.032498
INE1	0.032601
ZFYVE9	0.032607
SLCO5A1	0.032677
MICALCL	0.03293
LAX1	0.033053
RAB4A	0.033053
ACN9	0.033067
ABCC3	0.033135
GRPEL1	0.033176
FAM196A	0.033339
WSB1	0.033493
NYNRIN	0.033495
NRIP3	0.033517
B3GALTL	0.033883
C21orf63	0.033883
HIVEP3	0.034055
CDKN1B	0.034055
ERBB2IP	0.034104
INPP4B	0.034179

LOC728819	0.03463
BEX1	0.03463
ZNF419	0.03463
CCDC159	0.034841
CCDC149	0.035029
FKBP15	0.035129
PHF2	0.035129
PLCG1	0.035129
ZFP36	0.035149
LY96	0.035305
SAMD13	0.035365
TRIM2	0.035381
FAM176B	0.035792
TPP2	0.03599
LGSN	0.036257
MTSS1	0.036335
GAS6	0.036371
KIAA1958	0.036472
ANKS6	0.036743
RPL17	0.036779
TMEM102	0.036829
HLX	0.036857
PHF7	0.036857
FAM179B	0.036895
TNFAIP1	0.036895
PTRF	0.036994
ATP10A	0.037162
MGC12982	0.037164
MINA	0.037679
ZNF334	0.037679
MLLT4	0.037718
PKIB	0.037774
XPO6	0.037774
ARID5A	0.037877
SLC25A28	0.038128
ZNF597	0.038138
BEND6	0.038358
HOXA6	0.03864
STX12	0.038728
NDFIP2	0.038761
LOC149134	0.038875
C3orf45	0.03903
FAM198B	0.03903
SPATA13	0.039381
ARHGEF11	0.039562
C20orf29	0.039562
ENTPD7	0.039602

HMOX1	0.040033
TRANK1	0.040318
LNP1	0.04044
MYC	0.040569
KIAA1462	0.040569
RPS6KA1	0.040613
COL5A1	0.040613
SASH3	0.040957
FAM101B	0.040962
TARSL2	0.041075
NAPSB	0.041176
TBC1D8	0.042136
WDR12	0.042136
COPZ2	0.042136
ZNF74	0.042287
HEG1	0.042358
TRIP10	0.042678
AFMID	0.042881
ZFP36L1	0.0429
PTPN9	0.043366
ABCA7	0.04338
GGT5	0.043545
CCDC78	0.043552
CORO2B	0.044141
ARHGAP22	0.044202
TCP11L1	0.044563
MFNG	0.044742
NT5C2	0.044956
NUP93	0.045285
ABCC1	0.04533
LIMD1	0.045679
HIP1R	0.04576
LRRC16A	0.045858
HPSE	0.045948
TMEM63B	0.045967
UCHL1	0.046243
SIPA1L1	0.046262
AADAT	0.046546
DHCR24	0.046785
ABCC4	0.047058
NHS	0.04707
FADS1	0.047266
MALT1	0.047499
ABTB1	0.047657
UBA6	0.047657
SLC24A6	0.047657
ASB12	0.047817

TBC1D13	0.048115
HSPA5	0.048246
PDGFC	0.048264
APOLD1	0.048286
GALNT7	0.048616
SKIL	0.04866
GLCE	0.04866
SEMA3C	0.048688
MLLT10	0.048865
RPAIN	0.049456
CLASP2	0.049544
TGFB1I1	0.049909

List of genes used in the over-representation analysis that were significant to significance level 0.10 in the validation analysis of the comparison *RUNX1*+ vs. *RUNX1*- together with the corresponding (unadjusted) *p* values obtained in the validation analysis

Variable	*p* value from validation analysis (using AMLCG Cohort 1)
C10orf58	1.5E-06
BEND4	1.53E-05
CD109	0.000031
SCRN1	8.67E-05
KIAA0125	0.0003
IFITM3	0.000351
SLITRK5	0.000551
HOPX	0.000702
CHRDL1	0.00129
FAM171B	0.001593
FHL1	0.001643
CCDC85C	0.001881
SH3BP4	0.002814
C21orf63	0.003149
CYP2E1	0.004187
H1F0	0.004898
HMGA2	0.005262
ARHGEF10	0.006078
HTR1F	0.006274
TRPS1	0.007286
CCNA1	0.007855
IL12RB2	0.009275
SEMA3C	0.014794
ZNF135	0.018876
CMTM4	0.021011
GPR34	0.021433
FAM169A	0.022676
KIAA1804	0.023177
FRMD4B	0.023434
SLC12A7	0.024127
KLHL3	0.024173
USP18	0.024434
CLCN5	0.025957
BAI3	0.026028
OSBPL5	0.026974

ACSL3	0.02754
PIWIL4	0.029474
RASD1	0.035128
PLXNC1	0.035261
LRRC16A	0.036575
RFTN1	0.037568
PSRC1	0.039053
LOC283663	0.039097
AMOT	0.040024
HDC	0.041078
ANKMY2	0.042015
MLLT3	0.04233
SLC16A1	0.042495
PEAR1	0.05266
IGF2BP3	0.055242
ADM	0.056334
COBL	0.05785
AAK1	0.062933
ZNF193	0.06578
FTO	0.069394
ARHGAP31	0.071813
ABCG1	0.073791
MYEF2	0.075051
SLC41A1	0.079714
INHBA	0.09547
KLF9	0.096034
CDCP1	0.096192

List of genes used in the over-representation analysis that were significant to significance level 0.10 in the validation analysis of the comparison t(8;21)+ vs. t(8;21)- together with the corresponding (unadjusted) *p* values obtained in the validation analysis

Variable	*p* value from validation analysis (using AMLCG Cohort 1)
FAM92A1	3E-07
FADS1	2.4E-06
GALNT12	1.17E-05
CD109	0.000013
PHGDH	1.32E-05
GPR56	1.38E-05
DDIT4	2.05E-05
B3GNT1	2.36E-05
CLU	2.54E-05
NGFRAP1	3.09E-05
DOCK1	3.99E-05
SH3BP4	6.19E-05
IRX1	6.33E-05
NRBP1	0.000066
WBP5	8.22E-05
KIAA0125	8.89E-05
SYNJ2	0.000116
F12	0.000196
DLC1	0.000202
HDAC4	0.000328
MICALL2	0.000354
DNMT3B	0.000382
ZC3H12C	0.000472
GPR114	0.000653
HOPX	0.000653
GC18M052797_at	0.000669
TANC1	0.000769
ACOT7	0.000805
MMRN1	0.001215
TM4SF1	0.001237
ALDH2	0.001252
ARHGAP22	0.001342
HOMER3	0.001414
SAP30L	0.001452
ADRM1	0.001667

ATP13A2	0.001891
CALCRL	0.001994
KDELC1	0.002079
ZNF334	0.002149
ADRBK1	0.00215
MBTPS1	0.002194
C16orf93	0.002224
SCHIP1	0.002277
BEX2	0.002348
SEL1L3	0.002396
GOLGA8A	0.002537
IGF2BP3	0.002735
PCTP	0.002863
BIVM	0.002913
TPK1	0.002956
NYNRIN	0.002968
NCRNA00205	0.002999
MPO	0.003017
CYB5A	0.003148
HTR1F	0.003168
RFTN1	0.00325
TRAT1	0.003447
TSC22D4	0.003516
CCNG2	0.003836
YPEL2	0.003849
RAB27A	0.00386
TMEM163	0.004003
PCCA	0.00411
WDR54	0.00422
PYGB	0.004234
KIAA1274	0.004376
HMGN5	0.004837
KCTD15	0.004962
PRKCZ	0.005653
CCDC102A	0.005822
SLC25A12	0.005867
HOXA9	0.005902
TSEN15	0.006206
TSPAN2	0.006244
C7orf23	0.0069
C1orf186	0.007715
HAVCR2	0.008576
SPATS2L	0.008752
ZMIZ1	0.008816
RAB40B	0.008917
PCNX	0.009224
RAB43	0.009387

CUL9	0.009712
RNF44	0.010195
RDX	0.010657
FUT4	0.010907
CMTM4	0.010989
DPP9	0.011267
PLXNC1	0.011481
FRMD4B	0.01154
ZNF532	0.012205
SOLH	0.012648
MEF2C	0.012655
RAB5B	0.013157
VSTM1	0.013759
P2RX4	0.013967
NCRNA00173	0.013989
SH3BP5	0.014296
CPT1B	0.014386
CFD	0.014626
PHKA1	0.014888
STARD9	0.015106
TAF1C	0.0152
TMEM25	0.015291
NOTCH1	0.0155
INO80C	0.015745
CHIC1	0.016284
TMEM38B	0.016289
GC19P054794_at	0.016569
RPS6KL1	0.017649
ARL8A	0.01772
KPTN	0.018534
ITPKA	0.018739
ZC3HAV1	0.018852
MXRA7	0.019704
VAV1	0.020113
SREBF2	0.020437
NPDC1	0.020891
CPNE8	0.021398
MAP7	0.02142
HEXA	0.021736
CDR2	0.02223
ATP2B4	0.022364
RUNX3	0.022376
GNB5	0.022561
LAMA5	0.022773
SNAP47	0.024273
TXNIP	0.024731
TSC2	0.024971

ZEB1	0.024989
STAR	0.025573
CD82	0.025908
MLX	0.026422
TMEM216	0.026846
HSDL1	0.027065
FAM169A	0.027211
SLC12A7	0.027524
RNF130	0.028187
HERPUD2	0.028296
DEAF1	0.028393
MKNK2	0.028701
MAP1A	0.029185
PPAP2A	0.029774
TES	0.029914
NRD1	0.029939
WHAMML1	0.029956
ATP11A	0.030406
ANKS6	0.031032
GOLGA8B	0.031628
LARP1	0.033764
COMMD7	0.034099
MSI2	0.034108
GPR157	0.034981
MED12L	0.035056
AKT3	0.035454
AGTPBP1	0.0356
EDC4	0.036809
LPHN1	0.037045
PABPC4	0.037284
HSF5	0.037428
STARD10	0.038102
CLCN5	0.038108
LGSN	0.039505
USP45	0.039586
SFXN3	0.039598
TRIO	0.040723
PSMD5	0.040883
ETFB	0.041513
EI24	0.042518
ERMP1	0.042574
CTSF	0.042703
CRLF3	0.043431
TGIF1	0.045136
OBSL1	0.045233
ATL3	0.045892
ABCG1	0.04659

TNNT1	0.046813
ZNF438	0.046832
LY6G5C	0.048107
TSC22D1	0.048203
SC5DL	0.048415
GC17M016189_at	0.04873
ARMCX2	0.048768
SPP1	0.049073
CAMKK2	0.049337
FYCO1	0.04953
CCND3	0.049906
MID1IP1	0.050136
MAP3K1	0.051883
CLIP2	0.052332
AKAP2	0.054482
FAM100A	0.05491
TMEM9	0.055033
DPPA4	0.055653
ZNF711	0.055849
DNM3	0.055933
PRDM16	0.057061
VPS28	0.057344
CD59	0.057912
ENO2	0.058316
CLEC12B	0.05893
RABGAP1	0.058987
DCP2	0.059096
ABCA2	0.059188
SORBS3	0.060154
C11orf63	0.060297
PCGF1	0.060377
BSDC1	0.060389
IL7	0.061242
MARCKSL1	0.061864
PLSCR3	0.062599
CALR	0.063182
CD164	0.064037
STK10	0.064995
HARS	0.066741
TRIP10	0.067294
COL18A1	0.068721
CDK12	0.068921
PRKX	0.069385
EIF5A2	0.06941
LY75	0.070135
TLR2	0.070154
TNFRSF4	0.07084

REXO1	0.071105
MRPS22	0.071235
ESYT1	0.071678
RAB27B	0.072945
DTD1	0.073146
LPIN1	0.074487
SAMD9	0.076723
RASGRP2	0.081639
PPFIBP1	0.081791
PDE2A	0.082398
GUSB	0.082546
EFCAB2	0.082922
LCT	0.083932
TRAF7	0.085182
PTPRA	0.085931
RNF8	0.08624
MOGS	0.086471
SERPING1	0.086497
TNFSF4	0.086749
RNF31	0.086831
CNOT8	0.086993
GC14P100990_at	0.087777
ENSG00000227403	0.089545
SSPN	0.090212
PPCDC	0.093091
RNASET2	0.096315
GC11P003833_at	0.09694
UBE2I	0.097697
ANKRD50	0.099775
