# Supplementary material for: Mediation analysis reveals common mechanisms of RUNX1 point mutations and RUNX1/RUNX1T1 fusions influencing survival of patients with acute myeloid leukemia
Source: Sci Rep. 2018 Jul 26;8:11293. doi: 10.1038/s41598-018-29593-2 (PMC6062501; doi:10.1038/s41598-018-29593-2)
Supplement: Supplementary file 2 — Electronic appendix [file 41598_2018_29593_MOESM2_ESM.zip › Hornungetal2018_ElectronicAppendix/Results/FilesOverrepresentationAnalysis/RUNX1_OverrepresentationAnalysis_Output.docx]

| **Results summary** |  |
| --- | --- |
| uploaded list: | 62 |
| [mapped entities:](http://cpdb.molgen.mpg.de/CPDB/showTranslation) | [54](http://cpdb.molgen.mpg.de/CPDB/showTranslation) |
| [enriched neighborhood-based sets (NESTs):](http://cpdb.molgen.mpg.de/CPDB/findSignificantClusters#nests) | [1](http://cpdb.molgen.mpg.de/CPDB/findSignificantClusters#nests) |
| [enriched pathway-based sets:](http://cpdb.molgen.mpg.de/CPDB/findSignificantClusters#pests) | [3](http://cpdb.molgen.mpg.de/CPDB/findSignificantClusters#pests) |
| [enriched gene ontology-based sets:](http://cpdb.molgen.mpg.de/CPDB/findSignificantClusters#gosets) | [54](http://cpdb.molgen.mpg.de/CPDB/findSignificantClusters#gosets) |

Formularbeginn

| Enriched neighborhood-based sets (NESTs) [(download)](http://cpdb.molgen.mpg.de/CPDB/downloadEnrSets?typ=n) | | | | | | | |
| --- | --- | --- | --- | --- | --- | --- | --- |
| [**54** genes (100.0%)](http://cpdb.molgen.mpg.de/CPDB/showTranslation?highlight=Ng) from the input list are present in at least one NEST. The total number of genes present in at least one NEST and identifiable by 'hgnc-symbol' IDs is **18242**. | | | | | | | |
| **select all none** | **set centers** | **radius** | **set size** | **candidates contained** | **p-value** | **q-value** | **set sources** |
|  | q2m296-4 | 1 | [**39**](http://cpdb.molgen.mpg.de/CPDB/showSetDetails?sp=n&st=0) | [**3 (7.7%)**](http://cpdb.molgen.mpg.de/CPDB/showSetDetails?sp=n&st=0) | 0.000208 | 0.675 | **I** **B** |
| Enriched pathway-based sets [(download)](http://cpdb.molgen.mpg.de/CPDB/downloadEnrSets?typ=p) (show word cloud) | | | | | | | |
|  | | | | | | | |
| [**40** genes (74.1%)](http://cpdb.molgen.mpg.de/CPDB/showTranslation?highlight=Pg) from the input list are present in at least one pathway. The total number of genes present in at least one pathway and identifiable by 'hgnc-symbol' IDs is **12655**. | | | | | | | |
| **select all none** | **pathway name** | | **set size** | **candidates contained** | **p-value** | **q-value** | **pathway source** |
|  | DNA Damage/Telomere Stress Induced Senescence | | [**28**](http://cpdb.molgen.mpg.de/CPDB/showSetDetails?sp=p&st=0) | [**3 (10.7%)**](http://cpdb.molgen.mpg.de/CPDB/showSetDetails?sp=p&st=0) | 9.07e-05 | 0.00327 | **Reactome** |
|  | Formation of Senescence-Associated Heterochromatin Foci (SAHF) | | [**17**](http://cpdb.molgen.mpg.de/CPDB/showSetDetails?sp=p&st=1) | [**2 (11.8%)**](http://cpdb.molgen.mpg.de/CPDB/showSetDetails?sp=p&st=1) | 0.00129 | 0.0231 | **Reactome** |
|  | Nuclear Receptors in Lipid Metabolism and Toxicity | | [**35**](http://cpdb.molgen.mpg.de/CPDB/showSetDetails?sp=p&st=2) | [**2 (5.7%)**](http://cpdb.molgen.mpg.de/CPDB/showSetDetails?sp=p&st=2) | 0.00543 | 0.0651 | **Wikipathways** |
| Enriched gene ontology-based sets [(download)](http://cpdb.molgen.mpg.de/CPDB/downloadEnrSets?typ=g) (show word cloud) | | | | | | | |
|  | | | | | | | |
| [**53** genes (98.1%)](http://cpdb.molgen.mpg.de/CPDB/showTranslation?highlight=G) from the input list are present in at least one GO category. The total number of genes present in at least one GO category and identifiable by 'hgnc-symbol' IDs is **18839**. | | | | | | | |
| **select all none** | **gene ontology term** | **category, level** | **set size** | **candidates contained** | **p-value** | **q-value** |  |
|  | GO:0040008   regulation of growth | BP 3 | [**644**](http://cpdb.molgen.mpg.de/CPDB/showSetDetails?sp=g&st=0) | [**9 (1.4%)**](http://cpdb.molgen.mpg.de/CPDB/showSetDetails?sp=g&st=0) | 6.87e-05 | 0.0111 |  |
|  | GO:0030308   negative regulation of cell growth | BP 5 | [**172**](http://cpdb.molgen.mpg.de/CPDB/showSetDetails?sp=g&st=1) | [**5 (2.9%)**](http://cpdb.molgen.mpg.de/CPDB/showSetDetails?sp=g&st=1) | 0.00012 | 0.0192 |  |
|  | GO:0043116   negative regulation of vascular permeability | BP 4 | [**9**](http://cpdb.molgen.mpg.de/CPDB/showSetDetails?sp=g&st=2) | [**2 (22.2%)**](http://cpdb.molgen.mpg.de/CPDB/showSetDetails?sp=g&st=2) | 0.000276 | 0.0537 |  |
|  | GO:0016049   cell growth | BP 2 | [**501**](http://cpdb.molgen.mpg.de/CPDB/showSetDetails?sp=g&st=3) | [**7 (1.4%)**](http://cpdb.molgen.mpg.de/CPDB/showSetDetails?sp=g&st=3) | 0.000477 | 0.0148 |  |
|  | GO:0009653   anatomical structure morphogenesis | BP 2 | [**2366**](http://cpdb.molgen.mpg.de/CPDB/showSetDetails?sp=g&st=4) | [**16 (0.7%)**](http://cpdb.molgen.mpg.de/CPDB/showSetDetails?sp=g&st=4) | 0.000547 | 0.0148 |  |
|  | GO:0045926   negative regulation of growth | BP 4 | [**242**](http://cpdb.molgen.mpg.de/CPDB/showSetDetails?sp=g&st=5) | [**5 (2.1%)**](http://cpdb.molgen.mpg.de/CPDB/showSetDetails?sp=g&st=5) | 0.000583 | 0.0537 |  |
|  | GO:0001558   regulation of cell growth | BP 4 | [**396**](http://cpdb.molgen.mpg.de/CPDB/showSetDetails?sp=g&st=6) | [**6 (1.5%)**](http://cpdb.molgen.mpg.de/CPDB/showSetDetails?sp=g&st=6) | 0.000817 | 0.0537 |  |
|  | GO:0050793   regulation of developmental process | BP 3 | [**2225**](http://cpdb.molgen.mpg.de/CPDB/showSetDetails?sp=g&st=7) | [**15 (0.7%)**](http://cpdb.molgen.mpg.de/CPDB/showSetDetails?sp=g&st=7) | 0.000881 | 0.0491 |  |
|  | GO:0021756   striatum development | BP 3 | [**16**](http://cpdb.molgen.mpg.de/CPDB/showSetDetails?sp=g&st=8) | [**2 (12.5%)**](http://cpdb.molgen.mpg.de/CPDB/showSetDetails?sp=g&st=8) | 0.000909 | 0.0491 |  |
|  | GO:0006820   anion transport | BP 5 | [**561**](http://cpdb.molgen.mpg.de/CPDB/showSetDetails?sp=g&st=9) | [**7 (1.2%)**](http://cpdb.molgen.mpg.de/CPDB/showSetDetails?sp=g&st=9) | 0.00094 | 0.0747 |  |
|  | GO:0048589   developmental growth | BP 2 | [**580**](http://cpdb.molgen.mpg.de/CPDB/showSetDetails?sp=g&st=10) | [**7 (1.2%)**](http://cpdb.molgen.mpg.de/CPDB/showSetDetails?sp=g&st=10) | 0.00114 | 0.0177 |  |
|  | GO:0007379   segment specification | BP 4 | [**18**](http://cpdb.molgen.mpg.de/CPDB/showSetDetails?sp=g&st=11) | [**2 (11.1%)**](http://cpdb.molgen.mpg.de/CPDB/showSetDetails?sp=g&st=11) | 0.00115 | 0.0537 |  |
|  | GO:0048646   anatomical structure formation involved in morphogenesis | BP 2 | [**960**](http://cpdb.molgen.mpg.de/CPDB/showSetDetails?sp=g&st=12) | [**9 (0.9%)**](http://cpdb.molgen.mpg.de/CPDB/showSetDetails?sp=g&st=12) | 0.00131 | 0.0177 |  |
|  | GO:0048638   regulation of developmental growth | BP 4 | [**296**](http://cpdb.molgen.mpg.de/CPDB/showSetDetails?sp=g&st=13) | [**5 (1.7%)**](http://cpdb.molgen.mpg.de/CPDB/showSetDetails?sp=g&st=13) | 0.00143 | 0.0537 |  |
|  | GO:0008361   regulation of cell size | BP 4 | [**175**](http://cpdb.molgen.mpg.de/CPDB/showSetDetails?sp=g&st=14) | [**4 (2.3%)**](http://cpdb.molgen.mpg.de/CPDB/showSetDetails?sp=g&st=14) | 0.00148 | 0.0537 |  |
|  | GO:0048729   tissue morphogenesis | BP 3 | [**615**](http://cpdb.molgen.mpg.de/CPDB/showSetDetails?sp=g&st=15) | [**7 (1.1%)**](http://cpdb.molgen.mpg.de/CPDB/showSetDetails?sp=g&st=15) | 0.0016 | 0.0561 |  |
|  | GO:0021544   subpallium development | BP 3 | [**22**](http://cpdb.molgen.mpg.de/CPDB/showSetDetails?sp=g&st=16) | [**2 (9.1%)**](http://cpdb.molgen.mpg.de/CPDB/showSetDetails?sp=g&st=16) | 0.00173 | 0.0561 |  |
|  | GO:2000026   regulation of multicellular organismal development | BP 4 | [**1694**](http://cpdb.molgen.mpg.de/CPDB/showSetDetails?sp=g&st=17) | [**12 (0.7%)**](http://cpdb.molgen.mpg.de/CPDB/showSetDetails?sp=g&st=17) | 0.00216 | 0.0672 |  |
|  | GO:0035282   segmentation | BP 5 | [**96**](http://cpdb.molgen.mpg.de/CPDB/showSetDetails?sp=g&st=18) | [**3 (3.1%)**](http://cpdb.molgen.mpg.de/CPDB/showSetDetails?sp=g&st=18) | 0.0025 | 0.132 |  |
|  | GO:0042221   response to chemical | BP 2 | [**4129**](http://cpdb.molgen.mpg.de/CPDB/showSetDetails?sp=g&st=19) | [**21 (0.5%)**](http://cpdb.molgen.mpg.de/CPDB/showSetDetails?sp=g&st=19) | 0.00262 | 0.0283 |  |
|  | GO:0010876   lipid localization | BP 3 | [**342**](http://cpdb.molgen.mpg.de/CPDB/showSetDetails?sp=g&st=20) | [**5 (1.5%)**](http://cpdb.molgen.mpg.de/CPDB/showSetDetails?sp=g&st=20) | 0.00266 | 0.0718 |  |
|  | GO:0045595   regulation of cell differentiation | BP 4 | [**1517**](http://cpdb.molgen.mpg.de/CPDB/showSetDetails?sp=g&st=21) | [**11 (0.7%)**](http://cpdb.molgen.mpg.de/CPDB/showSetDetails?sp=g&st=21) | 0.00282 | 0.0768 |  |
|  | GO:0031252   cell leading edge | CC 2 | [**354**](http://cpdb.molgen.mpg.de/CPDB/showSetDetails?sp=g&st=22) | [**5 (1.4%)**](http://cpdb.molgen.mpg.de/CPDB/showSetDetails?sp=g&st=22) | 0.00312 | 0.109 |  |
|  | GO:0022804   active transmembrane transporter activity | MF 3 | [**357**](http://cpdb.molgen.mpg.de/CPDB/showSetDetails?sp=g&st=23) | [**5 (1.4%)**](http://cpdb.molgen.mpg.de/CPDB/showSetDetails?sp=g&st=23) | 0.0032 | 0.0959 |  |
|  | GO:0043114   regulation of vascular permeability | BP 3 | [**30**](http://cpdb.molgen.mpg.de/CPDB/showSetDetails?sp=g&st=24) | [**2 (6.7%)**](http://cpdb.molgen.mpg.de/CPDB/showSetDetails?sp=g&st=24) | 0.00321 | 0.0743 |  |
|  | GO:0048333   mesodermal cell differentiation | BP 5 | [**32**](http://cpdb.molgen.mpg.de/CPDB/showSetDetails?sp=g&st=25) | [**2 (6.2%)**](http://cpdb.molgen.mpg.de/CPDB/showSetDetails?sp=g&st=25) | 0.00365 | 0.141 |  |
|  | GO:0071827   plasma lipoprotein particle organization | BP 3 | [**34**](http://cpdb.molgen.mpg.de/CPDB/showSetDetails?sp=g&st=26) | [**2 (5.9%)**](http://cpdb.molgen.mpg.de/CPDB/showSetDetails?sp=g&st=26) | 0.00411 | 0.0816 |  |
|  | GO:0015291   secondary active transmembrane transporter activity | MF 4 | [**234**](http://cpdb.molgen.mpg.de/CPDB/showSetDetails?sp=g&st=27) | [**4 (1.7%)**](http://cpdb.molgen.mpg.de/CPDB/showSetDetails?sp=g&st=27) | 0.00422 | 0.0587 |  |
|  | GO:0051239   regulation of multicellular organismal process | BP 3 | [**2622**](http://cpdb.molgen.mpg.de/CPDB/showSetDetails?sp=g&st=28) | [**15 (0.6%)**](http://cpdb.molgen.mpg.de/CPDB/showSetDetails?sp=g&st=28) | 0.00457 | 0.0816 |  |
|  | GO:0071825   protein-lipid complex subunit organization | BP 4 | [**37**](http://cpdb.molgen.mpg.de/CPDB/showSetDetails?sp=g&st=29) | [**2 (5.4%)**](http://cpdb.molgen.mpg.de/CPDB/showSetDetails?sp=g&st=29) | 0.00486 | 0.0962 |  |
|  | GO:0071526   semaphorin-plexin signaling pathway | BP 4 | [**37**](http://cpdb.molgen.mpg.de/CPDB/showSetDetails?sp=g&st=30) | [**2 (5.4%)**](http://cpdb.molgen.mpg.de/CPDB/showSetDetails?sp=g&st=30) | 0.00486 | 0.0962 |  |
|  | GO:0060612   adipose tissue development | BP 5 | [**37**](http://cpdb.molgen.mpg.de/CPDB/showSetDetails?sp=g&st=31) | [**2 (5.4%)**](http://cpdb.molgen.mpg.de/CPDB/showSetDetails?sp=g&st=31) | 0.00486 | 0.141 |  |
|  | GO:0043616   keratinocyte proliferation | BP 4 | [**38**](http://cpdb.molgen.mpg.de/CPDB/showSetDetails?sp=g&st=32) | [**2 (5.3%)**](http://cpdb.molgen.mpg.de/CPDB/showSetDetails?sp=g&st=32) | 0.00512 | 0.0962 |  |
|  | GO:0015485   cholesterol binding | MF 4 | [**38**](http://cpdb.molgen.mpg.de/CPDB/showSetDetails?sp=g&st=33) | [**2 (5.3%)**](http://cpdb.molgen.mpg.de/CPDB/showSetDetails?sp=g&st=33) | 0.00512 | 0.0587 |  |
|  | GO:0048869   cellular developmental process | BP 3 | [**3779**](http://cpdb.molgen.mpg.de/CPDB/showSetDetails?sp=g&st=34) | [**19 (0.5%)**](http://cpdb.molgen.mpg.de/CPDB/showSetDetails?sp=g&st=34) | 0.0053 | 0.0816 |  |
|  | GO:0035295   tube development | BP 3 | [**579**](http://cpdb.molgen.mpg.de/CPDB/showSetDetails?sp=g&st=35) | [**6 (1.0%)**](http://cpdb.molgen.mpg.de/CPDB/showSetDetails?sp=g&st=35) | 0.00554 | 0.0816 |  |
|  | GO:0010883   regulation of lipid storage | BP 4 | [**41**](http://cpdb.molgen.mpg.de/CPDB/showSetDetails?sp=g&st=36) | [**2 (4.9%)**](http://cpdb.molgen.mpg.de/CPDB/showSetDetails?sp=g&st=36) | 0.00594 | 0.0962 |  |
|  | GO:0008202   steroid metabolic process | BP 4 | [**264**](http://cpdb.molgen.mpg.de/CPDB/showSetDetails?sp=g&st=37) | [**4 (1.5%)**](http://cpdb.molgen.mpg.de/CPDB/showSetDetails?sp=g&st=37) | 0.00636 | 0.0962 |  |
|  | GO:0034260   negative regulation of GTPase activity | BP 4 | [**43**](http://cpdb.molgen.mpg.de/CPDB/showSetDetails?sp=g&st=38) | [**2 (4.7%)**](http://cpdb.molgen.mpg.de/CPDB/showSetDetails?sp=g&st=38) | 0.00651 | 0.0962 |  |
|  | GO:0032934   sterol binding | MF 4 | [**43**](http://cpdb.molgen.mpg.de/CPDB/showSetDetails?sp=g&st=39) | [**2 (4.7%)**](http://cpdb.molgen.mpg.de/CPDB/showSetDetails?sp=g&st=39) | 0.00651 | 0.0587 |  |
|  | GO:0015711   organic anion transport | BP 5 | [**425**](http://cpdb.molgen.mpg.de/CPDB/showSetDetails?sp=g&st=40) | [**5 (1.2%)**](http://cpdb.molgen.mpg.de/CPDB/showSetDetails?sp=g&st=40) | 0.00674 | 0.141 |  |
|  | GO:0035987   endodermal cell differentiation | BP 5 | [**45**](http://cpdb.molgen.mpg.de/CPDB/showSetDetails?sp=g&st=41) | [**2 (4.4%)**](http://cpdb.molgen.mpg.de/CPDB/showSetDetails?sp=g&st=41) | 0.00712 | 0.141 |  |
|  | GO:1902284   neuron projection extension involved in neuron projection guidance | BP 5 | [**45**](http://cpdb.molgen.mpg.de/CPDB/showSetDetails?sp=g&st=42) | [**2 (4.4%)**](http://cpdb.molgen.mpg.de/CPDB/showSetDetails?sp=g&st=42) | 0.00712 | 0.141 |  |
|  | GO:0030154   cell differentiation | BP 4 | [**3603**](http://cpdb.molgen.mpg.de/CPDB/showSetDetails?sp=g&st=43) | [**18 (0.5%)**](http://cpdb.molgen.mpg.de/CPDB/showSetDetails?sp=g&st=43) | 0.0074 | 0.0962 |  |
|  | GO:0044089   positive regulation of cellular component biogenesis | BP 4 | [**437**](http://cpdb.molgen.mpg.de/CPDB/showSetDetails?sp=g&st=44) | [**5 (1.1%)**](http://cpdb.molgen.mpg.de/CPDB/showSetDetails?sp=g&st=44) | 0.00748 | 0.0962 |  |
|  | GO:0051128   regulation of cellular component organization | BP 4 | [**2231**](http://cpdb.molgen.mpg.de/CPDB/showSetDetails?sp=g&st=45) | [**13 (0.6%)**](http://cpdb.molgen.mpg.de/CPDB/showSetDetails?sp=g&st=45) | 0.0075 | 0.0962 |  |
|  | GO:0005548   phospholipid transporter activity | MF 4 | [**49**](http://cpdb.molgen.mpg.de/CPDB/showSetDetails?sp=g&st=46) | [**2 (4.1%)**](http://cpdb.molgen.mpg.de/CPDB/showSetDetails?sp=g&st=46) | 0.00839 | 0.0587 |  |
|  | GO:0032233   positive regulation of actin filament bundle assembly | BP 5 | [**49**](http://cpdb.molgen.mpg.de/CPDB/showSetDetails?sp=g&st=47) | [**2 (4.1%)**](http://cpdb.molgen.mpg.de/CPDB/showSetDetails?sp=g&st=47) | 0.00839 | 0.148 |  |
|  | GO:0009888   tissue development | BP 3 | [**1765**](http://cpdb.molgen.mpg.de/CPDB/showSetDetails?sp=g&st=48) | [**11 (0.6%)**](http://cpdb.molgen.mpg.de/CPDB/showSetDetails?sp=g&st=48) | 0.00892 | 0.101 |  |
|  | GO:1901618   organic hydroxy compound transmembrane transporter activity | MF 3 | [**51**](http://cpdb.molgen.mpg.de/CPDB/showSetDetails?sp=g&st=49) | [**2 (3.9%)**](http://cpdb.molgen.mpg.de/CPDB/showSetDetails?sp=g&st=49) | 0.00907 | 0.136 |  |
|  | GO:0021915   neural tube development | BP 3 | [**155**](http://cpdb.molgen.mpg.de/CPDB/showSetDetails?sp=g&st=50) | [**3 (1.9%)**](http://cpdb.molgen.mpg.de/CPDB/showSetDetails?sp=g&st=50) | 0.00947 | 0.101 |  |
|  | GO:0048731   system development | BP 3 | [**4290**](http://cpdb.molgen.mpg.de/CPDB/showSetDetails?sp=g&st=51) | [**20 (0.5%)**](http://cpdb.molgen.mpg.de/CPDB/showSetDetails?sp=g&st=51) | 0.00967 | 0.101 |  |
|  | GO:0010033   response to organic substance | BP 3 | [**2850**](http://cpdb.molgen.mpg.de/CPDB/showSetDetails?sp=g&st=52) | [**15 (0.5%)**](http://cpdb.molgen.mpg.de/CPDB/showSetDetails?sp=g&st=52) | 0.00993 | 0.101 |  |
|  | GO:0001726   ruffle | CC 3 | [**158**](http://cpdb.molgen.mpg.de/CPDB/showSetDetails?sp=g&st=53) | [**3 (1.9%)**](http://cpdb.molgen.mpg.de/CPDB/showSetDetails?sp=g&st=53) | 0.00998 | 0.329 |  |

Formularende
