# Supplementary material for: Mediation analysis reveals common mechanisms of RUNX1 point mutations and RUNX1/RUNX1T1 fusions influencing survival of patients with acute myeloid leukemia
Source: Sci Rep. 2018 Jul 26;8:11293. doi: 10.1038/s41598-018-29593-2 (PMC6062501; doi:10.1038/s41598-018-29593-2)
Supplement: Supplementary file 2 — Electronic appendix [file 41598_2018_29593_MOESM2_ESM.zip › Hornungetal2018_ElectronicAppendix/Results/FilesOverrepresentationAnalysis/t821_OverrepresentationAnalysis_Output.docx]

| **Results summary** |  |
| --- | --- |
| uploaded list: | 252 |
| [mapped entities:](http://cpdb.molgen.mpg.de/CPDB/showTranslation) | [223](http://cpdb.molgen.mpg.de/CPDB/showTranslation) |
| [enriched neighborhood-based sets (NESTs):](http://cpdb.molgen.mpg.de/CPDB/findSignificantClusters#nests) | [547](http://cpdb.molgen.mpg.de/CPDB/findSignificantClusters#nests) |
| [enriched pathway-based sets:](http://cpdb.molgen.mpg.de/CPDB/findSignificantClusters#pests) | [20](http://cpdb.molgen.mpg.de/CPDB/findSignificantClusters#pests) |
| [enriched gene ontology-based sets:](http://cpdb.molgen.mpg.de/CPDB/findSignificantClusters#gosets) | [102](http://cpdb.molgen.mpg.de/CPDB/findSignificantClusters#gosets) |
| [enriched protein complex-based sets:](http://cpdb.molgen.mpg.de/CPDB/findSignificantClusters#cpx) | [6](http://cpdb.molgen.mpg.de/CPDB/findSignificantClusters#cpx) |

Formularbeginn

| Enriched neighborhood-based sets (NESTs) [(download)](http://cpdb.molgen.mpg.de/CPDB/downloadEnrSets?typ=n) | | | | | | | |
| --- | --- | --- | --- | --- | --- | --- | --- |
| [**217** genes (97.3%)](http://cpdb.molgen.mpg.de/CPDB/showTranslation?highlight=Ng) from the input list are present in at least one NEST. The total number of genes present in at least one NEST and identifiable by 'hgnc-symbol' IDs is **18242**. | | | | | | | |
| **select allnone** | **set centers** | **radius** | **set size** | **candidates contained** | **p-value** | **q-value** | **set sources** |
|  | PPOX | 2 | [**770**](http://cpdb.molgen.mpg.de/CPDB/showSetDetails?sp=n&st=0) | [**28 (3.6%)**](http://cpdb.molgen.mpg.de/CPDB/showSetDetails?sp=n&st=0) | 1.4e-07 | 0.000965 | **P** **H** **R** **D** **B** **I** **IH** **C** **S** **P** **E** **B** **PK** **P** **M** **M** |
|  | TACSTD2 | 2 | [**2192**](http://cpdb.molgen.mpg.de/CPDB/showSetDetails?sp=n&st=1) | [**53 (2.4%)**](http://cpdb.molgen.mpg.de/CPDB/showSetDetails?sp=n&st=1) | 2.27e-07 | 0.000965 | **M** **P** **H** **R** **D** **NB** **I** **I** **H** **C** **B** **SP** **E** **B** **P** **K** **P** **MI** **M** **M** |
|  | apmap_human | 2 | [**1539**](http://cpdb.molgen.mpg.de/CPDB/showSetDetails?sp=n&st=2) | [**42 (2.7%)**](http://cpdb.molgen.mpg.de/CPDB/showSetDetails?sp=n&st=2) | 2.33e-07 | 0.000965 | **M** **P** **H** **D** **R** **NB** **I** **I** **H** **C** **S** **PE** **B** **P** **B** **K** **PM** **M** **M** |
|  | CHML | 2 | [**2158**](http://cpdb.molgen.mpg.de/CPDB/showSetDetails?sp=n&st=3) | [**52 (2.4%)**](http://cpdb.molgen.mpg.de/CPDB/showSetDetails?sp=n&st=3) | 3.71e-07 | 0.00115 | **M** **P** **H** **D** **R** **NB** **I** **I** **H** **C** **B** **SP** **E** **B** **P** **K** **P** **IM** |
|  | 4xHC-TIMM9 | 2 | [**1537**](http://cpdb.molgen.mpg.de/CPDB/showSetDetails?sp=n&st=4) | [**41 (2.7%)**](http://cpdb.molgen.mpg.de/CPDB/showSetDetails?sp=n&st=4) | 5.89e-07 | 0.00146 | **M** **P** **H** **R** **D** **NB** **I** **I** **H** **C** **S** **PE** **B** **P** **B** **K** **PM** **M** **M** |
|  | RAB2B | 2 | [**2143**](http://cpdb.molgen.mpg.de/CPDB/showSetDetails?sp=n&st=5) | [**50 (2.3%)**](http://cpdb.molgen.mpg.de/CPDB/showSetDetails?sp=n&st=5) | 1.59e-06 | 0.00284 | **M** **P** **H** **I** **R** **N** **BI** **I** **H** **C** **B** **S** **PE** **B** **P** **K** **P** **MM** **D** **M** |
|  | q6w6m8_human; q16094_human | 2 | [**929**](http://cpdb.molgen.mpg.de/CPDB/showSetDetails?sp=n&st=6) | [**29 (3.2%)**](http://cpdb.molgen.mpg.de/CPDB/showSetDetails?sp=n&st=6) | 1.61e-06 | 0.00284 | **M** **P** **H** **D** **R** **B** **II** **C** **B** **S** **P** **E** **BP** **K** **P** **M** **M** **M** |
|  | RABGAP1L | 2 | [**591**](http://cpdb.molgen.mpg.de/CPDB/showSetDetails?sp=n&st=7) | [**22 (3.7%)**](http://cpdb.molgen.mpg.de/CPDB/showSetDetails?sp=n&st=7) | 2.3e-06 | 0.00357 | **P** **H** **B** **S** **P** **D** **RP** **P** **B** **P** **M** **I** **I** |
|  | RAB27B | 2 | [**1173**](http://cpdb.molgen.mpg.de/CPDB/showSetDetails?sp=n&st=8) | [**33 (2.8%)**](http://cpdb.molgen.mpg.de/CPDB/showSetDetails?sp=n&st=8) | 3.09e-06 | 0.00367 | **M** **P** **H** **R** **D** **NB** **I** **I** **C** **B** **S** **PB** **P** **P** **I** **M** **M** |
|  | ANXA3 | 2 | [**1238**](http://cpdb.molgen.mpg.de/CPDB/showSetDetails?sp=n&st=9) | [**34 (2.8%)**](http://cpdb.molgen.mpg.de/CPDB/showSetDetails?sp=n&st=9) | 3.34e-06 | 0.00367 | **M** **P** **H** **R** **D** **NB** **I** **I** **C** **S** **P** **EB** **P** **B** **K** **P** **MM** **M** |
|  | CHD9 | 2 | [**2452**](http://cpdb.molgen.mpg.de/CPDB/showSetDetails?sp=n&st=10) | [**54 (2.2%)**](http://cpdb.molgen.mpg.de/CPDB/showSetDetails?sp=n&st=10) | 3.42e-06 | 0.00367 | **M** **P** **H** **I** **R** **N** **BI** **I** **H** **C** **B** **S** **PE** **B** **P** **K** **P** **M** **PM** **D** |
|  | i3l0n3_human | 2 | [**2016**](http://cpdb.molgen.mpg.de/CPDB/showSetDetails?sp=n&st=11) | [**47 (2.3%)**](http://cpdb.molgen.mpg.de/CPDB/showSetDetails?sp=n&st=11) | 3.55e-06 | 0.00367 | **P** **H** **R** **D** **N** **B** **II** **H** **C** **S** **P** **E** **BP** **B** **P** **P** **M** |
|  | cs052_human | 2 | [**3075**](http://cpdb.molgen.mpg.de/CPDB/showSetDetails?sp=n&st=12) | [**63 (2.1%)**](http://cpdb.molgen.mpg.de/CPDB/showSetDetails?sp=n&st=12) | 4.68e-06 | 0.00397 | **M** **P** **H** **I** **R** **N** **BI** **I** **H** **C** **B** **S** **PE** **B** **P** **K** **P** **M** **PM** **D** |
|  | COLGALT1 | 2 | [**3222**](http://cpdb.molgen.mpg.de/CPDB/showSetDetails?sp=n&st=13) | [**65 (2.0%)**](http://cpdb.molgen.mpg.de/CPDB/showSetDetails?sp=n&st=13) | 4.92e-06 | 0.00397 | **M** **P** **H** **R** **D** **NB** **I** **I** **H** **C** **B** **SP** **E** **B** **P** **K** **P** **MP** **I** **M** **M** |
|  | AEBP2 | 2 | [**1974**](http://cpdb.molgen.mpg.de/CPDB/showSetDetails?sp=n&st=14) | [**46 (2.3%)**](http://cpdb.molgen.mpg.de/CPDB/showSetDetails?sp=n&st=14) | 5.07e-06 | 0.00397 | **M** **P** **H** **D** **R** **NB** **P** **I** **I** **C** **B** **SP** **E** **B** **P** **K** **P** **MP** **M** |
|  | ICAM2 | 2 | [**929**](http://cpdb.molgen.mpg.de/CPDB/showSetDetails?sp=n&st=15) | [**28 (3.0%)**](http://cpdb.molgen.mpg.de/CPDB/showSetDetails?sp=n&st=15) | 5.13e-06 | 0.00397 | **P** **H** **D** **R** **B** **I** **IC** **S** **P** **B** **P** **B** **PM** **M** **M** |
|  | RAB32 | 2 | [**1387**](http://cpdb.molgen.mpg.de/CPDB/showSetDetails?sp=n&st=16) | [**36 (2.6%)**](http://cpdb.molgen.mpg.de/CPDB/showSetDetails?sp=n&st=16) | 6.58e-06 | 0.00472 | **M** **P** **H** **R** **D** **NB** **I** **I** **H** **C** **B** **SP** **B** **P** **P** **M** **M** |
|  | CHMP3 | 2 | [**3187**](http://cpdb.molgen.mpg.de/CPDB/showSetDetails?sp=n&st=17) | [**64 (2.0%)**](http://cpdb.molgen.mpg.de/CPDB/showSetDetails?sp=n&st=17) | 7.32e-06 | 0.00472 | **M** **P** **H** **D** **R** **NB** **I** **I** **H** **C** **B** **SP** **E** **B** **P** **K** **P** **MP** **I** **M** **M** |
|  | e5rhg8_human | 2 | [**3260**](http://cpdb.molgen.mpg.de/CPDB/showSetDetails?sp=n&st=18) | [**65 (2.0%)**](http://cpdb.molgen.mpg.de/CPDB/showSetDetails?sp=n&st=18) | 7.34e-06 | 0.00472 | **M** **P** **H** **I** **D** **N** **BI** **I** **H** **C** **B** **S** **PE** **B** **R** **P** **K** **PM** **P** **M** |
|  | RRAS | 2 | [**2584**](http://cpdb.molgen.mpg.de/CPDB/showSetDetails?sp=n&st=19) | [**55 (2.1%)**](http://cpdb.molgen.mpg.de/CPDB/showSetDetails?sp=n&st=19) | 7.77e-06 | 0.00472 | **M** **P** **H** **R** **D** **NB** **I** **I** **H** **C** **B** **SP** **E** **B** **P** **K** **P** **MI** **M** **M** |
|  | k1328_human | 2 | [**2655**](http://cpdb.molgen.mpg.de/CPDB/showSetDetails?sp=n&st=20) | [**56 (2.1%)**](http://cpdb.molgen.mpg.de/CPDB/showSetDetails?sp=n&st=20) | 8.03e-06 | 0.00472 | **M** **P** **H** **R** **D** **NB** **P** **I** **I** **H** **C** **BS** **P** **E** **B** **P** **K** **PM** **P** **I** **M** |
|  | ca052_human | 2 | [**2993**](http://cpdb.molgen.mpg.de/CPDB/showSetDetails?sp=n&st=21) | [**61 (2.0%)**](http://cpdb.molgen.mpg.de/CPDB/showSetDetails?sp=n&st=21) | 8.37e-06 | 0.00472 | **M** **P** **H** **R** **D** **NB** **I** **I** **C** **B** **S** **PE** **B** **P** **K** **P** **M** **IM** **M** |
|  | HBA1 | 2 | [**3639**](http://cpdb.molgen.mpg.de/CPDB/showSetDetails?sp=n&st=22) | [**70 (1.9%)**](http://cpdb.molgen.mpg.de/CPDB/showSetDetails?sp=n&st=22) | 9.6e-06 | 0.00518 | **M** **P** **H** **D** **R** **NB** **P** **I** **I** **H** **C** **BS** **P** **E** **B** **P** **K** **PM** **P** **I** **M** **M** |
|  | LST2 | 2 | [**416**](http://cpdb.molgen.mpg.de/CPDB/showSetDetails?sp=n&st=23) | [**17 (4.1%)**](http://cpdb.molgen.mpg.de/CPDB/showSetDetails?sp=n&st=23) | 1.03e-05 | 0.0053 | **P** **H** **I** **R** **N** **B** **I** **IC** **B** **S** **P** **B** **P** **PM** **M** |
|  | TRAPPC13 | 2 | [**1665**](http://cpdb.molgen.mpg.de/CPDB/showSetDetails?sp=n&st=24) | [**40 (2.4%)**](http://cpdb.molgen.mpg.de/CPDB/showSetDetails?sp=n&st=24) | 1.19e-05 | 0.00542 | **M** **P** **H** **I** **R** **N** **BI** **I** **H** **C** **P** **P** **EB** **B** **K** **P** **P** **MS** **D** |
|  | TGFA Gene | 2 | [**1544**](http://cpdb.molgen.mpg.de/CPDB/showSetDetails?sp=n&st=25) | [**38 (2.5%)**](http://cpdb.molgen.mpg.de/CPDB/showSetDetails?sp=n&st=25) | 1.2e-05 | 0.00542 | **M** **P** **H** **R** **D** **NB** **P** **I** **I** **H** **C** **BS** **P** **E** **B** **P** **P** **MI** **M** |
|  | HMG20A | 2 | [**3240**](http://cpdb.molgen.mpg.de/CPDB/showSetDetails?sp=n&st=26) | [**64 (2.0%)**](http://cpdb.molgen.mpg.de/CPDB/showSetDetails?sp=n&st=26) | 1.21e-05 | 0.00542 | **M** **P** **H** **D** **R** **NB** **P** **I** **I** **H** **C** **SP** **E** **B** **P** **B** **K** **PM** **P** **M** **M** |
|  | POLE3 | 2 | [**2493**](http://cpdb.molgen.mpg.de/CPDB/showSetDetails?sp=n&st=27) | [**53 (2.1%)**](http://cpdb.molgen.mpg.de/CPDB/showSetDetails?sp=n&st=27) | 1.23e-05 | 0.00542 | **M** **P** **H** **D** **R** **NB** **I** **I** **H** **C** **B** **SP** **E** **B** **P** **K** **P** **MP** **I** **M** |
|  | CCZ1B | 2 | [**659**](http://cpdb.molgen.mpg.de/CPDB/showSetDetails?sp=n&st=28) | [**22 (3.3%)**](http://cpdb.molgen.mpg.de/CPDB/showSetDetails?sp=n&st=28) | 1.27e-05 | 0.00542 | **P** **H** **R** **D** **N** **B** **II** **C** **B** **S** **B** **P** **PM** |
|  | (RefSeq) alpha-N-acetylgalactosaminidase | 2 | [**151**](http://cpdb.molgen.mpg.de/CPDB/showSetDetails?sp=n&st=29) | [**10 (6.6%)**](http://cpdb.molgen.mpg.de/CPDB/showSetDetails?sp=n&st=29) | 1.34e-05 | 0.00548 | **H** **S** **B** **E** **R** **K** **BM** **I** **H** |
|  | ITGA2 | 2 | [**4109**](http://cpdb.molgen.mpg.de/CPDB/showSetDetails?sp=n&st=30) | [**76 (1.9%)**](http://cpdb.molgen.mpg.de/CPDB/showSetDetails?sp=n&st=30) | 1.37e-05 | 0.00548 | **M** **P** **H** **D** **R** **ND** **B** **P** **I** **I** **H** **CB** **S** **P** **E** **B** **P** **KP** **M** **P** **I** **M** **M** |
|  | synem_human | 2 | [**2445**](http://cpdb.molgen.mpg.de/CPDB/showSetDetails?sp=n&st=31) | [**52 (2.1%)**](http://cpdb.molgen.mpg.de/CPDB/showSetDetails?sp=n&st=31) | 1.47e-05 | 0.00561 | **P** **H** **D** **R** **N** **B** **II** **H** **C** **B** **S** **P** **EB** **P** **K** **P** **P** **I** **M** |
|  | q17r31-2; k7ele3_human; a2a4f6_human | 2 | [**1041**](http://cpdb.molgen.mpg.de/CPDB/showSetDetails?sp=n&st=32) | [**29 (2.8%)**](http://cpdb.molgen.mpg.de/CPDB/showSetDetails?sp=n&st=32) | 1.61e-05 | 0.00561 | **C** **H** **B** **I** **E** **R** **IK** **P** **B** **P** **M** **S** **ID** **H** |
|  | f2z2m5_human | 2 | [**1097**](http://cpdb.molgen.mpg.de/CPDB/showSetDetails?sp=n&st=33) | [**30 (2.7%)**](http://cpdb.molgen.mpg.de/CPDB/showSetDetails?sp=n&st=33) | 1.62e-05 | 0.00561 | **C** **H** **B** **S** **P** **I** **ER** **K** **P** **B** **P** **MD** **I** **I** **H** |
|  | CHM | 2 | [**2995**](http://cpdb.molgen.mpg.de/CPDB/showSetDetails?sp=n&st=34) | [**60 (2.0%)**](http://cpdb.molgen.mpg.de/CPDB/showSetDetails?sp=n&st=34) | 1.65e-05 | 0.00561 | **M** **P** **H** **D** **R** **NB** **I** **I** **H** **C** **S** **PE** **B** **P** **B** **K** **P** **PI** **M** |
|  | PTPRJ | 2 | [**3914**](http://cpdb.molgen.mpg.de/CPDB/showSetDetails?sp=n&st=35) | [**73 (1.9%)**](http://cpdb.molgen.mpg.de/CPDB/showSetDetails?sp=n&st=35) | 1.66e-05 | 0.00561 | **M** **P** **H** **D** **R** **NB** **P** **I** **I** **H** **C** **BS** **P** **E** **B** **P** **K** **PM** **P** **I** **M** **M** |
|  | LPCAT2 | 2 | [**823**](http://cpdb.molgen.mpg.de/CPDB/showSetDetails?sp=n&st=36) | [**25 (3.0%)**](http://cpdb.molgen.mpg.de/CPDB/showSetDetails?sp=n&st=36) | 1.67e-05 | 0.00561 | **H** **S** **P** **R** **E** **B** **PK** **B** **I** **M** **D** **I** **I** **H** |
|  | B3GALTL | 2 | [**2938**](http://cpdb.molgen.mpg.de/CPDB/showSetDetails?sp=n&st=37) | [**59 (2.0%)**](http://cpdb.molgen.mpg.de/CPDB/showSetDetails?sp=n&st=37) | 1.87e-05 | 0.00609 | **M** **P** **H** **D** **R** **NB** **I** **I** **H** **C** **S** **PE** **B** **P** **B** **K** **P** **PI** **M** **M** |
|  | ABCD1 | 2 | [**2026**](http://cpdb.molgen.mpg.de/CPDB/showSetDetails?sp=n&st=38) | [**45 (2.2%)**](http://cpdb.molgen.mpg.de/CPDB/showSetDetails?sp=n&st=38) | 2.16e-05 | 0.0068 | **M** **P** **H** **D** **R** **BP** **I** **I** **H** **C** **S** **P** **EB** **P** **K** **P** **M** **I** **M** |
|  | NID1 | 2 | [**1403**](http://cpdb.molgen.mpg.de/CPDB/showSetDetails?sp=n&st=39) | [**35 (2.5%)**](http://cpdb.molgen.mpg.de/CPDB/showSetDetails?sp=n&st=39) | 2.19e-05 | 0.0068 | **M** **P** **H** **D** **E** **RN** **B** **I** **I** **H** **C** **PP** **S** **B** **B** **P** **M** **IM** **M** |
|  | MMRN1(20-1228) | 2 | [**2415**](http://cpdb.molgen.mpg.de/CPDB/showSetDetails?sp=n&st=40) | [**51 (2.1%)**](http://cpdb.molgen.mpg.de/CPDB/showSetDetails?sp=n&st=40) | 2.31e-05 | 0.0069 | **M** **P** **H** **D** **R** **NB** **I** **I** **H** **C** **B** **SP** **E** **B** **P** **K** **P** **IM** **M** |
|  | HLA class I histocompatibility antigen, alpha chain F precursor | 2 | [**1412**](http://cpdb.molgen.mpg.de/CPDB/showSetDetails?sp=n&st=41) | [**35 (2.5%)**](http://cpdb.molgen.mpg.de/CPDB/showSetDetails?sp=n&st=41) | 2.36e-05 | 0.0069 | **M** **P** **H** **I** **R** **N** **BI** **I** **S** **P** **E** **B** **P** **BK** **P** **M** **D** |
|  | RIN2 | 2 | [**791**](http://cpdb.molgen.mpg.de/CPDB/showSetDetails?sp=n&st=42) | [**24 (3.0%)**](http://cpdb.molgen.mpg.de/CPDB/showSetDetails?sp=n&st=42) | 2.39e-05 | 0.0069 | **P** **H** **D** **R** **N** **B** **II** **C** **B** **S** **P** **E** **BP** **K** **P** **P** **M** |
|  | LPA | 2 | [**445**](http://cpdb.molgen.mpg.de/CPDB/showSetDetails?sp=n&st=43) | [**17 (3.8%)**](http://cpdb.molgen.mpg.de/CPDB/showSetDetails?sp=n&st=43) | 2.51e-05 | 0.00708 | **P** **H** **D** **R** **B** **I** **IC** **S** **P** **B** **P** **B** **PM** **M** **M** |
|  | RAB38 | 2 | [**1245**](http://cpdb.molgen.mpg.de/CPDB/showSetDetails?sp=n&st=44) | [**32 (2.6%)**](http://cpdb.molgen.mpg.de/CPDB/showSetDetails?sp=n&st=44) | 2.8e-05 | 0.00758 | **M** **P** **H** **R** **D** **NB** **I** **I** **H** **C** **S** **PE** **B** **P** **B** **P** **I** **M** |
|  | hPASK | 2 | [**2845**](http://cpdb.molgen.mpg.de/CPDB/showSetDetails?sp=n&st=45) | [**57 (2.0%)**](http://cpdb.molgen.mpg.de/CPDB/showSetDetails?sp=n&st=45) | 2.85e-05 | 0.00758 | **M** **P** **H** **R** **D** **NB** **P** **I** **I** **H** **C** **BS** **P** **E** **B** **P** **K** **PP** **I** **M** **M** |
|  | CDC14B | 2 | [**3819**](http://cpdb.molgen.mpg.de/CPDB/showSetDetails?sp=n&st=46) | [**71 (1.9%)**](http://cpdb.molgen.mpg.de/CPDB/showSetDetails?sp=n&st=46) | 2.87e-05 | 0.00758 | **M** **P** **H** **I** **R** **N** **BP** **I** **I** **H** **C** **B** **SP** **E** **B** **P** **P** **M** **PM** **D** **M** |
|  | RAB30 | 2 | [**1309**](http://cpdb.molgen.mpg.de/CPDB/showSetDetails?sp=n&st=47) | [**33 (2.5%)**](http://cpdb.molgen.mpg.de/CPDB/showSetDetails?sp=n&st=47) | 3.06e-05 | 0.00788 | **M** **P** **H** **R** **D** **NB** **I** **I** **H** **C** **B** **SP** **B** **P** **P** **I** **M** |
|  | RAB34 | 2 | [**1674**](http://cpdb.molgen.mpg.de/CPDB/showSetDetails?sp=n&st=48) | [**39 (2.3%)**](http://cpdb.molgen.mpg.de/CPDB/showSetDetails?sp=n&st=48) | 3.17e-05 | 0.00788 | **M** **P** **H** **R** **D** **NB** **I** **I** **H** **C** **S** **PE** **B** **P** **B** **K** **P** **IM** |
|  | q6pkc3-2 | 2 | [**3125**](http://cpdb.molgen.mpg.de/CPDB/showSetDetails?sp=n&st=49) | [**61 (2.0%)**](http://cpdb.molgen.mpg.de/CPDB/showSetDetails?sp=n&st=49) | 3.18e-05 | 0.00788 | **M** **P** **H** **I** **R** **N** **BP** **I** **I** **H** **C** **B** **SP** **E** **B** **P** **K** **P** **MP** **M** **D** **M** |
|  | RABGGTA Gene | 1 | [**103**](http://cpdb.molgen.mpg.de/CPDB/showSetDetails?sp=n&st=50) | [**8 (7.8%)**](http://cpdb.molgen.mpg.de/CPDB/showSetDetails?sp=n&st=50) | 3.18e-05 | 0.0896 | **H** **S** **R** **P** **P** **B** **I** |
|  | INPP5K | 2 | [**1558**](http://cpdb.molgen.mpg.de/CPDB/showSetDetails?sp=n&st=51) | [**37 (2.4%)**](http://cpdb.molgen.mpg.de/CPDB/showSetDetails?sp=n&st=51) | 3.52e-05 | 0.00801 | **M** **P** **H** **R** **D** **NB** **P** **I** **I** **H** **C** **BS** **P** **E** **B** **P** **K** **PM** **P** **I** **M** |
|  | MULT_57_human | 2 | [**283**](http://cpdb.molgen.mpg.de/CPDB/showSetDetails?sp=n&st=52) | [**13 (4.6%)**](http://cpdb.molgen.mpg.de/CPDB/showSetDetails?sp=n&st=52) | 3.61e-05 | 0.00801 | **M** **P** **H** **I** **R** **N** **BI** **I** **C** **S** **P** **B** **PB** **P** **M** **D** **M** |
|  | GLUD2 | 2 | [**2074**](http://cpdb.molgen.mpg.de/CPDB/showSetDetails?sp=n&st=53) | [**45 (2.2%)**](http://cpdb.molgen.mpg.de/CPDB/showSetDetails?sp=n&st=53) | 3.64e-05 | 0.00801 | **M** **P** **H** **D** **R** **B** **II** **H** **C** **P** **P** **E** **BB** **K** **P** **M** **I** **M** **SM** |
|  | RRAGB | 2 | [**1385**](http://cpdb.molgen.mpg.de/CPDB/showSetDetails?sp=n&st=54) | [**34 (2.5%)**](http://cpdb.molgen.mpg.de/CPDB/showSetDetails?sp=n&st=54) | 3.65e-05 | 0.00801 | **M** **P** **H** **D** **R** **NB** **I** **I** **H** **C** **B** **SP** **E** **B** **P** **K** **P** **MP** **I** **M** |
|  | HSD11B1 | 2 | [**1091**](http://cpdb.molgen.mpg.de/CPDB/showSetDetails?sp=n&st=55) | [**29 (2.7%)**](http://cpdb.molgen.mpg.de/CPDB/showSetDetails?sp=n&st=55) | 3.7e-05 | 0.00801 | **M** **P** **H** **D** **R** **NB** **I** **I** **H** **C** **S** **PE** **B** **P** **B** **K** **PM** **I** **M** |
|  | DUSP4 | 2 | [**3995**](http://cpdb.molgen.mpg.de/CPDB/showSetDetails?sp=n&st=56) | [**73 (1.8%)**](http://cpdb.molgen.mpg.de/CPDB/showSetDetails?sp=n&st=56) | 3.78e-05 | 0.00801 | **M** **P** **H** **I** **R** **N** **BP** **I** **I** **H** **C** **B** **SP** **E** **B** **P** **P** **M** **PM** **D** **M** |
|  | CHID1 | 2 | [**1034**](http://cpdb.molgen.mpg.de/CPDB/showSetDetails?sp=n&st=57) | [**28 (2.7%)**](http://cpdb.molgen.mpg.de/CPDB/showSetDetails?sp=n&st=57) | 3.79e-05 | 0.00801 | **H** **D** **R** **N** **B** **I** **IH** **C** **S** **P** **E** **B** **PB** **K** **P** **M** |
|  | txd16_human | 2 | [**1035**](http://cpdb.molgen.mpg.de/CPDB/showSetDetails?sp=n&st=58) | [**28 (2.7%)**](http://cpdb.molgen.mpg.de/CPDB/showSetDetails?sp=n&st=58) | 3.79e-05 | 0.00801 | **M** **P** **H** **D** **R** **NB** **I** **I** **H** **C** **B** **SP** **E** **B** **P** **K** **P** **M** |
|  | RPE | 1 | [**53**](http://cpdb.molgen.mpg.de/CPDB/showSetDetails?sp=n&st=59) | [**6 (11.3%)**](http://cpdb.molgen.mpg.de/CPDB/showSetDetails?sp=n&st=59) | 3.81e-05 | 0.0896 | **B** **S** **I** **B** **H** |
|  | DOCK6 | 2 | [**3010**](http://cpdb.molgen.mpg.de/CPDB/showSetDetails?sp=n&st=60) | [**59 (2.0%)**](http://cpdb.molgen.mpg.de/CPDB/showSetDetails?sp=n&st=60) | 3.87e-05 | 0.00801 | **M** **P** **H** **R** **D** **NB** **I** **I** **H** **C** **S** **PE** **B** **P** **B** **K** **PM** **P** **I** **M** |
|  | CDC14 cell division cycle 14 homolog C | 2 | [**3640**](http://cpdb.molgen.mpg.de/CPDB/showSetDetails?sp=n&st=61) | [**68 (1.9%)**](http://cpdb.molgen.mpg.de/CPDB/showSetDetails?sp=n&st=61) | 3.95e-05 | 0.00801 | **M** **P** **H** **I** **R** **N** **BP** **I** **I** **H** **C** **B** **SP** **E** **B** **P** **P** **M** **PM** **D** **M** |
|  | RANBP10 | 2 | [**1879**](http://cpdb.molgen.mpg.de/CPDB/showSetDetails?sp=n&st=62) | [**42 (2.2%)**](http://cpdb.molgen.mpg.de/CPDB/showSetDetails?sp=n&st=62) | 3.98e-05 | 0.00801 | **M** **P** **H** **D** **R** **NB** **I** **I** **C** **S** **P** **EB** **P** **B** **P** **M** **I** **MM** |
|  | hSSH-1L | 2 | [**4370**](http://cpdb.molgen.mpg.de/CPDB/showSetDetails?sp=n&st=63) | [**78 (1.8%)**](http://cpdb.molgen.mpg.de/CPDB/showSetDetails?sp=n&st=63) | 4.04e-05 | 0.00801 | **M** **P** **H** **D** **R** **NB** **P** **I** **I** **H** **C** **BS** **P** **E** **B** **P** **K** **PM** **P** **I** **M** **M** |
|  | TTF1 | 2 | [**2535**](http://cpdb.molgen.mpg.de/CPDB/showSetDetails?sp=n&st=64) | [**52 (2.1%)**](http://cpdb.molgen.mpg.de/CPDB/showSetDetails?sp=n&st=64) | 4.07e-05 | 0.00801 | **M** **P** **H** **D** **R** **NB** **I** **I** **H** **C** **S** **PE** **B** **P** **B** **K** **P** **PI** **M** |
|  | HAPLN3 | 2 | [**107**](http://cpdb.molgen.mpg.de/CPDB/showSetDetails?sp=n&st=65) | [**8 (7.5%)**](http://cpdb.molgen.mpg.de/CPDB/showSetDetails?sp=n&st=65) | 4.19e-05 | 0.00811 | **H** **S** **R** **B** **M** **I** |
|  | MT3 | 2 | [**209**](http://cpdb.molgen.mpg.de/CPDB/showSetDetails?sp=n&st=66) | [**11 (5.3%)**](http://cpdb.molgen.mpg.de/CPDB/showSetDetails?sp=n&st=66) | 4.27e-05 | 0.00814 | **H** **S** **P** **B** **R** **P** **PB** **M** **I** **I** |
|  | TTR | 1 | [**330**](http://cpdb.molgen.mpg.de/CPDB/showSetDetails?sp=n&st=67) | [**14 (4.2%)**](http://cpdb.molgen.mpg.de/CPDB/showSetDetails?sp=n&st=67) | 4.32e-05 | 0.0896 | **H** **B** **S** **P** **D** **R** **PP** **B** **M** **I** **I** |
|  | e7emk3_human; b7z6l9_human; b4e1j8_human; e9pbb7_human | 2 | [**1155**](http://cpdb.molgen.mpg.de/CPDB/showSetDetails?sp=n&st=68) | [**30 (2.6%)**](http://cpdb.molgen.mpg.de/CPDB/showSetDetails?sp=n&st=68) | 4.41e-05 | 0.00829 | **M** **P** **H** **I** **R** **N** **BI** **I** **H** **C** **S** **P** **BP** **K** **P** **M** **M** **D** |
|  | TRAPPC2 | 2 | [**1766**](http://cpdb.molgen.mpg.de/CPDB/showSetDetails?sp=n&st=69) | [**40 (2.3%)**](http://cpdb.molgen.mpg.de/CPDB/showSetDetails?sp=n&st=69) | 4.5e-05 | 0.00832 | **M** **P** **H** **D** **R** **NB** **I** **I** **H** **C** **S** **PE** **B** **P** **B** **K** **PM** |
|  | GLS | 2 | [**2220**](http://cpdb.molgen.mpg.de/CPDB/showSetDetails?sp=n&st=70) | [**47 (2.1%)**](http://cpdb.molgen.mpg.de/CPDB/showSetDetails?sp=n&st=70) | 4.56e-05 | 0.00832 | **M** **P** **H** **I** **R** **N** **BP** **I** **I** **H** **C** **B** **SP** **E** **B** **P** **K** **P** **MM** **D** **M** |
|  | DUSP5 | 2 | [**3659**](http://cpdb.molgen.mpg.de/CPDB/showSetDetails?sp=n&st=71) | [**68 (1.9%)**](http://cpdb.molgen.mpg.de/CPDB/showSetDetails?sp=n&st=71) | 4.65e-05 | 0.00834 | **M** **P** **H** **I** **R** **N** **BP** **I** **I** **H** **C** **B** **SP** **E** **B** **P** **P** **M** **PM** **D** **M** |
|  | SEC24D | 2 | [**1959**](http://cpdb.molgen.mpg.de/CPDB/showSetDetails?sp=n&st=72) | [**43 (2.2%)**](http://cpdb.molgen.mpg.de/CPDB/showSetDetails?sp=n&st=72) | 4.75e-05 | 0.00834 | **M** **P** **H** **I** **R** **N** **BI** **I** **H** **C** **B** **S** **PE** **B** **P** **K** **P** **P** **MD** |
|  | RAB5A | 1 | [**291**](http://cpdb.molgen.mpg.de/CPDB/showSetDetails?sp=n&st=73) | [**13 (4.5%)**](http://cpdb.molgen.mpg.de/CPDB/showSetDetails?sp=n&st=73) | 4.8e-05 | 0.0896 | **P** **C** **H** **S** **P** **D** **BN** **B** **R** **P** **P** **BM** **I** |
|  | zer1_human | 2 | [**2552**](http://cpdb.molgen.mpg.de/CPDB/showSetDetails?sp=n&st=74) | [**52 (2.0%)**](http://cpdb.molgen.mpg.de/CPDB/showSetDetails?sp=n&st=74) | 4.82e-05 | 0.00834 | **M** **P** **H** **D** **R** **NB** **I** **I** **H** **C** **S** **PE** **B** **P** **B** **K** **PM** **P** **I** **M** |
|  | 3x4Hyp-GalHyl-COL18A1(?-1754) | 2 | [**2827**](http://cpdb.molgen.mpg.de/CPDB/showSetDetails?sp=n&st=75) | [**56 (2.0%)**](http://cpdb.molgen.mpg.de/CPDB/showSetDetails?sp=n&st=75) | 4.85e-05 | 0.00834 | **M** **P** **H** **D** **R** **NB** **P** **I** **I** **C** **S** **P** **EB** **P** **B** **K** **P** **MP** **I** **M** **M** |
|  | PDGFB(82-241) | 2 | [**3669**](http://cpdb.molgen.mpg.de/CPDB/showSetDetails?sp=n&st=76) | [**68 (1.9%)**](http://cpdb.molgen.mpg.de/CPDB/showSetDetails?sp=n&st=76) | 4.95e-05 | 0.0084 | **M** **P** **H** **I** **R** **N** **BP** **I** **I** **H** **C** **B** **SP** **E** **B** **P** **K** **P** **MP** **M** **D** **M** |
|  | Low molecular weight dual specificity phosphatase 20 | 2 | [**3668**](http://cpdb.molgen.mpg.de/CPDB/showSetDetails?sp=n&st=77) | [**68 (1.9%)**](http://cpdb.molgen.mpg.de/CPDB/showSetDetails?sp=n&st=77) | 5.08e-05 | 0.00851 | **M** **P** **H** **I** **R** **N** **BP** **I** **I** **H** **C** **B** **SP** **E** **B** **P** **P** **M** **PM** **D** **M** |
|  | RAB3B | 2 | [**1344**](http://cpdb.molgen.mpg.de/CPDB/showSetDetails?sp=n&st=78) | [**33 (2.5%)**](http://cpdb.molgen.mpg.de/CPDB/showSetDetails?sp=n&st=78) | 5.15e-05 | 0.00851 | **M** **P** **H** **I** **R** **N** **BI** **I** **H** **C** **S** **P** **EB** **P** **B** **K** **P** **MD** |
|  | S100A10 | 2 | [**2638**](http://cpdb.molgen.mpg.de/CPDB/showSetDetails?sp=n&st=79) | [**53 (2.0%)**](http://cpdb.molgen.mpg.de/CPDB/showSetDetails?sp=n&st=79) | 5.34e-05 | 0.00868 | **M** **P** **H** **R** **D** **NB** **I** **I** **H** **C** **B** **SP** **E** **B** **P** **K** **P** **MP** **I** **M** **M** |
|  | 3D-PROC(33-197) | 2 | [**1411**](http://cpdb.molgen.mpg.de/CPDB/showSetDetails?sp=n&st=80) | [**34 (2.4%)**](http://cpdb.molgen.mpg.de/CPDB/showSetDetails?sp=n&st=80) | 5.39e-05 | 0.00868 | **M** **P** **H** **I** **R** **N** **BI** **I** **H** **C** **S** **P** **BP** **B** **K** **P** **M** **MD** **M** |
|  | UGGT2 | 2 | [**2709**](http://cpdb.molgen.mpg.de/CPDB/showSetDetails?sp=n&st=81) | [**54 (2.0%)**](http://cpdb.molgen.mpg.de/CPDB/showSetDetails?sp=n&st=81) | 5.47e-05 | 0.0087 | **M** **P** **H** **I** **R** **N** **BP** **I** **I** **H** **C** **B** **SP** **E** **B** **P** **K** **P** **MP** **M** **D** **M** |
|  | CD9 | 2 | [**2569**](http://cpdb.molgen.mpg.de/CPDB/showSetDetails?sp=n&st=82) | [**52 (2.0%)**](http://cpdb.molgen.mpg.de/CPDB/showSetDetails?sp=n&st=82) | 5.7e-05 | 0.00888 | **M** **P** **H** **D** **R** **NB** **P** **I** **I** **H** **C** **BS** **P** **E** **B** **P** **K** **PM** **I** **M** **M** |
|  | CoCoA | 2 | [**889**](http://cpdb.molgen.mpg.de/CPDB/showSetDetails?sp=n&st=83) | [**25 (2.8%)**](http://cpdb.molgen.mpg.de/CPDB/showSetDetails?sp=n&st=83) | 5.73e-05 | 0.00888 | **M** **P** **H** **D** **R** **NB** **I** **I** **C** **S** **P** **EB** **P** **B** **P** **M** |
|  | DUSP2 | 2 | [**3691**](http://cpdb.molgen.mpg.de/CPDB/showSetDetails?sp=n&st=84) | [**68 (1.8%)**](http://cpdb.molgen.mpg.de/CPDB/showSetDetails?sp=n&st=84) | 6.22e-05 | 0.00952 | **M** **P** **H** **I** **R** **N** **BP** **I** **I** **H** **C** **B** **SP** **E** **B** **P** **P** **M** **PM** **D** **M** |
|  | ZACN | 2 | [**1240**](http://cpdb.molgen.mpg.de/CPDB/showSetDetails?sp=n&st=85) | [**31 (2.5%)**](http://cpdb.molgen.mpg.de/CPDB/showSetDetails?sp=n&st=85) | 6.47e-05 | 0.00952 | **M** **P** **H** **R** **D** **NB** **I** **I** **H** **C** **S** **PE** **B** **P** **B** **K** **PM** **M** **M** |
|  | gl8d1_human | 2 | [**219**](http://cpdb.molgen.mpg.de/CPDB/showSetDetails?sp=n&st=86) | [**11 (5.0%)**](http://cpdb.molgen.mpg.de/CPDB/showSetDetails?sp=n&st=86) | 6.52e-05 | 0.00952 | **C** **H** **S** **B** **R** **N** **PB** **M** **I** **I** |
|  | RBP4(19-201) | 2 | [**219**](http://cpdb.molgen.mpg.de/CPDB/showSetDetails?sp=n&st=87) | [**11 (5.0%)**](http://cpdb.molgen.mpg.de/CPDB/showSetDetails?sp=n&st=87) | 6.52e-05 | 0.00952 | **H** **S** **P** **B** **E** **R** **PK** **P** **B** **M** **I** **I** |
|  | ENTPD6 (77-484) | 2 | [**1988**](http://cpdb.molgen.mpg.de/CPDB/showSetDetails?sp=n&st=88) | [**43 (2.2%)**](http://cpdb.molgen.mpg.de/CPDB/showSetDetails?sp=n&st=88) | 6.54e-05 | 0.00952 | **M** **P** **H** **D** **R** **NB** **P** **I** **I** **H** **C** **BS** **P** **E** **B** **P** **K** **PM** **P** **M** **M** |
|  | (RefSeq) fragile histidine triad | 1 | [**19**](http://cpdb.molgen.mpg.de/CPDB/showSetDetails?sp=n&st=89) | [**4 (21.1%)**](http://cpdb.molgen.mpg.de/CPDB/showSetDetails?sp=n&st=89) | 6.56e-05 | 0.0904 | **P** **H** **S** **E** **D** **K** **PB** **I** **H** |
|  | tm192_human | 2 | [**1480**](http://cpdb.molgen.mpg.de/CPDB/showSetDetails?sp=n&st=90) | [**35 (2.4%)**](http://cpdb.molgen.mpg.de/CPDB/showSetDetails?sp=n&st=90) | 6.68e-05 | 0.00952 | **M** **P** **H** **I** **R** **N** **BI** **I** **H** **C** **S** **P** **EB** **P** **K** **P** **M** **MD** |
|  | NDK | 2 | [**1490**](http://cpdb.molgen.mpg.de/CPDB/showSetDetails?sp=n&st=91) | [**35 (2.4%)**](http://cpdb.molgen.mpg.de/CPDB/showSetDetails?sp=n&st=91) | 6.68e-05 | 0.00952 | **M** **P** **H** **R** **D** **NB** **I** **I** **C** **S** **P** **EB** **P** **B** **K** **P** **MM** **M** |
|  | q96aq3_human | 2 | [**484**](http://cpdb.molgen.mpg.de/CPDB/showSetDetails?sp=n&st=92) | [**17 (3.5%)**](http://cpdb.molgen.mpg.de/CPDB/showSetDetails?sp=n&st=92) | 6.79e-05 | 0.00957 | **C** **H** **S** **P** **R** **B** **PP** **B** **M** **I** **I** |
|  | e7eta6_human | 2 | [**2794**](http://cpdb.molgen.mpg.de/CPDB/showSetDetails?sp=n&st=93) | [**55 (2.0%)**](http://cpdb.molgen.mpg.de/CPDB/showSetDetails?sp=n&st=93) | 7.02e-05 | 0.00965 | **M** **P** **H** **D** **R** **NB** **P** **I** **I** **H** **C** **SP** **E** **B** **P** **B** **K** **PP** **I** **M** |
|  | RAB22A | 2 | [**1865**](http://cpdb.molgen.mpg.de/CPDB/showSetDetails?sp=n&st=94) | [**41 (2.2%)**](http://cpdb.molgen.mpg.de/CPDB/showSetDetails?sp=n&st=94) | 7.19e-05 | 0.00965 | **M** **P** **H** **R** **D** **NB** **I** **I** **H** **C** **B** **SP** **B** **P** **K** **P** **M** **IM** |
|  | h0yh87_human | 2 | [**1615**](http://cpdb.molgen.mpg.de/CPDB/showSetDetails?sp=n&st=95) | [**37 (2.3%)**](http://cpdb.molgen.mpg.de/CPDB/showSetDetails?sp=n&st=95) | 7.23e-05 | 0.00965 | **P** **H** **D** **R** **N** **B** **II** **C** **B** **S** **P** **E** **BP** **K** **P** **P** **M** |
|  | RAB40B | 2 | [**1017**](http://cpdb.molgen.mpg.de/CPDB/showSetDetails?sp=n&st=96) | [**27 (2.7%)**](http://cpdb.molgen.mpg.de/CPDB/showSetDetails?sp=n&st=96) | 7.24e-05 | 0.00965 | **M** **P** **H** **R** **D** **NB** **I** **I** **H** **C** **S** **PE** **B** **P** **B** **K** **PM** |
|  | fkb15_human | 2 | [**3152**](http://cpdb.molgen.mpg.de/CPDB/showSetDetails?sp=n&st=97) | [**60 (1.9%)**](http://cpdb.molgen.mpg.de/CPDB/showSetDetails?sp=n&st=97) | 7.26e-05 | 0.00965 | **M** **P** **H** **D** **R** **NB** **I** **I** **H** **C** **S** **PE** **B** **P** **B** **K** **PM** **P** **I** **M** |
|  | SELP | 1 | [**37**](http://cpdb.molgen.mpg.de/CPDB/showSetDetails?sp=n&st=98) | [**5 (13.5%)**](http://cpdb.molgen.mpg.de/CPDB/showSetDetails?sp=n&st=98) | 7.27e-05 | 0.0904 | **H** **S** **P** **D** **R** **P** **BI** **I** **M** |
|  | FBXL2 | 2 | [**1370**](http://cpdb.molgen.mpg.de/CPDB/showSetDetails?sp=n&st=99) | [**33 (2.4%)**](http://cpdb.molgen.mpg.de/CPDB/showSetDetails?sp=n&st=99) | 7.37e-05 | 0.00965 | **M** **P** **H** **R** **D** **NB** **I** **I** **C** **S** **P** **BP** **B** **P** **P** **M** |
|  | CENPC | 2 | [**2937**](http://cpdb.molgen.mpg.de/CPDB/showSetDetails?sp=n&st=100) | [**57 (1.9%)**](http://cpdb.molgen.mpg.de/CPDB/showSetDetails?sp=n&st=100) | 7.56e-05 | 0.00965 | **M** **P** **H** **D** **R** **NB** **P** **I** **I** **H** **C** **BS** **P** **E** **B** **P** **K** **PM** **P** **I** **M** |
|  | AKR7A2 | 2 | [**4090**](http://cpdb.molgen.mpg.de/CPDB/showSetDetails?sp=n&st=101) | [**73 (1.8%)**](http://cpdb.molgen.mpg.de/CPDB/showSetDetails?sp=n&st=101) | 7.63e-05 | 0.00965 | **M** **P** **H** **R** **D** **NB** **I** **I** **H** **C** **B** **SP** **E** **B** **P** **K** **P** **MP** **I** **M** |
|  | MIRab13 | 2 | [**2739**](http://cpdb.molgen.mpg.de/CPDB/showSetDetails?sp=n&st=102) | [**54 (2.0%)**](http://cpdb.molgen.mpg.de/CPDB/showSetDetails?sp=n&st=102) | 7.7e-05 | 0.00965 | **M** **P** **H** **R** **D** **NB** **I** **I** **H** **C** **S** **PE** **B** **P** **B** **K** **P** **PI** **M** **M** |
|  | NLI-IF | 2 | [**3074**](http://cpdb.molgen.mpg.de/CPDB/showSetDetails?sp=n&st=103) | [**59 (1.9%)**](http://cpdb.molgen.mpg.de/CPDB/showSetDetails?sp=n&st=103) | 7.73e-05 | 0.00965 | **M** **P** **H** **I** **R** **N** **BP** **I** **I** **H** **C** **B** **SP** **E** **B** **P** **P** **M** **PM** **D** |
|  | glycosylasparaginase alpha chain | 2 | [**150**](http://cpdb.molgen.mpg.de/CPDB/showSetDetails?sp=n&st=104) | [**9 (6.0%)**](http://cpdb.molgen.mpg.de/CPDB/showSetDetails?sp=n&st=104) | 7.87e-05 | 0.00965 | **B** **M** **I** **E** **R** **H** **H** |
|  | SUPT4H1 | 2 | [**1436**](http://cpdb.molgen.mpg.de/CPDB/showSetDetails?sp=n&st=105) | [**34 (2.4%)**](http://cpdb.molgen.mpg.de/CPDB/showSetDetails?sp=n&st=105) | 7.96e-05 | 0.00965 | **P** **H** **D** **R** **N** **B** **II** **C** **B** **S** **P** **E** **BP** **P** **P** **M** **M** |
|  | tnpo3_human | 2 | [**3506**](http://cpdb.molgen.mpg.de/CPDB/showSetDetails?sp=n&st=106) | [**65 (1.9%)**](http://cpdb.molgen.mpg.de/CPDB/showSetDetails?sp=n&st=106) | 7.97e-05 | 0.00965 | **M** **P** **H** **D** **R** **NB** **P** **I** **I** **H** **C** **BS** **P** **E** **B** **P** **K** **PM** **P** **I** **M** |
|  | DAB1 | 2 | [**2537**](http://cpdb.molgen.mpg.de/CPDB/showSetDetails?sp=n&st=107) | [**51 (2.0%)**](http://cpdb.molgen.mpg.de/CPDB/showSetDetails?sp=n&st=107) | 8.04e-05 | 0.00965 | **M** **P** **H** **D** **R** **NB** **P** **I** **I** **H** **C** **BS** **P** **E** **B** **P** **K** **PM** **I** **M** **M** |
|  | ARL13B | 2 | [**1026**](http://cpdb.molgen.mpg.de/CPDB/showSetDetails?sp=n&st=108) | [**27 (2.6%)**](http://cpdb.molgen.mpg.de/CPDB/showSetDetails?sp=n&st=108) | 8.26e-05 | 0.00965 | **P** **H** **R** **D** **N** **B** **II** **H** **S** **P** **E** **B** **PB** **K** **P** **I** **M** |
|  | HLA class II histocompatibility antigen, DP alpha chain precursor | 2 | [**2951**](http://cpdb.molgen.mpg.de/CPDB/showSetDetails?sp=n&st=109) | [**57 (1.9%)**](http://cpdb.molgen.mpg.de/CPDB/showSetDetails?sp=n&st=109) | 8.31e-05 | 0.00965 | **M** **P** **H** **D** **R** **NB** **P** **I** **I** **H** **C** **BS** **P** **E** **B** **P** **K** **PM** **P** **I** **M** **M** |
|  | KIF21B | 2 | [**2139**](http://cpdb.molgen.mpg.de/CPDB/showSetDetails?sp=n&st=110) | [**45 (2.1%)**](http://cpdb.molgen.mpg.de/CPDB/showSetDetails?sp=n&st=110) | 8.33e-05 | 0.00965 | **M** **P** **H** **R** **D** **NB** **I** **I** **H** **C** **B** **SP** **E** **B** **P** **K** **P** **PI** **M** |
|  | ANXA5 | 2 | [**2412**](http://cpdb.molgen.mpg.de/CPDB/showSetDetails?sp=n&st=111) | [**49 (2.0%)**](http://cpdb.molgen.mpg.de/CPDB/showSetDetails?sp=n&st=111) | 8.39e-05 | 0.00965 | **M** **P** **H** **I** **R** **N** **BI** **I** **H** **C** **B** **S** **PE** **B** **P** **K** **P** **M** **PM** **D** **M** |
|  | CTDP1 | 2 | [**4390**](http://cpdb.molgen.mpg.de/CPDB/showSetDetails?sp=n&st=112) | [**77 (1.8%)**](http://cpdb.molgen.mpg.de/CPDB/showSetDetails?sp=n&st=112) | 8.4e-05 | 0.00965 | **M** **P** **H** **D** **R** **NB** **P** **I** **I** **H** **C** **BS** **P** **E** **B** **P** **K** **PM** **P** **I** **M** |
|  | TGFBR2 R528C | 2 | [**3090**](http://cpdb.molgen.mpg.de/CPDB/showSetDetails?sp=n&st=113) | [**59 (1.9%)**](http://cpdb.molgen.mpg.de/CPDB/showSetDetails?sp=n&st=113) | 8.4e-05 | 0.00965 | **M** **P** **H** **I** **R** **N** **BP** **I** **I** **H** **C** **B** **SP** **E** **B** **P** **P** **M** **PM** **D** **M** |
|  | a2abh1_human | 2 | [**2010**](http://cpdb.molgen.mpg.de/CPDB/showSetDetails?sp=n&st=114) | [**43 (2.1%)**](http://cpdb.molgen.mpg.de/CPDB/showSetDetails?sp=n&st=114) | 8.63e-05 | 0.00981 | **H** **I** **R** **N** **B** **I** **I** **HC** **B** **S** **P** **E** **B** **PK** **P** **P** **M** **D** |
|  | PHKG2 | 2 | [**750**](http://cpdb.molgen.mpg.de/CPDB/showSetDetails?sp=n&st=115) | [**22 (2.9%)**](http://cpdb.molgen.mpg.de/CPDB/showSetDetails?sp=n&st=115) | 8.7e-05 | 0.00981 | **P** **H** **D** **R** **N** **B** **IH** **C** **S** **P** **E** **B** **PB** **K** **P** **M** **M** |
|  | CDCA2 | 2 | [**2611**](http://cpdb.molgen.mpg.de/CPDB/showSetDetails?sp=n&st=116) | [**52 (2.0%)**](http://cpdb.molgen.mpg.de/CPDB/showSetDetails?sp=n&st=116) | 8.84e-05 | 0.00988 | **M** **P** **H** **R** **D** **NB** **I** **I** **H** **C** **S** **PE** **B** **P** **B** **K** **P** **PI** **M** |
|  | RABAC1 | 2 | [**2683**](http://cpdb.molgen.mpg.de/CPDB/showSetDetails?sp=n&st=117) | [**53 (2.0%)**](http://cpdb.molgen.mpg.de/CPDB/showSetDetails?sp=n&st=117) | 9.12e-05 | 0.0101 | **M** **P** **H** **I** **R** **N** **BP** **I** **I** **C** **B** **S** **P** **EB** **P** **K** **P** **M** **MD** **M** |
|  | DUSP10 | 2 | [**3884**](http://cpdb.molgen.mpg.de/CPDB/showSetDetails?sp=n&st=118) | [**70 (1.8%)**](http://cpdb.molgen.mpg.de/CPDB/showSetDetails?sp=n&st=118) | 9.27e-05 | 0.0102 | **M** **P** **H** **I** **R** **N** **BP** **I** **I** **H** **C** **B** **SP** **E** **B** **P** **P** **M** **PM** **D** **M** |
|  | DUSP8 | 2 | [**3956**](http://cpdb.molgen.mpg.de/CPDB/showSetDetails?sp=n&st=119) | [**71 (1.8%)**](http://cpdb.molgen.mpg.de/CPDB/showSetDetails?sp=n&st=119) | 9.37e-05 | 0.0102 | **M** **P** **H** **I** **R** **N** **BP** **I** **I** **H** **C** **B** **SP** **E** **B** **P** **P** **M** **PM** **D** **M** |
|  | fakd2_human | 2 | [**2217**](http://cpdb.molgen.mpg.de/CPDB/showSetDetails?sp=n&st=120) | [**46 (2.1%)**](http://cpdb.molgen.mpg.de/CPDB/showSetDetails?sp=n&st=120) | 9.53e-05 | 0.0103 | **P** **H** **I** **R** **N** **B** **I** **IH** **C** **S** **P** **E** **B** **PB** **K** **P** **P** **M** **D** |
|  | bin3_human | 2 | [**2553**](http://cpdb.molgen.mpg.de/CPDB/showSetDetails?sp=n&st=121) | [**51 (2.0%)**](http://cpdb.molgen.mpg.de/CPDB/showSetDetails?sp=n&st=121) | 9.65e-05 | 0.0103 | **M** **P** **H** **I** **R** **N** **BP** **I** **I** **H** **C** **S** **P** **EB** **P** **B** **K** **P** **MP** **M** **D** **M** |
|  | PRL-1 | 2 | [**2421**](http://cpdb.molgen.mpg.de/CPDB/showSetDetails?sp=n&st=122) | [**49 (2.0%)**](http://cpdb.molgen.mpg.de/CPDB/showSetDetails?sp=n&st=122) | 9.8e-05 | 0.0104 | **M** **P** **H** **D** **R** **NB** **P** **I** **I** **C** **B** **SP** **E** **B** **P** **K** **P** **MI** **M** **M** |
|  | KIF3B | 2 | [**2553**](http://cpdb.molgen.mpg.de/CPDB/showSetDetails?sp=n&st=123) | [**51 (2.0%)**](http://cpdb.molgen.mpg.de/CPDB/showSetDetails?sp=n&st=123) | 9.95e-05 | 0.0104 | **M** **P** **H** **R** **D** **NB** **P** **I** **I** **H** **C** **BS** **P** **E** **B** **P** **K** **PP** **I** **M** **M** |
|  | KCNN3 | 2 | [**155**](http://cpdb.molgen.mpg.de/CPDB/showSetDetails?sp=n&st=124) | [**9 (5.8%)**](http://cpdb.molgen.mpg.de/CPDB/showSetDetails?sp=n&st=124) | 0.000101 | 0.0104 | **P** **C** **H** **S** **P** **R** **BN** **B** **P** **P** **B** **M** **I** |
|  | CDC25C Gene | 2 | [**4115**](http://cpdb.molgen.mpg.de/CPDB/showSetDetails?sp=n&st=125) | [**73 (1.8%)**](http://cpdb.molgen.mpg.de/CPDB/showSetDetails?sp=n&st=125) | 0.000102 | 0.0104 | **M** **P** **H** **I** **R** **N** **BP** **I** **I** **H** **C** **B** **SP** **E** **B** **P** **K** **P** **MP** **M** **D** **M** |
|  | TYRP1 | 2 | [**271**](http://cpdb.molgen.mpg.de/CPDB/showSetDetails?sp=n&st=126) | [**12 (4.4%)**](http://cpdb.molgen.mpg.de/CPDB/showSetDetails?sp=n&st=126) | 0.000102 | 0.0104 | **P** **H** **I** **R** **N** **B** **PI** **I** **H** **C** **S** **P** **EB** **P** **B** **K** **P** **MD** |
|  | NRAS | 2 | [**4650**](http://cpdb.molgen.mpg.de/CPDB/showSetDetails?sp=n&st=127) | [**80 (1.7%)**](http://cpdb.molgen.mpg.de/CPDB/showSetDetails?sp=n&st=127) | 0.000104 | 0.0104 | **M** **P** **H** **D** **R** **NB** **P** **I** **I** **H** **C** **BS** **P** **E** **B** **P** **K** **PM** **P** **I** **M** **M** |
|  | NPC1 | 2 | [**708**](http://cpdb.molgen.mpg.de/CPDB/showSetDetails?sp=n&st=128) | [**21 (3.0%)**](http://cpdb.molgen.mpg.de/CPDB/showSetDetails?sp=n&st=128) | 0.000104 | 0.0104 | **P** **H** **D** **R** **B** **I** **IH** **C** **S** **P** **E** **B** **PK** **P** **M** **M** |
|  | CDKN2C | 2 | [**2494**](http://cpdb.molgen.mpg.de/CPDB/showSetDetails?sp=n&st=129) | [**50 (2.0%)**](http://cpdb.molgen.mpg.de/CPDB/showSetDetails?sp=n&st=129) | 0.000104 | 0.0104 | **M** **P** **H** **R** **D** **NB** **I** **I** **H** **C** **B** **SP** **E** **B** **P** **K** **P** **MP** **I** **M** **M** |
|  | CYP2C9 | 2 | [**1837**](http://cpdb.molgen.mpg.de/CPDB/showSetDetails?sp=n&st=130) | [**40 (2.2%)**](http://cpdb.molgen.mpg.de/CPDB/showSetDetails?sp=n&st=130) | 0.000105 | 0.0104 | **M** **P** **H** **R** **D** **NB** **C** **I** **I** **H** **D** **BS** **P** **E** **B** **P** **K** **PM** **P** **I** **M** |
|  | NHLRC2 | 2 | [**1769**](http://cpdb.molgen.mpg.de/CPDB/showSetDetails?sp=n&st=131) | [**39 (2.2%)**](http://cpdb.molgen.mpg.de/CPDB/showSetDetails?sp=n&st=131) | 0.000106 | 0.0104 | **M** **P** **H** **D** **R** **NB** **I** **I** **H** **C** **B** **SP** **B** **P** **P** **I** **M** **M** |
|  | RAB40C | 2 | [**1277**](http://cpdb.molgen.mpg.de/CPDB/showSetDetails?sp=n&st=132) | [**31 (2.4%)**](http://cpdb.molgen.mpg.de/CPDB/showSetDetails?sp=n&st=132) | 0.000106 | 0.0104 | **M** **P** **H** **R** **D** **NB** **I** **I** **C** **S** **P** **BP** **B** **P** **M** |
|  | ENPP4 | 2 | [**1463**](http://cpdb.molgen.mpg.de/CPDB/showSetDetails?sp=n&st=133) | [**34 (2.3%)**](http://cpdb.molgen.mpg.de/CPDB/showSetDetails?sp=n&st=133) | 0.000108 | 0.0105 | **M** **P** **H** **R** **D** **NB** **I** **I** **H** **C** **S** **PE** **B** **P** **B** **K** **P** **IM** |
|  | LMAN1L | 2 | [**926**](http://cpdb.molgen.mpg.de/CPDB/showSetDetails?sp=n&st=134) | [**25 (2.7%)**](http://cpdb.molgen.mpg.de/CPDB/showSetDetails?sp=n&st=134) | 0.00011 | 0.0105 | **M** **P** **H** **D** **R** **NB** **I** **I** **H** **C** **B** **SP** **B** **P** **P** **M** |
|  | MATK | 2 | [**988**](http://cpdb.molgen.mpg.de/CPDB/showSetDetails?sp=n&st=135) | [**26 (2.7%)**](http://cpdb.molgen.mpg.de/CPDB/showSetDetails?sp=n&st=135) | 0.000111 | 0.0105 | **M** **P** **H** **I** **R** **N** **BP** **I** **I** **C** **S** **P** **E** **BP** **B** **P** **M** **P** **MD** **M** |
|  | ccdc9_human | 2 | [**2915**](http://cpdb.molgen.mpg.de/CPDB/showSetDetails?sp=n&st=136) | [**56 (1.9%)**](http://cpdb.molgen.mpg.de/CPDB/showSetDetails?sp=n&st=136) | 0.000112 | 0.0106 | **M** **P** **H** **R** **D** **NB** **I** **I** **H** **C** **S** **PE** **B** **P** **B** **K** **P** **PI** **M** **M** |
|  | RAB8B | 1 | [**123**](http://cpdb.molgen.mpg.de/CPDB/showSetDetails?sp=n&st=137) | [**8 (6.5%)**](http://cpdb.molgen.mpg.de/CPDB/showSetDetails?sp=n&st=137) | 0.000112 | 0.0945 | **B** **B** **S** **R** **I** **H** |
|  | FBXO5 | 2 | [**2432**](http://cpdb.molgen.mpg.de/CPDB/showSetDetails?sp=n&st=138) | [**49 (2.0%)**](http://cpdb.molgen.mpg.de/CPDB/showSetDetails?sp=n&st=138) | 0.000114 | 0.0107 | **M** **P** **H** **D** **R** **NB** **P** **I** **I** **C** **S** **P** **EB** **P** **B** **K** **P** **MP** **M** |
|  | TBC1D3 | 2 | [**158**](http://cpdb.molgen.mpg.de/CPDB/showSetDetails?sp=n&st=139) | [**9 (5.7%)**](http://cpdb.molgen.mpg.de/CPDB/showSetDetails?sp=n&st=139) | 0.000117 | 0.0108 | **P** **C** **H** **S** **P** **R** **BN** **B** **P** **P** **B** **M** **I** |
|  | CD58 | 2 | [**2510**](http://cpdb.molgen.mpg.de/CPDB/showSetDetails?sp=n&st=140) | [**50 (2.0%)**](http://cpdb.molgen.mpg.de/CPDB/showSetDetails?sp=n&st=140) | 0.000117 | 0.0108 | **M** **P** **H** **I** **R** **N** **BI** **I** **H** **C** **S** **P** **EB** **P** **B** **K** **P** **MP** **M** **D** **M** |
|  | COL4A2(184-1712) | 2 | [**2440**](http://cpdb.molgen.mpg.de/CPDB/showSetDetails?sp=n&st=141) | [**49 (2.0%)**](http://cpdb.molgen.mpg.de/CPDB/showSetDetails?sp=n&st=141) | 0.000118 | 0.0108 | **M** **P** **H** **D** **R** **NB** **I** **I** **H** **C** **S** **PE** **B** **P** **B** **K** **PM** **P** **I** **M** **M** |
|  | tbc30_human | 2 | [**196**](http://cpdb.molgen.mpg.de/CPDB/showSetDetails?sp=n&st=142) | [**10 (5.1%)**](http://cpdb.molgen.mpg.de/CPDB/showSetDetails?sp=n&st=142) | 0.000118 | 0.0108 | **P** **C** **B** **S** **R** **I** **H** |
|  | ITGA3(33-1051) | 2 | [**2176**](http://cpdb.molgen.mpg.de/CPDB/showSetDetails?sp=n&st=143) | [**45 (2.1%)**](http://cpdb.molgen.mpg.de/CPDB/showSetDetails?sp=n&st=143) | 0.000125 | 0.0112 | **M** **P** **H** **D** **R** **NB** **P** **I** **I** **H** **C** **BS** **P** **E** **B** **P** **P** **MI** **M** **M** |
|  | JNK-stimulatory phosphatase-1 | 2 | [**3773**](http://cpdb.molgen.mpg.de/CPDB/showSetDetails?sp=n&st=144) | [**68 (1.8%)**](http://cpdb.molgen.mpg.de/CPDB/showSetDetails?sp=n&st=144) | 0.000125 | 0.0112 | **M** **P** **H** **I** **R** **N** **BP** **I** **I** **H** **C** **B** **SP** **E** **B** **P** **P** **M** **PM** **D** **M** |
|  | LRPAP1 | 2 | [**2308**](http://cpdb.molgen.mpg.de/CPDB/showSetDetails?sp=n&st=145) | [**47 (2.0%)**](http://cpdb.molgen.mpg.de/CPDB/showSetDetails?sp=n&st=145) | 0.000125 | 0.0112 | **M** **P** **H** **D** **R** **NB** **I** **I** **H** **C** **S** **PE** **B** **P** **B** **K** **PM** **P** **I** **M** |
|  | ESYT1 | 1 | [**822**](http://cpdb.molgen.mpg.de/CPDB/showSetDetails?sp=n&st=146) | [**23 (2.8%)**](http://cpdb.molgen.mpg.de/CPDB/showSetDetails?sp=n&st=146) | 0.000126 | 0.0945 | **R** **B** **S** **M** **I** **D** |
|  | EPM2A | 2 | [**3852**](http://cpdb.molgen.mpg.de/CPDB/showSetDetails?sp=n&st=147) | [**69 (1.8%)**](http://cpdb.molgen.mpg.de/CPDB/showSetDetails?sp=n&st=147) | 0.000132 | 0.0116 | **M** **P** **H** **I** **R** **N** **BP** **I** **I** **H** **C** **B** **SP** **E** **B** **P** **K** **P** **MP** **M** **D** **M** |
|  | tisd_human | 2 | [**2725**](http://cpdb.molgen.mpg.de/CPDB/showSetDetails?sp=n&st=148) | [**53 (2.0%)**](http://cpdb.molgen.mpg.de/CPDB/showSetDetails?sp=n&st=148) | 0.000132 | 0.0116 | **M** **P** **H** **I** **R** **N** **BP** **I** **I** **H** **C** **B** **SP** **E** **B** **P** **K** **P** **PM** **D** |
|  | DEFA3(20-94) | 2 | [**322**](http://cpdb.molgen.mpg.de/CPDB/showSetDetails?sp=n&st=149) | [**13 (4.0%)**](http://cpdb.molgen.mpg.de/CPDB/showSetDetails?sp=n&st=149) | 0.000133 | 0.0116 | **M** **P** **H** **I** **R** **N** **BI** **I** **C** **B** **S** **P** **BP** **P** **M** **D** |
|  | TFB2M | 2 | [**2186**](http://cpdb.molgen.mpg.de/CPDB/showSetDetails?sp=n&st=150) | [**45 (2.1%)**](http://cpdb.molgen.mpg.de/CPDB/showSetDetails?sp=n&st=150) | 0.000134 | 0.0117 | **P** **H** **R** **D** **N** **B** **II** **H** **C** **S** **P** **E** **BP** **B** **K** **P** **P** **M** |
|  | RAB7A | 1 | [**1600**](http://cpdb.molgen.mpg.de/CPDB/showSetDetails?sp=n&st=151) | [**36 (2.3%)**](http://cpdb.molgen.mpg.de/CPDB/showSetDetails?sp=n&st=151) | 0.000135 | 0.0945 | **H** **S** **P** **D** **R** **P** **BM** **I** **I** |
|  | RAB11B | 1 | [**999**](http://cpdb.molgen.mpg.de/CPDB/showSetDetails?sp=n&st=152) | [**26 (2.6%)**](http://cpdb.molgen.mpg.de/CPDB/showSetDetails?sp=n&st=152) | 0.000137 | 0.0945 | **B** **S** **R** **I** **D** **H** |
|  | CD74 | 2 | [**1056**](http://cpdb.molgen.mpg.de/CPDB/showSetDetails?sp=n&st=153) | [**27 (2.6%)**](http://cpdb.molgen.mpg.de/CPDB/showSetDetails?sp=n&st=153) | 0.000138 | 0.0118 | **M** **P** **H** **R** **D** **NB** **I** **I** **H** **C** **B** **SP** **B** **P** **P** **I** **M** **M** |
|  | CLPTM1 | 1 | [**95**](http://cpdb.molgen.mpg.de/CPDB/showSetDetails?sp=n&st=154) | [**7 (7.4%)**](http://cpdb.molgen.mpg.de/CPDB/showSetDetails?sp=n&st=154) | 0.000139 | 0.0945 | **I** **B** |
|  | hxc13_human | 2 | [**3286**](http://cpdb.molgen.mpg.de/CPDB/showSetDetails?sp=n&st=155) | [**61 (1.9%)**](http://cpdb.molgen.mpg.de/CPDB/showSetDetails?sp=n&st=155) | 0.00014 | 0.012 | **M** **P** **H** **R** **D** **NB** **I** **I** **H** **C** **B** **SP** **E** **B** **P** **K** **P** **MP** **I** **M** |
|  | DBI | 2 | [**1865**](http://cpdb.molgen.mpg.de/CPDB/showSetDetails?sp=n&st=156) | [**40 (2.2%)**](http://cpdb.molgen.mpg.de/CPDB/showSetDetails?sp=n&st=156) | 0.000142 | 0.0121 | **P** **H** **R** **D** **N** **B** **II** **H** **S** **P** **E** **B** **PB** **K** **P** **M** **I** **M** |
|  | j3qt39_human | 2 | [**1356**](http://cpdb.molgen.mpg.de/CPDB/showSetDetails?sp=n&st=157) | [**32 (2.4%)**](http://cpdb.molgen.mpg.de/CPDB/showSetDetails?sp=n&st=157) | 0.000144 | 0.0121 | **P** **H** **I** **D** **N** **B** **I** **IH** **C** **B** **S** **E** **B** **RP** **K** **P** **P** **M** |
|  | BRMS1L | 2 | [**3088**](http://cpdb.molgen.mpg.de/CPDB/showSetDetails?sp=n&st=158) | [**58 (1.9%)**](http://cpdb.molgen.mpg.de/CPDB/showSetDetails?sp=n&st=158) | 0.000152 | 0.0126 | **M** **P** **H** **R** **D** **NB** **I** **I** **H** **C** **S** **PE** **B** **P** **B** **K** **PM** **P** **I** **M** |
|  | GAS6(31-691) | 2 | [**2062**](http://cpdb.molgen.mpg.de/CPDB/showSetDetails?sp=n&st=159) | [**43 (2.1%)**](http://cpdb.molgen.mpg.de/CPDB/showSetDetails?sp=n&st=159) | 0.000152 | 0.0126 | **M** **P** **H** **D** **R** **NB** **I** **I** **H** **C** **B** **SP** **E** **B** **P** **K** **P** **MI** **M** **M** |
|  | Low molecular weight dual specificity phosphatase 21 | 2 | [**4019**](http://cpdb.molgen.mpg.de/CPDB/showSetDetails?sp=n&st=160) | [**71 (1.8%)**](http://cpdb.molgen.mpg.de/CPDB/showSetDetails?sp=n&st=160) | 0.000152 | 0.0126 | **M** **P** **H** **I** **R** **N** **BP** **I** **I** **H** **C** **B** **SP** **E** **B** **P** **P** **M** **PM** **D** **M** |
|  | PDGFA-2 | 2 | [**3228**](http://cpdb.molgen.mpg.de/CPDB/showSetDetails?sp=n&st=161) | [**60 (1.9%)**](http://cpdb.molgen.mpg.de/CPDB/showSetDetails?sp=n&st=161) | 0.000154 | 0.0126 | **M** **P** **H** **I** **R** **N** **BP** **I** **I** **H** **C** **B** **SP** **E** **B** **P** **K** **P** **MP** **M** **D** **M** |
|  | CDH2 | 2 | [**2332**](http://cpdb.molgen.mpg.de/CPDB/showSetDetails?sp=n&st=162) | [**47 (2.0%)**](http://cpdb.molgen.mpg.de/CPDB/showSetDetails?sp=n&st=162) | 0.000154 | 0.0126 | **M** **P** **H** **D** **R** **NB** **P** **I** **I** **H** **C** **BS** **P** **E** **B** **P** **K** **PM** **I** **M** **M** |
|  | h0y3a3_human | 2 | [**1805**](http://cpdb.molgen.mpg.de/CPDB/showSetDetails?sp=n&st=163) | [**39 (2.2%)**](http://cpdb.molgen.mpg.de/CPDB/showSetDetails?sp=n&st=163) | 0.000156 | 0.0127 | **M** **P** **H** **D** **R** **NB** **I** **I** **C** **B** **S** **PE** **B** **P** **P** **P** **M** |
|  | CD63 | 2 | [**2810**](http://cpdb.molgen.mpg.de/CPDB/showSetDetails?sp=n&st=164) | [**54 (1.9%)**](http://cpdb.molgen.mpg.de/CPDB/showSetDetails?sp=n&st=164) | 0.00016 | 0.0128 | **M** **P** **H** **D** **R** **NB** **I** **I** **H** **C** **B** **SP** **E** **B** **P** **K** **P** **MI** **M** **M** |
|  | ST5 | 2 | [**2605**](http://cpdb.molgen.mpg.de/CPDB/showSetDetails?sp=n&st=165) | [**51 (2.0%)**](http://cpdb.molgen.mpg.de/CPDB/showSetDetails?sp=n&st=165) | 0.000161 | 0.0128 | **M** **P** **H** **D** **R** **NB** **I** **I** **H** **C** **S** **PE** **B** **P** **B** **K** **P** **PI** **M** **M** |
|  | FGF2(10-155) | 2 | [**3093**](http://cpdb.molgen.mpg.de/CPDB/showSetDetails?sp=n&st=166) | [**58 (1.9%)**](http://cpdb.molgen.mpg.de/CPDB/showSetDetails?sp=n&st=166) | 0.000162 | 0.0128 | **M** **P** **H** **D** **R** **NB** **P** **I** **I** **H** **C** **BS** **P** **E** **B** **P** **K** **PM** **P** **I** **M** **M** |
|  | TYROBP | 2 | [**2337**](http://cpdb.molgen.mpg.de/CPDB/showSetDetails?sp=n&st=167) | [**47 (2.0%)**](http://cpdb.molgen.mpg.de/CPDB/showSetDetails?sp=n&st=167) | 0.000162 | 0.0128 | **M** **P** **H** **D** **R** **NB** **I** **I** **H** **C** **S** **PE** **B** **P** **B** **K** **PM** **I** **M** **M** |
|  | COL7A1 | 2 | [**2807**](http://cpdb.molgen.mpg.de/CPDB/showSetDetails?sp=n&st=168) | [**54 (1.9%)**](http://cpdb.molgen.mpg.de/CPDB/showSetDetails?sp=n&st=168) | 0.000163 | 0.0128 | **M** **P** **H** **D** **E** **RN** **B** **I** **I** **H** **C** **PP** **S** **B** **B** **K** **PM** **P** **I** **M** **M** |
|  | Dual specificity tyrosine phosphatase YVH1 | 2 | [**3881**](http://cpdb.molgen.mpg.de/CPDB/showSetDetails?sp=n&st=169) | [**69 (1.8%)**](http://cpdb.molgen.mpg.de/CPDB/showSetDetails?sp=n&st=169) | 0.000165 | 0.0128 | **M** **P** **H** **D** **R** **NB** **P** **I** **I** **H** **C** **BS** **P** **E** **B** **P** **K** **PM** **P** **I** **M** **M** |
|  | Epiregulin | 2 | [**1558**](http://cpdb.molgen.mpg.de/CPDB/showSetDetails?sp=n&st=170) | [**35 (2.3%)**](http://cpdb.molgen.mpg.de/CPDB/showSetDetails?sp=n&st=170) | 0.000166 | 0.0128 | **M** **P** **H** **I** **R** **N** **BP** **I** **I** **H** **C** **B** **SP** **E** **B** **P** **K** **P** **MP** **M** **D** **M** |
|  | RAB40A | 2 | [**1435**](http://cpdb.molgen.mpg.de/CPDB/showSetDetails?sp=n&st=171) | [**33 (2.3%)**](http://cpdb.molgen.mpg.de/CPDB/showSetDetails?sp=n&st=171) | 0.000166 | 0.0128 | **M** **P** **H** **R** **D** **NB** **I** **I** **C** **B** **S** **PB** **P** **P** **M** |
|  | CNIH1 | 2 | [**1008**](http://cpdb.molgen.mpg.de/CPDB/showSetDetails?sp=n&st=172) | [**26 (2.6%)**](http://cpdb.molgen.mpg.de/CPDB/showSetDetails?sp=n&st=172) | 0.000168 | 0.0128 | **M** **P** **H** **D** **R** **NB** **I** **I** **H** **C** **B** **SP** **B** **P** **P** **M** |
|  | PROS1(25-676) | 2 | [**2137**](http://cpdb.molgen.mpg.de/CPDB/showSetDetails?sp=n&st=173) | [**44 (2.1%)**](http://cpdb.molgen.mpg.de/CPDB/showSetDetails?sp=n&st=173) | 0.00017 | 0.0129 | **M** **P** **H** **D** **R** **NB** **I** **I** **H** **C** **B** **SP** **E** **B** **P** **K** **P** **IM** **M** |
|  | PLXNA1 | 2 | [**1313**](http://cpdb.molgen.mpg.de/CPDB/showSetDetails?sp=n&st=174) | [**31 (2.4%)**](http://cpdb.molgen.mpg.de/CPDB/showSetDetails?sp=n&st=174) | 0.000173 | 0.0131 | **M** **P** **H** **R** **D** **NB** **I** **I** **C** **B** **S** **PE** **B** **P** **P** **I** **M** **M** |
|  | RAB21 | 2 | [**1193**](http://cpdb.molgen.mpg.de/CPDB/showSetDetails?sp=n&st=175) | [**29 (2.4%)**](http://cpdb.molgen.mpg.de/CPDB/showSetDetails?sp=n&st=175) | 0.000181 | 0.0136 | **M** **P** **H** **D** **R** **NB** **I** **I** **C** **B** **S** **PB** **P** **P** **M** |
|  | d3dqz6_human; e5riw3_human | 2 | [**246**](http://cpdb.molgen.mpg.de/CPDB/showSetDetails?sp=n&st=176) | [**11 (4.5%)**](http://cpdb.molgen.mpg.de/CPDB/showSetDetails?sp=n&st=176) | 0.000182 | 0.0136 | **P** **H** **D** **R** **P** **P** **BM** **I** |
|  | RAB14 | 1 | [**576**](http://cpdb.molgen.mpg.de/CPDB/showSetDetails?sp=n&st=177) | [**18 (3.1%)**](http://cpdb.molgen.mpg.de/CPDB/showSetDetails?sp=n&st=177) | 0.000182 | 0.113 | **H** **S** **P** **R** **B** **BM** **I** |
|  | TAF11 | 2 | [**3399**](http://cpdb.molgen.mpg.de/CPDB/showSetDetails?sp=n&st=178) | [**62 (1.8%)**](http://cpdb.molgen.mpg.de/CPDB/showSetDetails?sp=n&st=178) | 0.000184 | 0.0137 | **M** **P** **H** **R** **D** **NB** **I** **I** **H** **C** **B** **SP** **E** **B** **P** **K** **P** **MP** **I** **M** |
|  | UPF3A | 2 | [**2213**](http://cpdb.molgen.mpg.de/CPDB/showSetDetails?sp=n&st=179) | [**45 (2.0%)**](http://cpdb.molgen.mpg.de/CPDB/showSetDetails?sp=n&st=179) | 0.000188 | 0.0138 | **M** **P** **H** **I** **R** **N** **BI** **I** **H** **C** **B** **S** **PE** **B** **P** **K** **P** **P** **MD** |
|  | TRAPPC5 | 2 | [**1753**](http://cpdb.molgen.mpg.de/CPDB/showSetDetails?sp=n&st=180) | [**38 (2.2%)**](http://cpdb.molgen.mpg.de/CPDB/showSetDetails?sp=n&st=180) | 0.000188 | 0.0138 | **M** **P** **H** **R** **D** **NB** **I** **I** **H** **C** **B** **SP** **E** **B** **P** **K** **P** **PI** **M** **M** |
|  | j3krn4_human | 2 | [**292**](http://cpdb.molgen.mpg.de/CPDB/showSetDetails?sp=n&st=181) | [**12 (4.1%)**](http://cpdb.molgen.mpg.de/CPDB/showSetDetails?sp=n&st=181) | 0.000192 | 0.014 | **C** **H** **B** **S** **P** **R** **DK** **B** **P** **M** **I** **I** **H** |
|  | PPIL2 | 2 | [**2214**](http://cpdb.molgen.mpg.de/CPDB/showSetDetails?sp=n&st=182) | [**45 (2.0%)**](http://cpdb.molgen.mpg.de/CPDB/showSetDetails?sp=n&st=182) | 0.000194 | 0.0141 | **M** **P** **H** **I** **R** **N** **BI** **I** **H** **C** **S** **P** **EB** **P** **B** **K** **P** **PM** **D** |
|  | PML | 2 | [**5425**](http://cpdb.molgen.mpg.de/CPDB/showSetDetails?sp=n&st=183) | [**89 (1.7%)**](http://cpdb.molgen.mpg.de/CPDB/showSetDetails?sp=n&st=183) | 0.000196 | 0.0141 | **M** **P** **H** **D** **R** **NB** **P** **I** **I** **H** **C** **BS** **P** **E** **B** **P** **K** **PM** **P** **I** **M** **M** |
|  | RHOBTB3 | 2 | [**1207**](http://cpdb.molgen.mpg.de/CPDB/showSetDetails?sp=n&st=184) | [**29 (2.4%)**](http://cpdb.molgen.mpg.de/CPDB/showSetDetails?sp=n&st=184) | 0.000203 | 0.0143 | **M** **P** **H** **R** **D** **NB** **I** **I** **C** **B** **S** **PB** **P** **P** **M** **I** **M** |
|  | ATP9A | 2 | [**2706**](http://cpdb.molgen.mpg.de/CPDB/showSetDetails?sp=n&st=185) | [**52 (1.9%)**](http://cpdb.molgen.mpg.de/CPDB/showSetDetails?sp=n&st=185) | 0.000203 | 0.0143 | **M** **P** **H** **I** **R** **N** **BP** **I** **I** **H** **C** **B** **SP** **E** **B** **P** **K** **P** **MP** **M** **D** **M** |
|  | CIAO1 | 2 | [**1827**](http://cpdb.molgen.mpg.de/CPDB/showSetDetails?sp=n&st=186) | [**39 (2.1%)**](http://cpdb.molgen.mpg.de/CPDB/showSetDetails?sp=n&st=186) | 0.000203 | 0.0143 | **M** **P** **H** **D** **R** **NB** **I** **I** **H** **C** **B** **SP** **B** **P** **K** **P** **M** **PI** **M** |
|  | ADRBK1 | 2 | [**4067**](http://cpdb.molgen.mpg.de/CPDB/showSetDetails?sp=n&st=187) | [**71 (1.8%)**](http://cpdb.molgen.mpg.de/CPDB/showSetDetails?sp=n&st=187) | 0.000204 | 0.0143 | **M** **P** **H** **R** **D** **NB** **P** **I** **I** **H** **C** **BS** **P** **E** **B** **P** **K** **PM** **P** **I** **M** **M** |
|  | PLD1 | 2 | [**4138**](http://cpdb.molgen.mpg.de/CPDB/showSetDetails?sp=n&st=188) | [**72 (1.7%)**](http://cpdb.molgen.mpg.de/CPDB/showSetDetails?sp=n&st=188) | 0.000205 | 0.0143 | **M** **P** **H** **D** **R** **NB** **I** **I** **H** **C** **B** **SP** **E** **B** **P** **K** **P** **MP** **I** **M** **M** |
|  | HLA class II histocompatibility antigen, DP | 2 | [**2026**](http://cpdb.molgen.mpg.de/CPDB/showSetDetails?sp=n&st=189) | [**42 (2.1%)**](http://cpdb.molgen.mpg.de/CPDB/showSetDetails?sp=n&st=189) | 0.000207 | 0.0143 | **M** **P** **H** **D** **R** **NB** **P** **I** **I** **H** **C** **BS** **P** **E** **B** **P** **P** **MI** **M** **M** |
|  | MTF2 | 2 | [**2090**](http://cpdb.molgen.mpg.de/CPDB/showSetDetails?sp=n&st=190) | [**43 (2.1%)**](http://cpdb.molgen.mpg.de/CPDB/showSetDetails?sp=n&st=190) | 0.000208 | 0.0143 | **M** **P** **H** **D** **R** **NB** **I** **I** **H** **C** **B** **SP** **E** **B** **P** **K** **P** **MP** **I** **M** |
|  | h3bsk8_human | 2 | [**2628**](http://cpdb.molgen.mpg.de/CPDB/showSetDetails?sp=n&st=191) | [**51 (1.9%)**](http://cpdb.molgen.mpg.de/CPDB/showSetDetails?sp=n&st=191) | 0.000208 | 0.0143 | **M** **P** **H** **D** **R** **NB** **I** **I** **H** **C** **S** **PE** **B** **P** **B** **K** **P** **PI** **M** |
|  | RAB29 | 2 | [**1204**](http://cpdb.molgen.mpg.de/CPDB/showSetDetails?sp=n&st=192) | [**29 (2.4%)**](http://cpdb.molgen.mpg.de/CPDB/showSetDetails?sp=n&st=192) | 0.000209 | 0.0143 | **M** **P** **H** **R** **D** **NB** **I** **I** **C** **B** **S** **PE** **B** **P** **P** **M** |
|  | CCDC33 | 2 | [**2913**](http://cpdb.molgen.mpg.de/CPDB/showSetDetails?sp=n&st=193) | [**55 (1.9%)**](http://cpdb.molgen.mpg.de/CPDB/showSetDetails?sp=n&st=193) | 0.000209 | 0.0143 | **M** **P** **H** **I** **R** **N** **BP** **I** **I** **C** **S** **P** **E** **BP** **B** **P** **P** **M** **DM** |
|  | GPRC5A | 1 | [**741**](http://cpdb.molgen.mpg.de/CPDB/showSetDetails?sp=n&st=194) | [**21 (2.8%)**](http://cpdb.molgen.mpg.de/CPDB/showSetDetails?sp=n&st=194) | 0.000211 | 0.117 | **P** **B** **S** **I** **N** |
|  | CD109 | 2 | [**2983**](http://cpdb.molgen.mpg.de/CPDB/showSetDetails?sp=n&st=195) | [**56 (1.9%)**](http://cpdb.molgen.mpg.de/CPDB/showSetDetails?sp=n&st=195) | 0.000211 | 0.0143 | **M** **P** **H** **D** **R** **NB** **P** **I** **I** **H** **C** **BS** **P** **E** **B** **P** **K** **PM** **I** **M** **M** |
|  | FABP3 | 2 | [**1516**](http://cpdb.molgen.mpg.de/CPDB/showSetDetails?sp=n&st=196) | [**34 (2.3%)**](http://cpdb.molgen.mpg.de/CPDB/showSetDetails?sp=n&st=196) | 0.000212 | 0.0143 | **M** **P** **H** **D** **R** **NB** **P** **I** **I** **C** **S** **P** **EB** **P** **B** **P** **M** **MM** |
|  | MAP/microtubule affinity-regulating kinase 1 | 2 | [**2494**](http://cpdb.molgen.mpg.de/CPDB/showSetDetails?sp=n&st=197) | [**49 (2.0%)**](http://cpdb.molgen.mpg.de/CPDB/showSetDetails?sp=n&st=197) | 0.000214 | 0.0143 | **M** **P** **H** **I** **R** **N** **BP** **I** **I** **H** **C** **B** **SP** **E** **B** **P** **K** **P** **MP** **M** **D** |
|  | PARD3 | 1 | [**691**](http://cpdb.molgen.mpg.de/CPDB/showSetDetails?sp=n&st=198) | [**20 (2.9%)**](http://cpdb.molgen.mpg.de/CPDB/showSetDetails?sp=n&st=198) | 0.000219 | 0.117 | **M** **I** **R** |
|  | bscl2_human | 2 | [**1148**](http://cpdb.molgen.mpg.de/CPDB/showSetDetails?sp=n&st=199) | [**28 (2.5%)**](http://cpdb.molgen.mpg.de/CPDB/showSetDetails?sp=n&st=199) | 0.00022 | 0.0145 | **H** **R** **B** **I** **I** **H** **CS** **P** **E** **B** **P** **B** **KM** **M** **M** |
|  | znhi3_human | 2 | [**2506**](http://cpdb.molgen.mpg.de/CPDB/showSetDetails?sp=n&st=200) | [**49 (2.0%)**](http://cpdb.molgen.mpg.de/CPDB/showSetDetails?sp=n&st=200) | 0.000222 | 0.0145 | **M** **P** **H** **D** **R** **NB** **I** **I** **H** **C** **P** **PE** **B** **B** **K** **P** **MP** **I** **M** **S** |
|  | PRTN3 | 2 | [**2502**](http://cpdb.molgen.mpg.de/CPDB/showSetDetails?sp=n&st=201) | [**49 (2.0%)**](http://cpdb.molgen.mpg.de/CPDB/showSetDetails?sp=n&st=201) | 0.000224 | 0.0145 | **M** **P** **H** **I** **R** **N** **BI** **I** **H** **C** **B** **S** **PE** **B** **P** **K** **P** **MD** **M** **D** **M** |
|  | HTRA1 | 2 | [**1968**](http://cpdb.molgen.mpg.de/CPDB/showSetDetails?sp=n&st=202) | [**41 (2.1%)**](http://cpdb.molgen.mpg.de/CPDB/showSetDetails?sp=n&st=202) | 0.000225 | 0.0145 | **M** **P** **H** **I** **R** **N** **BP** **I** **I** **H** **C** **S** **P** **EB** **P** **B** **K** **P** **MP** **M** **D** **M** |
|  | CNKSR1 | 2 | [**1711**](http://cpdb.molgen.mpg.de/CPDB/showSetDetails?sp=n&st=203) | [**37 (2.2%)**](http://cpdb.molgen.mpg.de/CPDB/showSetDetails?sp=n&st=203) | 0.000226 | 0.0145 | **M** **P** **H** **D** **R** **NB** **I** **I** **H** **C** **S** **PE** **B** **P** **B** **K** **PM** **I** **M** **M** |
|  | MMP20 | 2 | [**535**](http://cpdb.molgen.mpg.de/CPDB/showSetDetails?sp=n&st=204) | [**17 (3.2%)**](http://cpdb.molgen.mpg.de/CPDB/showSetDetails?sp=n&st=204) | 0.000226 | 0.0145 | **M** **P** **H** **D** **R** **NB** **I** **I** **C** **B** **S** **PB** **P** **P** **M** **M** |
|  | CCNH | 2 | [**3774**](http://cpdb.molgen.mpg.de/CPDB/showSetDetails?sp=n&st=205) | [**67 (1.8%)**](http://cpdb.molgen.mpg.de/CPDB/showSetDetails?sp=n&st=205) | 0.000227 | 0.0145 | **M** **P** **H** **R** **D** **NB** **P** **I** **I** **H** **C** **BS** **P** **E** **B** **P** **K** **PM** **P** **I** **M** **M** |
|  | SEPSECS | 2 | [**694**](http://cpdb.molgen.mpg.de/CPDB/showSetDetails?sp=n&st=206) | [**20 (2.9%)**](http://cpdb.molgen.mpg.de/CPDB/showSetDetails?sp=n&st=206) | 0.000227 | 0.0145 | **H** **R** **D** **N** **B** **I** **IH** **C** **S** **E** **B** **P** **BK** **M** **I** **M** |
|  | GRIA1 | 2 | [**2364**](http://cpdb.molgen.mpg.de/CPDB/showSetDetails?sp=n&st=207) | [**47 (2.0%)**](http://cpdb.molgen.mpg.de/CPDB/showSetDetails?sp=n&st=207) | 0.000228 | 0.0145 | **M** **P** **H** **D** **R** **NB** **P** **I** **I** **H** **C** **BS** **P** **E** **B** **P** **K** **PM** **I** **M** **M** |
|  | CCNK | 2 | [**4154**](http://cpdb.molgen.mpg.de/CPDB/showSetDetails?sp=n&st=208) | [**72 (1.7%)**](http://cpdb.molgen.mpg.de/CPDB/showSetDetails?sp=n&st=208) | 0.000228 | 0.0145 | **M** **P** **H** **R** **D** **NB** **P** **I** **I** **H** **C** **BS** **P** **E** **B** **P** **K** **PP** **I** **M** **M** |
|  | DAPK2 | 2 | [**2709**](http://cpdb.molgen.mpg.de/CPDB/showSetDetails?sp=n&st=209) | [**52 (1.9%)**](http://cpdb.molgen.mpg.de/CPDB/showSetDetails?sp=n&st=209) | 0.000234 | 0.0146 | **M** **P** **H** **D** **R** **NB** **P** **I** **I** **H** **C** **BS** **P** **E** **B** **P** **P** **MP** **I** **M** |
|  | Ecotropic viral integration site1 | 2 | [**587**](http://cpdb.molgen.mpg.de/CPDB/showSetDetails?sp=n&st=210) | [**18 (3.1%)**](http://cpdb.molgen.mpg.de/CPDB/showSetDetails?sp=n&st=210) | 0.000235 | 0.0146 | **M** **P** **H** **D** **R** **NB** **I** **I** **H** **C** **S** **PB** **P** **B** **P** **P** **I** **M** |
|  | PCYOX1L | 2 | [**1837**](http://cpdb.molgen.mpg.de/CPDB/showSetDetails?sp=n&st=211) | [**39 (2.1%)**](http://cpdb.molgen.mpg.de/CPDB/showSetDetails?sp=n&st=211) | 0.000235 | 0.0146 | **M** **P** **H** **D** **R** **NB** **I** **I** **H** **C** **B** **SP** **B** **P** **P** **M** **I** **MM** |
|  | CRTAP | 2 | [**2643**](http://cpdb.molgen.mpg.de/CPDB/showSetDetails?sp=n&st=212) | [**51 (1.9%)**](http://cpdb.molgen.mpg.de/CPDB/showSetDetails?sp=n&st=212) | 0.000235 | 0.0146 | **M** **P** **H** **R** **D** **NB** **I** **I** **H** **C** **B** **SP** **E** **B** **P** **K** **P** **MP** **I** **M** **M** |
|  | LMAN2L | 2 | [**1093**](http://cpdb.molgen.mpg.de/CPDB/showSetDetails?sp=n&st=213) | [**27 (2.5%)**](http://cpdb.molgen.mpg.de/CPDB/showSetDetails?sp=n&st=213) | 0.000237 | 0.0146 | **M** **P** **H** **D** **R** **NB** **I** **I** **H** **C** **B** **SP** **B** **P** **P** **M** |
|  | ITGAX | 2 | [**3071**](http://cpdb.molgen.mpg.de/CPDB/showSetDetails?sp=n&st=214) | [**57 (1.9%)**](http://cpdb.molgen.mpg.de/CPDB/showSetDetails?sp=n&st=214) | 0.000239 | 0.0146 | **M** **P** **H** **D** **R** **NB** **I** **I** **H** **C** **S** **PE** **B** **P** **B** **K** **PM** **I** **M** **M** |
|  | TRAPPC2L | 2 | [**1776**](http://cpdb.molgen.mpg.de/CPDB/showSetDetails?sp=n&st=215) | [**38 (2.1%)**](http://cpdb.molgen.mpg.de/CPDB/showSetDetails?sp=n&st=215) | 0.000239 | 0.0146 | **M** **P** **H** **R** **D** **NB** **I** **I** **H** **C** **B** **SP** **B** **P** **P** **M** |
|  | CTSL2 | 2 | [**1716**](http://cpdb.molgen.mpg.de/CPDB/showSetDetails?sp=n&st=216) | [**37 (2.2%)**](http://cpdb.molgen.mpg.de/CPDB/showSetDetails?sp=n&st=216) | 0.000243 | 0.0146 | **M** **P** **H** **I** **R** **N** **BI** **I** **H** **C** **B** **S** **PE** **B** **P** **K** **P** **MM** **D** **M** |
|  | ITGA1 | 2 | [**3067**](http://cpdb.molgen.mpg.de/CPDB/showSetDetails?sp=n&st=217) | [**57 (1.9%)**](http://cpdb.molgen.mpg.de/CPDB/showSetDetails?sp=n&st=217) | 0.000243 | 0.0146 | **M** **P** **H** **D** **R** **NB** **P** **I** **I** **H** **C** **BS** **P** **E** **B** **P** **P** **MI** **M** **M** |
|  | DUSP16 | 2 | [**3931**](http://cpdb.molgen.mpg.de/CPDB/showSetDetails?sp=n&st=218) | [**69 (1.8%)**](http://cpdb.molgen.mpg.de/CPDB/showSetDetails?sp=n&st=218) | 0.000244 | 0.0146 | **M** **P** **H** **I** **R** **N** **BP** **I** **I** **H** **C** **B** **SP** **E** **B** **P** **P** **M** **PM** **D** **M** |
|  | STRA6 | 2 | [**487**](http://cpdb.molgen.mpg.de/CPDB/showSetDetails?sp=n&st=219) | [**16 (3.3%)**](http://cpdb.molgen.mpg.de/CPDB/showSetDetails?sp=n&st=219) | 0.000244 | 0.0146 | **H** **S** **P** **B** **D** **R** **PP** **B** **M** **I** **I** **M** |
|  | HIST1H2AJ | 2 | [**3788**](http://cpdb.molgen.mpg.de/CPDB/showSetDetails?sp=n&st=220) | [**67 (1.8%)**](http://cpdb.molgen.mpg.de/CPDB/showSetDetails?sp=n&st=220) | 0.000244 | 0.0146 | **M** **P** **H** **D** **R** **NB** **I** **I** **H** **C** **B** **SP** **E** **B** **P** **K** **P** **MP** **I** **M** **M** |
|  | OxA-GNS | 2 | [**3720**](http://cpdb.molgen.mpg.de/CPDB/showSetDetails?sp=n&st=221) | [**66 (1.8%)**](http://cpdb.molgen.mpg.de/CPDB/showSetDetails?sp=n&st=221) | 0.000245 | 0.0146 | **M** **P** **H** **I** **R** **N** **BP** **I** **I** **H** **C** **B** **SP** **E** **B** **P** **K** **P** **PM** **D** **M** |
|  | ICAM5 | 2 | [**4234**](http://cpdb.molgen.mpg.de/CPDB/showSetDetails?sp=n&st=222) | [**73 (1.7%)**](http://cpdb.molgen.mpg.de/CPDB/showSetDetails?sp=n&st=222) | 0.000246 | 0.0146 | **M** **P** **H** **D** **R** **NB** **P** **I** **I** **H** **C** **SP** **E** **B** **P** **B** **K** **PM** **P** **I** **M** **M** |
|  | TAF5 | 2 | [**2382**](http://cpdb.molgen.mpg.de/CPDB/showSetDetails?sp=n&st=223) | [**47 (2.0%)**](http://cpdb.molgen.mpg.de/CPDB/showSetDetails?sp=n&st=223) | 0.000249 | 0.0147 | **M** **P** **H** **R** **D** **NB** **I** **I** **C** **B** **S** **PE** **B** **P** **P** **P** **I** **M** |
|  | PGAP1 | 2 | [**1786**](http://cpdb.molgen.mpg.de/CPDB/showSetDetails?sp=n&st=224) | [**38 (2.1%)**](http://cpdb.molgen.mpg.de/CPDB/showSetDetails?sp=n&st=224) | 0.00025 | 0.0147 | **M** **P** **H** **R** **D** **NB** **I** **I** **H** **C** **S** **PE** **B** **P** **B** **K** **PM** **P** **I** **M** **M** |
|  | e9psf2_human | 2 | [**1402**](http://cpdb.molgen.mpg.de/CPDB/showSetDetails?sp=n&st=225) | [**32 (2.3%)**](http://cpdb.molgen.mpg.de/CPDB/showSetDetails?sp=n&st=225) | 0.000253 | 0.0147 | **P** **H** **R** **D** **N** **B** **II** **C** **S** **P** **E** **B** **PK** **P** **P** **M** |
|  | ARNTL gene | 2 | [**2727**](http://cpdb.molgen.mpg.de/CPDB/showSetDetails?sp=n&st=226) | [**52 (1.9%)**](http://cpdb.molgen.mpg.de/CPDB/showSetDetails?sp=n&st=226) | 0.000254 | 0.0147 | **M** **P** **H** **D** **R** **NB** **I** **I** **H** **C** **S** **PE** **B** **P** **B** **K** **PM** **P** **I** **M** **M** |
|  | actl8_human | 2 | [**3003**](http://cpdb.molgen.mpg.de/CPDB/showSetDetails?sp=n&st=227) | [**56 (1.9%)**](http://cpdb.molgen.mpg.de/CPDB/showSetDetails?sp=n&st=227) | 0.000254 | 0.0147 | **M** **P** **H** **I** **R** **N** **BP** **I** **I** **H** **C** **S** **P** **EB** **P** **B** **K** **P** **MP** **M** **D** |
|  | TUBA1A | 2 | [**4542**](http://cpdb.molgen.mpg.de/CPDB/showSetDetails?sp=n&st=228) | [**77 (1.7%)**](http://cpdb.molgen.mpg.de/CPDB/showSetDetails?sp=n&st=228) | 0.000258 | 0.0148 | **M** **P** **H** **R** **D** **NB** **I** **I** **H** **C** **B** **SP** **E** **B** **P** **K** **P** **MP** **I** **M** **M** |
|  | sodium channel, voltage-gated, type I, beta | 2 | [**1469**](http://cpdb.molgen.mpg.de/CPDB/showSetDetails?sp=n&st=229) | [**33 (2.3%)**](http://cpdb.molgen.mpg.de/CPDB/showSetDetails?sp=n&st=229) | 0.000259 | 0.0148 | **M** **P** **H** **D** **R** **NB** **I** **I** **H** **C** **S** **PE** **B** **P** **B** **K** **PM** **P** **M** **M** |
|  | LTBP3 | 2 | [**595**](http://cpdb.molgen.mpg.de/CPDB/showSetDetails?sp=n&st=230) | [**18 (3.0%)**](http://cpdb.molgen.mpg.de/CPDB/showSetDetails?sp=n&st=230) | 0.00026 | 0.0148 | **M** **P** **H** **R** **D** **NB** **I** **I** **C** **S** **P** **BP** **B** **P** **I** **M** **M** |
|  | FBXO3 | 2 | [**1528**](http://cpdb.molgen.mpg.de/CPDB/showSetDetails?sp=n&st=231) | [**34 (2.2%)**](http://cpdb.molgen.mpg.de/CPDB/showSetDetails?sp=n&st=231) | 0.000261 | 0.0148 | **M** **P** **H** **I** **R** **N** **BI** **I** **H** **C** **S** **P** **EB** **P** **B** **K** **P** **MP** **M** **D** |
|  | HLA class I histocompatibility antigen, E alpha chain precursor | 2 | [**2049**](http://cpdb.molgen.mpg.de/CPDB/showSetDetails?sp=n&st=232) | [**42 (2.1%)**](http://cpdb.molgen.mpg.de/CPDB/showSetDetails?sp=n&st=232) | 0.000265 | 0.015 | **M** **P** **H** **I** **R** **N** **BP** **I** **I** **H** **C** **B** **SP** **E** **B** **P** **K** **P** **MM** **D** |
|  | cq096_human | 2 | [**1102**](http://cpdb.molgen.mpg.de/CPDB/showSetDetails?sp=n&st=233) | [**27 (2.5%)**](http://cpdb.molgen.mpg.de/CPDB/showSetDetails?sp=n&st=233) | 0.000266 | 0.015 | **P** **H** **R** **D** **N** **B** **II** **C** **B** **S** **P** **E** **BP** **K** **P** **P** **M** |
|  | SELL | 2 | [**2457**](http://cpdb.molgen.mpg.de/CPDB/showSetDetails?sp=n&st=234) | [**48 (2.0%)**](http://cpdb.molgen.mpg.de/CPDB/showSetDetails?sp=n&st=234) | 0.000267 | 0.015 | **M** **P** **H** **D** **R** **NB** **I** **I** **H** **C** **B** **SP** **E** **B** **P** **K** **P** **MI** **M** **M** |
|  | RAB30 | 1 | [**75**](http://cpdb.molgen.mpg.de/CPDB/showSetDetails?sp=n&st=235) | [**6 (8.0%)**](http://cpdb.molgen.mpg.de/CPDB/showSetDetails?sp=n&st=235) | 0.000269 | 0.134 | **I** **H** **R** **B** **B** |
|  | C1QA | 2 | [**1986**](http://cpdb.molgen.mpg.de/CPDB/showSetDetails?sp=n&st=236) | [**41 (2.1%)**](http://cpdb.molgen.mpg.de/CPDB/showSetDetails?sp=n&st=236) | 0.00027 | 0.0151 | **M** **P** **H** **D** **R** **ND** **B** **I** **I** **H** **C** **BS** **P** **E** **B** **P** **K** **PM** **P** **I** **M** **M** |
|  | ZSCAN10 | 2 | [**348**](http://cpdb.molgen.mpg.de/CPDB/showSetDetails?sp=n&st=237) | [**13 (3.7%)**](http://cpdb.molgen.mpg.de/CPDB/showSetDetails?sp=n&st=237) | 0.000277 | 0.0153 | **P** **C** **H** **B** **S** **D** **RP** **B** **M** **I** |
|  | Fibronectin matrix | 2 | [**1596**](http://cpdb.molgen.mpg.de/CPDB/showSetDetails?sp=n&st=238) | [**35 (2.2%)**](http://cpdb.molgen.mpg.de/CPDB/showSetDetails?sp=n&st=238) | 0.000277 | 0.0153 | **M** **P** **H** **D** **R** **NB** **P** **I** **I** **C** **B** **SP** **E** **B** **P** **P** **M** **IM** **M** |
|  | SLC27A2 | 2 | [**3957**](http://cpdb.molgen.mpg.de/CPDB/showSetDetails?sp=n&st=239) | [**69 (1.8%)**](http://cpdb.molgen.mpg.de/CPDB/showSetDetails?sp=n&st=239) | 0.00028 | 0.0154 | **M** **P** **H** **I** **R** **N** **BC** **I** **I** **H** **D** **B** **SP** **E** **B** **P** **K** **P** **MP** **M** **D** **M** |
|  | pex6_human | 2 | [**217**](http://cpdb.molgen.mpg.de/CPDB/showSetDetails?sp=n&st=240) | [**10 (4.6%)**](http://cpdb.molgen.mpg.de/CPDB/showSetDetails?sp=n&st=240) | 0.000281 | 0.0154 | **C** **H** **S** **R** **B** **P** **BP** **M** **I** **I** |
|  | MAPK9 | 2 | [**4251**](http://cpdb.molgen.mpg.de/CPDB/showSetDetails?sp=n&st=241) | [**73 (1.7%)**](http://cpdb.molgen.mpg.de/CPDB/showSetDetails?sp=n&st=241) | 0.000283 | 0.0155 | **M** **P** **H** **I** **R** **N** **BP** **I** **I** **H** **C** **S** **P** **EB** **P** **B** **K** **P** **MP** **M** **D** **M** |
|  | e9ph98_human | 2 | [**178**](http://cpdb.molgen.mpg.de/CPDB/showSetDetails?sp=n&st=242) | [**9 (5.1%)**](http://cpdb.molgen.mpg.de/CPDB/showSetDetails?sp=n&st=242) | 0.000287 | 0.0156 | **P** **C** **H** **S** **P** **B** **RB** **P** **M** **I** |
|  | e7etz0_human | 2 | [**1409**](http://cpdb.molgen.mpg.de/CPDB/showSetDetails?sp=n&st=243) | [**32 (2.3%)**](http://cpdb.molgen.mpg.de/CPDB/showSetDetails?sp=n&st=243) | 0.000287 | 0.0156 | **M** **P** **H** **R** **D** **NB** **I** **I** **H** **C** **S** **PB** **P** **K** **P** **M** **I** **M** |
|  | MYH6 | 2 | [**2882**](http://cpdb.molgen.mpg.de/CPDB/showSetDetails?sp=n&st=244) | [**54 (1.9%)**](http://cpdb.molgen.mpg.de/CPDB/showSetDetails?sp=n&st=244) | 0.00029 | 0.0157 | **M** **P** **H** **R** **D** **NB** **P** **I** **I** **H** **C** **BS** **P** **E** **B** **P** **K** **PM** **P** **I** **M** **M** |
|  | CACNA2D2(19-1150) | 2 | [**1602**](http://cpdb.molgen.mpg.de/CPDB/showSetDetails?sp=n&st=245) | [**35 (2.2%)**](http://cpdb.molgen.mpg.de/CPDB/showSetDetails?sp=n&st=245) | 0.000294 | 0.0158 | **M** **P** **H** **R** **D** **NB** **I** **I** **H** **C** **S** **PE** **B** **P** **B** **K** **PM** **P** **I** **M** **M** |
|  | AP1S1 | 2 | [**2264**](http://cpdb.molgen.mpg.de/CPDB/showSetDetails?sp=n&st=246) | [**45 (2.0%)**](http://cpdb.molgen.mpg.de/CPDB/showSetDetails?sp=n&st=246) | 0.000296 | 0.0158 | **M** **P** **H** **D** **R** **NB** **P** **I** **I** **C** **B** **SP** **E** **B** **P** **K** **P** **MI** **M** |
|  | LCOR | 2 | [**2819**](http://cpdb.molgen.mpg.de/CPDB/showSetDetails?sp=n&st=247) | [**53 (1.9%)**](http://cpdb.molgen.mpg.de/CPDB/showSetDetails?sp=n&st=247) | 0.0003 | 0.016 | **M** **P** **H** **R** **D** **NB** **I** **I** **H** **C** **S** **PE** **B** **P** **B** **K** **PM** **P** **I** **M** **M** |
|  | Laminin Alpha; Laminin Gamma; Laminin Beta | 2 | [**764**](http://cpdb.molgen.mpg.de/CPDB/showSetDetails?sp=n&st=248) | [**21 (2.8%)**](http://cpdb.molgen.mpg.de/CPDB/showSetDetails?sp=n&st=248) | 0.000301 | 0.016 | **M** **P** **H** **I** **R** **N** **BI** **I** **C** **B** **S** **E** **BP** **P** **M** **D** **M** |
|  | s35f6_human | 2 | [**3820**](http://cpdb.molgen.mpg.de/CPDB/showSetDetails?sp=n&st=249) | [**67 (1.8%)**](http://cpdb.molgen.mpg.de/CPDB/showSetDetails?sp=n&st=249) | 0.000306 | 0.0161 | **M** **P** **H** **R** **D** **NB** **I** **I** **H** **C** **S** **PE** **B** **P** **B** **K** **PM** **P** **I** **M** |
|  | GIT1 | 2 | [**3599**](http://cpdb.molgen.mpg.de/CPDB/showSetDetails?sp=n&st=250) | [**64 (1.8%)**](http://cpdb.molgen.mpg.de/CPDB/showSetDetails?sp=n&st=250) | 0.000306 | 0.0161 | **M** **P** **H** **D** **R** **NB** **P** **I** **I** **H** **C** **BS** **P** **E** **B** **P** **K** **PM** **P** **I** **M** **M** |
|  | LDLR | 2 | [**2880**](http://cpdb.molgen.mpg.de/CPDB/showSetDetails?sp=n&st=251) | [**54 (1.9%)**](http://cpdb.molgen.mpg.de/CPDB/showSetDetails?sp=n&st=251) | 0.000309 | 0.0162 | **M** **P** **H** **D** **R** **NB** **P** **I** **I** **H** **C** **BS** **P** **E** **B** **P** **K** **PM** **I** **M** **M** |
|  | LATS1 | 2 | [**2676**](http://cpdb.molgen.mpg.de/CPDB/showSetDetails?sp=n&st=252) | [**51 (1.9%)**](http://cpdb.molgen.mpg.de/CPDB/showSetDetails?sp=n&st=252) | 0.00031 | 0.0162 | **M** **P** **H** **D** **R** **NB** **P** **I** **I** **H** **C** **SP** **E** **B** **P** **B** **K** **PI** **M** |
|  | RAB11FIP3 | 2 | [**711**](http://cpdb.molgen.mpg.de/CPDB/showSetDetails?sp=n&st=253) | [**20 (2.8%)**](http://cpdb.molgen.mpg.de/CPDB/showSetDetails?sp=n&st=253) | 0.000311 | 0.0162 | **P** **H** **D** **R** **N** **B** **II** **C** **S** **P** **E** **B** **PB** **P** **M** **M** |
|  | Aprataxin | 2 | [**3096**](http://cpdb.molgen.mpg.de/CPDB/showSetDetails?sp=n&st=254) | [**57 (1.8%)**](http://cpdb.molgen.mpg.de/CPDB/showSetDetails?sp=n&st=254) | 0.000314 | 0.0162 | **M** **P** **H** **D** **R** **NB** **P** **I** **I** **H** **C** **SP** **E** **B** **P** **B** **K** **PM** **P** **I** **M** **M** |
|  | protein; CNIH2 | 2 | [**876**](http://cpdb.molgen.mpg.de/CPDB/showSetDetails?sp=n&st=255) | [**23 (2.6%)**](http://cpdb.molgen.mpg.de/CPDB/showSetDetails?sp=n&st=255) | 0.000316 | 0.0162 | **M** **P** **H** **D** **R** **NB** **I** **I** **H** **C** **B** **SP** **B** **P** **P** **M** |
|  | LAMC1 | 2 | [**3029**](http://cpdb.molgen.mpg.de/CPDB/showSetDetails?sp=n&st=256) | [**56 (1.9%)**](http://cpdb.molgen.mpg.de/CPDB/showSetDetails?sp=n&st=256) | 0.000319 | 0.0162 | **M** **P** **H** **D** **R** **NB** **I** **I** **H** **C** **S** **PE** **B** **P** **B** **P** **M** **PI** **M** **M** |
|  | PPCS | 2 | [**28**](http://cpdb.molgen.mpg.de/CPDB/showSetDetails?sp=n&st=257) | [**4 (14.3%)**](http://cpdb.molgen.mpg.de/CPDB/showSetDetails?sp=n&st=257) | 0.000319 | 0.0162 | **I** **B** |
|  | EIF3K | 2 | [**2337**](http://cpdb.molgen.mpg.de/CPDB/showSetDetails?sp=n&st=258) | [**46 (2.0%)**](http://cpdb.molgen.mpg.de/CPDB/showSetDetails?sp=n&st=258) | 0.000319 | 0.0162 | **M** **P** **H** **D** **R** **NB** **P** **I** **I** **H** **C** **BS** **P** **E** **B** **P** **K** **PM** **I** **M** |
|  | TAF1 | 2 | [**3828**](http://cpdb.molgen.mpg.de/CPDB/showSetDetails?sp=n&st=259) | [**67 (1.8%)**](http://cpdb.molgen.mpg.de/CPDB/showSetDetails?sp=n&st=259) | 0.00032 | 0.0162 | **M** **P** **H** **D** **R** **NB** **I** **I** **H** **C** **B** **SP** **E** **B** **P** **K** **P** **MP** **I** **M** **M** |
|  | SEL1L | 2 | [**3103**](http://cpdb.molgen.mpg.de/CPDB/showSetDetails?sp=n&st=260) | [**57 (1.8%)**](http://cpdb.molgen.mpg.de/CPDB/showSetDetails?sp=n&st=260) | 0.000325 | 0.0163 | **M** **P** **H** **R** **D** **NB** **P** **I** **I** **H** **C** **SP** **E** **B** **P** **B** **K** **PM** **P** **I** **M** **M** |
|  | ASF1A | 2 | [**1940**](http://cpdb.molgen.mpg.de/CPDB/showSetDetails?sp=n&st=261) | [**40 (2.1%)**](http://cpdb.molgen.mpg.de/CPDB/showSetDetails?sp=n&st=261) | 0.000325 | 0.0163 | **M** **P** **H** **D** **R** **NB** **I** **I** **H** **C** **B** **SP** **E** **B** **P** **K** **P** **MP** **I** **M** |
|  | HLA class I histocompatibility antigen, alpha chain G precursor | 2 | [**1177**](http://cpdb.molgen.mpg.de/CPDB/showSetDetails?sp=n&st=262) | [**28 (2.4%)**](http://cpdb.molgen.mpg.de/CPDB/showSetDetails?sp=n&st=262) | 0.00033 | 0.0164 | **M** **P** **H** **I** **R** **N** **BI** **I** **S** **P** **E** **B** **P** **BK** **P** **M** **D** |
|  | NHERF2 | 2 | [**3386**](http://cpdb.molgen.mpg.de/CPDB/showSetDetails?sp=n&st=263) | [**61 (1.8%)**](http://cpdb.molgen.mpg.de/CPDB/showSetDetails?sp=n&st=263) | 0.00033 | 0.0164 | **M** **P** **H** **I** **R** **N** **BP** **I** **I** **H** **C** **B** **SP** **E** **B** **P** **K** **P** **MM** **D** **M** |
|  | RABGGTB | 1 | [**144**](http://cpdb.molgen.mpg.de/CPDB/showSetDetails?sp=n&st=264) | [**8 (5.6%)**](http://cpdb.molgen.mpg.de/CPDB/showSetDetails?sp=n&st=264) | 0.000332 | 0.148 | **H** **S** **R** **B** **P** **P** **BM** **I** |
|  | P4HA2 | 2 | [**2959**](http://cpdb.molgen.mpg.de/CPDB/showSetDetails?sp=n&st=265) | [**55 (1.9%)**](http://cpdb.molgen.mpg.de/CPDB/showSetDetails?sp=n&st=265) | 0.000334 | 0.0165 | **M** **P** **H** **I** **R** **N** **BI** **I** **H** **C** **B** **S** **PE** **B** **P** **K** **P** **MM** **D** **M** |
|  | ITGAD | 2 | [**450**](http://cpdb.molgen.mpg.de/CPDB/showSetDetails?sp=n&st=266) | [**15 (3.3%)**](http://cpdb.molgen.mpg.de/CPDB/showSetDetails?sp=n&st=266) | 0.000334 | 0.0165 | **P** **H** **R** **D** **N** **B** **II** **C** **S** **P** **B** **P** **BP** **M** |
|  | HLA class I histocompatibility antigen, Cw-3 alpha chain precursor | 2 | [**1178**](http://cpdb.molgen.mpg.de/CPDB/showSetDetails?sp=n&st=267) | [**28 (2.4%)**](http://cpdb.molgen.mpg.de/CPDB/showSetDetails?sp=n&st=267) | 0.000335 | 0.0165 | **M** **P** **H** **I** **R** **N** **BI** **I** **S** **P** **E** **B** **P** **BK** **P** **M** **D** |
|  | AGA | 1 | [**222**](http://cpdb.molgen.mpg.de/CPDB/showSetDetails?sp=n&st=268) | [**10 (4.5%)**](http://cpdb.molgen.mpg.de/CPDB/showSetDetails?sp=n&st=268) | 0.000337 | 0.148 | **B** **E** **R** **H** **H** |
|  | CDR2 | 2 | [**1612**](http://cpdb.molgen.mpg.de/CPDB/showSetDetails?sp=n&st=269) | [**35 (2.2%)**](http://cpdb.molgen.mpg.de/CPDB/showSetDetails?sp=n&st=269) | 0.000342 | 0.0166 | **M** **P** **H** **D** **R** **NB** **I** **I** **C** **B** **S** **PE** **B** **P** **P** **P** **M** |
|  | melt_human | 2 | [**453**](http://cpdb.molgen.mpg.de/CPDB/showSetDetails?sp=n&st=270) | [**15 (3.3%)**](http://cpdb.molgen.mpg.de/CPDB/showSetDetails?sp=n&st=270) | 0.000342 | 0.0166 | **P** **H** **I** **R** **N** **B** **I** **IH** **S** **P** **E** **B** **P** **BP** **M** **D** |
|  | UNC93B1 | 2 | [**1747**](http://cpdb.molgen.mpg.de/CPDB/showSetDetails?sp=n&st=271) | [**37 (2.1%)**](http://cpdb.molgen.mpg.de/CPDB/showSetDetails?sp=n&st=271) | 0.000345 | 0.0166 | **M** **P** **H** **D** **R** **NB** **I** **I** **H** **C** **S** **PE** **B** **P** **B** **K** **PM** **I** **M** |
|  | S100A2 | 2 | [**1620**](http://cpdb.molgen.mpg.de/CPDB/showSetDetails?sp=n&st=272) | [**35 (2.2%)**](http://cpdb.molgen.mpg.de/CPDB/showSetDetails?sp=n&st=272) | 0.000346 | 0.0166 | **M** **P** **H** **D** **R** **B** **II** **C** **S** **P** **E** **B** **PB** **K** **P** **M** **M** **M** |
|  | ICAM4 | 2 | [**1238**](http://cpdb.molgen.mpg.de/CPDB/showSetDetails?sp=n&st=273) | [**29 (2.3%)**](http://cpdb.molgen.mpg.de/CPDB/showSetDetails?sp=n&st=273) | 0.000346 | 0.0166 | **M** **P** **H** **D** **R** **NB** **I** **I** **C** **B** **S** **PB** **P** **P** **M** **M** **M** |
|  | FBLN1 | 2 | [**2007**](http://cpdb.molgen.mpg.de/CPDB/showSetDetails?sp=n&st=274) | [**41 (2.1%)**](http://cpdb.molgen.mpg.de/CPDB/showSetDetails?sp=n&st=274) | 0.000347 | 0.0166 | **M** **P** **H** **D** **R** **NB** **I** **I** **C** **S** **P** **EB** **P** **B** **P** **M** **I** **MM** |
|  | SOX2 | 2 | [**4129**](http://cpdb.molgen.mpg.de/CPDB/showSetDetails?sp=n&st=275) | [**71 (1.7%)**](http://cpdb.molgen.mpg.de/CPDB/showSetDetails?sp=n&st=275) | 0.000347 | 0.0166 | **M** **P** **H** **D** **R** **NB** **P** **I** **I** **H** **C** **BS** **P** **E** **B** **P** **K** **PM** **P** **I** **M** **M** |
|  | ATF-5 | 2 | [**1181**](http://cpdb.molgen.mpg.de/CPDB/showSetDetails?sp=n&st=276) | [**28 (2.4%)**](http://cpdb.molgen.mpg.de/CPDB/showSetDetails?sp=n&st=276) | 0.000349 | 0.0166 | **M** **P** **H** **R** **D** **NB** **I** **I** **C** **S** **P** **EB** **P** **B** **P** **M** **M** |
|  | CASP8(1-479) | 2 | [**5116**](http://cpdb.molgen.mpg.de/CPDB/showSetDetails?sp=n&st=277) | [**84 (1.6%)**](http://cpdb.molgen.mpg.de/CPDB/showSetDetails?sp=n&st=277) | 0.00035 | 0.0166 | **M** **P** **H** **D** **R** **NB** **P** **I** **I** **H** **C** **BS** **P** **E** **B** **P** **K** **PM** **P** **I** **M** **M** |
|  | FH | 2 | [**2145**](http://cpdb.molgen.mpg.de/CPDB/showSetDetails?sp=n&st=278) | [**43 (2.0%)**](http://cpdb.molgen.mpg.de/CPDB/showSetDetails?sp=n&st=278) | 0.000351 | 0.0166 | **M** **P** **H** **I** **R** **N** **BI** **I** **H** **C** **B** **S** **PE** **B** **P** **K** **P** **MM** **D** **M** |
|  | pab4l_human | 2 | [**183**](http://cpdb.molgen.mpg.de/CPDB/showSetDetails?sp=n&st=279) | [**9 (4.9%)**](http://cpdb.molgen.mpg.de/CPDB/showSetDetails?sp=n&st=279) | 0.000352 | 0.0166 | **P** **C** **H** **S** **P** **R** **BB** **P** **M** **I** |
|  | tm9s1_human | 2 | [**2010**](http://cpdb.molgen.mpg.de/CPDB/showSetDetails?sp=n&st=280) | [**41 (2.0%)**](http://cpdb.molgen.mpg.de/CPDB/showSetDetails?sp=n&st=280) | 0.000354 | 0.0166 | **M** **P** **H** **D** **R** **NB** **P** **I** **I** **H** **C** **BS** **P** **B** **P** **K** **P** **MI** **M** |
|  | CNIH3 | 2 | [**884**](http://cpdb.molgen.mpg.de/CPDB/showSetDetails?sp=n&st=281) | [**23 (2.6%)**](http://cpdb.molgen.mpg.de/CPDB/showSetDetails?sp=n&st=281) | 0.000359 | 0.0168 | **M** **P** **H** **D** **R** **NB** **I** **I** **H** **C** **B** **SP** **B** **P** **P** **M** |
|  | HLA class I histocompatibility antigen, A-2 alpha chain | 2 | [**2351**](http://cpdb.molgen.mpg.de/CPDB/showSetDetails?sp=n&st=282) | [**46 (2.0%)**](http://cpdb.molgen.mpg.de/CPDB/showSetDetails?sp=n&st=282) | 0.000362 | 0.0169 | **M** **P** **H** **I** **R** **N** **BP** **I** **I** **H** **C** **B** **SP** **E** **B** **P** **K** **P** **MM** **D** **M** |
|  | CARD9 | 2 | [**3619**](http://cpdb.molgen.mpg.de/CPDB/showSetDetails?sp=n&st=283) | [**64 (1.8%)**](http://cpdb.molgen.mpg.de/CPDB/showSetDetails?sp=n&st=283) | 0.000365 | 0.0169 | **M** **P** **H** **D** **R** **NB** **P** **I** **I** **H** **C** **SP** **E** **B** **P** **B** **K** **PM** **P** **I** **M** **M** |
|  | CORO1A | 2 | [**1246**](http://cpdb.molgen.mpg.de/CPDB/showSetDetails?sp=n&st=284) | [**29 (2.3%)**](http://cpdb.molgen.mpg.de/CPDB/showSetDetails?sp=n&st=284) | 0.00037 | 0.0171 | **P** **H** **D** **R** **N** **B** **II** **C** **S** **P** **E** **B** **PB** **K** **P** **M** **I** **M** **M** |
|  | TIP30 | 2 | [**507**](http://cpdb.molgen.mpg.de/CPDB/showSetDetails?sp=n&st=285) | [**16 (3.2%)**](http://cpdb.molgen.mpg.de/CPDB/showSetDetails?sp=n&st=285) | 0.000373 | 0.0172 | **P** **H** **R** **D** **N** **B** **II** **H** **C** **S** **P** **E** **BP** **B** **K** **P** **M** **PM** |
|  | ZWINT | 2 | [**3337**](http://cpdb.molgen.mpg.de/CPDB/showSetDetails?sp=n&st=286) | [**60 (1.8%)**](http://cpdb.molgen.mpg.de/CPDB/showSetDetails?sp=n&st=286) | 0.000375 | 0.0172 | **M** **P** **H** **D** **R** **NB** **I** **I** **H** **C** **B** **SP** **E** **B** **P** **K** **P** **MP** **I** **M** **M** |
|  | p-T,Y-MAPK8 | 2 | [**5827**](http://cpdb.molgen.mpg.de/CPDB/showSetDetails?sp=n&st=287) | [**93 (1.6%)**](http://cpdb.molgen.mpg.de/CPDB/showSetDetails?sp=n&st=287) | 0.000376 | 0.0172 | **M** **P** **H** **D** **R** **NB** **I** **I** **H** **C** **B** **SP** **E** **B** **P** **K** **P** **MP** **I** **M** **M** |
|  | DFFB | 2 | [**776**](http://cpdb.molgen.mpg.de/CPDB/showSetDetails?sp=n&st=288) | [**21 (2.7%)**](http://cpdb.molgen.mpg.de/CPDB/showSetDetails?sp=n&st=288) | 0.000377 | 0.0172 | **M** **P** **H** **D** **R** **NB** **I** **I** **C** **B** **S** **BP** **P** **P** **I** **M** |
|  | ANKRD27 | 2 | [**2838**](http://cpdb.molgen.mpg.de/CPDB/showSetDetails?sp=n&st=289) | [**53 (1.9%)**](http://cpdb.molgen.mpg.de/CPDB/showSetDetails?sp=n&st=289) | 0.000381 | 0.0172 | **M** **P** **H** **I** **R** **N** **BI** **I** **H** **C** **B** **S** **PE** **B** **P** **K** **P** **M** **PM** **D** |
|  | (2-5,)oligo(A) synthetase 2 | 2 | [**147**](http://cpdb.molgen.mpg.de/CPDB/showSetDetails?sp=n&st=290) | [**8 (5.4%)**](http://cpdb.molgen.mpg.de/CPDB/showSetDetails?sp=n&st=290) | 0.000382 | 0.0172 | **H** **S** **E** **P** **B** **M** **I** |
|  | LTBP1 | 2 | [**1248**](http://cpdb.molgen.mpg.de/CPDB/showSetDetails?sp=n&st=291) | [**29 (2.3%)**](http://cpdb.molgen.mpg.de/CPDB/showSetDetails?sp=n&st=291) | 0.000385 | 0.0172 | **M** **P** **H** **D** **R** **NB** **I** **I** **H** **C** **S** **PB** **P** **B** **P** **M** **P** **IM** **M** |
|  | PARD3 | 2 | [**4143**](http://cpdb.molgen.mpg.de/CPDB/showSetDetails?sp=n&st=292) | [**71 (1.7%)**](http://cpdb.molgen.mpg.de/CPDB/showSetDetails?sp=n&st=292) | 0.000385 | 0.0172 | **M** **P** **H** **D** **R** **NB** **P** **I** **I** **H** **C** **SP** **E** **B** **P** **B** **K** **PM** **P** **I** **M** |
|  | Shc1 | 2 | [**316**](http://cpdb.molgen.mpg.de/CPDB/showSetDetails?sp=n&st=293) | [**12 (3.8%)**](http://cpdb.molgen.mpg.de/CPDB/showSetDetails?sp=n&st=293) | 0.000385 | 0.0172 | **M** **P** **H** **I** **R** **N** **BI** **I** **S** **E** **P** **B** **PM** **D** **M** |
|  | ptar1_human | 2 | [**1623**](http://cpdb.molgen.mpg.de/CPDB/showSetDetails?sp=n&st=294) | [**35 (2.2%)**](http://cpdb.molgen.mpg.de/CPDB/showSetDetails?sp=n&st=294) | 0.000388 | 0.0173 | **M** **P** **H** **R** **D** **BP** **I** **I** **H** **C** **S** **P** **EB** **P** **K** **P** **I** **M** |
|  | SKIV2L | 2 | [**2362**](http://cpdb.molgen.mpg.de/CPDB/showSetDetails?sp=n&st=295) | [**46 (2.0%)**](http://cpdb.molgen.mpg.de/CPDB/showSetDetails?sp=n&st=295) | 0.00039 | 0.0173 | **M** **P** **H** **D** **R** **NB** **I** **I** **H** **C** **S** **PE** **B** **P** **B** **P** **P** **M** |
|  | CHMP2A | 2 | [**3487**](http://cpdb.molgen.mpg.de/CPDB/showSetDetails?sp=n&st=296) | [**62 (1.8%)**](http://cpdb.molgen.mpg.de/CPDB/showSetDetails?sp=n&st=296) | 0.000391 | 0.0173 | **M** **P** **H** **R** **D** **NB** **I** **I** **H** **C** **B** **SP** **E** **B** **P** **K** **P** **IM** |
|  | GTF2H1 | 2 | [**3265**](http://cpdb.molgen.mpg.de/CPDB/showSetDetails?sp=n&st=297) | [**59 (1.8%)**](http://cpdb.molgen.mpg.de/CPDB/showSetDetails?sp=n&st=297) | 0.000392 | 0.0173 | **M** **P** **H** **D** **R** **NB** **P** **I** **I** **H** **C** **BS** **P** **E** **B** **P** **K** **PM** **P** **I** **M** **M** |
|  | ccd61_human | 2 | [**2985**](http://cpdb.molgen.mpg.de/CPDB/showSetDetails?sp=n&st=298) | [**55 (1.9%)**](http://cpdb.molgen.mpg.de/CPDB/showSetDetails?sp=n&st=298) | 0.000393 | 0.0173 | **M** **P** **H** **R** **D** **NB** **I** **I** **H** **C** **S** **PE** **B** **P** **B** **K** **P** **PI** **M** |
|  | SLCO4C1 | 1 | [**409**](http://cpdb.molgen.mpg.de/CPDB/showSetDetails?sp=n&st=299) | [**14 (3.4%)**](http://cpdb.molgen.mpg.de/CPDB/showSetDetails?sp=n&st=299) | 0.000395 | 0.149 | **R** **I** **B** |
|  | EWI2 | 2 | [**2292**](http://cpdb.molgen.mpg.de/CPDB/showSetDetails?sp=n&st=300) | [**45 (2.0%)**](http://cpdb.molgen.mpg.de/CPDB/showSetDetails?sp=n&st=300) | 0.000397 | 0.0174 | **M** **P** **H** **D** **R** **NB** **I** **I** **C** **B** **S** **PB** **P** **P** **M** **P** **I** **MM** |
|  | q9y573-2 | 2 | [**2223**](http://cpdb.molgen.mpg.de/CPDB/showSetDetails?sp=n&st=301) | [**44 (2.0%)**](http://cpdb.molgen.mpg.de/CPDB/showSetDetails?sp=n&st=301) | 0.000399 | 0.0174 | **M** **P** **H** **I** **R** **N** **BP** **I** **I** **H** **C** **B** **SP** **E** **B** **P** **P** **M** **PM** **D** |
|  | ITGB2 | 2 | [**4077**](http://cpdb.molgen.mpg.de/CPDB/showSetDetails?sp=n&st=302) | [**70 (1.7%)**](http://cpdb.molgen.mpg.de/CPDB/showSetDetails?sp=n&st=302) | 0.000403 | 0.0175 | **M** **P** **H** **R** **D** **NB** **I** **I** **H** **C** **B** **SP** **E** **B** **P** **K** **P** **MI** **M** **M** |
|  | FarC-PTP4A2 | 2 | [**2568**](http://cpdb.molgen.mpg.de/CPDB/showSetDetails?sp=n&st=303) | [**49 (1.9%)**](http://cpdb.molgen.mpg.de/CPDB/showSetDetails?sp=n&st=303) | 0.000404 | 0.0175 | **M** **P** **H** **D** **R** **NB** **P** **I** **I** **H** **C** **BS** **P** **E** **B** **P** **K** **PM** **I** **M** **M** |
|  | LAMP2 | 2 | [**3201**](http://cpdb.molgen.mpg.de/CPDB/showSetDetails?sp=n&st=304) | [**58 (1.8%)**](http://cpdb.molgen.mpg.de/CPDB/showSetDetails?sp=n&st=304) | 0.000407 | 0.0176 | **M** **P** **H** **D** **R** **NB** **I** **I** **H** **C** **B** **SP** **E** **B** **P** **K** **P** **MP** **I** **M** **M** |
|  | RAP2A | 2 | [**1627**](http://cpdb.molgen.mpg.de/CPDB/showSetDetails?sp=n&st=305) | [**35 (2.2%)**](http://cpdb.molgen.mpg.de/CPDB/showSetDetails?sp=n&st=305) | 0.000411 | 0.0176 | **M** **P** **H** **D** **R** **NB** **P** **I** **I** **H** **C** **BS** **P** **E** **B** **P** **K** **PI** **M** **M** |
|  | SUSD5 | 2 | [**187**](http://cpdb.molgen.mpg.de/CPDB/showSetDetails?sp=n&st=306) | [**9 (4.8%)**](http://cpdb.molgen.mpg.de/CPDB/showSetDetails?sp=n&st=306) | 0.000412 | 0.0176 | **H** **S** **M** **R** **I** **I** **B** |
|  | ERGIC1 | 2 | [**3066**](http://cpdb.molgen.mpg.de/CPDB/showSetDetails?sp=n&st=307) | [**56 (1.8%)**](http://cpdb.molgen.mpg.de/CPDB/showSetDetails?sp=n&st=307) | 0.000414 | 0.0176 | **M** **P** **H** **R** **D** **NB** **I** **I** **H** **C** **B** **SP** **E** **B** **P** **K** **P** **MP** **I** **M** **M** |
|  | MIR29A | 2 | [**410**](http://cpdb.molgen.mpg.de/CPDB/showSetDetails?sp=n&st=308) | [**14 (3.4%)**](http://cpdb.molgen.mpg.de/CPDB/showSetDetails?sp=n&st=308) | 0.000415 | 0.0176 | **P** **H** **R** **D** **N** **B** **II** **C** **S** **P** **E** **B** **PB** **P** **M** **M** **M** |
|  | LTBP4 | 2 | [**1256**](http://cpdb.molgen.mpg.de/CPDB/showSetDetails?sp=n&st=309) | [**29 (2.3%)**](http://cpdb.molgen.mpg.de/CPDB/showSetDetails?sp=n&st=309) | 0.000416 | 0.0176 | **M** **P** **H** **D** **R** **NB** **I** **I** **C** **B** **S** **PB** **P** **P** **M** **P** **I** **MM** |
|  | sp110_human | 2 | [**2925**](http://cpdb.molgen.mpg.de/CPDB/showSetDetails?sp=n&st=310) | [**54 (1.9%)**](http://cpdb.molgen.mpg.de/CPDB/showSetDetails?sp=n&st=310) | 0.000418 | 0.0176 | **M** **P** **H** **D** **R** **NB** **I** **I** **H** **C** **S** **PE** **B** **P** **B** **K** **P** **PI** **M** |
|  | mto1_human | 2 | [**1316**](http://cpdb.molgen.mpg.de/CPDB/showSetDetails?sp=n&st=311) | [**30 (2.3%)**](http://cpdb.molgen.mpg.de/CPDB/showSetDetails?sp=n&st=311) | 0.000419 | 0.0176 | **H** **R** **D** **B** **I** **I** **HC** **S** **P** **E** **B** **P** **BK** **I** **M** |
|  | TNFSF4 | 1 | [**3**](http://cpdb.molgen.mpg.de/CPDB/showSetDetails?sp=n&st=312) | [**2 (66.7%)**](http://cpdb.molgen.mpg.de/CPDB/showSetDetails?sp=n&st=312) | 0.000419 | 0.149 | **H** **S** **P** **R** **D** **I** |
|  | gog8a_human | 1 | [**3**](http://cpdb.molgen.mpg.de/CPDB/showSetDetails?sp=n&st=313) | [**2 (66.7%)**](http://cpdb.molgen.mpg.de/CPDB/showSetDetails?sp=n&st=313) | 0.000419 | 0.149 | **I** |
|  | PARD6B | 2 | [**3348**](http://cpdb.molgen.mpg.de/CPDB/showSetDetails?sp=n&st=314) | [**60 (1.8%)**](http://cpdb.molgen.mpg.de/CPDB/showSetDetails?sp=n&st=314) | 0.00042 | 0.0176 | **M** **P** **H** **I** **R** **N** **BP** **I** **I** **H** **C** **B** **SP** **E** **B** **P** **K** **P** **PM** **D** |
|  | nrbp2_human | 2 | [**1502**](http://cpdb.molgen.mpg.de/CPDB/showSetDetails?sp=n&st=315) | [**33 (2.2%)**](http://cpdb.molgen.mpg.de/CPDB/showSetDetails?sp=n&st=315) | 0.000422 | 0.0177 | **M** **P** **H** **R** **D** **NB** **P** **I** **I** **H** **C** **BS** **E** **B** **P** **K** **P** **IM** |
|  | LPL | 2 | [**1760**](http://cpdb.molgen.mpg.de/CPDB/showSetDetails?sp=n&st=316) | [**37 (2.1%)**](http://cpdb.molgen.mpg.de/CPDB/showSetDetails?sp=n&st=316) | 0.000425 | 0.0177 | **M** **P** **H** **D** **R** **NB** **P** **I** **I** **H** **C** **BS** **P** **E** **B** **P** **K** **PM** **I** **M** |
|  | NAPG | 2 | [**620**](http://cpdb.molgen.mpg.de/CPDB/showSetDetails?sp=n&st=317) | [**18 (2.9%)**](http://cpdb.molgen.mpg.de/CPDB/showSetDetails?sp=n&st=317) | 0.000427 | 0.0177 | **C** **H** **B** **S** **D** **E** **RK** **P** **B** **M** **I** **I** |
|  | KDM6B Gene | 2 | [**899**](http://cpdb.molgen.mpg.de/CPDB/showSetDetails?sp=n&st=318) | [**23 (2.6%)**](http://cpdb.molgen.mpg.de/CPDB/showSetDetails?sp=n&st=318) | 0.000427 | 0.0177 | **P** **H** **R** **D** **N** **B** **II** **H** **C** **B** **S** **P** **BP** **K** **P** **P** **I** **M** |
|  | PHKB | 2 | [**953**](http://cpdb.molgen.mpg.de/CPDB/showSetDetails?sp=n&st=319) | [**24 (2.5%)**](http://cpdb.molgen.mpg.de/CPDB/showSetDetails?sp=n&st=319) | 0.000428 | 0.0177 | **M** **P** **H** **D** **R** **NB** **I** **I** **H** **C** **B** **SE** **B** **P** **K** **P** **I** **M** |
|  | SERPINA3 | 2 | [**3717**](http://cpdb.molgen.mpg.de/CPDB/showSetDetails?sp=n&st=320) | [**65 (1.8%)**](http://cpdb.molgen.mpg.de/CPDB/showSetDetails?sp=n&st=320) | 0.00043 | 0.0177 | **M** **P** **H** **D** **R** **NB** **I** **I** **H** **C** **B** **SP** **E** **B** **P** **K** **P** **MP** **I** **M** **M** |
|  | fprp_human | 2 | [**901**](http://cpdb.molgen.mpg.de/CPDB/showSetDetails?sp=n&st=321) | [**23 (2.6%)**](http://cpdb.molgen.mpg.de/CPDB/showSetDetails?sp=n&st=321) | 0.000434 | 0.0178 | **M** **P** **H** **D** **R** **NB** **I** **I** **C** **B** **S** **PB** **P** **K** **P** **M** **I** **M** |
|  | RAB25 | 1 | [**82**](http://cpdb.molgen.mpg.de/CPDB/showSetDetails?sp=n&st=322) | [**6 (7.3%)**](http://cpdb.molgen.mpg.de/CPDB/showSetDetails?sp=n&st=322) | 0.000436 | 0.149 | **H** **S** **P** **R** **B** **P** **BI** |
|  | TIMP1 | 2 | [**2094**](http://cpdb.molgen.mpg.de/CPDB/showSetDetails?sp=n&st=323) | [**42 (2.0%)**](http://cpdb.molgen.mpg.de/CPDB/showSetDetails?sp=n&st=323) | 0.000436 | 0.0179 | **M** **P** **H** **D** **R** **NB** **I** **I** **H** **C** **B** **SP** **B** **P** **P** **I** **M** **M** |
|  | RIT2 | 2 | [**672**](http://cpdb.molgen.mpg.de/CPDB/showSetDetails?sp=n&st=324) | [**19 (2.8%)**](http://cpdb.molgen.mpg.de/CPDB/showSetDetails?sp=n&st=324) | 0.000444 | 0.0181 | **M** **P** **H** **I** **R** **N** **BP** **I** **I** **C** **S** **P** **E** **BP** **B** **P** **M** **D** |
|  | Cleaved fibrillin-1 | 2 | [**366**](http://cpdb.molgen.mpg.de/CPDB/showSetDetails?sp=n&st=325) | [**13 (3.6%)**](http://cpdb.molgen.mpg.de/CPDB/showSetDetails?sp=n&st=325) | 0.000448 | 0.0182 | **M** **P** **H** **R** **D** **NB** **I** **I** **C** **B** **S** **PB** **P** **P** **M** **M** |
|  | class I MHC B13 | 2 | [**1200**](http://cpdb.molgen.mpg.de/CPDB/showSetDetails?sp=n&st=326) | [**28 (2.3%)**](http://cpdb.molgen.mpg.de/CPDB/showSetDetails?sp=n&st=326) | 0.00045 | 0.0182 | **M** **P** **H** **I** **R** **N** **BI** **I** **C** **S** **P** **E** **B** **PB** **K** **P** **P** **M** **D** |
|  | MMP19 | 2 | [**518**](http://cpdb.molgen.mpg.de/CPDB/showSetDetails?sp=n&st=327) | [**16 (3.1%)**](http://cpdb.molgen.mpg.de/CPDB/showSetDetails?sp=n&st=327) | 0.000452 | 0.0183 | **M** **P** **H** **D** **R** **NB** **I** **I** **C** **B** **S** **PB** **P** **P** **M** **M** |
|  | NUMB | 1 | [**900**](http://cpdb.molgen.mpg.de/CPDB/showSetDetails?sp=n&st=328) | [**23 (2.6%)**](http://cpdb.molgen.mpg.de/CPDB/showSetDetails?sp=n&st=328) | 0.000454 | 0.149 | **P** **C** **H** **B** **S** **D** **RN** **P** **P** **B** **I** **M** **I** **I** |
|  | SYT1 | 2 | [**3071**](http://cpdb.molgen.mpg.de/CPDB/showSetDetails?sp=n&st=329) | [**56 (1.8%)**](http://cpdb.molgen.mpg.de/CPDB/showSetDetails?sp=n&st=329) | 0.000458 | 0.0184 | **M** **P** **H** **R** **D** **NB** **P** **I** **I** **H** **C** **BS** **P** **E** **B** **P** **K** **PM** **I** **M** **M** |
|  | H2AFJ | 2 | [**3286**](http://cpdb.molgen.mpg.de/CPDB/showSetDetails?sp=n&st=330) | [**59 (1.8%)**](http://cpdb.molgen.mpg.de/CPDB/showSetDetails?sp=n&st=330) | 0.000458 | 0.0184 | **M** **P** **H** **D** **R** **NB** **I** **I** **H** **C** **B** **SP** **E** **B** **P** **K** **P** **MP** **I** **M** **M** |
|  | Ca(2+)/calmodulin-dependent protein kinase phosphatase N | 2 | [**2855**](http://cpdb.molgen.mpg.de/CPDB/showSetDetails?sp=n&st=331) | [**53 (1.9%)**](http://cpdb.molgen.mpg.de/CPDB/showSetDetails?sp=n&st=331) | 0.000464 | 0.0185 | **M** **P** **H** **D** **R** **NB** **P** **I** **I** **H** **C** **BS** **P** **E** **B** **P** **P** **MP** **I** **M** |
|  | SCARB2 | 2 | [**3869**](http://cpdb.molgen.mpg.de/CPDB/showSetDetails?sp=n&st=332) | [**67 (1.7%)**](http://cpdb.molgen.mpg.de/CPDB/showSetDetails?sp=n&st=332) | 0.000465 | 0.0185 | **M** **P** **H** **I** **R** **N** **BP** **I** **I** **H** **C** **S** **P** **EB** **P** **B** **K** **P** **MP** **D** **M** **D** **M** |
|  | TUBGCP5 | 2 | [**2235**](http://cpdb.molgen.mpg.de/CPDB/showSetDetails?sp=n&st=333) | [**44 (2.0%)**](http://cpdb.molgen.mpg.de/CPDB/showSetDetails?sp=n&st=333) | 0.00047 | 0.0187 | **M** **P** **H** **D** **R** **NB** **P** **I** **I** **H** **C** **SP** **E** **B** **P** **B** **K** **PP** **I** **M** |
|  | LDP-3 | 2 | [**4316**](http://cpdb.molgen.mpg.de/CPDB/showSetDetails?sp=n&st=334) | [**73 (1.7%)**](http://cpdb.molgen.mpg.de/CPDB/showSetDetails?sp=n&st=334) | 0.000472 | 0.0187 | **M** **P** **H** **I** **R** **N** **BP** **I** **I** **H** **C** **B** **SP** **E** **B** **P** **P** **M** **PM** **D** **M** |
|  | UBE2I-G92-SUMO3 | 2 | [**3292**](http://cpdb.molgen.mpg.de/CPDB/showSetDetails?sp=n&st=335) | [**59 (1.8%)**](http://cpdb.molgen.mpg.de/CPDB/showSetDetails?sp=n&st=335) | 0.000476 | 0.0187 | **M** **P** **H** **I** **R** **N** **BI** **I** **H** **C** **B** **S** **PE** **B** **P** **K** **P** **M** **PM** **D** |
|  | CNPY3 | 2 | [**1203**](http://cpdb.molgen.mpg.de/CPDB/showSetDetails?sp=n&st=336) | [**28 (2.3%)**](http://cpdb.molgen.mpg.de/CPDB/showSetDetails?sp=n&st=336) | 0.000487 | 0.0187 | **P** **H** **I** **R** **N** **B** **I** **IH** **C** **S** **P** **E** **B** **PB** **K** **P** **M** **D** **M** |
|  | ACOT1 | 2 | [**1780**](http://cpdb.molgen.mpg.de/CPDB/showSetDetails?sp=n&st=337) | [**37 (2.1%)**](http://cpdb.molgen.mpg.de/CPDB/showSetDetails?sp=n&st=337) | 0.000489 | 0.0187 | **P** **H** **D** **R** **N** **B** **II** **H** **C** **S** **P** **E** **BP** **B** **K** **P** **M** **I** **M** |
|  | class I MHC B47; class I MHC B18; class I MHC B54; class I MHC B78; class I MHC B82; class I MHC B46; class I MHC B37; class I MHC B81; HLA class I histocompatibility antigen, Cw-5 alpha chain precursor; HLA class I histocompatibility antigen, A-33 alpha chain; class I MHC B49; class I MHC B15; class I MHC B53; class I MHC B39; class I MHC B40; class I MHC B58; class I MHC B41; class I MHC B50; class I MHC B14; HLA class I histocompatibility antigen, A-26 alpha chain; HLA class I histocompatibility antigen, A-80 alpha chain; class I MHC B59; class I MHC B51; class I MHC B8; class I MHC B52; class I MHC B56; class I MHC B55; HLA class I histocompatibility antigen, A-25 alpha chain; HLA class I histocompatibility antigen, A-32 alpha chain; HLA class I histocompatibility antigen, A-36 alpha chain; HLA class I histocompatibility antigen, Cw-17 alpha chain; HLA class I histocompatibility antigen, Cw-18 alpha chain; HLA class I histocompatibility antigen, A-23 alpha chain; HLA class I histocompatibility antigen, Cw-16 alpha chain; HLA class I histocompatibility antigen, Cw-14 alpha chain; HLA class I histocompatibility antigen, Cw-1 alpha chain; HLA-B; HLA class I histocompatibility antigen, A-43 alpha chain; class I MHC B67 | 2 | [**1146**](http://cpdb.molgen.mpg.de/CPDB/showSetDetails?sp=n&st=338) | [**27 (2.4%)**](http://cpdb.molgen.mpg.de/CPDB/showSetDetails?sp=n&st=338) | 0.000492 | 0.0187 | **M** **P** **H** **I** **R** **N** **BI** **I** **S** **P** **E** **B** **P** **BK** **P** **M** **D** |
|  | DPPA4 gene | 2 | [**2312**](http://cpdb.molgen.mpg.de/CPDB/showSetDetails?sp=n&st=339) | [**45 (2.0%)**](http://cpdb.molgen.mpg.de/CPDB/showSetDetails?sp=n&st=339) | 0.000494 | 0.0187 | **M** **P** **H** **R** **D** **NB** **I** **I** **H** **C** **S** **PE** **B** **P** **B** **K** **P** **PI** **M** |
|  | PAPSS1 | 2 | [**3224**](http://cpdb.molgen.mpg.de/CPDB/showSetDetails?sp=n&st=340) | [**58 (1.8%)**](http://cpdb.molgen.mpg.de/CPDB/showSetDetails?sp=n&st=340) | 0.000494 | 0.0187 | **M** **P** **H** **D** **R** **NB** **I** **I** **H** **C** **S** **PE** **B** **P** **B** **K** **PM** **I** **M** **M** |
|  | kif5c_human | 2 | [**849**](http://cpdb.molgen.mpg.de/CPDB/showSetDetails?sp=n&st=341) | [**22 (2.6%)**](http://cpdb.molgen.mpg.de/CPDB/showSetDetails?sp=n&st=341) | 0.000495 | 0.0187 | **P** **H** **I** **R** **B** **P** **I** **IC** **S** **P** **B** **B** **PM** **D** |
|  | SLC35A5 | 2 | [**372**](http://cpdb.molgen.mpg.de/CPDB/showSetDetails?sp=n&st=342) | [**13 (3.5%)**](http://cpdb.molgen.mpg.de/CPDB/showSetDetails?sp=n&st=342) | 0.000496 | 0.0187 | **C** **H** **S** **P** **D** **E** **RB** **P** **K** **B** **M** **I** **I** |
|  | RAB32 | 1 | [**84**](http://cpdb.molgen.mpg.de/CPDB/showSetDetails?sp=n&st=343) | [**6 (7.1%)**](http://cpdb.molgen.mpg.de/CPDB/showSetDetails?sp=n&st=343) | 0.000497 | 0.149 | **B** **P** **R** **I** **H** |
|  | TRADD | 2 | [**3085**](http://cpdb.molgen.mpg.de/CPDB/showSetDetails?sp=n&st=344) | [**56 (1.8%)**](http://cpdb.molgen.mpg.de/CPDB/showSetDetails?sp=n&st=344) | 0.000497 | 0.0187 | **M** **P** **H** **I** **R** **N** **BP** **I** **I** **H** **C** **B** **SP** **E** **B** **P** **K** **P** **MP** **M** **D** **M** |
|  | CYR61 | 2 | [**1456**](http://cpdb.molgen.mpg.de/CPDB/showSetDetails?sp=n&st=345) | [**32 (2.2%)**](http://cpdb.molgen.mpg.de/CPDB/showSetDetails?sp=n&st=345) | 0.000498 | 0.0187 | **M** **P** **H** **D** **R** **NB** **I** **I** **H** **C** **S** **PB** **P** **B** **P** **M** **PM** **M** |
|  | Factor V precursor | 2 | [**2310**](http://cpdb.molgen.mpg.de/CPDB/showSetDetails?sp=n&st=346) | [**45 (2.0%)**](http://cpdb.molgen.mpg.de/CPDB/showSetDetails?sp=n&st=346) | 0.000499 | 0.0187 | **M** **P** **H** **I** **R** **N** **BI** **I** **H** **C** **B** **S** **PB** **P** **P** **M** **D** **M** |
|  | Ig lambda chain V-I region HA | 2 | [**1913**](http://cpdb.molgen.mpg.de/CPDB/showSetDetails?sp=n&st=347) | [**39 (2.0%)**](http://cpdb.molgen.mpg.de/CPDB/showSetDetails?sp=n&st=347) | 0.000504 | 0.0187 | **M** **P** **H** **D** **R** **ND** **B** **I** **I** **H** **C** **SP** **E** **B** **P** **B** **K** **PM** **P** **I** **M** **M** |
|  | PP2C-eta | 2 | [**2655**](http://cpdb.molgen.mpg.de/CPDB/showSetDetails?sp=n&st=348) | [**50 (1.9%)**](http://cpdb.molgen.mpg.de/CPDB/showSetDetails?sp=n&st=348) | 0.000504 | 0.0187 | **M** **P** **H** **D** **R** **NB** **P** **I** **I** **H** **C** **BS** **P** **E** **B** **P** **P** **MP** **I** **M** |
|  | IL8 | 2 | [**3081**](http://cpdb.molgen.mpg.de/CPDB/showSetDetails?sp=n&st=349) | [**56 (1.8%)**](http://cpdb.molgen.mpg.de/CPDB/showSetDetails?sp=n&st=349) | 0.000509 | 0.0187 | **M** **P** **H** **R** **D** **NB** **P** **I** **I** **H** **C** **BS** **P** **E** **B** **P** **K** **PM** **I** **M** **M** |
|  | Hypothetical protein MGC44903 | 2 | [**573**](http://cpdb.molgen.mpg.de/CPDB/showSetDetails?sp=n&st=350) | [**17 (3.0%)**](http://cpdb.molgen.mpg.de/CPDB/showSetDetails?sp=n&st=350) | 0.000509 | 0.0187 | **M** **P** **H** **D** **R** **NB** **I** **I** **C** **S** **P** **BP** **B** **P** **P** **I** **M** |
|  | PARG | 2 | [**323**](http://cpdb.molgen.mpg.de/CPDB/showSetDetails?sp=n&st=351) | [**12 (3.7%)**](http://cpdb.molgen.mpg.de/CPDB/showSetDetails?sp=n&st=351) | 0.00051 | 0.0187 | **M** **P** **H** **D** **R** **NB** **I** **I** **C** **B** **S** **BP** **P** **P** **I** **M** |
|  | PDCD4 | 2 | [**2046**](http://cpdb.molgen.mpg.de/CPDB/showSetDetails?sp=n&st=352) | [**41 (2.0%)**](http://cpdb.molgen.mpg.de/CPDB/showSetDetails?sp=n&st=352) | 0.000511 | 0.0187 | **M** **P** **H** **I** **R** **N** **BI** **I** **H** **C** **B** **S** **PE** **B** **P** **K** **P** **M** **PM** **D** |
|  | HIST1H2AC | 2 | [**3738**](http://cpdb.molgen.mpg.de/CPDB/showSetDetails?sp=n&st=353) | [**65 (1.7%)**](http://cpdb.molgen.mpg.de/CPDB/showSetDetails?sp=n&st=353) | 0.000511 | 0.0187 | **M** **P** **H** **D** **R** **NB** **I** **I** **H** **C** **B** **SP** **E** **B** **P** **K** **P** **MP** **I** **M** **M** |
|  | COL4A5(27-?) | 2 | [**4035**](http://cpdb.molgen.mpg.de/CPDB/showSetDetails?sp=n&st=354) | [**69 (1.7%)**](http://cpdb.molgen.mpg.de/CPDB/showSetDetails?sp=n&st=354) | 0.000513 | 0.0187 | **M** **P** **H** **I** **R** **N** **BI** **I** **H** **C** **B** **S** **PE** **B** **P** **K** **P** **M** **PM** **D** **M** |
|  | MLLT3 | 2 | [**2876**](http://cpdb.molgen.mpg.de/CPDB/showSetDetails?sp=n&st=355) | [**53 (1.9%)**](http://cpdb.molgen.mpg.de/CPDB/showSetDetails?sp=n&st=355) | 0.000514 | 0.0187 | **M** **P** **H** **D** **R** **NB** **P** **I** **I** **C** **B** **SP** **E** **B** **P** **K** **P** **PI** **M** **M** |
|  | TMX3 | 2 | [**1918**](http://cpdb.molgen.mpg.de/CPDB/showSetDetails?sp=n&st=356) | [**39 (2.0%)**](http://cpdb.molgen.mpg.de/CPDB/showSetDetails?sp=n&st=356) | 0.000514 | 0.0187 | **M** **P** **H** **D** **R** **NB** **I** **I** **H** **C** **S** **PE** **B** **P** **B** **K** **PM** **I** **M** **M** |
|  | SMAD7 | 2 | [**2806**](http://cpdb.molgen.mpg.de/CPDB/showSetDetails?sp=n&st=357) | [**52 (1.9%)**](http://cpdb.molgen.mpg.de/CPDB/showSetDetails?sp=n&st=357) | 0.000515 | 0.0187 | **M** **P** **H** **D** **R** **NB** **P** **I** **I** **H** **C** **SP** **E** **B** **P** **B** **K** **PM** **P** **I** **M** **M** |
|  | VTI1B | 2 | [**4177**](http://cpdb.molgen.mpg.de/CPDB/showSetDetails?sp=n&st=358) | [**71 (1.7%)**](http://cpdb.molgen.mpg.de/CPDB/showSetDetails?sp=n&st=358) | 0.000515 | 0.0187 | **M** **P** **H** **I** **R** **N** **BI** **I** **H** **C** **B** **S** **PE** **B** **P** **K** **P** **MM** **D** **M** |
|  | sil1_human | 2 | [**1589**](http://cpdb.molgen.mpg.de/CPDB/showSetDetails?sp=n&st=359) | [**34 (2.2%)**](http://cpdb.molgen.mpg.de/CPDB/showSetDetails?sp=n&st=359) | 0.000515 | 0.0187 | **M** **P** **H** **D** **R** **NB** **P** **I** **I** **H** **C** **SP** **E** **B** **P** **B** **K** **PI** **M** |
|  | mydgf_human | 2 | [**1401**](http://cpdb.molgen.mpg.de/CPDB/showSetDetails?sp=n&st=360) | [**31 (2.2%)**](http://cpdb.molgen.mpg.de/CPDB/showSetDetails?sp=n&st=360) | 0.000516 | 0.0187 | **M** **P** **H** **D** **R** **B** **II** **C** **S** **P** **E** **B** **PB** **K** **P** **M** **M** **M** |
|  | magc1_human | 2 | [**279**](http://cpdb.molgen.mpg.de/CPDB/showSetDetails?sp=n&st=361) | [**11 (4.0%)**](http://cpdb.molgen.mpg.de/CPDB/showSetDetails?sp=n&st=361) | 0.000517 | 0.0187 | **C** **H** **B** **S** **P** **D** **ER** **B** **M** **I** **H** |
|  | MME | 2 | [**3020**](http://cpdb.molgen.mpg.de/CPDB/showSetDetails?sp=n&st=362) | [**55 (1.8%)**](http://cpdb.molgen.mpg.de/CPDB/showSetDetails?sp=n&st=362) | 0.000518 | 0.0187 | **M** **P** **H** **D** **R** **NB** **P** **I** **I** **H** **C** **BS** **P** **E** **B** **P** **K** **PM** **I** **M** **M** |
|  | plrkt_human | 2 | [**1272**](http://cpdb.molgen.mpg.de/CPDB/showSetDetails?sp=n&st=363) | [**29 (2.3%)**](http://cpdb.molgen.mpg.de/CPDB/showSetDetails?sp=n&st=363) | 0.000518 | 0.0187 | **P** **H** **I** **R** **N** **B** **PI** **I** **C** **S** **E** **B** **PB** **K** **P** **M** **M** **DM** |
|  | (RefSeq) fucosyltransferase 4 | 2 | [**577**](http://cpdb.molgen.mpg.de/CPDB/showSetDetails?sp=n&st=364) | [**17 (3.0%)**](http://cpdb.molgen.mpg.de/CPDB/showSetDetails?sp=n&st=364) | 0.00052 | 0.0187 | **P** **H** **R** **D** **N** **B** **II** **H** **C** **B** **S** **P** **EB** **P** **K** **P** **M** **M** |
|  | NAGLU(59-743) | 2 | [**682**](http://cpdb.molgen.mpg.de/CPDB/showSetDetails?sp=n&st=365) | [**19 (2.8%)**](http://cpdb.molgen.mpg.de/CPDB/showSetDetails?sp=n&st=365) | 0.000522 | 0.0187 | **C** **H** **B** **S** **P** **R** **ED** **P** **K** **P** **B** **M** **II** **H** **M** |
|  | PDPR | 2 | [**371**](http://cpdb.molgen.mpg.de/CPDB/showSetDetails?sp=n&st=366) | [**13 (3.5%)**](http://cpdb.molgen.mpg.de/CPDB/showSetDetails?sp=n&st=366) | 0.000522 | 0.0187 | **P** **C** **H** **S** **D** **E** **RP** **K** **P** **B** **I** **M** **I** **IH** |
|  | DUSP9 | 2 | [**4184**](http://cpdb.molgen.mpg.de/CPDB/showSetDetails?sp=n&st=367) | [**71 (1.7%)**](http://cpdb.molgen.mpg.de/CPDB/showSetDetails?sp=n&st=367) | 0.000523 | 0.0187 | **M** **P** **H** **I** **R** **N** **BP** **I** **I** **H** **C** **B** **SP** **E** **B** **P** **K** **P** **MP** **M** **D** **M** |
|  | MCM10 | 2 | [**2871**](http://cpdb.molgen.mpg.de/CPDB/showSetDetails?sp=n&st=368) | [**53 (1.9%)**](http://cpdb.molgen.mpg.de/CPDB/showSetDetails?sp=n&st=368) | 0.000523 | 0.0187 | **M** **P** **H** **R** **D** **NB** **P** **I** **I** **C** **B** **SP** **E** **B** **P** **P** **M** **PI** **M** |
|  | HRG | 1 | [**85**](http://cpdb.molgen.mpg.de/CPDB/showSetDetails?sp=n&st=369) | [**6 (7.1%)**](http://cpdb.molgen.mpg.de/CPDB/showSetDetails?sp=n&st=369) | 0.000529 | 0.149 | **H** **R** **I** **I** **B** |
|  | VPS72 | 2 | [**2734**](http://cpdb.molgen.mpg.de/CPDB/showSetDetails?sp=n&st=370) | [**51 (1.9%)**](http://cpdb.molgen.mpg.de/CPDB/showSetDetails?sp=n&st=370) | 0.000533 | 0.019 | **M** **P** **H** **R** **D** **NB** **I** **I** **H** **C** **B** **SP** **E** **B** **P** **K** **P** **PI** **M** |
|  | snx16_human | 2 | [**795**](http://cpdb.molgen.mpg.de/CPDB/showSetDetails?sp=n&st=371) | [**21 (2.6%)**](http://cpdb.molgen.mpg.de/CPDB/showSetDetails?sp=n&st=371) | 0.000535 | 0.019 | **H** **R** **D** **B** **I** **I** **HC** **S** **P** **E** **B** **P** **BK** **I** **M** |
|  | mic25_human | 2 | [**2740**](http://cpdb.molgen.mpg.de/CPDB/showSetDetails?sp=n&st=372) | [**51 (1.9%)**](http://cpdb.molgen.mpg.de/CPDB/showSetDetails?sp=n&st=372) | 0.000537 | 0.019 | **M** **P** **H** **I** **R** **N** **BP** **I** **I** **H** **C** **S** **P** **EB** **P** **K** **P** **M** **MD** **M** |
|  | h0yln8_human | 2 | [**2461**](http://cpdb.molgen.mpg.de/CPDB/showSetDetails?sp=n&st=373) | [**47 (1.9%)**](http://cpdb.molgen.mpg.de/CPDB/showSetDetails?sp=n&st=373) | 0.000538 | 0.019 | **M** **P** **H** **D** **R** **NB** **P** **I** **I** **H** **C** **SP** **E** **B** **P** **B** **K** **PP** **I** **M** |
|  | lyst_human | 2 | [**2950**](http://cpdb.molgen.mpg.de/CPDB/showSetDetails?sp=n&st=374) | [**54 (1.8%)**](http://cpdb.molgen.mpg.de/CPDB/showSetDetails?sp=n&st=374) | 0.000538 | 0.019 | **M** **P** **H** **D** **R** **NB** **I** **I** **H** **C** **S** **PE** **B** **P** **B** **K** **P** **PI** **M** |
|  | TRAPPC6B | 2 | [**1150**](http://cpdb.molgen.mpg.de/CPDB/showSetDetails?sp=n&st=375) | [**27 (2.4%)**](http://cpdb.molgen.mpg.de/CPDB/showSetDetails?sp=n&st=375) | 0.000548 | 0.0193 | **M** **P** **H** **R** **D** **NB** **I** **I** **H** **C** **B** **SP** **B** **P** **P** **M** |
|  | mica3_human | 2 | [**2671**](http://cpdb.molgen.mpg.de/CPDB/showSetDetails?sp=n&st=376) | [**50 (1.9%)**](http://cpdb.molgen.mpg.de/CPDB/showSetDetails?sp=n&st=376) | 0.00055 | 0.0193 | **M** **P** **H** **D** **R** **NB** **P** **I** **I** **H** **C** **SP** **E** **B** **P** **B** **K** **PM** **P** **I** **M** **M** |
|  | TBC1D22B | 1 | [**577**](http://cpdb.molgen.mpg.de/CPDB/showSetDetails?sp=n&st=377) | [**17 (3.0%)**](http://cpdb.molgen.mpg.de/CPDB/showSetDetails?sp=n&st=377) | 0.000551 | 0.149 | **I** **B** **I** |
|  | class I MHC B57 | 2 | [**1156**](http://cpdb.molgen.mpg.de/CPDB/showSetDetails?sp=n&st=378) | [**27 (2.3%)**](http://cpdb.molgen.mpg.de/CPDB/showSetDetails?sp=n&st=378) | 0.000562 | 0.0195 | **M** **P** **H** **I** **R** **N** **BI** **I** **S** **P** **E** **B** **P** **BK** **P** **M** **D** |
|  | d6ra89_human | 2 | [**1857**](http://cpdb.molgen.mpg.de/CPDB/showSetDetails?sp=n&st=379) | [**38 (2.1%)**](http://cpdb.molgen.mpg.de/CPDB/showSetDetails?sp=n&st=379) | 0.000564 | 0.0195 | **M** **P** **H** **D** **R** **NB** **I** **I** **H** **C** **B** **SP** **E** **B** **P** **P** **P** **M** |
|  | RARA | 2 | [**4573**](http://cpdb.molgen.mpg.de/CPDB/showSetDetails?sp=n&st=380) | [**76 (1.7%)**](http://cpdb.molgen.mpg.de/CPDB/showSetDetails?sp=n&st=380) | 0.000565 | 0.0195 | **M** **P** **H** **D** **R** **NB** **P** **I** **I** **H** **C** **BS** **P** **E** **B** **P** **K** **PM** **P** **I** **M** |
|  | SELP | 2 | [**1342**](http://cpdb.molgen.mpg.de/CPDB/showSetDetails?sp=n&st=381) | [**30 (2.2%)**](http://cpdb.molgen.mpg.de/CPDB/showSetDetails?sp=n&st=381) | 0.000565 | 0.0195 | **M** **P** **H** **I** **R** **N** **BI** **I** **H** **C** **B** **S** **PE** **B** **P** **K** **P** **MM** **D** **M** |
|  | GLI3 | 2 | [**2816**](http://cpdb.molgen.mpg.de/CPDB/showSetDetails?sp=n&st=382) | [**52 (1.9%)**](http://cpdb.molgen.mpg.de/CPDB/showSetDetails?sp=n&st=382) | 0.000565 | 0.0195 | **M** **P** **H** **I** **R** **N** **BP** **I** **I** **H** **C** **S** **P** **EB** **P** **B** **K** **P** **MP** **M** **D** **M** |
|  | SOX4 | 2 | [**1279**](http://cpdb.molgen.mpg.de/CPDB/showSetDetails?sp=n&st=383) | [**29 (2.3%)**](http://cpdb.molgen.mpg.de/CPDB/showSetDetails?sp=n&st=383) | 0.000566 | 0.0195 | **M** **P** **H** **I** **R** **N** **BP** **I** **I** **C** **S** **P** **B** **PB** **P** **P** **M** **D** |
|  | PLG(20-810) | 2 | [**3173**](http://cpdb.molgen.mpg.de/CPDB/showSetDetails?sp=n&st=384) | [**57 (1.8%)**](http://cpdb.molgen.mpg.de/CPDB/showSetDetails?sp=n&st=384) | 0.000569 | 0.0196 | **M** **P** **H** **D** **R** **NB** **I** **I** **H** **C** **B** **SP** **E** **B** **P** **P** **M** **PI** **M** **M** |
|  | SUMF2 | 2 | [**1037**](http://cpdb.molgen.mpg.de/CPDB/showSetDetails?sp=n&st=385) | [**25 (2.4%)**](http://cpdb.molgen.mpg.de/CPDB/showSetDetails?sp=n&st=385) | 0.000573 | 0.0196 | **M** **P** **H** **R** **D** **NB** **I** **I** **H** **C** **S** **PE** **B** **P** **B** **K** **P** **IM** **M** |
|  | SHCBP1 | 2 | [**1791**](http://cpdb.molgen.mpg.de/CPDB/showSetDetails?sp=n&st=386) | [**37 (2.1%)**](http://cpdb.molgen.mpg.de/CPDB/showSetDetails?sp=n&st=386) | 0.000573 | 0.0196 | **M** **P** **H** **R** **D** **NB** **P** **I** **I** **C** **B** **SP** **E** **B** **P** **P** **M** **PI** **M** |
|  | RAB44 | 1 | [**196**](http://cpdb.molgen.mpg.de/CPDB/showSetDetails?sp=n&st=387) | [**9 (4.6%)**](http://cpdb.molgen.mpg.de/CPDB/showSetDetails?sp=n&st=387) | 0.00058 | 0.149 | **R** |
|  | RAB40AL | 2 | [**198**](http://cpdb.molgen.mpg.de/CPDB/showSetDetails?sp=n&st=388) | [**9 (4.6%)**](http://cpdb.molgen.mpg.de/CPDB/showSetDetails?sp=n&st=388) | 0.00058 | 0.0198 | **R** **H** **S** **P** **I** **D** **B** |
|  | kdel2_human | 2 | [**803**](http://cpdb.molgen.mpg.de/CPDB/showSetDetails?sp=n&st=389) | [**21 (2.6%)**](http://cpdb.molgen.mpg.de/CPDB/showSetDetails?sp=n&st=389) | 0.000581 | 0.0198 | **C** **H** **S** **P** **R** **E** **BB** **N** **P** **K** **B** **MD** **I** **I** **H** |
|  | MB21D1 | 2 | [**1283**](http://cpdb.molgen.mpg.de/CPDB/showSetDetails?sp=n&st=390) | [**29 (2.3%)**](http://cpdb.molgen.mpg.de/CPDB/showSetDetails?sp=n&st=390) | 0.000587 | 0.0199 | **H** **D** **R** **N** **B** **I** **IH** **C** **S** **P** **E** **B** **PK** **P** **P** **I** **M** |
|  | RAB42; RAB20 | 2 | [**920**](http://cpdb.molgen.mpg.de/CPDB/showSetDetails?sp=n&st=391) | [**23 (2.5%)**](http://cpdb.molgen.mpg.de/CPDB/showSetDetails?sp=n&st=391) | 0.000589 | 0.0199 | **M** **P** **H** **R** **D** **NB** **I** **I** **C** **B** **S** **PB** **P** **P** **M** |
|  | GDE1 | 2 | [**428**](http://cpdb.molgen.mpg.de/CPDB/showSetDetails?sp=n&st=392) | [**14 (3.3%)**](http://cpdb.molgen.mpg.de/CPDB/showSetDetails?sp=n&st=392) | 0.000591 | 0.0199 | **M** **P** **C** **H** **S** **PB** **R** **B** **P** **P** **BM** **D** **I** **I** |
|  | FBXO42 | 2 | [**2065**](http://cpdb.molgen.mpg.de/CPDB/showSetDetails?sp=n&st=393) | [**41 (2.0%)**](http://cpdb.molgen.mpg.de/CPDB/showSetDetails?sp=n&st=393) | 0.000592 | 0.0199 | **M** **P** **H** **R** **D** **NB** **I** **I** **H** **C** **B** **SP** **E** **B** **P** **K** **P** **MP** **I** **M** |
|  | h7c5q0_human | 2 | [**1471**](http://cpdb.molgen.mpg.de/CPDB/showSetDetails?sp=n&st=394) | [**32 (2.2%)**](http://cpdb.molgen.mpg.de/CPDB/showSetDetails?sp=n&st=394) | 0.000594 | 0.02 | **M** **P** **H** **D** **R** **BP** **I** **I** **C** **B** **S** **BP** **P** **M** **P** **M** |
|  | ALS2CL | 2 | [**285**](http://cpdb.molgen.mpg.de/CPDB/showSetDetails?sp=n&st=395) | [**11 (3.9%)**](http://cpdb.molgen.mpg.de/CPDB/showSetDetails?sp=n&st=395) | 0.000599 | 0.02 | **P** **C** **H** **B** **S** **P** **BR** **N** **P** **P** **B** **M** **I** |
|  | ART1 | 2 | [**283**](http://cpdb.molgen.mpg.de/CPDB/showSetDetails?sp=n&st=396) | [**11 (3.9%)**](http://cpdb.molgen.mpg.de/CPDB/showSetDetails?sp=n&st=396) | 0.000599 | 0.02 | **M** **P** **H** **D** **R** **NB** **I** **I** **H** **B** **S** **PE** **P** **P** **I** |
|  | TSPAN7 | 2 | [**977**](http://cpdb.molgen.mpg.de/CPDB/showSetDetails?sp=n&st=397) | [**24 (2.5%)**](http://cpdb.molgen.mpg.de/CPDB/showSetDetails?sp=n&st=397) | 0.0006 | 0.02 | **M** **P** **H** **D** **R** **NB** **P** **I** **I** **C** **S** **PB** **P** **B** **P** **M** **MM** |
|  | g5e9h3_human | 2 | [**1796**](http://cpdb.molgen.mpg.de/CPDB/showSetDetails?sp=n&st=398) | [**37 (2.1%)**](http://cpdb.molgen.mpg.de/CPDB/showSetDetails?sp=n&st=398) | 0.000611 | 0.0203 | **M** **P** **H** **R** **D** **NB** **I** **I** **C** **S** **P** **EB** **P** **B** **K** **P** **MP** **I** **M** |
|  | gse1_human | 2 | [**3186**](http://cpdb.molgen.mpg.de/CPDB/showSetDetails?sp=n&st=399) | [**57 (1.8%)**](http://cpdb.molgen.mpg.de/CPDB/showSetDetails?sp=n&st=399) | 0.000612 | 0.0203 | **M** **P** **H** **D** **R** **NB** **I** **I** **H** **C** **B** **SP** **E** **B** **P** **K** **P** **PI** **M** **M** |
|  | SEC24B | 1 | [**1925**](http://cpdb.molgen.mpg.de/CPDB/showSetDetails?sp=n&st=400) | [**39 (2.0%)**](http://cpdb.molgen.mpg.de/CPDB/showSetDetails?sp=n&st=400) | 0.000612 | 0.149 | **B** **S** **I** **R** **I** **H** |
|  | Striatin | 2 | [**3473**](http://cpdb.molgen.mpg.de/CPDB/showSetDetails?sp=n&st=401) | [**61 (1.8%)**](http://cpdb.molgen.mpg.de/CPDB/showSetDetails?sp=n&st=401) | 0.000615 | 0.0203 | **M** **P** **H** **D** **R** **NB** **I** **I** **H** **C** **S** **PE** **B** **P** **B** **K** **P** **PI** **M** **M** |
|  | UROD | 2 | [**481**](http://cpdb.molgen.mpg.de/CPDB/showSetDetails?sp=n&st=402) | [**15 (3.1%)**](http://cpdb.molgen.mpg.de/CPDB/showSetDetails?sp=n&st=402) | 0.000626 | 0.0206 | **H** **I** **R** **B** **I** **I** **H** **CS** **P** **E** **B** **P** **K** **PM** **D** |
|  | MRPL21 | 2 | [**1734**](http://cpdb.molgen.mpg.de/CPDB/showSetDetails?sp=n&st=403) | [**36 (2.1%)**](http://cpdb.molgen.mpg.de/CPDB/showSetDetails?sp=n&st=403) | 0.000626 | 0.0206 | **H** **I** **R** **N** **B** **I** **I** **HC** **S** **E** **B** **P** **B** **KP** **M** **D** |
|  | HLA class I histocompatibility antigen, A-74 alpha chain; HLA class I histocompatibility antigen, A-34 alpha chain | 2 | [**2549**](http://cpdb.molgen.mpg.de/CPDB/showSetDetails?sp=n&st=404) | [**48 (1.9%)**](http://cpdb.molgen.mpg.de/CPDB/showSetDetails?sp=n&st=404) | 0.000627 | 0.0206 | **M** **P** **H** **I** **R** **N** **BI** **I** **H** **C** **S** **P** **EB** **P** **B** **K** **P** **MM** **D** **M** |
|  | POU5F1 | 2 | [**4516**](http://cpdb.molgen.mpg.de/CPDB/showSetDetails?sp=n&st=405) | [**75 (1.7%)**](http://cpdb.molgen.mpg.de/CPDB/showSetDetails?sp=n&st=405) | 0.000637 | 0.0208 | **M** **P** **H** **R** **D** **NB** **I** **I** **H** **C** **S** **PE** **B** **P** **B** **K** **PM** **P** **I** **M** **M** |
|  | Ub-SMAD1 | 2 | [**4296**](http://cpdb.molgen.mpg.de/CPDB/showSetDetails?sp=n&st=406) | [**72 (1.7%)**](http://cpdb.molgen.mpg.de/CPDB/showSetDetails?sp=n&st=406) | 0.000639 | 0.0208 | **M** **P** **H** **D** **R** **NB** **P** **I** **I** **H** **C** **BS** **P** **E** **B** **P** **K** **PM** **P** **I** **M** **M** |
|  | CHMP5 | 2 | [**4215**](http://cpdb.molgen.mpg.de/CPDB/showSetDetails?sp=n&st=407) | [**71 (1.7%)**](http://cpdb.molgen.mpg.de/CPDB/showSetDetails?sp=n&st=407) | 0.000642 | 0.0209 | **M** **P** **H** **R** **D** **NB** **I** **I** **H** **C** **B** **SP** **E** **B** **P** **K** **P** **MP** **I** **M** |
|  | SLC9A6 | 2 | [**434**](http://cpdb.molgen.mpg.de/CPDB/showSetDetails?sp=n&st=408) | [**14 (3.3%)**](http://cpdb.molgen.mpg.de/CPDB/showSetDetails?sp=n&st=408) | 0.000648 | 0.021 | **P** **H** **R** **D** **B** **P** **II** **C** **S** **B** **P** **B** **PM** |
|  | k7ek07_human | 2 | [**3695**](http://cpdb.molgen.mpg.de/CPDB/showSetDetails?sp=n&st=409) | [**64 (1.7%)**](http://cpdb.molgen.mpg.de/CPDB/showSetDetails?sp=n&st=409) | 0.000651 | 0.021 | **M** **P** **H** **I** **R** **N** **BP** **I** **I** **H** **C** **B** **SP** **E** **B** **P** **K** **P** **PM** **D** |
|  | GPIN-CD14(20-345) | 2 | [**3701**](http://cpdb.molgen.mpg.de/CPDB/showSetDetails?sp=n&st=410) | [**64 (1.7%)**](http://cpdb.molgen.mpg.de/CPDB/showSetDetails?sp=n&st=410) | 0.000651 | 0.021 | **M** **P** **H** **I** **R** **N** **BI** **I** **H** **C** **B** **S** **PE** **B** **P** **K** **P** **M** **PM** **D** **M** |
|  | herp2_human | 2 | [**1478**](http://cpdb.molgen.mpg.de/CPDB/showSetDetails?sp=n&st=411) | [**32 (2.2%)**](http://cpdb.molgen.mpg.de/CPDB/showSetDetails?sp=n&st=411) | 0.000652 | 0.021 | **M** **P** **H** **I** **D** **N** **BP** **I** **I** **H** **C** **B** **SP** **E** **B** **R** **P** **K** **PM** **M** |
|  | SERPINF2 | 2 | [**1935**](http://cpdb.molgen.mpg.de/CPDB/showSetDetails?sp=n&st=412) | [**39 (2.0%)**](http://cpdb.molgen.mpg.de/CPDB/showSetDetails?sp=n&st=412) | 0.000663 | 0.0213 | **M** **P** **H** **D** **R** **NB** **I** **I** **H** **C** **B** **SP** **B** **P** **P** **I** **M** **M** |
|  | RAB23 | 1 | [**431**](http://cpdb.molgen.mpg.de/CPDB/showSetDetails?sp=n&st=413) | [**14 (3.3%)**](http://cpdb.molgen.mpg.de/CPDB/showSetDetails?sp=n&st=413) | 0.000663 | 0.149 | **P** **S** **I** **R** **N** **P** **BM** **I** |
|  | PLD4 | 2 | [**333**](http://cpdb.molgen.mpg.de/CPDB/showSetDetails?sp=n&st=414) | [**12 (3.6%)**](http://cpdb.molgen.mpg.de/CPDB/showSetDetails?sp=n&st=414) | 0.000667 | 0.0213 | **H** **S** **R** **E** **B** **P** **KB** **M** **I** **I** **H** |
|  | UBXN1 | 2 | [**3339**](http://cpdb.molgen.mpg.de/CPDB/showSetDetails?sp=n&st=415) | [**59 (1.8%)**](http://cpdb.molgen.mpg.de/CPDB/showSetDetails?sp=n&st=415) | 0.000668 | 0.0213 | **M** **P** **H** **I** **R** **N** **BI** **I** **H** **C** **B** **S** **PE** **B** **P** **K** **P** **M** **PM** **D** |
|  | PTPRC | 2 | [**3782**](http://cpdb.molgen.mpg.de/CPDB/showSetDetails?sp=n&st=416) | [**65 (1.7%)**](http://cpdb.molgen.mpg.de/CPDB/showSetDetails?sp=n&st=416) | 0.000668 | 0.0213 | **M** **P** **H** **I** **R** **N** **BP** **I** **I** **H** **C** **B** **SP** **E** **B** **P** **K** **P** **MM** **D** **M** |
|  | RAB37 | 1 | [**200**](http://cpdb.molgen.mpg.de/CPDB/showSetDetails?sp=n&st=417) | [**9 (4.5%)**](http://cpdb.molgen.mpg.de/CPDB/showSetDetails?sp=n&st=417) | 0.00067 | 0.149 | **H** **S** **M** **R** **I** **B** |
|  | DCP1A | 2 | [**2074**](http://cpdb.molgen.mpg.de/CPDB/showSetDetails?sp=n&st=418) | [**41 (2.0%)**](http://cpdb.molgen.mpg.de/CPDB/showSetDetails?sp=n&st=418) | 0.000672 | 0.0214 | **M** **P** **H** **D** **R** **NB** **I** **I** **C** **B** **S** **PE** **B** **P** **P** **P** **I** **M** |
|  | PLBD1 | 2 | [**1416**](http://cpdb.molgen.mpg.de/CPDB/showSetDetails?sp=n&st=419) | [**31 (2.2%)**](http://cpdb.molgen.mpg.de/CPDB/showSetDetails?sp=n&st=419) | 0.000678 | 0.0215 | **M** **P** **H** **I** **R** **N** **BI** **I** **H** **C** **S** **P** **EB** **P** **K** **P** **M** **MD** |
|  | nenf_human | 2 | [**1232**](http://cpdb.molgen.mpg.de/CPDB/showSetDetails?sp=n&st=420) | [**28 (2.3%)**](http://cpdb.molgen.mpg.de/CPDB/showSetDetails?sp=n&st=420) | 0.000681 | 0.0215 | **M** **P** **H** **D** **R** **NB** **I** **I** **H** **C** **S** **PE** **B** **P** **B** **K** **PM** **M** |
|  | MEGF8 | 2 | [**536**](http://cpdb.molgen.mpg.de/CPDB/showSetDetails?sp=n&st=421) | [**16 (3.0%)**](http://cpdb.molgen.mpg.de/CPDB/showSetDetails?sp=n&st=421) | 0.000682 | 0.0215 | **M** **P** **H** **D** **R** **NB** **I** **I** **C** **S** **E** **BP** **B** **P** **P** **M** **M** |
|  | LAMA2 | 2 | [**2838**](http://cpdb.molgen.mpg.de/CPDB/showSetDetails?sp=n&st=422) | [**52 (1.8%)**](http://cpdb.molgen.mpg.de/CPDB/showSetDetails?sp=n&st=422) | 0.000686 | 0.0216 | **M** **P** **H** **D** **R** **NB** **I** **I** **H** **C** **S** **PE** **B** **P** **B** **K** **PM** **P** **I** **M** **M** |
|  | vps16_human | 2 | [**1677**](http://cpdb.molgen.mpg.de/CPDB/showSetDetails?sp=n&st=423) | [**35 (2.1%)**](http://cpdb.molgen.mpg.de/CPDB/showSetDetails?sp=n&st=423) | 0.000698 | 0.0218 | **M** **P** **H** **D** **R** **NB** **I** **I** **H** **C** **S** **PE** **B** **P** **B** **K** **P** **IM** |
|  | BLOC1S1 | 2 | [**1228**](http://cpdb.molgen.mpg.de/CPDB/showSetDetails?sp=n&st=424) | [**28 (2.3%)**](http://cpdb.molgen.mpg.de/CPDB/showSetDetails?sp=n&st=424) | 0.000698 | 0.0218 | **C** **H** **S** **D** **B** **R** **PK** **P** **B** **M** **I** **I** |
|  | tarb1_human | 2 | [**3267**](http://cpdb.molgen.mpg.de/CPDB/showSetDetails?sp=n&st=425) | [**58 (1.8%)**](http://cpdb.molgen.mpg.de/CPDB/showSetDetails?sp=n&st=425) | 0.0007 | 0.0218 | **M** **P** **H** **R** **D** **NB** **P** **I** **I** **H** **C** **SP** **E** **B** **P** **B** **K** **PM** **P** **I** **M** |
|  | INPP5B(321-993) | 2 | [**4006**](http://cpdb.molgen.mpg.de/CPDB/showSetDetails?sp=n&st=426) | [**68 (1.7%)**](http://cpdb.molgen.mpg.de/CPDB/showSetDetails?sp=n&st=426) | 0.0007 | 0.0218 | **M** **P** **H** **D** **R** **NB** **P** **I** **I** **H** **C** **SP** **E** **B** **P** **B** **K** **PM** **P** **I** **M** |
|  | SCCPDH | 2 | [**2556**](http://cpdb.molgen.mpg.de/CPDB/showSetDetails?sp=n&st=427) | [**48 (1.9%)**](http://cpdb.molgen.mpg.de/CPDB/showSetDetails?sp=n&st=427) | 0.000702 | 0.0218 | **M** **P** **H** **D** **R** **NB** **I** **I** **H** **C** **B** **SP** **E** **B** **P** **K** **P** **MP** **I** **M** **M** |
|  | NDUFB5 | 2 | [**3415**](http://cpdb.molgen.mpg.de/CPDB/showSetDetails?sp=n&st=428) | [**60 (1.8%)**](http://cpdb.molgen.mpg.de/CPDB/showSetDetails?sp=n&st=428) | 0.000703 | 0.0218 | **M** **P** **H** **D** **R** **NB** **P** **I** **I** **H** **C** **SP** **E** **B** **P** **B** **K** **PM** **P** **I** **M** **M** |
|  | PLEKHA7 | 2 | [**2417**](http://cpdb.molgen.mpg.de/CPDB/showSetDetails?sp=n&st=429) | [**46 (1.9%)**](http://cpdb.molgen.mpg.de/CPDB/showSetDetails?sp=n&st=429) | 0.000704 | 0.0218 | **M** **P** **H** **D** **R** **NB** **P** **I** **I** **C** **B** **SP** **E** **B** **P** **P** **M** **PI** **M** |
|  | G6PD | 2 | [**2152**](http://cpdb.molgen.mpg.de/CPDB/showSetDetails?sp=n&st=430) | [**42 (2.0%)**](http://cpdb.molgen.mpg.de/CPDB/showSetDetails?sp=n&st=430) | 0.000709 | 0.0219 | **M** **P** **H** **I** **R** **N** **BP** **I** **I** **H** **C** **S** **P** **EB** **P** **B** **K** **P** **MP** **M** **D** **M** |
|  | COL15A1(28-1388) | 2 | [**1172**](http://cpdb.molgen.mpg.de/CPDB/showSetDetails?sp=n&st=431) | [**27 (2.3%)**](http://cpdb.molgen.mpg.de/CPDB/showSetDetails?sp=n&st=431) | 0.000712 | 0.0219 | **M** **P** **H** **D** **R** **NB** **I** **I** **C** **B** **S** **PE** **B** **P** **K** **P** **MM** |
|  | APOA1(25-267) | 1 | [**290**](http://cpdb.molgen.mpg.de/CPDB/showSetDetails?sp=n&st=432) | [**11 (3.8%)**](http://cpdb.molgen.mpg.de/CPDB/showSetDetails?sp=n&st=432) | 0.000712 | 0.149 | **H** **B** **S** **P** **D** **R** **PB** **M** **I** **I** **M** |
|  | ELP3 | 2 | [**3200**](http://cpdb.molgen.mpg.de/CPDB/showSetDetails?sp=n&st=433) | [**57 (1.8%)**](http://cpdb.molgen.mpg.de/CPDB/showSetDetails?sp=n&st=433) | 0.000716 | 0.0219 | **M** **P** **H** **R** **D** **NB** **I** **I** **H** **C** **B** **SP** **E** **B** **P** **K** **P** **PI** **M** |
|  | TIAM1 | 2 | [**4915**](http://cpdb.molgen.mpg.de/CPDB/showSetDetails?sp=n&st=434) | [**80 (1.6%)**](http://cpdb.molgen.mpg.de/CPDB/showSetDetails?sp=n&st=434) | 0.000719 | 0.0219 | **M** **P** **H** **D** **R** **ND** **B** **P** **I** **I** **H** **CB** **S** **P** **E** **B** **P** **KP** **M** **P** **I** **M** **M** |
|  | q9h930-2 | 2 | [**1616**](http://cpdb.molgen.mpg.de/CPDB/showSetDetails?sp=n&st=435) | [**34 (2.1%)**](http://cpdb.molgen.mpg.de/CPDB/showSetDetails?sp=n&st=435) | 0.000719 | 0.0219 | **M** **P** **H** **I** **R** **N** **BP** **I** **I** **H** **C** **S** **P** **EB** **B** **K** **P** **M** **D** |
|  | atg2a_human | 2 | [**2015**](http://cpdb.molgen.mpg.de/CPDB/showSetDetails?sp=n&st=436) | [**40 (2.0%)**](http://cpdb.molgen.mpg.de/CPDB/showSetDetails?sp=n&st=436) | 0.00072 | 0.0219 | **M** **P** **H** **D** **R** **NB** **I** **I** **H** **C** **S** **PE** **B** **P** **B** **K** **PM** |
|  | NLI-interacting factor 2 | 2 | [**3341**](http://cpdb.molgen.mpg.de/CPDB/showSetDetails?sp=n&st=437) | [**59 (1.8%)**](http://cpdb.molgen.mpg.de/CPDB/showSetDetails?sp=n&st=437) | 0.000722 | 0.0219 | **M** **P** **H** **D** **R** **NB** **P** **I** **I** **H** **C** **BS** **P** **E** **B** **P** **K** **PM** **P** **I** **M** |
|  | RNASE8 | 2 | [**60**](http://cpdb.molgen.mpg.de/CPDB/showSetDetails?sp=n&st=438) | [**5 (8.3%)**](http://cpdb.molgen.mpg.de/CPDB/showSetDetails?sp=n&st=438) | 0.00073 | 0.0221 | **B** **S** **M** **I** **R** **H** |
|  | WDR61 | 2 | [**3211**](http://cpdb.molgen.mpg.de/CPDB/showSetDetails?sp=n&st=439) | [**57 (1.8%)**](http://cpdb.molgen.mpg.de/CPDB/showSetDetails?sp=n&st=439) | 0.000745 | 0.0225 | **M** **P** **H** **R** **D** **NB** **P** **I** **I** **H** **C** **BS** **P** **E** **B** **P** **K** **PM** **P** **I** **M** |
|  | class I MHC B38 | 2 | [**1178**](http://cpdb.molgen.mpg.de/CPDB/showSetDetails?sp=n&st=440) | [**27 (2.3%)**](http://cpdb.molgen.mpg.de/CPDB/showSetDetails?sp=n&st=440) | 0.000749 | 0.0226 | **M** **P** **H** **I** **R** **N** **BI** **I** **S** **P** **E** **B** **P** **BK** **P** **M** **D** |
|  | q59fp7_human | 2 | [**2153**](http://cpdb.molgen.mpg.de/CPDB/showSetDetails?sp=n&st=441) | [**42 (2.0%)**](http://cpdb.molgen.mpg.de/CPDB/showSetDetails?sp=n&st=441) | 0.00075 | 0.0226 | **M** **P** **H** **D** **R** **NB** **I** **I** **H** **C** **B** **SP** **E** **B** **P** **K** **P** **PM** |
|  | HLA class II histocompatibility antigen, DQ | 2 | [**2221**](http://cpdb.molgen.mpg.de/CPDB/showSetDetails?sp=n&st=442) | [**43 (1.9%)**](http://cpdb.molgen.mpg.de/CPDB/showSetDetails?sp=n&st=442) | 0.000752 | 0.0226 | **M** **P** **H** **D** **R** **NB** **P** **I** **I** **H** **C** **BS** **P** **E** **B** **P** **P** **MI** **M** **M** |
|  | k2013_human | 2 | [**3504**](http://cpdb.molgen.mpg.de/CPDB/showSetDetails?sp=n&st=443) | [**61 (1.8%)**](http://cpdb.molgen.mpg.de/CPDB/showSetDetails?sp=n&st=443) | 0.000755 | 0.0226 | **M** **P** **H** **D** **R** **NB** **I** **I** **H** **C** **B** **SP** **E** **B** **P** **K** **P** **MP** **I** **M** |
|  | H2AFB1 | 2 | [**3279**](http://cpdb.molgen.mpg.de/CPDB/showSetDetails?sp=n&st=444) | [**58 (1.8%)**](http://cpdb.molgen.mpg.de/CPDB/showSetDetails?sp=n&st=444) | 0.000763 | 0.0228 | **M** **P** **H** **D** **R** **NB** **I** **I** **H** **C** **B** **SP** **E** **B** **P** **K** **P** **MP** **I** **M** **M** |
|  | ATF7IP | 2 | [**2925**](http://cpdb.molgen.mpg.de/CPDB/showSetDetails?sp=n&st=445) | [**53 (1.8%)**](http://cpdb.molgen.mpg.de/CPDB/showSetDetails?sp=n&st=445) | 0.000765 | 0.0228 | **M** **P** **H** **D** **R** **NB** **I** **I** **H** **C** **S** **PE** **B** **P** **B** **K** **PM** **P** **M** **M** |
|  | AKAP11 | 2 | [**2570**](http://cpdb.molgen.mpg.de/CPDB/showSetDetails?sp=n&st=446) | [**48 (1.9%)**](http://cpdb.molgen.mpg.de/CPDB/showSetDetails?sp=n&st=446) | 0.000766 | 0.0228 | **M** **P** **H** **I** **R** **N** **BI** **I** **H** **C** **B** **S** **PE** **B** **P** **K** **P** **M** **PM** **D** |
|  | LAMP1 | 2 | [**3064**](http://cpdb.molgen.mpg.de/CPDB/showSetDetails?sp=n&st=447) | [**55 (1.8%)**](http://cpdb.molgen.mpg.de/CPDB/showSetDetails?sp=n&st=447) | 0.00077 | 0.0228 | **M** **P** **H** **R** **D** **NB** **I** **I** **H** **C** **S** **PE** **B** **P** **B** **K** **PM** **I** **M** |
|  | SERPING1 | 1 | [**204**](http://cpdb.molgen.mpg.de/CPDB/showSetDetails?sp=n&st=448) | [**9 (4.4%)**](http://cpdb.molgen.mpg.de/CPDB/showSetDetails?sp=n&st=448) | 0.000771 | 0.149 | **B** **H** **S** **R** **I** **I** **B** |
|  | BZRAP1 | 2 | [**1367**](http://cpdb.molgen.mpg.de/CPDB/showSetDetails?sp=n&st=449) | [**30 (2.2%)**](http://cpdb.molgen.mpg.de/CPDB/showSetDetails?sp=n&st=449) | 0.000773 | 0.0229 | **M** **P** **H** **D** **R** **NB** **P** **I** **I** **C** **S** **PB** **P** **B** **P** **M** |
|  | TLR2 | 2 | [**3149**](http://cpdb.molgen.mpg.de/CPDB/showSetDetails?sp=n&st=450) | [**56 (1.8%)**](http://cpdb.molgen.mpg.de/CPDB/showSetDetails?sp=n&st=450) | 0.000779 | 0.0229 | **M** **P** **H** **D** **R** **NB** **I** **I** **H** **C** **S** **PE** **B** **P** **B** **K** **PM** **I** **M** **M** |
|  | RPTOR | 2 | [**3136**](http://cpdb.molgen.mpg.de/CPDB/showSetDetails?sp=n&st=451) | [**56 (1.8%)**](http://cpdb.molgen.mpg.de/CPDB/showSetDetails?sp=n&st=451) | 0.000779 | 0.0229 | **M** **P** **H** **I** **R** **N** **BP** **I** **I** **C** **B** **S** **P** **EB** **P** **K** **P** **M** **PM** **D** **M** |
|  | EIF1B | 2 | [**1831**](http://cpdb.molgen.mpg.de/CPDB/showSetDetails?sp=n&st=452) | [**37 (2.0%)**](http://cpdb.molgen.mpg.de/CPDB/showSetDetails?sp=n&st=452) | 0.000783 | 0.0229 | **M** **P** **H** **R** **D** **NB** **I** **I** **H** **C** **S** **PE** **B** **P** **B** **K** **PM** **I** **M** |
|  | rgps2_human | 2 | [**1818**](http://cpdb.molgen.mpg.de/CPDB/showSetDetails?sp=n&st=453) | [**37 (2.0%)**](http://cpdb.molgen.mpg.de/CPDB/showSetDetails?sp=n&st=453) | 0.000783 | 0.0229 | **M** **P** **H** **I** **R** **N** **BP** **I** **I** **H** **C** **B** **SP** **E** **B** **P** **K** **P** **PM** **D** |
|  | PDE4B | 1 | [**164**](http://cpdb.molgen.mpg.de/CPDB/showSetDetails?sp=n&st=454) | [**8 (4.9%)**](http://cpdb.molgen.mpg.de/CPDB/showSetDetails?sp=n&st=454) | 0.000787 | 0.149 | **I** **P** **P** **B** **S** **C** **RH** |
|  | CD46 | 2 | [**712**](http://cpdb.molgen.mpg.de/CPDB/showSetDetails?sp=n&st=455) | [**19 (2.7%)**](http://cpdb.molgen.mpg.de/CPDB/showSetDetails?sp=n&st=455) | 0.000804 | 0.0232 | **M** **P** **H** **I** **R** **N** **BP** **I** **I** **C** **S** **P** **E** **BP** **B** **P** **M** **M** **DM** |
|  | bcl7b_human | 2 | [**2857**](http://cpdb.molgen.mpg.de/CPDB/showSetDetails?sp=n&st=456) | [**52 (1.8%)**](http://cpdb.molgen.mpg.de/CPDB/showSetDetails?sp=n&st=456) | 0.000809 | 0.0232 | **M** **P** **H** **D** **R** **NB** **P** **I** **I** **H** **C** **SP** **E** **B** **P** **B** **K** **PM** **P** **M** |
|  | ATP13A3 | 2 | [**394**](http://cpdb.molgen.mpg.de/CPDB/showSetDetails?sp=n&st=457) | [**13 (3.3%)**](http://cpdb.molgen.mpg.de/CPDB/showSetDetails?sp=n&st=457) | 0.00081 | 0.0232 | **P** **H** **S** **D** **R** **P** **PB** **M** **I** **I** |
|  | hmgx4_human | 2 | [**3721**](http://cpdb.molgen.mpg.de/CPDB/showSetDetails?sp=n&st=458) | [**64 (1.7%)**](http://cpdb.molgen.mpg.de/CPDB/showSetDetails?sp=n&st=458) | 0.000811 | 0.0232 | **M** **P** **H** **R** **D** **NB** **P** **I** **I** **H** **C** **SP** **E** **B** **P** **B** **K** **PM** **P** **I** **M** **M** |
|  | ING2 | 2 | [**2437**](http://cpdb.molgen.mpg.de/CPDB/showSetDetails?sp=n&st=459) | [**46 (1.9%)**](http://cpdb.molgen.mpg.de/CPDB/showSetDetails?sp=n&st=459) | 0.000811 | 0.0232 | **M** **P** **H** **I** **R** **N** **BI** **I** **H** **C** **B** **S** **PE** **B** **P** **K** **P** **M** **PM** **D** |
|  | grin1_human | 2 | [**1825**](http://cpdb.molgen.mpg.de/CPDB/showSetDetails?sp=n&st=460) | [**37 (2.0%)**](http://cpdb.molgen.mpg.de/CPDB/showSetDetails?sp=n&st=460) | 0.000815 | 0.0232 | **M** **P** **H** **I** **R** **N** **BI** **I** **H** **C** **B** **S** **PE** **B** **P** **K** **P** **M** **PM** **D** |
|  | egfl7_human | 2 | [**1432**](http://cpdb.molgen.mpg.de/CPDB/showSetDetails?sp=n&st=461) | [**31 (2.2%)**](http://cpdb.molgen.mpg.de/CPDB/showSetDetails?sp=n&st=461) | 0.000816 | 0.0232 | **M** **P** **H** **D** **R** **NB** **I** **I** **C** **B** **S** **PE** **B** **P** **P** **M** **I** **MM** |
|  | ELL2 | 2 | [**3154**](http://cpdb.molgen.mpg.de/CPDB/showSetDetails?sp=n&st=462) | [**56 (1.8%)**](http://cpdb.molgen.mpg.de/CPDB/showSetDetails?sp=n&st=462) | 0.000816 | 0.0232 | **M** **P** **H** **R** **D** **NB** **I** **I** **H** **C** **B** **SP** **E** **B** **P** **K** **P** **MP** **I** **M** |
|  | CXCR2 | 2 | [**2930**](http://cpdb.molgen.mpg.de/CPDB/showSetDetails?sp=n&st=463) | [**53 (1.8%)**](http://cpdb.molgen.mpg.de/CPDB/showSetDetails?sp=n&st=463) | 0.000816 | 0.0232 | **M** **P** **H** **D** **R** **NB** **P** **I** **I** **H** **C** **BS** **P** **E** **B** **P** **K** **PM** **I** **M** **M** |
|  | h3bnt4_human | 2 | [**2232**](http://cpdb.molgen.mpg.de/CPDB/showSetDetails?sp=n&st=464) | [**43 (1.9%)**](http://cpdb.molgen.mpg.de/CPDB/showSetDetails?sp=n&st=464) | 0.000817 | 0.0232 | **M** **P** **H** **I** **R** **N** **BP** **I** **I** **H** **C** **S** **P** **EB** **P** **B** **K** **P** **MP** **M** **D** **M** |
|  | KLHL36 | 2 | [**1308**](http://cpdb.molgen.mpg.de/CPDB/showSetDetails?sp=n&st=465) | [**29 (2.2%)**](http://cpdb.molgen.mpg.de/CPDB/showSetDetails?sp=n&st=465) | 0.000818 | 0.0232 | **P** **H** **I** **R** **N** **B** **I** **IH** **C** **S** **P** **E** **B** **PB** **K** **P** **M** **D** |
|  | CRIM1 | 2 | [**295**](http://cpdb.molgen.mpg.de/CPDB/showSetDetails?sp=n&st=466) | [**11 (3.7%)**](http://cpdb.molgen.mpg.de/CPDB/showSetDetails?sp=n&st=466) | 0.00082 | 0.0232 | **M** **P** **C** **H** **S** **BB** **N** **R** **P** **P** **B** **PM** **I** **I** |
|  | palm_human | 1 | [**598**](http://cpdb.molgen.mpg.de/CPDB/showSetDetails?sp=n&st=467) | [**17 (2.8%)**](http://cpdb.molgen.mpg.de/CPDB/showSetDetails?sp=n&st=467) | 0.00082 | 0.149 | **I** **B** |
|  | LGALS9 | 2 | [**2304**](http://cpdb.molgen.mpg.de/CPDB/showSetDetails?sp=n&st=468) | [**44 (1.9%)**](http://cpdb.molgen.mpg.de/CPDB/showSetDetails?sp=n&st=468) | 0.000822 | 0.0232 | **M** **P** **H** **D** **R** **NB** **P** **I** **I** **H** **C** **SP** **E** **B** **P** **B** **K** **PM** **I** **M** **M** |
|  | AKT1S1 | 2 | [**2296**](http://cpdb.molgen.mpg.de/CPDB/showSetDetails?sp=n&st=469) | [**44 (1.9%)**](http://cpdb.molgen.mpg.de/CPDB/showSetDetails?sp=n&st=469) | 0.000822 | 0.0232 | **M** **P** **H** **D** **R** **NB** **P** **I** **I** **H** **C** **BS** **P** **E** **B** **P** **K** **PM** **P** **I** **M** |
|  | ADAM10 | 2 | [**3004**](http://cpdb.molgen.mpg.de/CPDB/showSetDetails?sp=n&st=470) | [**54 (1.8%)**](http://cpdb.molgen.mpg.de/CPDB/showSetDetails?sp=n&st=470) | 0.000822 | 0.0232 | **M** **P** **H** **D** **R** **NB** **P** **I** **I** **H** **C** **SP** **E** **B** **P** **B** **K** **PM** **I** **M** **M** |
|  | PNPLA6 | 2 | [**2368**](http://cpdb.molgen.mpg.de/CPDB/showSetDetails?sp=n&st=471) | [**45 (1.9%)**](http://cpdb.molgen.mpg.de/CPDB/showSetDetails?sp=n&st=471) | 0.000825 | 0.0232 | **M** **P** **H** **R** **D** **NB** **I** **I** **C** **B** **S** **PE** **B** **P** **K** **P** **M** **PI** **M** |
|  | q6kcm7-3 | 2 | [**2370**](http://cpdb.molgen.mpg.de/CPDB/showSetDetails?sp=n&st=472) | [**45 (1.9%)**](http://cpdb.molgen.mpg.de/CPDB/showSetDetails?sp=n&st=472) | 0.000825 | 0.0232 | **M** **P** **H** **I** **R** **N** **BP** **I** **I** **H** **C** **S** **P** **EB** **P** **B** **K** **P** **MP** **M** **D** **M** |
|  | TAB2 | 2 | [**3878**](http://cpdb.molgen.mpg.de/CPDB/showSetDetails?sp=n&st=473) | [**66 (1.7%)**](http://cpdb.molgen.mpg.de/CPDB/showSetDetails?sp=n&st=473) | 0.000826 | 0.0232 | **M** **P** **H** **D** **R** **NB** **P** **I** **I** **H** **C** **BS** **P** **E** **B** **P** **K** **PM** **P** **I** **M** **M** |
|  | ITGB3BP | 2 | [**3507**](http://cpdb.molgen.mpg.de/CPDB/showSetDetails?sp=n&st=474) | [**61 (1.7%)**](http://cpdb.molgen.mpg.de/CPDB/showSetDetails?sp=n&st=474) | 0.000826 | 0.0232 | **M** **P** **H** **D** **R** **NB** **I** **I** **H** **C** **S** **PE** **B** **P** **B** **K** **PM** **P** **I** **M** **M** |
|  | GORAB | 1 | [**441**](http://cpdb.molgen.mpg.de/CPDB/showSetDetails?sp=n&st=475) | [**14 (3.2%)**](http://cpdb.molgen.mpg.de/CPDB/showSetDetails?sp=n&st=475) | 0.000828 | 0.149 | **H** **S** **B** **P** **B** **M** **II** |
|  | b3kqf8_human | 1 | [**4**](http://cpdb.molgen.mpg.de/CPDB/showSetDetails?sp=n&st=476) | [**2 (50.0%)**](http://cpdb.molgen.mpg.de/CPDB/showSetDetails?sp=n&st=476) | 0.000832 | 0.149 | **I** |
|  | GALNS oligomer | 2 | [**4**](http://cpdb.molgen.mpg.de/CPDB/showSetDetails?sp=n&st=477) | [**2 (50.0%)**](http://cpdb.molgen.mpg.de/CPDB/showSetDetails?sp=n&st=477) | 0.000832 | 0.0232 | **E** **R** |
|  | CCDC102B | 2 | [**2371**](http://cpdb.molgen.mpg.de/CPDB/showSetDetails?sp=n&st=478) | [**45 (1.9%)**](http://cpdb.molgen.mpg.de/CPDB/showSetDetails?sp=n&st=478) | 0.000832 | 0.0232 | **M** **P** **H** **I** **R** **N** **BI** **I** **H** **C** **S** **P** **EB** **P** **B** **K** **P** **MP** **M** **D** |
|  | mzt1_human | 2 | [**1498**](http://cpdb.molgen.mpg.de/CPDB/showSetDetails?sp=n&st=479) | [**32 (2.1%)**](http://cpdb.molgen.mpg.de/CPDB/showSetDetails?sp=n&st=479) | 0.000837 | 0.0233 | **M** **P** **H** **I** **R** **N** **BP** **I** **I** **H** **C** **S** **P** **EB** **P** **B** **K** **P** **PM** **D** |
|  | ica69_human | 2 | [**494**](http://cpdb.molgen.mpg.de/CPDB/showSetDetails?sp=n&st=480) | [**15 (3.0%)**](http://cpdb.molgen.mpg.de/CPDB/showSetDetails?sp=n&st=480) | 0.000839 | 0.0233 | **M** **C** **H** **S** **D** **BR** **P** **B** **P** **M** **P** **II** **M** |
|  | ETS1 | 2 | [**3006**](http://cpdb.molgen.mpg.de/CPDB/showSetDetails?sp=n&st=481) | [**54 (1.8%)**](http://cpdb.molgen.mpg.de/CPDB/showSetDetails?sp=n&st=481) | 0.000842 | 0.0233 | **M** **P** **H** **I** **R** **N** **BP** **I** **I** **H** **C** **S** **P** **EB** **P** **B** **K** **P** **MP** **M** **D** **M** |
|  | MVD gene | 2 | [**1502**](http://cpdb.molgen.mpg.de/CPDB/showSetDetails?sp=n&st=482) | [**32 (2.1%)**](http://cpdb.molgen.mpg.de/CPDB/showSetDetails?sp=n&st=482) | 0.000846 | 0.0233 | **M** **P** **H** **I** **R** **N** **BP** **I** **I** **H** **C** **B** **SP** **E** **B** **P** **K** **P** **PM** **D** |
|  | DNMT3B | 2 | [**2369**](http://cpdb.molgen.mpg.de/CPDB/showSetDetails?sp=n&st=483) | [**45 (1.9%)**](http://cpdb.molgen.mpg.de/CPDB/showSetDetails?sp=n&st=483) | 0.000847 | 0.0233 | **M** **P** **H** **R** **D** **NB** **I** **I** **H** **C** **S** **PE** **B** **P** **B** **K** **P** **PI** **M** |
|  | FKBP1A | 2 | [**2868**](http://cpdb.molgen.mpg.de/CPDB/showSetDetails?sp=n&st=484) | [**52 (1.8%)**](http://cpdb.molgen.mpg.de/CPDB/showSetDetails?sp=n&st=484) | 0.000849 | 0.0233 | **M** **P** **H** **D** **R** **NB** **I** **I** **H** **C** **B** **SP** **E** **B** **P** **K** **P** **MP** **I** **M** **M** |
|  | COBLL1 | 2 | [**3661**](http://cpdb.molgen.mpg.de/CPDB/showSetDetails?sp=n&st=485) | [**63 (1.7%)**](http://cpdb.molgen.mpg.de/CPDB/showSetDetails?sp=n&st=485) | 0.000849 | 0.0233 | **M** **P** **H** **D** **R** **NB** **P** **I** **I** **H** **C** **BS** **P** **E** **B** **P** **K** **PM** **P** **I** **M** **M** |
|  | BNIP2 | 2 | [**1065**](http://cpdb.molgen.mpg.de/CPDB/showSetDetails?sp=n&st=486) | [**25 (2.4%)**](http://cpdb.molgen.mpg.de/CPDB/showSetDetails?sp=n&st=486) | 0.000852 | 0.0233 | **M** **P** **H** **D** **R** **NB** **P** **I** **I** **H** **C** **SP** **E** **B** **P** **B** **K** **PM** **I** **M** **M** |
|  | spag1_human | 1 | [**93**](http://cpdb.molgen.mpg.de/CPDB/showSetDetails?sp=n&st=487) | [**6 (6.5%)**](http://cpdb.molgen.mpg.de/CPDB/showSetDetails?sp=n&st=487) | 0.000853 | 0.149 | **P** **I** **B** **H** |
|  | SPHK1 | 2 | [**1899**](http://cpdb.molgen.mpg.de/CPDB/showSetDetails?sp=n&st=488) | [**38 (2.0%)**](http://cpdb.molgen.mpg.de/CPDB/showSetDetails?sp=n&st=488) | 0.000854 | 0.0233 | **M** **P** **H** **D** **R** **NB** **P** **I** **I** **H** **C** **BS** **P** **E** **B** **P** **P** **MP** **I** **M** **M** |
|  | TSPYL1 | 2 | [**1505**](http://cpdb.molgen.mpg.de/CPDB/showSetDetails?sp=n&st=489) | [**32 (2.1%)**](http://cpdb.molgen.mpg.de/CPDB/showSetDetails?sp=n&st=489) | 0.000856 | 0.0233 | **P** **H** **I** **R** **N** **B** **I** **IH** **C** **B** **S** **P** **E** **BP** **K** **P** **P** **M** **D** |
|  | TXK | 2 | [**1505**](http://cpdb.molgen.mpg.de/CPDB/showSetDetails?sp=n&st=490) | [**32 (2.1%)**](http://cpdb.molgen.mpg.de/CPDB/showSetDetails?sp=n&st=490) | 0.000856 | 0.0233 | **M** **P** **H** **R** **D** **NB** **I** **I** **H** **C** **S** **PE** **B** **P** **B** **K** **PM** **I** **M** **M** |
|  | EPN2 | 1 | [**548**](http://cpdb.molgen.mpg.de/CPDB/showSetDetails?sp=n&st=491) | [**16 (2.9%)**](http://cpdb.molgen.mpg.de/CPDB/showSetDetails?sp=n&st=491) | 0.000863 | 0.149 | **B** **I** **R** **I** **B** **H** |
|  | PTPRB | 2 | [**3520**](http://cpdb.molgen.mpg.de/CPDB/showSetDetails?sp=n&st=492) | [**61 (1.7%)**](http://cpdb.molgen.mpg.de/CPDB/showSetDetails?sp=n&st=492) | 0.000864 | 0.0234 | **M** **P** **H** **I** **R** **N** **BP** **I** **I** **H** **C** **S** **P** **EB** **P** **B** **K** **P** **MM** **D** **M** |
|  | PLAT(36-562) | 2 | [**1436**](http://cpdb.molgen.mpg.de/CPDB/showSetDetails?sp=n&st=493) | [**31 (2.2%)**](http://cpdb.molgen.mpg.de/CPDB/showSetDetails?sp=n&st=493) | 0.000864 | 0.0234 | **M** **P** **H** **D** **R** **NB** **I** **I** **H** **C** **B** **SP** **E** **B** **P** **P** **MM** **M** |
|  | RABGAP1 | 2 | [**1503**](http://cpdb.molgen.mpg.de/CPDB/showSetDetails?sp=n&st=494) | [**32 (2.1%)**](http://cpdb.molgen.mpg.de/CPDB/showSetDetails?sp=n&st=494) | 0.000865 | 0.0234 | **M** **P** **H** **D** **R** **NB** **I** **I** **H** **C** **B** **SP** **E** **B** **P** **K** **P** **MP** **M** **M** |
|  | CDCA7L | 2 | [**2729**](http://cpdb.molgen.mpg.de/CPDB/showSetDetails?sp=n&st=495) | [**50 (1.8%)**](http://cpdb.molgen.mpg.de/CPDB/showSetDetails?sp=n&st=495) | 0.000871 | 0.0235 | **M** **P** **H** **D** **R** **NB** **P** **I** **I** **C** **S** **PB** **P** **B** **P** **M** **P** **IM** |
|  | smbp2_human | 2 | [**1705**](http://cpdb.molgen.mpg.de/CPDB/showSetDetails?sp=n&st=496) | [**35 (2.1%)**](http://cpdb.molgen.mpg.de/CPDB/showSetDetails?sp=n&st=496) | 0.00088 | 0.0236 | **P** **H** **D** **R** **N** **B** **II** **H** **C** **S** **P** **E** **BP** **B** **K** **P** **M** |
|  | GTF2H4 | 2 | [**2794**](http://cpdb.molgen.mpg.de/CPDB/showSetDetails?sp=n&st=497) | [**51 (1.8%)**](http://cpdb.molgen.mpg.de/CPDB/showSetDetails?sp=n&st=497) | 0.000882 | 0.0236 | **M** **P** **H** **D** **R** **NB** **P** **I** **I** **H** **C** **BS** **P** **E** **B** **P** **K** **PM** **P** **I** **M** **M** |
|  | TOR1A | 2 | [**1968**](http://cpdb.molgen.mpg.de/CPDB/showSetDetails?sp=n&st=498) | [**39 (2.0%)**](http://cpdb.molgen.mpg.de/CPDB/showSetDetails?sp=n&st=498) | 0.000882 | 0.0236 | **P** **H** **D** **R** **N** **B** **II** **H** **C** **P** **P** **E** **BB** **K** **P** **I** **M** **S** **M** |
|  | NDUFB8 | 2 | [**2871**](http://cpdb.molgen.mpg.de/CPDB/showSetDetails?sp=n&st=499) | [**52 (1.8%)**](http://cpdb.molgen.mpg.de/CPDB/showSetDetails?sp=n&st=499) | 0.000884 | 0.0236 | **M** **P** **H** **I** **R** **N** **BP** **I** **I** **H** **C** **B** **SP** **E** **B** **P** **K** **P** **MM** **D** **M** |
|  | MGAT5 | 2 | [**661**](http://cpdb.molgen.mpg.de/CPDB/showSetDetails?sp=n&st=500) | [**18 (2.7%)**](http://cpdb.molgen.mpg.de/CPDB/showSetDetails?sp=n&st=500) | 0.000885 | 0.0236 | **P** **H** **R** **D** **N** **B** **II** **H** **C** **S** **P** **E** **BP** **B** **K** **P** **M** **I** **M** |
|  | NRG4 | 2 | [**1703**](http://cpdb.molgen.mpg.de/CPDB/showSetDetails?sp=n&st=501) | [**35 (2.1%)**](http://cpdb.molgen.mpg.de/CPDB/showSetDetails?sp=n&st=501) | 0.00089 | 0.0237 | **M** **P** **H** **I** **R** **N** **BP** **I** **I** **H** **C** **B** **SP** **E** **B** **P** **K** **P** **MP** **M** **D** **M** |
|  | MRC2 | 2 | [**664**](http://cpdb.molgen.mpg.de/CPDB/showSetDetails?sp=n&st=502) | [**18 (2.7%)**](http://cpdb.molgen.mpg.de/CPDB/showSetDetails?sp=n&st=502) | 0.000901 | 0.0239 | **P** **C** **H** **S** **P** **D** **RN** **P** **P** **B** **P** **M** **II** |
|  | NFX1 | 2 | [**833**](http://cpdb.molgen.mpg.de/CPDB/showSetDetails?sp=n&st=503) | [**21 (2.5%)**](http://cpdb.molgen.mpg.de/CPDB/showSetDetails?sp=n&st=503) | 0.000901 | 0.0239 | **M** **P** **H** **I** **R** **N** **BI** **I** **H** **C** **S** **P** **BP** **B** **K** **P** **M** **PM** **D** |
|  | f19a1_human; PGBD5 | 2 | [**94**](http://cpdb.molgen.mpg.de/CPDB/showSetDetails?sp=n&st=504) | [**6 (6.4%)**](http://cpdb.molgen.mpg.de/CPDB/showSetDetails?sp=n&st=504) | 0.000902 | 0.0239 | **P** **B** **R** **I** **H** |
|  | RCOR3 | 2 | [**3737**](http://cpdb.molgen.mpg.de/CPDB/showSetDetails?sp=n&st=505) | [**64 (1.7%)**](http://cpdb.molgen.mpg.de/CPDB/showSetDetails?sp=n&st=505) | 0.000903 | 0.0239 | **M** **P** **H** **D** **R** **NB** **P** **I** **I** **H** **C** **BS** **P** **E** **B** **P** **K** **PM** **P** **I** **M** **M** |
|  | TMEM179B | 2 | [**2876**](http://cpdb.molgen.mpg.de/CPDB/showSetDetails?sp=n&st=506) | [**52 (1.8%)**](http://cpdb.molgen.mpg.de/CPDB/showSetDetails?sp=n&st=506) | 0.000906 | 0.0239 | **M** **P** **H** **D** **R** **NB** **I** **I** **H** **C** **S** **PE** **B** **P** **B** **K** **PM** **I** **M** **M** |
|  | RAB4A | 2 | [**1968**](http://cpdb.molgen.mpg.de/CPDB/showSetDetails?sp=n&st=507) | [**39 (2.0%)**](http://cpdb.molgen.mpg.de/CPDB/showSetDetails?sp=n&st=507) | 0.000908 | 0.0239 | **M** **P** **H** **R** **D** **NB** **I** **I** **H** **C** **B** **SP** **E** **B** **P** **K** **P** **MP** **M** |
|  | ccd51_human | 2 | [**1133**](http://cpdb.molgen.mpg.de/CPDB/showSetDetails?sp=n&st=508) | [**26 (2.3%)**](http://cpdb.molgen.mpg.de/CPDB/showSetDetails?sp=n&st=508) | 0.000912 | 0.024 | **M** **P** **H** **D** **R** **NB** **P** **I** **I** **H** **C** **BS** **P** **D** **B** **P** **K** **PM** **M** |
|  | STAM | 1 | [**829**](http://cpdb.molgen.mpg.de/CPDB/showSetDetails?sp=n&st=509) | [**21 (2.5%)**](http://cpdb.molgen.mpg.de/CPDB/showSetDetails?sp=n&st=509) | 0.000914 | 0.149 | **H** **B** **S** **P** **R** **D** **NP** **P** **B** **M** **I** **I** |
|  | BHLHE41 gene | 2 | [**3304**](http://cpdb.molgen.mpg.de/CPDB/showSetDetails?sp=n&st=510) | [**58 (1.8%)**](http://cpdb.molgen.mpg.de/CPDB/showSetDetails?sp=n&st=510) | 0.000916 | 0.024 | **M** **P** **H** **I** **R** **N** **BP** **I** **I** **H** **C** **B** **SP** **E** **B** **P** **K** **P** **MP** **M** **D** |
|  | e9pri1_human | 2 | [**1707**](http://cpdb.molgen.mpg.de/CPDB/showSetDetails?sp=n&st=511) | [**35 (2.1%)**](http://cpdb.molgen.mpg.de/CPDB/showSetDetails?sp=n&st=511) | 0.000918 | 0.024 | **M** **P** **H** **I** **R** **N** **BP** **I** **I** **H** **C** **S** **P** **EB** **P** **B** **K** **P** **MP** **M** **D** **M** |
|  | ARHGEF7 | 2 | [**3898**](http://cpdb.molgen.mpg.de/CPDB/showSetDetails?sp=n&st=512) | [**66 (1.7%)**](http://cpdb.molgen.mpg.de/CPDB/showSetDetails?sp=n&st=512) | 0.000918 | 0.024 | **M** **P** **H** **I** **R** **N** **BP** **I** **I** **H** **C** **S** **P** **EB** **P** **B** **K** **P** **MP** **M** **D** **M** |
|  | rabe2_human | 2 | [**1068**](http://cpdb.molgen.mpg.de/CPDB/showSetDetails?sp=n&st=513) | [**25 (2.3%)**](http://cpdb.molgen.mpg.de/CPDB/showSetDetails?sp=n&st=513) | 0.000923 | 0.024 | **P** **C** **H** **B** **S** **P** **RD** **N** **P** **B** **P** **BM** **I** **I** |
|  | GTF2H5 | 2 | [**2659**](http://cpdb.molgen.mpg.de/CPDB/showSetDetails?sp=n&st=514) | [**49 (1.8%)**](http://cpdb.molgen.mpg.de/CPDB/showSetDetails?sp=n&st=514) | 0.000924 | 0.024 | **M** **P** **H** **R** **D** **NB** **P** **I** **I** **H** **C** **BS** **P** **E** **B** **P** **K** **PM** **P** **I** **M** **M** |
|  | RILP | 2 | [**1196**](http://cpdb.molgen.mpg.de/CPDB/showSetDetails?sp=n&st=515) | [**27 (2.3%)**](http://cpdb.molgen.mpg.de/CPDB/showSetDetails?sp=n&st=515) | 0.000929 | 0.0241 | **P** **H** **I** **R** **N** **B** **I** **IC** **S** **P** **E** **B** **P** **KP** **M** **D** |
|  | rnf13_human | 2 | [**1841**](http://cpdb.molgen.mpg.de/CPDB/showSetDetails?sp=n&st=516) | [**37 (2.0%)**](http://cpdb.molgen.mpg.de/CPDB/showSetDetails?sp=n&st=516) | 0.000938 | 0.0241 | **M** **P** **H** **D** **R** **NB** **I** **I** **C** **S** **E** **BP** **B** **P** **I** **M** |
|  | PVRL3 | 2 | [**3161**](http://cpdb.molgen.mpg.de/CPDB/showSetDetails?sp=n&st=517) | [**56 (1.8%)**](http://cpdb.molgen.mpg.de/CPDB/showSetDetails?sp=n&st=517) | 0.000939 | 0.0241 | **M** **P** **H** **R** **D** **NB** **I** **I** **H** **C** **S** **PE** **B** **P** **B** **K** **P** **PI** **M** |
|  | KDELC1 | 2 | [**130**](http://cpdb.molgen.mpg.de/CPDB/showSetDetails?sp=n&st=518) | [**7 (5.4%)**](http://cpdb.molgen.mpg.de/CPDB/showSetDetails?sp=n&st=518) | 0.000939 | 0.0241 | **H** **S** **E** **R** **K** **BM** **I** |
|  | PRC1 | 2 | [**1773**](http://cpdb.molgen.mpg.de/CPDB/showSetDetails?sp=n&st=519) | [**36 (2.0%)**](http://cpdb.molgen.mpg.de/CPDB/showSetDetails?sp=n&st=519) | 0.00094 | 0.0241 | **M** **P** **H** **D** **R** **NB** **P** **I** **I** **C** **S** **PB** **P** **B** **P** **M** **M** |
|  | FIGF | 2 | [**2315**](http://cpdb.molgen.mpg.de/CPDB/showSetDetails?sp=n&st=520) | [**44 (1.9%)**](http://cpdb.molgen.mpg.de/CPDB/showSetDetails?sp=n&st=520) | 0.00094 | 0.0241 | **M** **P** **H** **D** **R** **NB** **P** **I** **I** **H** **C** **BS** **P** **E** **B** **P** **P** **MI** **M** **M** |
|  | CEBPA | 2 | [**3166**](http://cpdb.molgen.mpg.de/CPDB/showSetDetails?sp=n&st=521) | [**56 (1.8%)**](http://cpdb.molgen.mpg.de/CPDB/showSetDetails?sp=n&st=521) | 0.000946 | 0.0242 | **M** **P** **H** **R** **D** **NB** **P** **I** **I** **H** **C** **BS** **P** **E** **B** **P** **K** **PM** **P** **I** **M** **M** |
|  | RAB8B | 2 | [**1708**](http://cpdb.molgen.mpg.de/CPDB/showSetDetails?sp=n&st=522) | [**35 (2.1%)**](http://cpdb.molgen.mpg.de/CPDB/showSetDetails?sp=n&st=522) | 0.000947 | 0.0242 | **M** **P** **H** **I** **R** **N** **BI** **I** **H** **C** **S** **P** **EB** **P** **B** **K** **P** **MP** **M** **D** |
|  | Cut like 1 | 2 | [**1707**](http://cpdb.molgen.mpg.de/CPDB/showSetDetails?sp=n&st=523) | [**35 (2.1%)**](http://cpdb.molgen.mpg.de/CPDB/showSetDetails?sp=n&st=523) | 0.000947 | 0.0242 | **M** **P** **H** **R** **D** **NB** **I** **I** **C** **B** **S** **PB** **P** **P** **M** **P** **I** **M** |
|  | ORM2 | 1 | [**211**](http://cpdb.molgen.mpg.de/CPDB/showSetDetails?sp=n&st=524) | [**9 (4.3%)**](http://cpdb.molgen.mpg.de/CPDB/showSetDetails?sp=n&st=524) | 0.000947 | 0.149 | **R** **I** **B** |
|  | GGC-RAB5B | 1 | [**664**](http://cpdb.molgen.mpg.de/CPDB/showSetDetails?sp=n&st=525) | [**18 (2.7%)**](http://cpdb.molgen.mpg.de/CPDB/showSetDetails?sp=n&st=525) | 0.000948 | 0.149 | **P** **P** **B** **R** **I** **H** |
|  | CCNL2 | 2 | [**3453**](http://cpdb.molgen.mpg.de/CPDB/showSetDetails?sp=n&st=526) | [**60 (1.7%)**](http://cpdb.molgen.mpg.de/CPDB/showSetDetails?sp=n&st=526) | 0.00095 | 0.0242 | **M** **P** **H** **D** **R** **NB** **I** **I** **H** **C** **B** **SP** **E** **B** **P** **K** **P** **MP** **I** **M** |
|  | e7erk7_human | 2 | [**2043**](http://cpdb.molgen.mpg.de/CPDB/showSetDetails?sp=n&st=527) | [**40 (2.0%)**](http://cpdb.molgen.mpg.de/CPDB/showSetDetails?sp=n&st=527) | 0.00096 | 0.0244 | **M** **P** **H** **R** **D** **NB** **I** **I** **H** **C** **S** **PE** **B** **P** **B** **K** **P** **PI** **M** |
|  | KL-2 | 2 | [**1583**](http://cpdb.molgen.mpg.de/CPDB/showSetDetails?sp=n&st=528) | [**33 (2.1%)**](http://cpdb.molgen.mpg.de/CPDB/showSetDetails?sp=n&st=528) | 0.00096 | 0.0244 | **M** **P** **H** **I** **R** **N** **BP** **I** **I** **H** **C** **B** **SP** **E** **B** **P** **K** **P** **MP** **M** **D** **M** |
|  | HLA class I histocompatibility antigen, A-24 alpha chain | 2 | [**1198**](http://cpdb.molgen.mpg.de/CPDB/showSetDetails?sp=n&st=529) | [**27 (2.3%)**](http://cpdb.molgen.mpg.de/CPDB/showSetDetails?sp=n&st=529) | 0.000965 | 0.0244 | **M** **P** **H** **D** **R** **NB** **I** **I** **S** **P** **E** **B** **PB** **K** **P** **P** **I** **M** |
|  | ddx60_human | 2 | [**1978**](http://cpdb.molgen.mpg.de/CPDB/showSetDetails?sp=n&st=530) | [**39 (2.0%)**](http://cpdb.molgen.mpg.de/CPDB/showSetDetails?sp=n&st=530) | 0.000972 | 0.0244 | **M** **P** **H** **I** **R** **N** **BP** **I** **I** **H** **C** **S** **P** **EB** **P** **B** **K** **P** **MP** **M** **D** **M** |
|  | CKS1B | 2 | [**3904**](http://cpdb.molgen.mpg.de/CPDB/showSetDetails?sp=n&st=531) | [**66 (1.7%)**](http://cpdb.molgen.mpg.de/CPDB/showSetDetails?sp=n&st=531) | 0.000972 | 0.0244 | **M** **P** **H** **R** **D** **NB** **I** **I** **H** **C** **B** **SP** **E** **B** **P** **K** **P** **MP** **I** **M** |
|  | q66k14-2 | 2 | [**1387**](http://cpdb.molgen.mpg.de/CPDB/showSetDetails?sp=n&st=532) | [**30 (2.2%)**](http://cpdb.molgen.mpg.de/CPDB/showSetDetails?sp=n&st=532) | 0.000975 | 0.0244 | **P** **H** **I** **R** **B** **I** **I** **HC** **S** **P** **E** **B** **P** **KP** **P** **M** **D** |
|  | capon_human | 2 | [**4131**](http://cpdb.molgen.mpg.de/CPDB/showSetDetails?sp=n&st=533) | [**69 (1.7%)**](http://cpdb.molgen.mpg.de/CPDB/showSetDetails?sp=n&st=533) | 0.000977 | 0.0244 | **M** **P** **H** **R** **D** **NB** **I** **I** **H** **C** **S** **PE** **B** **P** **B** **K** **P** **PI** **M** |
|  | mcri2_human | 2 | [**955**](http://cpdb.molgen.mpg.de/CPDB/showSetDetails?sp=n&st=534) | [**23 (2.4%)**](http://cpdb.molgen.mpg.de/CPDB/showSetDetails?sp=n&st=534) | 0.000977 | 0.0244 | **M** **P** **H** **R** **D** **NB** **I** **I** **C** **S** **P** **BP** **B** **P** **P** **M** |
|  | NID2 | 2 | [**1325**](http://cpdb.molgen.mpg.de/CPDB/showSetDetails?sp=n&st=535) | [**29 (2.2%)**](http://cpdb.molgen.mpg.de/CPDB/showSetDetails?sp=n&st=535) | 0.000978 | 0.0244 | **P** **H** **I** **R** **N** **B** **I** **IC** **S** **P** **B** **P** **B** **PM** **D** **M** |
|  | Apoptosis-associated tyrosine kinase 2 | 2 | [**2250**](http://cpdb.molgen.mpg.de/CPDB/showSetDetails?sp=n&st=536) | [**43 (1.9%)**](http://cpdb.molgen.mpg.de/CPDB/showSetDetails?sp=n&st=536) | 0.000979 | 0.0244 | **M** **P** **H** **I** **R** **N** **BP** **I** **I** **H** **C** **B** **SP** **E** **B** **P** **K** **P** **MP** **M** **D** **M** |
|  | MOSPD2 | 1 | [**719**](http://cpdb.molgen.mpg.de/CPDB/showSetDetails?sp=n&st=537) | [**19 (2.6%)**](http://cpdb.molgen.mpg.de/CPDB/showSetDetails?sp=n&st=537) | 0.000981 | 0.149 | **R** **I** **B** |
|  | RAB42; RAB20 | 1 | [**64**](http://cpdb.molgen.mpg.de/CPDB/showSetDetails?sp=n&st=538) | [**5 (7.8%)**](http://cpdb.molgen.mpg.de/CPDB/showSetDetails?sp=n&st=538) | 0.000981 | 0.149 | **R** |
|  | STT3A | 2 | [**2119**](http://cpdb.molgen.mpg.de/CPDB/showSetDetails?sp=n&st=539) | [**41 (1.9%)**](http://cpdb.molgen.mpg.de/CPDB/showSetDetails?sp=n&st=539) | 0.000982 | 0.0244 | **M** **P** **H** **R** **D** **NB** **I** **I** **H** **C** **S** **PE** **B** **P** **B** **K** **P** **PI** **M** **M** |
|  | daaf5_human | 2 | [**2740**](http://cpdb.molgen.mpg.de/CPDB/showSetDetails?sp=n&st=540) | [**50 (1.8%)**](http://cpdb.molgen.mpg.de/CPDB/showSetDetails?sp=n&st=540) | 0.000985 | 0.0245 | **M** **P** **H** **R** **D** **NB** **I** **I** **H** **C** **B** **SP** **E** **B** **P** **K** **P** **PI** **M** |
|  | PTAFR | 2 | [**3247**](http://cpdb.molgen.mpg.de/CPDB/showSetDetails?sp=n&st=541) | [**57 (1.8%)**](http://cpdb.molgen.mpg.de/CPDB/showSetDetails?sp=n&st=541) | 0.00099 | 0.0245 | **M** **P** **H** **R** **D** **NB** **P** **I** **I** **H** **C** **BS** **P** **E** **B** **P** **K** **PM** **I** **M** **M** |
|  | TSC22D1 gene | 1 | [**301**](http://cpdb.molgen.mpg.de/CPDB/showSetDetails?sp=n&st=542) | [**11 (3.7%)**](http://cpdb.molgen.mpg.de/CPDB/showSetDetails?sp=n&st=542) | 0.000993 | 0.149 | **H** **S** **R** **P** **B** **M** **I** |
|  | MMP7 | 2 | [**1856**](http://cpdb.molgen.mpg.de/CPDB/showSetDetails?sp=n&st=543) | [**37 (2.0%)**](http://cpdb.molgen.mpg.de/CPDB/showSetDetails?sp=n&st=543) | 0.000996 | 0.0246 | **M** **P** **H** **R** **D** **NB** **I** **I** **C** **B** **S** **PB** **P** **P** **P** **I** **M** **M** |
|  | TOR4A | 2 | [**1711**](http://cpdb.molgen.mpg.de/CPDB/showSetDetails?sp=n&st=544) | [**35 (2.1%)**](http://cpdb.molgen.mpg.de/CPDB/showSetDetails?sp=n&st=544) | 0.000997 | 0.0246 | **M** **P** **H** **D** **R** **NB** **I** **I** **H** **C** **B** **SP** **B** **P** **P** **I** **M** **M** |
|  | ABLIM2 | 1 | [**17**](http://cpdb.molgen.mpg.de/CPDB/showSetDetails?sp=n&st=545) | [**3 (17.6%)**](http://cpdb.molgen.mpg.de/CPDB/showSetDetails?sp=n&st=545) | 0.000998 | 0.149 | **B** **S** **I** **R** |
|  | hps5_human | 2 | [**3171**](http://cpdb.molgen.mpg.de/CPDB/showSetDetails?sp=n&st=546) | [**56 (1.8%)**](http://cpdb.molgen.mpg.de/CPDB/showSetDetails?sp=n&st=546) | 0.000998 | 0.0246 | **M** **P** **H** **D** **R** **NB** **I** **I** **H** **C** **S** **PE** **B** **P** **B** **K** **PM** **P** **I** **M** **M** |
| Enriched pathway-based sets [(download)](http://cpdb.molgen.mpg.de/CPDB/downloadEnrSets?typ=p) (show word cloud) | | | | | | | |
|  | | | | | | | |
| [**156** genes (70.0%)](http://cpdb.molgen.mpg.de/CPDB/showTranslation?highlight=Pg) from the input list are present in at least one pathway. The total number of genes present in at least one pathway and identifiable by 'hgnc-symbol' IDs is **12655**. | | | | | | | |
| **select allnone** | **pathway name** | | **set size** | **candidates contained** | **p-value** | **q-value** | **pathway source** |
|  | Human Complement System | | [**92**](http://cpdb.molgen.mpg.de/CPDB/showSetDetails?sp=p&st=0) | [**7 (7.6%)**](http://cpdb.molgen.mpg.de/CPDB/showSetDetails?sp=p&st=0) | 0.000138 | 0.0656 | **Wikipathways** |
|  | Platelet activation, signaling and aggregation | | [**283**](http://cpdb.molgen.mpg.de/CPDB/showSetDetails?sp=p&st=1) | [**11 (3.9%)**](http://cpdb.molgen.mpg.de/CPDB/showSetDetails?sp=p&st=1) | 0.000762 | 0.165 | **Reactome** |
|  | RAB geranylgeranylation | | [**65**](http://cpdb.molgen.mpg.de/CPDB/showSetDetails?sp=p&st=2) | [**5 (7.7%)**](http://cpdb.molgen.mpg.de/CPDB/showSetDetails?sp=p&st=2) | 0.00122 | 0.165 | **Reactome** |
|  | p53 signaling pathway - Homo sapiens (human) | | [**69**](http://cpdb.molgen.mpg.de/CPDB/showSetDetails?sp=p&st=3) | [**5 (7.2%)**](http://cpdb.molgen.mpg.de/CPDB/showSetDetails?sp=p&st=3) | 0.00159 | 0.165 | **KEGG** |
|  | Insulin signaling pathway - Homo sapiens (human) | | [**140**](http://cpdb.molgen.mpg.de/CPDB/showSetDetails?sp=p&st=4) | [**7 (5.0%)**](http://cpdb.molgen.mpg.de/CPDB/showSetDetails?sp=p&st=4) | 0.00173 | 0.165 | **KEGG** |
|  | RNA degradation - Homo sapiens (human) | | [**77**](http://cpdb.molgen.mpg.de/CPDB/showSetDetails?sp=p&st=5) | [**5 (6.5%)**](http://cpdb.molgen.mpg.de/CPDB/showSetDetails?sp=p&st=5) | 0.00258 | 0.197 | **KEGG** |
|  | Complement and coagulation cascades - Homo sapiens (human) | | [**79**](http://cpdb.molgen.mpg.de/CPDB/showSetDetails?sp=p&st=6) | [**5 (6.3%)**](http://cpdb.molgen.mpg.de/CPDB/showSetDetails?sp=p&st=6) | 0.00289 | 0.197 | **KEGG** |
|  | srebp control of lipid synthesis | | [**8**](http://cpdb.molgen.mpg.de/CPDB/showSetDetails?sp=p&st=7) | [**2 (25.0%)**](http://cpdb.molgen.mpg.de/CPDB/showSetDetails?sp=p&st=7) | 0.00403 | 0.197 | **BioCarta** |
|  | Complement and Coagulation Cascades | | [**59**](http://cpdb.molgen.mpg.de/CPDB/showSetDetails?sp=p&st=8) | [**4 (6.8%)**](http://cpdb.molgen.mpg.de/CPDB/showSetDetails?sp=p&st=8) | 0.00598 | 0.197 | **Wikipathways** |
|  | Platelet degranulation | | [**133**](http://cpdb.molgen.mpg.de/CPDB/showSetDetails?sp=p&st=9) | [**6 (4.5%)**](http://cpdb.molgen.mpg.de/CPDB/showSetDetails?sp=p&st=9) | 0.00605 | 0.197 | **Reactome** |
|  | Dengue-2 Interactions with Complement and Coagulation Cascades | | [**60**](http://cpdb.molgen.mpg.de/CPDB/showSetDetails?sp=p&st=10) | [**4 (6.7%)**](http://cpdb.molgen.mpg.de/CPDB/showSetDetails?sp=p&st=10) | 0.00634 | 0.197 | **Wikipathways** |
|  | Hyaluronan uptake and degradation | | [**10**](http://cpdb.molgen.mpg.de/CPDB/showSetDetails?sp=p&st=11) | [**2 (20.0%)**](http://cpdb.molgen.mpg.de/CPDB/showSetDetails?sp=p&st=11) | 0.00637 | 0.197 | **Reactome** |
|  | Negative regulation of activity of TFAP2 (AP-2) family transcription factors | | [**10**](http://cpdb.molgen.mpg.de/CPDB/showSetDetails?sp=p&st=12) | [**2 (20.0%)**](http://cpdb.molgen.mpg.de/CPDB/showSetDetails?sp=p&st=12) | 0.00637 | 0.197 | **Reactome** |
|  | Coregulation of Androgen receptor activity | | [**61**](http://cpdb.molgen.mpg.de/CPDB/showSetDetails?sp=p&st=13) | [**4 (6.6%)**](http://cpdb.molgen.mpg.de/CPDB/showSetDetails?sp=p&st=13) | 0.00672 | 0.197 | **PID** |
|  | Response to elevated platelet cytosolic Ca2+ | | [**138**](http://cpdb.molgen.mpg.de/CPDB/showSetDetails?sp=p&st=14) | [**6 (4.3%)**](http://cpdb.molgen.mpg.de/CPDB/showSetDetails?sp=p&st=14) | 0.00722 | 0.197 | **Reactome** |
|  | Endochondral Ossification | | [**64**](http://cpdb.molgen.mpg.de/CPDB/showSetDetails?sp=p&st=15) | [**4 (6.3%)**](http://cpdb.molgen.mpg.de/CPDB/showSetDetails?sp=p&st=15) | 0.00753 | 0.197 | **Wikipathways** |
|  | Alpha4 beta1 integrin signaling events | | [**34**](http://cpdb.molgen.mpg.de/CPDB/showSetDetails?sp=p&st=16) | [**3 (8.8%)**](http://cpdb.molgen.mpg.de/CPDB/showSetDetails?sp=p&st=16) | 0.00831 | 0.197 | **PID** |
|  | Hyaluronan metabolism | | [**12**](http://cpdb.molgen.mpg.de/CPDB/showSetDetails?sp=p&st=17) | [**2 (16.7%)**](http://cpdb.molgen.mpg.de/CPDB/showSetDetails?sp=p&st=17) | 0.00919 | 0.197 | **Reactome** |
|  | Import of palmitoyl-CoA into the mitochondrial matrix | | [**12**](http://cpdb.molgen.mpg.de/CPDB/showSetDetails?sp=p&st=18) | [**2 (16.7%)**](http://cpdb.molgen.mpg.de/CPDB/showSetDetails?sp=p&st=18) | 0.00919 | 0.197 | **Reactome** |
|  | rac1 cell motility signaling pathway | | [**38**](http://cpdb.molgen.mpg.de/CPDB/showSetDetails?sp=p&st=19) | [**3 (8.3%)**](http://cpdb.molgen.mpg.de/CPDB/showSetDetails?sp=p&st=19) | 0.00974 | 0.197 | **BioCarta** |
| Enriched gene ontology-based sets [(download)](http://cpdb.molgen.mpg.de/CPDB/downloadEnrSets?typ=g) (show word cloud) | | | | | | | |
|  | | | | | | | |
| [**220** genes (98.7%)](http://cpdb.molgen.mpg.de/CPDB/showTranslation?highlight=G) from the input list are present in at least one GO category. The total number of genes present in at least one GO category and identifiable by 'hgnc-symbol' IDs is **18839**. | | | | | | | |
| **select allnone** | **gene ontology term** | **category, level** | **set size** | **candidates contained** | **p-value** | **q-value** |  |
|  | GO:0005737   cytoplasm | CC 3 | [**10661**](http://cpdb.molgen.mpg.de/CPDB/showSetDetails?sp=g&st=0) | [**161 (1.5%)**](http://cpdb.molgen.mpg.de/CPDB/showSetDetails?sp=g&st=0) | 1.74e-07 | 1.46e-05 |  |
|  | GO:0000323   lytic vacuole | CC 5 | [**526**](http://cpdb.molgen.mpg.de/CPDB/showSetDetails?sp=g&st=1) | [**19 (3.6%)**](http://cpdb.molgen.mpg.de/CPDB/showSetDetails?sp=g&st=1) | 1.41e-05 | 0.000354 |  |
|  | GO:0005622   intracellular | CC 2 | [**14076**](http://cpdb.molgen.mpg.de/CPDB/showSetDetails?sp=g&st=2) | [**189 (1.3%)**](http://cpdb.molgen.mpg.de/CPDB/showSetDetails?sp=g&st=2) | 2.32e-05 | 0.00102 |  |
|  | GO:0044424   intracellular part | CC 2 | [**13719**](http://cpdb.molgen.mpg.de/CPDB/showSetDetails?sp=g&st=3) | [**185 (1.4%)**](http://cpdb.molgen.mpg.de/CPDB/showSetDetails?sp=g&st=3) | 3.46e-05 | 0.00102 |  |
|  | GO:0044444   cytoplasmic part | CC 3 | [**8019**](http://cpdb.molgen.mpg.de/CPDB/showSetDetails?sp=g&st=4) | [**122 (1.5%)**](http://cpdb.molgen.mpg.de/CPDB/showSetDetails?sp=g&st=4) | 6.48e-05 | 0.00272 |  |
|  | GO:0032585   multivesicular body membrane | CC 5 | [**9**](http://cpdb.molgen.mpg.de/CPDB/showSetDetails?sp=g&st=5) | [**3 (37.5%)**](http://cpdb.molgen.mpg.de/CPDB/showSetDetails?sp=g&st=5) | 8.43e-05 | 0.00105 |  |
|  | GO:0034285   response to disaccharide | BP 5 | [**9**](http://cpdb.molgen.mpg.de/CPDB/showSetDetails?sp=g&st=6) | [**3 (33.3%)**](http://cpdb.molgen.mpg.de/CPDB/showSetDetails?sp=g&st=6) | 0.000125 | 0.0504 |  |
|  | GO:0005773   vacuole | CC 4 | [**619**](http://cpdb.molgen.mpg.de/CPDB/showSetDetails?sp=g&st=7) | [**19 (3.1%)**](http://cpdb.molgen.mpg.de/CPDB/showSetDetails?sp=g&st=7) | 0.000126 | 0.0076 |  |
|  | GO:0005768   endosome | CC 5 | [**803**](http://cpdb.molgen.mpg.de/CPDB/showSetDetails?sp=g&st=8) | [**22 (2.8%)**](http://cpdb.molgen.mpg.de/CPDB/showSetDetails?sp=g&st=8) | 0.000179 | 0.00149 |  |
|  | GO:0031410   cytoplasmic vesicle | CC 4 | [**1670**](http://cpdb.molgen.mpg.de/CPDB/showSetDetails?sp=g&st=9) | [**36 (2.2%)**](http://cpdb.molgen.mpg.de/CPDB/showSetDetails?sp=g&st=9) | 0.000227 | 0.0076 |  |
|  | GO:0097708   intracellular vesicle | CC 3 | [**1672**](http://cpdb.molgen.mpg.de/CPDB/showSetDetails?sp=g&st=10) | [**36 (2.2%)**](http://cpdb.molgen.mpg.de/CPDB/showSetDetails?sp=g&st=10) | 0.000232 | 0.0065 |  |
|  | GO:0012505   endomembrane system | CC 2 | [**3859**](http://cpdb.molgen.mpg.de/CPDB/showSetDetails?sp=g&st=11) | [**67 (1.7%)**](http://cpdb.molgen.mpg.de/CPDB/showSetDetails?sp=g&st=11) | 0.000252 | 0.00496 |  |
|  | GO:0032633   interleukin-4 production | BP 4 | [**32**](http://cpdb.molgen.mpg.de/CPDB/showSetDetails?sp=g&st=12) | [**4 (12.5%)**](http://cpdb.molgen.mpg.de/CPDB/showSetDetails?sp=g&st=12) | 0.000503 | 0.108 |  |
|  | GO:1903409   reactive oxygen species biosynthetic process | BP 3 | [**86**](http://cpdb.molgen.mpg.de/CPDB/showSetDetails?sp=g&st=13) | [**6 (7.0%)**](http://cpdb.molgen.mpg.de/CPDB/showSetDetails?sp=g&st=13) | 0.000512 | 0.097 |  |
|  | GO:0003299   muscle hypertrophy in response to stress | BP 3 | [**15**](http://cpdb.molgen.mpg.de/CPDB/showSetDetails?sp=g&st=14) | [**3 (20.0%)**](http://cpdb.molgen.mpg.de/CPDB/showSetDetails?sp=g&st=14) | 0.000644 | 0.097 |  |
|  | GO:0014887   cardiac muscle adaptation | BP 4 | [**15**](http://cpdb.molgen.mpg.de/CPDB/showSetDetails?sp=g&st=15) | [**3 (20.0%)**](http://cpdb.molgen.mpg.de/CPDB/showSetDetails?sp=g&st=15) | 0.000644 | 0.108 |  |
|  | GO:0014898   cardiac muscle hypertrophy in response to stress | BP 4 | [**15**](http://cpdb.molgen.mpg.de/CPDB/showSetDetails?sp=g&st=16) | [**3 (20.0%)**](http://cpdb.molgen.mpg.de/CPDB/showSetDetails?sp=g&st=16) | 0.000644 | 0.108 |  |
|  | GO:0010468   regulation of gene expression | BP 5 | [**4304**](http://cpdb.molgen.mpg.de/CPDB/showSetDetails?sp=g&st=17) | [**71 (1.7%)**](http://cpdb.molgen.mpg.de/CPDB/showSetDetails?sp=g&st=17) | 0.000725 | 0.139 |  |
|  | GO:0014888   striated muscle adaptation | BP 3 | [**38**](http://cpdb.molgen.mpg.de/CPDB/showSetDetails?sp=g&st=18) | [**4 (10.5%)**](http://cpdb.molgen.mpg.de/CPDB/showSetDetails?sp=g&st=18) | 0.000978 | 0.0982 |  |
|  | GO:0043209   myelin sheath | CC 2 | [**174**](http://cpdb.molgen.mpg.de/CPDB/showSetDetails?sp=g&st=19) | [**8 (4.6%)**](http://cpdb.molgen.mpg.de/CPDB/showSetDetails?sp=g&st=19) | 0.00103 | 0.0152 |  |
|  | GO:0006869   lipid transport | BP 4 | [**309**](http://cpdb.molgen.mpg.de/CPDB/showSetDetails?sp=g&st=20) | [**11 (3.6%)**](http://cpdb.molgen.mpg.de/CPDB/showSetDetails?sp=g&st=20) | 0.00103 | 0.13 |  |
|  | GO:1903426   regulation of reactive oxygen species biosynthetic process | BP 5 | [**66**](http://cpdb.molgen.mpg.de/CPDB/showSetDetails?sp=g&st=21) | [**5 (7.6%)**](http://cpdb.molgen.mpg.de/CPDB/showSetDetails?sp=g&st=21) | 0.00104 | 0.139 |  |
|  | GO:0043227   membrane-bounded organelle | CC 2 | [**12089**](http://cpdb.molgen.mpg.de/CPDB/showSetDetails?sp=g&st=22) | [**162 (1.3%)**](http://cpdb.molgen.mpg.de/CPDB/showSetDetails?sp=g&st=22) | 0.00132 | 0.0155 |  |
|  | GO:0002520   immune system development | BP 2 | [**822**](http://cpdb.molgen.mpg.de/CPDB/showSetDetails?sp=g&st=23) | [**20 (2.4%)**](http://cpdb.molgen.mpg.de/CPDB/showSetDetails?sp=g&st=23) | 0.00153 | 0.0555 |  |
|  | GO:0031902   late endosome membrane | CC 4 | [**109**](http://cpdb.molgen.mpg.de/CPDB/showSetDetails?sp=g&st=24) | [**6 (5.6%)**](http://cpdb.molgen.mpg.de/CPDB/showSetDetails?sp=g&st=24) | 0.00169 | 0.0376 |  |
|  | GO:0043500   muscle adaptation | BP 2 | [**77**](http://cpdb.molgen.mpg.de/CPDB/showSetDetails?sp=g&st=25) | [**5 (6.5%)**](http://cpdb.molgen.mpg.de/CPDB/showSetDetails?sp=g&st=25) | 0.00207 | 0.0555 |  |
|  | GO:0030097   hemopoiesis | BP 4 | [**729**](http://cpdb.molgen.mpg.de/CPDB/showSetDetails?sp=g&st=26) | [**18 (2.5%)**](http://cpdb.molgen.mpg.de/CPDB/showSetDetails?sp=g&st=26) | 0.00226 | 0.19 |  |
|  | GO:0010876   lipid localization | BP 3 | [**342**](http://cpdb.molgen.mpg.de/CPDB/showSetDetails?sp=g&st=27) | [**11 (3.2%)**](http://cpdb.molgen.mpg.de/CPDB/showSetDetails?sp=g&st=27) | 0.00231 | 0.125 |  |
|  | GO:0002253   activation of immune response | BP 2 | [**557**](http://cpdb.molgen.mpg.de/CPDB/showSetDetails?sp=g&st=28) | [**15 (2.7%)**](http://cpdb.molgen.mpg.de/CPDB/showSetDetails?sp=g&st=28) | 0.00237 | 0.0555 |  |
|  | GO:0043229   intracellular organelle | CC 2 | [**11927**](http://cpdb.molgen.mpg.de/CPDB/showSetDetails?sp=g&st=29) | [**159 (1.3%)**](http://cpdb.molgen.mpg.de/CPDB/showSetDetails?sp=g&st=29) | 0.00239 | 0.0235 |  |
|  | GO:0005548   phospholipid transporter activity | MF 4 | [**49**](http://cpdb.molgen.mpg.de/CPDB/showSetDetails?sp=g&st=30) | [**4 (8.2%)**](http://cpdb.molgen.mpg.de/CPDB/showSetDetails?sp=g&st=30) | 0.00254 | 0.0552 |  |
|  | GO:0044437   vacuolar part | CC 3 | [**400**](http://cpdb.molgen.mpg.de/CPDB/showSetDetails?sp=g&st=31) | [**12 (3.0%)**](http://cpdb.molgen.mpg.de/CPDB/showSetDetails?sp=g&st=31) | 0.0027 | 0.0566 |  |
|  | GO:0051599   response to hydrostatic pressure | BP 3 | [**7**](http://cpdb.molgen.mpg.de/CPDB/showSetDetails?sp=g&st=32) | [**2 (28.6%)**](http://cpdb.molgen.mpg.de/CPDB/showSetDetails?sp=g&st=32) | 0.00274 | 0.125 |  |
|  | GO:0009056   catabolic process | BP 2 | [**1920**](http://cpdb.molgen.mpg.de/CPDB/showSetDetails?sp=g&st=33) | [**36 (1.9%)**](http://cpdb.molgen.mpg.de/CPDB/showSetDetails?sp=g&st=33) | 0.00285 | 0.0555 |  |
|  | GO:0007264   small GTPase mediated signal transduction | BP 4 | [**572**](http://cpdb.molgen.mpg.de/CPDB/showSetDetails?sp=g&st=34) | [**15 (2.6%)**](http://cpdb.molgen.mpg.de/CPDB/showSetDetails?sp=g&st=34) | 0.0029 | 0.19 |  |
|  | GO:0035556   intracellular signal transduction | BP 3 | [**2634**](http://cpdb.molgen.mpg.de/CPDB/showSetDetails?sp=g&st=35) | [**46 (1.7%)**](http://cpdb.molgen.mpg.de/CPDB/showSetDetails?sp=g&st=35) | 0.00291 | 0.125 |  |
|  | GO:0010035   response to inorganic substance | BP 3 | [**515**](http://cpdb.molgen.mpg.de/CPDB/showSetDetails?sp=g&st=36) | [**14 (2.7%)**](http://cpdb.molgen.mpg.de/CPDB/showSetDetails?sp=g&st=36) | 0.00297 | 0.125 |  |
|  | GO:0050778   positive regulation of immune response | BP 5 | [**687**](http://cpdb.molgen.mpg.de/CPDB/showSetDetails?sp=g&st=37) | [**17 (2.5%)**](http://cpdb.molgen.mpg.de/CPDB/showSetDetails?sp=g&st=37) | 0.003 | 0.238 |  |
|  | GO:0032555   purine ribonucleotide binding | MF 4 | [**1877**](http://cpdb.molgen.mpg.de/CPDB/showSetDetails?sp=g&st=38) | [**35 (1.9%)**](http://cpdb.molgen.mpg.de/CPDB/showSetDetails?sp=g&st=38) | 0.00331 | 0.0552 |  |
|  | GO:0097062   dendritic spine maintenance | BP 4 | [**8**](http://cpdb.molgen.mpg.de/CPDB/showSetDetails?sp=g&st=39) | [**2 (25.0%)**](http://cpdb.molgen.mpg.de/CPDB/showSetDetails?sp=g&st=39) | 0.00363 | 0.19 |  |
|  | GO:0035256   G-protein coupled glutamate receptor binding | MF 5 | [**8**](http://cpdb.molgen.mpg.de/CPDB/showSetDetails?sp=g&st=40) | [**2 (25.0%)**](http://cpdb.molgen.mpg.de/CPDB/showSetDetails?sp=g&st=40) | 0.00363 | 0.105 |  |
|  | GO:0017076   purine nucleotide binding | MF 4 | [**1890**](http://cpdb.molgen.mpg.de/CPDB/showSetDetails?sp=g&st=41) | [**35 (1.9%)**](http://cpdb.molgen.mpg.de/CPDB/showSetDetails?sp=g&st=41) | 0.0037 | 0.0552 |  |
|  | GO:0043202   lysosomal lumen | CC 5 | [**88**](http://cpdb.molgen.mpg.de/CPDB/showSetDetails?sp=g&st=42) | [**5 (5.7%)**](http://cpdb.molgen.mpg.de/CPDB/showSetDetails?sp=g&st=42) | 0.00371 | 0.0232 |  |
|  | GO:0032553   ribonucleotide binding | MF 3 | [**1893**](http://cpdb.molgen.mpg.de/CPDB/showSetDetails?sp=g&st=43) | [**35 (1.9%)**](http://cpdb.molgen.mpg.de/CPDB/showSetDetails?sp=g&st=43) | 0.00379 | 0.0989 |  |
|  | GO:1901575   organic substance catabolic process | BP 3 | [**1819**](http://cpdb.molgen.mpg.de/CPDB/showSetDetails?sp=g&st=44) | [**34 (1.9%)**](http://cpdb.molgen.mpg.de/CPDB/showSetDetails?sp=g&st=44) | 0.00394 | 0.125 |  |
|  | GO:0071559   response to transforming growth factor beta | BP 3 | [**216**](http://cpdb.molgen.mpg.de/CPDB/showSetDetails?sp=g&st=45) | [**8 (3.7%)**](http://cpdb.molgen.mpg.de/CPDB/showSetDetails?sp=g&st=45) | 0.00396 | 0.125 |  |
|  | GO:0048534   hematopoietic or lymphoid organ development | BP 3 | [**772**](http://cpdb.molgen.mpg.de/CPDB/showSetDetails?sp=g&st=46) | [**18 (2.3%)**](http://cpdb.molgen.mpg.de/CPDB/showSetDetails?sp=g&st=46) | 0.00414 | 0.125 |  |
|  | GO:0032673   regulation of interleukin-4 production | BP 5 | [**28**](http://cpdb.molgen.mpg.de/CPDB/showSetDetails?sp=g&st=47) | [**3 (10.7%)**](http://cpdb.molgen.mpg.de/CPDB/showSetDetails?sp=g&st=47) | 0.00415 | 0.238 |  |
|  | GO:0035639   purine ribonucleoside triphosphate binding | MF 4 | [**1835**](http://cpdb.molgen.mpg.de/CPDB/showSetDetails?sp=g&st=48) | [**34 (1.9%)**](http://cpdb.molgen.mpg.de/CPDB/showSetDetails?sp=g&st=48) | 0.00418 | 0.0552 |  |
|  | GO:0098805   whole membrane | CC 2 | [**1355**](http://cpdb.molgen.mpg.de/CPDB/showSetDetails?sp=g&st=49) | [**27 (2.0%)**](http://cpdb.molgen.mpg.de/CPDB/showSetDetails?sp=g&st=49) | 0.0045 | 0.0379 |  |
|  | GO:0032550   purine ribonucleoside binding | MF 5 | [**1845**](http://cpdb.molgen.mpg.de/CPDB/showSetDetails?sp=g&st=50) | [**34 (1.9%)**](http://cpdb.molgen.mpg.de/CPDB/showSetDetails?sp=g&st=50) | 0.00455 | 0.105 |  |
|  | GO:0071374   cellular response to parathyroid hormone stimulus | BP 5 | [**9**](http://cpdb.molgen.mpg.de/CPDB/showSetDetails?sp=g&st=51) | [**2 (22.2%)**](http://cpdb.molgen.mpg.de/CPDB/showSetDetails?sp=g&st=51) | 0.00463 | 0.238 |  |
|  | GO:0001883   purine nucleoside binding | MF 4 | [**1848**](http://cpdb.molgen.mpg.de/CPDB/showSetDetails?sp=g&st=52) | [**34 (1.9%)**](http://cpdb.molgen.mpg.de/CPDB/showSetDetails?sp=g&st=52) | 0.00467 | 0.0552 |  |
|  | GO:0032549   ribonucleoside binding | MF 4 | [**1848**](http://cpdb.molgen.mpg.de/CPDB/showSetDetails?sp=g&st=53) | [**34 (1.9%)**](http://cpdb.molgen.mpg.de/CPDB/showSetDetails?sp=g&st=53) | 0.00467 | 0.0552 |  |
|  | GO:0005775   vacuolar lumen | CC 4 | [**93**](http://cpdb.molgen.mpg.de/CPDB/showSetDetails?sp=g&st=54) | [**5 (5.4%)**](http://cpdb.molgen.mpg.de/CPDB/showSetDetails?sp=g&st=54) | 0.00469 | 0.0786 |  |
|  | GO:0001882   nucleoside binding | MF 3 | [**1855**](http://cpdb.molgen.mpg.de/CPDB/showSetDetails?sp=g&st=55) | [**34 (1.8%)**](http://cpdb.molgen.mpg.de/CPDB/showSetDetails?sp=g&st=55) | 0.00495 | 0.0989 |  |
|  | GO:0014897   striated muscle hypertrophy | BP 5 | [**59**](http://cpdb.molgen.mpg.de/CPDB/showSetDetails?sp=g&st=56) | [**4 (6.8%)**](http://cpdb.molgen.mpg.de/CPDB/showSetDetails?sp=g&st=56) | 0.00499 | 0.238 |  |
|  | GO:0031982   vesicle | CC 3 | [**4005**](http://cpdb.molgen.mpg.de/CPDB/showSetDetails?sp=g&st=57) | [**63 (1.6%)**](http://cpdb.molgen.mpg.de/CPDB/showSetDetails?sp=g&st=57) | 0.00528 | 0.0761 |  |
|  | GO:0044433   cytoplasmic vesicle part | CC 3 | [**917**](http://cpdb.molgen.mpg.de/CPDB/showSetDetails?sp=g&st=58) | [**20 (2.2%)**](http://cpdb.molgen.mpg.de/CPDB/showSetDetails?sp=g&st=58) | 0.00544 | 0.0761 |  |
|  | GO:0006952   defense response | BP 3 | [**1591**](http://cpdb.molgen.mpg.de/CPDB/showSetDetails?sp=g&st=59) | [**30 (1.9%)**](http://cpdb.molgen.mpg.de/CPDB/showSetDetails?sp=g&st=59) | 0.00557 | 0.14 |  |
|  | GO:0014896   muscle hypertrophy | BP 4 | [**61**](http://cpdb.molgen.mpg.de/CPDB/showSetDetails?sp=g&st=60) | [**4 (6.6%)**](http://cpdb.molgen.mpg.de/CPDB/showSetDetails?sp=g&st=60) | 0.00562 | 0.19 |  |
|  | GO:0071216   cellular response to biotic stimulus | BP 3 | [**182**](http://cpdb.molgen.mpg.de/CPDB/showSetDetails?sp=g&st=61) | [**7 (3.8%)**](http://cpdb.molgen.mpg.de/CPDB/showSetDetails?sp=g&st=61) | 0.00563 | 0.14 |  |
|  | GO:0036500   ATF6-mediated unfolded protein response | BP 4 | [**10**](http://cpdb.molgen.mpg.de/CPDB/showSetDetails?sp=g&st=62) | [**2 (20.0%)**](http://cpdb.molgen.mpg.de/CPDB/showSetDetails?sp=g&st=62) | 0.00574 | 0.19 |  |
|  | GO:0003211   cardiac ventricle formation | BP 4 | [**10**](http://cpdb.molgen.mpg.de/CPDB/showSetDetails?sp=g&st=63) | [**2 (20.0%)**](http://cpdb.molgen.mpg.de/CPDB/showSetDetails?sp=g&st=63) | 0.00574 | 0.19 |  |
|  | GO:0072593   reactive oxygen species metabolic process | BP 3 | [**232**](http://cpdb.molgen.mpg.de/CPDB/showSetDetails?sp=g&st=64) | [**8 (3.4%)**](http://cpdb.molgen.mpg.de/CPDB/showSetDetails?sp=g&st=64) | 0.00604 | 0.14 |  |
|  | GO:0042127   regulation of cell proliferation | BP 4 | [**1532**](http://cpdb.molgen.mpg.de/CPDB/showSetDetails?sp=g&st=65) | [**29 (1.9%)**](http://cpdb.molgen.mpg.de/CPDB/showSetDetails?sp=g&st=65) | 0.00617 | 0.19 |  |
|  | GO:0043502   regulation of muscle adaptation | BP 4 | [**63**](http://cpdb.molgen.mpg.de/CPDB/showSetDetails?sp=g&st=66) | [**4 (6.3%)**](http://cpdb.molgen.mpg.de/CPDB/showSetDetails?sp=g&st=66) | 0.00629 | 0.19 |  |
|  | GO:0044724   single-organism carbohydrate catabolic process | BP 4 | [**142**](http://cpdb.molgen.mpg.de/CPDB/showSetDetails?sp=g&st=67) | [**6 (4.2%)**](http://cpdb.molgen.mpg.de/CPDB/showSetDetails?sp=g&st=67) | 0.00651 | 0.19 |  |
|  | GO:0002449   lymphocyte mediated immunity | BP 4 | [**286**](http://cpdb.molgen.mpg.de/CPDB/showSetDetails?sp=g&st=68) | [**9 (3.1%)**](http://cpdb.molgen.mpg.de/CPDB/showSetDetails?sp=g&st=68) | 0.00661 | 0.19 |  |
|  | GO:0002460   adaptive immune response based on somatic recombination of immune receptors built from immunoglobulin superfamily domains | BP 4 | [**287**](http://cpdb.molgen.mpg.de/CPDB/showSetDetails?sp=g&st=69) | [**9 (3.1%)**](http://cpdb.molgen.mpg.de/CPDB/showSetDetails?sp=g&st=69) | 0.00676 | 0.19 |  |
|  | GO:0034374   low-density lipoprotein particle remodeling | BP 5 | [**11**](http://cpdb.molgen.mpg.de/CPDB/showSetDetails?sp=g&st=70) | [**2 (18.2%)**](http://cpdb.molgen.mpg.de/CPDB/showSetDetails?sp=g&st=70) | 0.00697 | 0.238 |  |
|  | GO:0032354   response to follicle-stimulating hormone | BP 5 | [**11**](http://cpdb.molgen.mpg.de/CPDB/showSetDetails?sp=g&st=71) | [**2 (18.2%)**](http://cpdb.molgen.mpg.de/CPDB/showSetDetails?sp=g&st=71) | 0.00697 | 0.238 |  |
|  | GO:0044248   cellular catabolic process | BP 3 | [**1540**](http://cpdb.molgen.mpg.de/CPDB/showSetDetails?sp=g&st=72) | [**29 (1.9%)**](http://cpdb.molgen.mpg.de/CPDB/showSetDetails?sp=g&st=72) | 0.00698 | 0.144 |  |
|  | GO:0034405   response to fluid shear stress | BP 3 | [**34**](http://cpdb.molgen.mpg.de/CPDB/showSetDetails?sp=g&st=73) | [**3 (8.8%)**](http://cpdb.molgen.mpg.de/CPDB/showSetDetails?sp=g&st=73) | 0.0072 | 0.144 |  |
|  | GO:0010038   response to metal ion | BP 4 | [**345**](http://cpdb.molgen.mpg.de/CPDB/showSetDetails?sp=g&st=74) | [**10 (2.9%)**](http://cpdb.molgen.mpg.de/CPDB/showSetDetails?sp=g&st=74) | 0.00739 | 0.19 |  |
|  | GO:0060205   cytoplasmic vesicle lumen | CC 4 | [**104**](http://cpdb.molgen.mpg.de/CPDB/showSetDetails?sp=g&st=75) | [**5 (4.8%)**](http://cpdb.molgen.mpg.de/CPDB/showSetDetails?sp=g&st=75) | 0.0075 | 0.0924 |  |
|  | GO:0010008   endosome membrane | CC 3 | [**402**](http://cpdb.molgen.mpg.de/CPDB/showSetDetails?sp=g&st=76) | [**11 (2.7%)**](http://cpdb.molgen.mpg.de/CPDB/showSetDetails?sp=g&st=76) | 0.0077 | 0.0851 |  |
|  | GO:0009889   regulation of biosynthetic process | BP 4 | [**4308**](http://cpdb.molgen.mpg.de/CPDB/showSetDetails?sp=g&st=77) | [**66 (1.5%)**](http://cpdb.molgen.mpg.de/CPDB/showSetDetails?sp=g&st=77) | 0.00783 | 0.19 |  |
|  | GO:0031983   vesicle lumen | CC 3 | [**106**](http://cpdb.molgen.mpg.de/CPDB/showSetDetails?sp=g&st=78) | [**5 (4.7%)**](http://cpdb.molgen.mpg.de/CPDB/showSetDetails?sp=g&st=78) | 0.00811 | 0.0851 |  |
|  | GO:0031349   positive regulation of defense response | BP 5 | [**405**](http://cpdb.molgen.mpg.de/CPDB/showSetDetails?sp=g&st=79) | [**11 (2.7%)**](http://cpdb.molgen.mpg.de/CPDB/showSetDetails?sp=g&st=79) | 0.00826 | 0.238 |  |
|  | GO:0005829   cytosol | CC 4 | [**3466**](http://cpdb.molgen.mpg.de/CPDB/showSetDetails?sp=g&st=80) | [**55 (1.6%)**](http://cpdb.molgen.mpg.de/CPDB/showSetDetails?sp=g&st=80) | 0.00827 | 0.0924 |  |
|  | GO:0003207   cardiac chamber formation | BP 3 | [**12**](http://cpdb.molgen.mpg.de/CPDB/showSetDetails?sp=g&st=81) | [**2 (16.7%)**](http://cpdb.molgen.mpg.de/CPDB/showSetDetails?sp=g&st=81) | 0.0083 | 0.156 |  |
|  | GO:0051787   misfolded protein binding | MF 3 | [**12**](http://cpdb.molgen.mpg.de/CPDB/showSetDetails?sp=g&st=82) | [**2 (16.7%)**](http://cpdb.molgen.mpg.de/CPDB/showSetDetails?sp=g&st=82) | 0.0083 | 0.0989 |  |
|  | GO:0042118   endothelial cell activation | BP 4 | [**12**](http://cpdb.molgen.mpg.de/CPDB/showSetDetails?sp=g&st=83) | [**2 (16.7%)**](http://cpdb.molgen.mpg.de/CPDB/showSetDetails?sp=g&st=83) | 0.0083 | 0.19 |  |
|  | GO:0051172   negative regulation of nitrogen compound metabolic process | BP 5 | [**1490**](http://cpdb.molgen.mpg.de/CPDB/showSetDetails?sp=g&st=84) | [**28 (1.9%)**](http://cpdb.molgen.mpg.de/CPDB/showSetDetails?sp=g&st=84) | 0.00836 | 0.238 |  |
|  | GO:0016052   carbohydrate catabolic process | BP 4 | [**150**](http://cpdb.molgen.mpg.de/CPDB/showSetDetails?sp=g&st=85) | [**6 (4.0%)**](http://cpdb.molgen.mpg.de/CPDB/showSetDetails?sp=g&st=85) | 0.00843 | 0.19 |  |
|  | GO:1903035   negative regulation of response to wounding | BP 5 | [**69**](http://cpdb.molgen.mpg.de/CPDB/showSetDetails?sp=g&st=86) | [**4 (5.8%)**](http://cpdb.molgen.mpg.de/CPDB/showSetDetails?sp=g&st=86) | 0.00866 | 0.238 |  |
|  | GO:0001917   photoreceptor inner segment | CC 3 | [**37**](http://cpdb.molgen.mpg.de/CPDB/showSetDetails?sp=g&st=87) | [**3 (8.1%)**](http://cpdb.molgen.mpg.de/CPDB/showSetDetails?sp=g&st=87) | 0.00911 | 0.0851 |  |
|  | GO:0002684   positive regulation of immune system process | BP 4 | [**963**](http://cpdb.molgen.mpg.de/CPDB/showSetDetails?sp=g&st=88) | [**20 (2.1%)**](http://cpdb.molgen.mpg.de/CPDB/showSetDetails?sp=g&st=88) | 0.00913 | 0.19 |  |
|  | GO:0099503   secretory vesicle | CC 5 | [**468**](http://cpdb.molgen.mpg.de/CPDB/showSetDetails?sp=g&st=89) | [**12 (2.6%)**](http://cpdb.molgen.mpg.de/CPDB/showSetDetails?sp=g&st=89) | 0.00915 | 0.0458 |  |
|  | GO:0005319   lipid transporter activity | MF 3 | [**110**](http://cpdb.molgen.mpg.de/CPDB/showSetDetails?sp=g&st=90) | [**5 (4.5%)**](http://cpdb.molgen.mpg.de/CPDB/showSetDetails?sp=g&st=90) | 0.00944 | 0.0989 |  |
|  | GO:0000166   nucleotide binding | MF 3 | [**2373**](http://cpdb.molgen.mpg.de/CPDB/showSetDetails?sp=g&st=91) | [**40 (1.7%)**](http://cpdb.molgen.mpg.de/CPDB/showSetDetails?sp=g&st=91) | 0.00951 | 0.0989 |  |
|  | GO:1901265   nucleoside phosphate binding | MF 3 | [**2374**](http://cpdb.molgen.mpg.de/CPDB/showSetDetails?sp=g&st=92) | [**40 (1.7%)**](http://cpdb.molgen.mpg.de/CPDB/showSetDetails?sp=g&st=92) | 0.00957 | 0.0989 |  |
|  | GO:0008284   positive regulation of cell proliferation | BP 5 | [**839**](http://cpdb.molgen.mpg.de/CPDB/showSetDetails?sp=g&st=93) | [**18 (2.1%)**](http://cpdb.molgen.mpg.de/CPDB/showSetDetails?sp=g&st=93) | 0.00962 | 0.238 |  |
|  | GO:0010467   gene expression | BP 4 | [**5310**](http://cpdb.molgen.mpg.de/CPDB/showSetDetails?sp=g&st=94) | [**78 (1.5%)**](http://cpdb.molgen.mpg.de/CPDB/showSetDetails?sp=g&st=94) | 0.00972 | 0.19 |  |
|  | GO:0071107   response to parathyroid hormone | BP 4 | [**13**](http://cpdb.molgen.mpg.de/CPDB/showSetDetails?sp=g&st=95) | [**2 (15.4%)**](http://cpdb.molgen.mpg.de/CPDB/showSetDetails?sp=g&st=95) | 0.00973 | 0.19 |  |
|  | GO:0071985   multivesicular body sorting pathway | BP 4 | [**13**](http://cpdb.molgen.mpg.de/CPDB/showSetDetails?sp=g&st=96) | [**2 (15.4%)**](http://cpdb.molgen.mpg.de/CPDB/showSetDetails?sp=g&st=96) | 0.00973 | 0.19 |  |
|  | GO:0010885   regulation of cholesterol storage | BP 4 | [**13**](http://cpdb.molgen.mpg.de/CPDB/showSetDetails?sp=g&st=97) | [**2 (15.4%)**](http://cpdb.molgen.mpg.de/CPDB/showSetDetails?sp=g&st=97) | 0.00973 | 0.19 |  |
|  | GO:0015936   coenzyme A metabolic process | BP 5 | [**13**](http://cpdb.molgen.mpg.de/CPDB/showSetDetails?sp=g&st=98) | [**2 (15.4%)**](http://cpdb.molgen.mpg.de/CPDB/showSetDetails?sp=g&st=98) | 0.00973 | 0.238 |  |
|  | GO:0017127   cholesterol transporter activity | MF 5 | [**13**](http://cpdb.molgen.mpg.de/CPDB/showSetDetails?sp=g&st=99) | [**2 (15.4%)**](http://cpdb.molgen.mpg.de/CPDB/showSetDetails?sp=g&st=99) | 0.00973 | 0.118 |  |
|  | GO:0097367   carbohydrate derivative binding | MF 2 | [**2230**](http://cpdb.molgen.mpg.de/CPDB/showSetDetails?sp=g&st=100) | [**38 (1.7%)**](http://cpdb.molgen.mpg.de/CPDB/showSetDetails?sp=g&st=100) | 0.00982 | 0.132 |  |
|  | GO:0010605   negative regulation of macromolecule metabolic process | BP 5 | [**2297**](http://cpdb.molgen.mpg.de/CPDB/showSetDetails?sp=g&st=101) | [**39 (1.7%)**](http://cpdb.molgen.mpg.de/CPDB/showSetDetails?sp=g&st=101) | 0.00997 | 0.238 |  |
| Enriched protein complex-based sets [(download)](http://cpdb.molgen.mpg.de/CPDB/downloadEnrSets?typ=c) | | | | | | | |
| [**111** genes (49.8%)](http://cpdb.molgen.mpg.de/CPDB/showTranslation?highlight=C) from the input list are present in at least one protein complex. The total number of genes present in at least one protein complex and identifiable by 'hgnc-symbol' IDs is **9211**. | | | | | | | |
| **select allnone** | **complex name** | | **set size** | **candidates contained** | **p-value** | **q-value** | **complex source** |
|  | factor XIIa:C1Inh | | [**2**](http://cpdb.molgen.mpg.de/CPDB/showSetDetails?sp=c&st=0) | [**2 (100.0%)**](http://cpdb.molgen.mpg.de/CPDB/showSetDetails?sp=c&st=0) | 0.000144 | 0.00036 | **Reactome** |
|  | HDAC4/MEF2C | | [**2**](http://cpdb.molgen.mpg.de/CPDB/showSetDetails?sp=c&st=1) | [**2 (100.0%)**](http://cpdb.molgen.mpg.de/CPDB/showSetDetails?sp=c&st=1) | 0.000144 | 0.00036 | **PID** |
|  | 3xOX40:OX40L trimer | | [**2**](http://cpdb.molgen.mpg.de/CPDB/showSetDetails?sp=c&st=2) | [**2 (100.0%)**](http://cpdb.molgen.mpg.de/CPDB/showSetDetails?sp=c&st=2) | 0.000144 | 0.00036 | **Reactome** |
|  | HDAC4/Ubc9 | | [**2**](http://cpdb.molgen.mpg.de/CPDB/showSetDetails?sp=c&st=3) | [**2 (100.0%)**](http://cpdb.molgen.mpg.de/CPDB/showSetDetails?sp=c&st=3) | 0.000144 | 0.00036 | **PID** |
|  | Decapping complex | | [**5**](http://cpdb.molgen.mpg.de/CPDB/showSetDetails?sp=c&st=4) | [**2 (40.0%)**](http://cpdb.molgen.mpg.de/CPDB/showSetDetails?sp=c&st=4) | 0.00141 | 0.00281 | **CORUM** |
|  | DCP1-DCP2 Decapping Complex | | [**6**](http://cpdb.molgen.mpg.de/CPDB/showSetDetails?sp=c&st=5) | [**2 (33.3%)**](http://cpdb.molgen.mpg.de/CPDB/showSetDetails?sp=c&st=5) | 0.00209 | 0.00349 | **Reactome** |

Formularende
